# A new horned and long-necked herbivorous stem-archosaur from the Middle Triassic of India

**DOI:** 10.1038/s41598-017-08658-8

**Published:** 2017-08-21

**Authors:** Saradee Sengupta, Martín D. Ezcurra, Saswati Bandyopadhyay

**Affiliations:** 1 Geological Studies Unit, Indian Statistical Institute, 203, B. T. Road, Kolkata, 700108 India; 2 Durgapur Govt. College, J. L. N. Road, Durgapur, 713214 India; 3 Sección Paleontología de Vertebrados, CONICET–Museo Argentino de Ciencias Naturales “Bernardino Rivadavia”, Avenida Ángel Gallardo 470, Buenos Aires, C1405DJR Argentina

## Abstract

The early evolution of archosauromorphs (bird- and crocodile-line archosaurs and stem-archosaurs) represents an important case of adaptive radiation that occurred in the aftermath of the Permo-Triassic mass extinction. Here we enrich the early archosauromorph record with the description of a moderately large (3–4 m in total length), herbivorous new allokotosaurian, *Shringasaurus indicus*, from the early Middle Triassic of India. The most striking feature of *Shringasaurus indicus* is the presence of a pair of large supraorbital horns that resemble those of some ceratopsid dinosaurs. The presence of horns in the new species is dimorphic and, as occurs in horned extant bovid mammals, these structures were probably sexually selected and used as weapons in intraspecific combats. The relatively large size and unusual anatomy of *Shringasaurus indicus* broadens the morphological diversity of Early–Middle Triassic tetrapods and complements the understanding of the evolutionary mechanisms involved in the early archosauromorph diversification.

## Introduction

**Figure 1:**
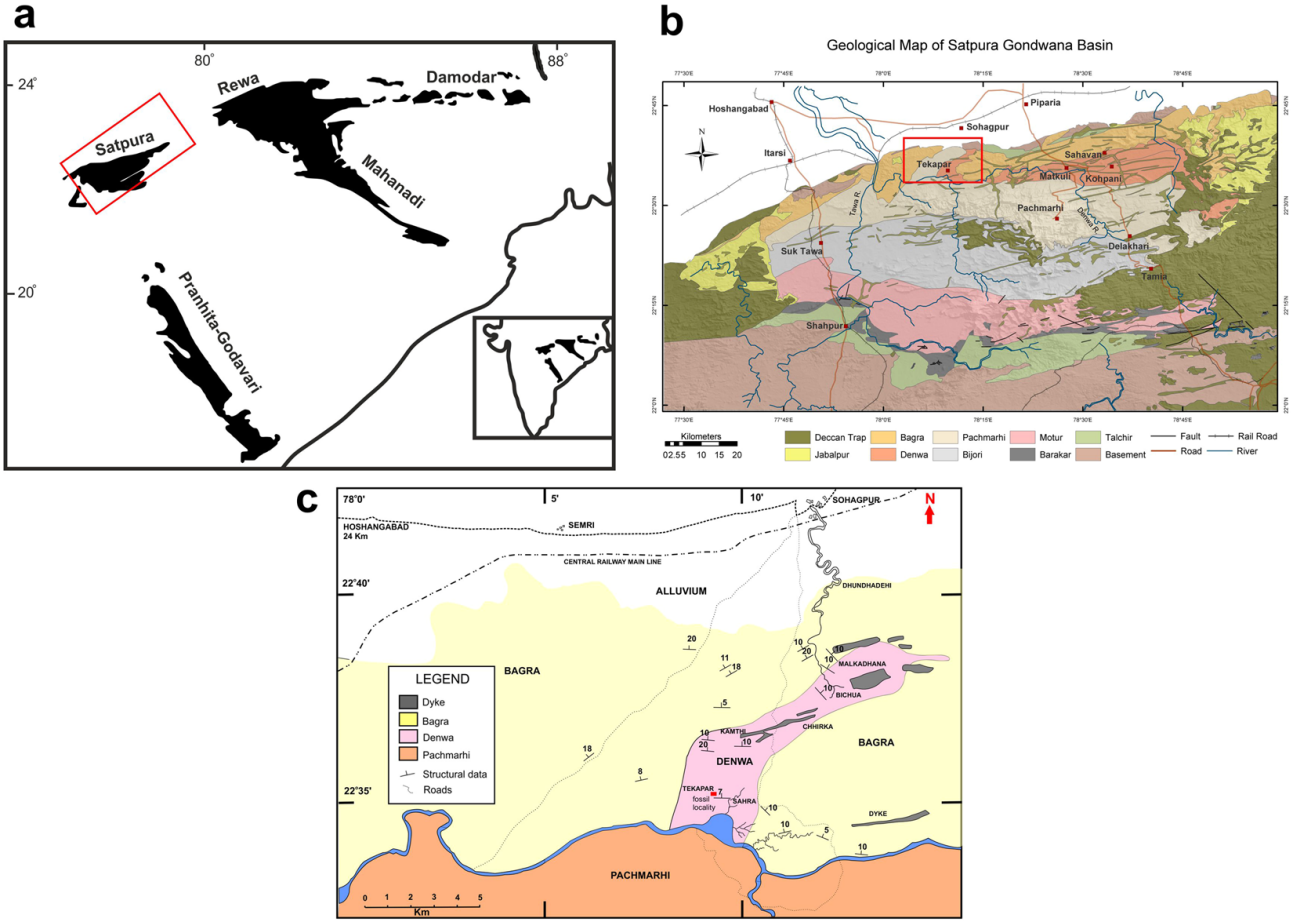
Geographic and geological occurrence of *Shringasaurus indicus* gen. et sp. nov. (**a**) Map of the major Gondwana basins of peninsular India (after^45^), in which the red rectangle indicates the Satpura Basin. (**b**) Complete geological map of the Satpura Gondwana Basin (after^45^). (**c**) Close up of the geology of the area (marked in red rectangle in (**b**) from where *Shringasaurus* was collected (after^9^). Note that the *Shringasaurus* bone-bed is in the Denwa Formation and the red rectangle marked in the map indicates the *Shringasaurus* locality.

The evolutionary radiation of archosauromorphs (archosaurs – crocodylians and dinosaurs – and several extinct stem-clades) in the aftermath of the catastrophic Permo-Triassic mass extinction (~252 Ma) contributed to reshape Mesozoic terrestrial ecosystems and lead to the dominance of dinosaurs^1, 2^. As part of the outstanding diversification of dinosaurs, multiple theropods and ornithischians (e.g. ceratosaurians, oviraptorosaurians, hadrosaurids, ceratopsids) developed elaborate cranial structures, including bony weapons and ornaments^3–7^. Cranial weapons have been considered exclusive of dinosaurs during the Mesozoic and generally interpreted as sexually selected traits and evidence of social behaviour^6, 7^. Here we describe a new herbivorous allokotosaurian stem-archosaur, *Shringasaurus indicus* gen. et sp. nov., from the early Middle Triassic of central India (Fig. 1) that possesses a pair of anterodorsally projecting and sub-conical supraorbital horns, closely resembling those of some ceratopsid dinosaurs. The presence of horns in *Shringasaurus indicus* is dimorphic and as in horned extant mammals, these structures were probably used as weapons in intrasexual combats driven by sexual selection^8^. *Shringasaurus indicus* expands the ecomorphotypes recorded during the early diversification of archosauromorphs and shows that morphologies driven by sexual selection were also involved in this macroevolutionary process.

## Results

### Geological and palaeontological background

The holotype and referred specimens of *Shringasaurus indicus* have been collected from the Denwa Formation of the Satpura Gondwana Basin^9^ (Fig. 1). The Denwa Formation overlies the Lower Triassic Pachmarhi Formation and is overlain unconformably by the Cretaceous Bagra Formation. Maulik *et al*.^10^ divided the Denwa Formation into lower and upper parts on the basis of their lithology. The lower Denwa comprises multistoreyed, 3–15 metres thick, sheet-like medium to fine grained sandstone bodies interleaved by red mudstones. By contrast, the upper Denwa is a mudstone-dominated unit characterised by the presence of layers of ribbon-shaped channel-fill bodies and sandy to heterolithic sheet sandstones encased within mudstones^10^. The sandy or muddy heterolithic sheets and the red mudstones represent rapidly emplaced splay deposits and slowly accumulated floodplain deposits, respectively^11^. The upper unit of the Denwa Formation has been interpreted as deposited by an anabranching fluvial system^11^.

The fossil bones of *Shringasaurus indicus* were recovered from a red mudstone in the upper part of the Denwa Formation. At least seven individuals of different ontogenetic stages were excavated from an area of 25 square metres. Most of them were disarticulated, with exception of a partially articulated skeleton.

The vertebrate fossil assemblage of the Denwa Formation includes the dipnoan *Ceratodus* sp., the capitosaurid *Paracyclotosaurus crookshanki*, the mastodonsaurid *Cherninia denwai*, an undescribed brachyopid, a lonchorhynchine trematosaurid, an undescribed rhynchosaurid rhynchosaur, and small to large-sized dicynodonts^9^. In the nineteenth century, the Denwa Formation was considered as Late Triassic in age based on the presence of a partial skull bone originally assigned to the temnospondyl genus “*Mastodontosaurus*”^12^. Later, Chatterjee & Roy-Chowdhury^13^ suggested a late Early Triassic to early Middle Triassic age; an assignment also supported by Mukherjee & Sengupta^14^ on the basis of the recovery of additional temnospondyl remains that they assigned to *Parotosuchus*. Nandi & Raha^15^ suggested that the carboniferous shale of the Denwa Formation could be given a Late Triassic age based on its microfloral assemblage. Veevers & Tewari^16^ assigned a Middle Triassic (Anisian to early Ladinian) age to the Denwa Formation on the basis of its vertebrate assemblage, but without giving further details. Bandyopadhyay & Sengupta^17^ proposed an early Anisian age to the upper part of the Denwa Formation based on its brachyopid temnospondyl assemblage and also suggested a late Spathian to earliest Anisian age for the lower part of the unit. Abdala *et al*.^18^ also recognized that the upper Denwa Formation can be assigned to the early Anisian based on the presence of a brachyopid temnospondyl related to *Hadrokkosaurus bradyi* from the lower Anisian Holbrook Member of the Moenkopi Formation, USA. However, those authors also pointed out that the Denwa Formation can be directly correlated with the late Anisian subzone C of the *Cynognathus* Assemblage Zone of South Africa and the approximately coeval Ashfield Shale of the Wianamatta Group of the Sydney Basin (Australia) based on the presence of the temnospondyl genus *Paracyclotosaurus*. Damiani (in Abdala *et al*.^18^) argued that the presence of *Paracyclotosaurus* and the mastodonsaurid *Cherninia denwai* in the upper Denwa Formation indicate that a younger age (i.e. latest Anisian) is also possible for this unit. Unfortunately, the archosauromorph content of the formation currently does not shed light on the age of the unit. As a result, here we consider the upper Denwa Formation as broadly Anisian in age.

### Systematic Palaeontology

Diapsida Osborn, 1903

Archosauromorpha Huene, 1946 sensu Dilkes^19^

Allokotosauria Nesbitt *et al*., 2015

Azendohsauridae Nesbitt *et al*., 2015

***Shringasaurus indicus*** **gen**. et sp. nov.

#### Etymology

‘Śṛṅga’ (Shringa), horn (ancient Sanskrit), and ‘sauros’ (σαῦρος), lizard (ancient Greek), referring to the horned skull; ‘indicus’, Indian (Latin English), refers to the country where such species was discovered.

#### Holotype

**Figure 2:**
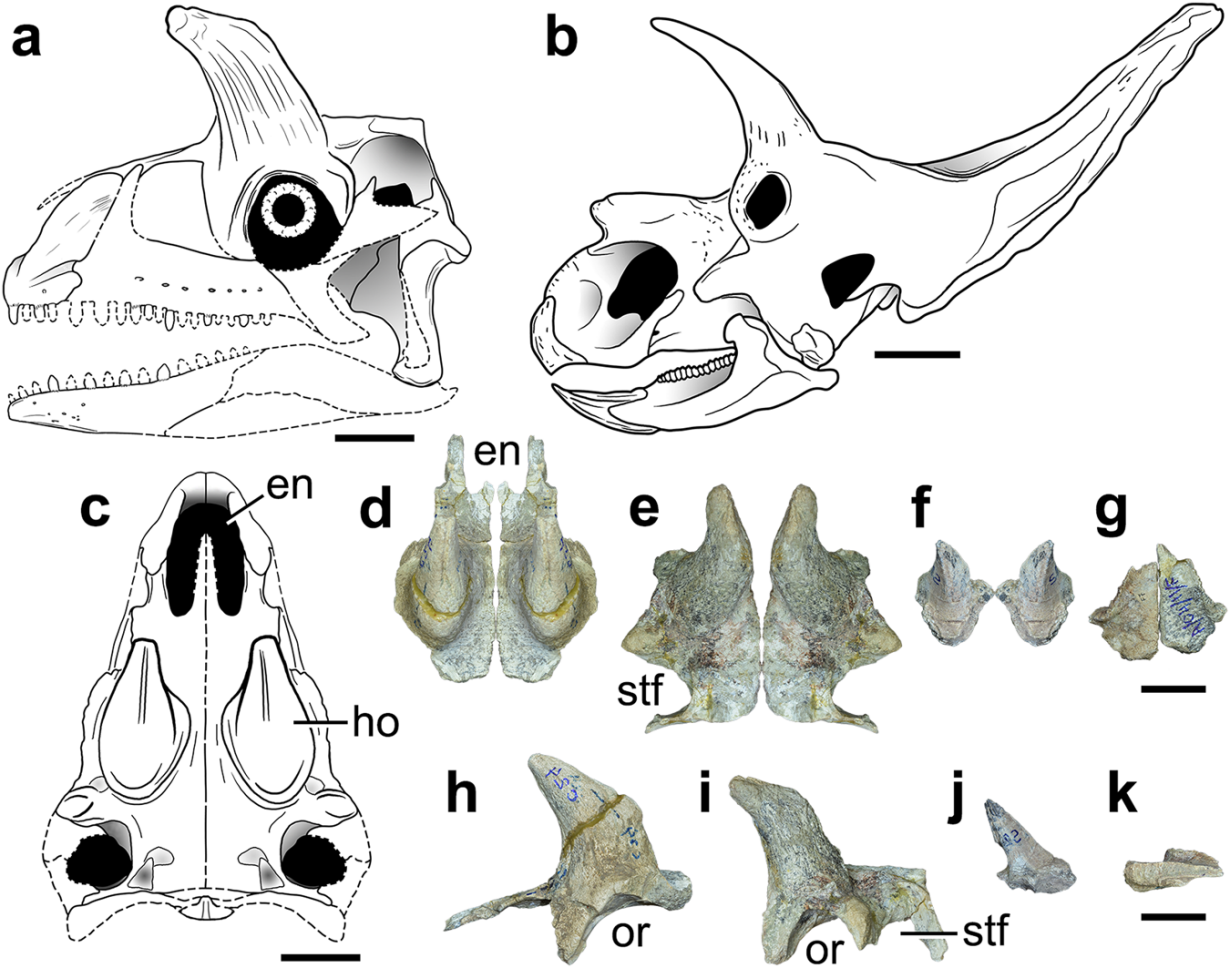
Cranial anatomy of *Shringasaurus indicus* gen. et sp. nov. and comparison with the skull of a ceratopsid dinosaur that possesses convergent﻿ supraorbital horns. **(a**) Reconstruction of the skull of *Shringasaurus indicus* in left lateral view. (**b**) Drawing of the skull of *Arrhinoceratops brachyops* in left lateral view (based on ROM 796^48^). (**c**) Reconstruction of the skull of *Shringasaurus indicus* in dorsal view. (**d–g**) Partial skull tables of *Shringasaurus indicus* in dorsal views (ISIR 781, 780, 786, 789, 790 from left to right), one side has been digitally mirrored in (**d–f). (h–k**) Partial skull tables of *Shringasaurus indicus* in left lateral views (ISIR 781, 780, 786, 790 from left to right). Specimens (**d–f)** and (**h–j)** possesses horns and specimen/s **(g)** and (**k)** lacks horns. Scales = 4 cm for **(a)** and **(** **c**–**k)**, and 20 cm for (**b)**. en, external naris; ho, horn; or, orbit; stf, supratemporal fenestra.

ISIR (Indian Statistical Institute, Reptile, India) 780: partial skull roof (prefrontal, frontal, postfrontal, and parietal) with a pair of large supraorbital horns (Fig. 2e,i).

**Figure 3:**
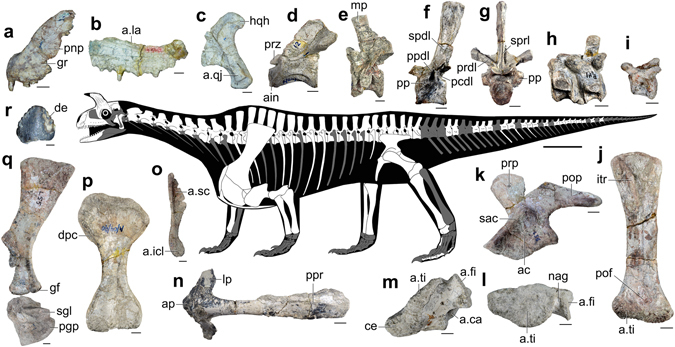
Skeletal anatomy of *Shringasaurus indicus* gen. et sp. nov. **(a**) Left premaxilla (ISIR 793) in lateral view. (**b**) Left maxilla (ISIR 795) in lateral view. (**c**) Left quadrate (ISIR 797) in lateral view. (**d**) Axis (ISIR 803) in left lateral view. (**e**) Posterior cervical vertebra (ISIR 820) in left lateral view. (**f,g**) Anterior dorsal vertebra (ISIR 825) in left lateral view in (**f)**, and anterior view in (**g**). (**h**) Two anterior caudal vertebrae (ISIR 875) in right lateral view (mirrored). (**i**) Posterior caudal vertebra (ISIR 892) in left lateral view. (**j**) Right femur (ISIR 1016) in ventral view. (**k**) Left ilium (ISIR 991) in lateral view. (**l)**, (**m**) Right astragalus and fused lateral centrale (ISIR 1059) in proximal view in (**l**), and dorsal view in (**m**). (**n**) Interclavicle (ISIR 950) in ventral view. (**o**) Left clavicle (ISIR 948) in medial view. (**p**) Left humerus (ISIR 951) in ventral view. (**q**) Left scapula (ISIR 929) and coracoid (ISIR 941) in lateral view. (**r**) Tooth crown (ISIR 801A) in labial view. Scales = 1 cm for (**a–c**,**i**,**m﻿,l)**, 2 cm for (**d–h**,**j**,**k,n–q)**, and 1 mm for **(r)**, and skeleton = 25 cm. a. articulates with; ac, acetabulum; ain, axial intercentrum; ap, anterior process; ca, calcaneum; ce, lateral centrale; de, denticles; dpc, deltopectoral crest; fi, fibula; gf, glenoid fossa; gr, groove; hqh, hooked quadrate head; icl, interclavicle; itr, internal trochanter; la, lacrimal; lp, lateral process; mp, mammillary process; nag, non-articular gap; pcdl, posterior centrodiapophyseal lamina; pgp, postglenoid process; pnp, postnasal process; pof, popliteal fossa; pop, postacetabular process; pp, parapophysis; ppr, posterior process; ppdl, paradiapophyseal lamina; prdl, prezygodiapophyseal lamina; prp, preacetabular process; prz, prezygapophysis; qj, quadratojugal; sac, supraacetabular crest; sc, scapula; sgl, subglenoid lip; spdl, spinodiapophyseal lamina; sprdl, spinoprezygapophyseal lamina; ti, tibia.

#### Paratypes

ISIR 781–1072. Cranial and postcranial bones of at least seven individuals of different ontogenetic stages collected from a single, monospecific ﻿﻿5 metres × 5 metres﻿ ﻿bone-bed (Figs 2 and 3, S1–S3; Supplementary Tables S1, S2).

#### Locality and horizon

Near Tekapar village, Hoshangabad district, Madhya Pradesh, India (Fig. 1); Denwa Formation, Anisian, early Middle Triassic^18^, Satpura Gondwana Basin.

#### Diagnosis

Relatively large (3–4 m total body length; Fig. S1) allokotosaurian archosauromorph that differs from other stem-archosaurs in the following combination of character-states: confluent external nares; pair of anterodorsally oriented supraorbital horns; similar sized and leaf-shaped marginal and palatal teeth with large denticles; middle-posterior cervical, dorsal, and at least the first two caudal vertebrae with mammillary processes on the neural spines; middle-posterior cervical, dorsal, and sacral vertebrae with hyposphene-hypantrum accessory articulations; cervical vertebrae 2–5 with epipophyses (unknown in Cv6); dorsal vertebrae with spinoprezygapophyseal and spinopostzygapophyseal laminae; dorsal vertebrae 1–12 with spinodiapophyseal laminae; anterior dorsal vertebrae with neural spines two times taller than its respective centrum (see Supplementary Information for differential diagnosis).

#### Description

*Shringasaurus indicus* has a proportionally small skull with a short, rounded snout and confluent external nares (Figs 2 and 3). The premaxilla lacks a prenarial process and the postnarial process is plate-like and possesses an anteroventrally oriented lateral groove at its base, as occurs in *Azendohsaurus madagaskarensis* ^20^. The premaxilla has four tooth positions. The nasal has a long anterior process that arcs ventrally. The marginal tooth crowns are low, with a slightly bulbous base and large denticles on both margins, resembling those of *Pamelaria dolichotrachela* (ISIR 316/1). The prefrontal and postfrontal are thick and almost exclude the frontal from the border of the orbit. The prefrontal, nasal, frontal, and postfrontal of each side of the skull are fused to each other in large individuals (i.e. bones remain unfused to their counterpart on the sagittal line). The skull roof possesses an anteriorly curved, conical bony horn, almost equal in height to the rest of the skull in large individuals (Fig. 2). Specimens without co-ossified skull roof bones show that the base of the horn occupies the frontal and most of the transverse width of the pachyostotic skull roof. The surface of the horn is ornamented by tangential rugosities and grooves, which are features that have been identified as osteological correlates of cornified sheaths^21^. The parietal has a very narrow supratemporal fossa that is separated from its counterpart by a flat, broad surface, lacking a sagittal ridge. The quadrate has a hook-shaped dorsal end, as occurs in other allokotosaurians^20^. Vomerine crowns are more lanceolate than those of the marginal dentition. The parabasisphenoid has an oblique, anteroventrally slanting, main axis (Fig. S2).

The anterior-middle cervical centra of *Shringasaurus indicus* are approximately 1.5 times longer than tall, indicating a relatively long neck (Figs 3, S1), but proportionally shorter than in *Azendohsaurus madagaskarensis* ^20^ and *Pamelaria dolichotrachela* ^22^. Besides, the cervical neural spines are proportionally taller than in the latter two species. The first to twelfth dorsal vertebra possess well developed paradiapophyseal, posterior centrodiapophyseal, prezygodiapophyseal, spinodiapophyseal, and spinoprezygapophyseal laminae that bound deep fossae, similar to those of basal sauropods^23^. Epipophyses are present in the anterior cervical vertebrae and are absent in the seventh to ninth cervical vertebra. Mammillary processes (a pair of transverse expansions on the distal portion of the neural spine that is not confluent with the apex of the spine, see ref. 2) are low, laterally projecting, and displaced anteriorly to the anteroposterior mid-depth of the distal end of the neural spine in, at least, the fifth to the ninth cervical, all recovered dorsal vertebrae, and the first two caudal vertebrae (Fig. S3a). The first sacral vertebra is slightly longer than the second and both possess similar-sized ribs. An intercentrum is preserved between two anterior caudal vertebrae.

The clavicle is constricted close to its ventral end and the interclavicle is T-shaped with a short anterior process and a long, paddle-shaped posterior process, similar to *Azendohsaurus madagaskarensis* ^20^. The scapular blade has a distinctly concave anterior margin, as in *Azendohsaurus madagaskarensis* ^20^, but unlike the sub-rectangular blade of *Pamelaria dolichotrachela* ^22^. The scapular blade is moderately expanded anteroposteriorly at its distal end. The coracoid forms part of a posterolaterally oriented glenoid fossa and has a short post-glenoid process. The humerus is strongly constricted at mid-length and the deltopectoral crest occupies half of the length of the bone. The ulna has a low olecranon process.

The ilium possesses a well-developed, semi-circular preacetabular process, and a longer and dorsoventrally shallower postacetabular process. The acetabulum is fully closed and anterodorsally bounded by a low and thick supraacetabular crest. The pubis has a transversely broad apron that contacts its counterpart and, proximally, an extensive plate-like contact with the ischium. The femur is sigmoid with a prominent internal trochanter that does not converge with the femoral head, as in *Azendohsaurus madagaskarensis* ^20^ and *Trilophosaurus buettneri* ^24^. The distal end of the femur is transversely broader than the proximal end and the fibular condyle is slightly more distally extended than the tibial condyle. The fibular shaft is approximately two times narrower than the tibial shaft. The astragalus has tibial and fibular facets separated by a broad non-articular surface and a laterally facing concavity to receive the calcaneum, resembling other early archosauromorphs^1, 2^. The lateral centrale is fused to the astragalus and has a broad articular facet for the reception of the tibia.

#### Phylogeny

**Figure 4:**
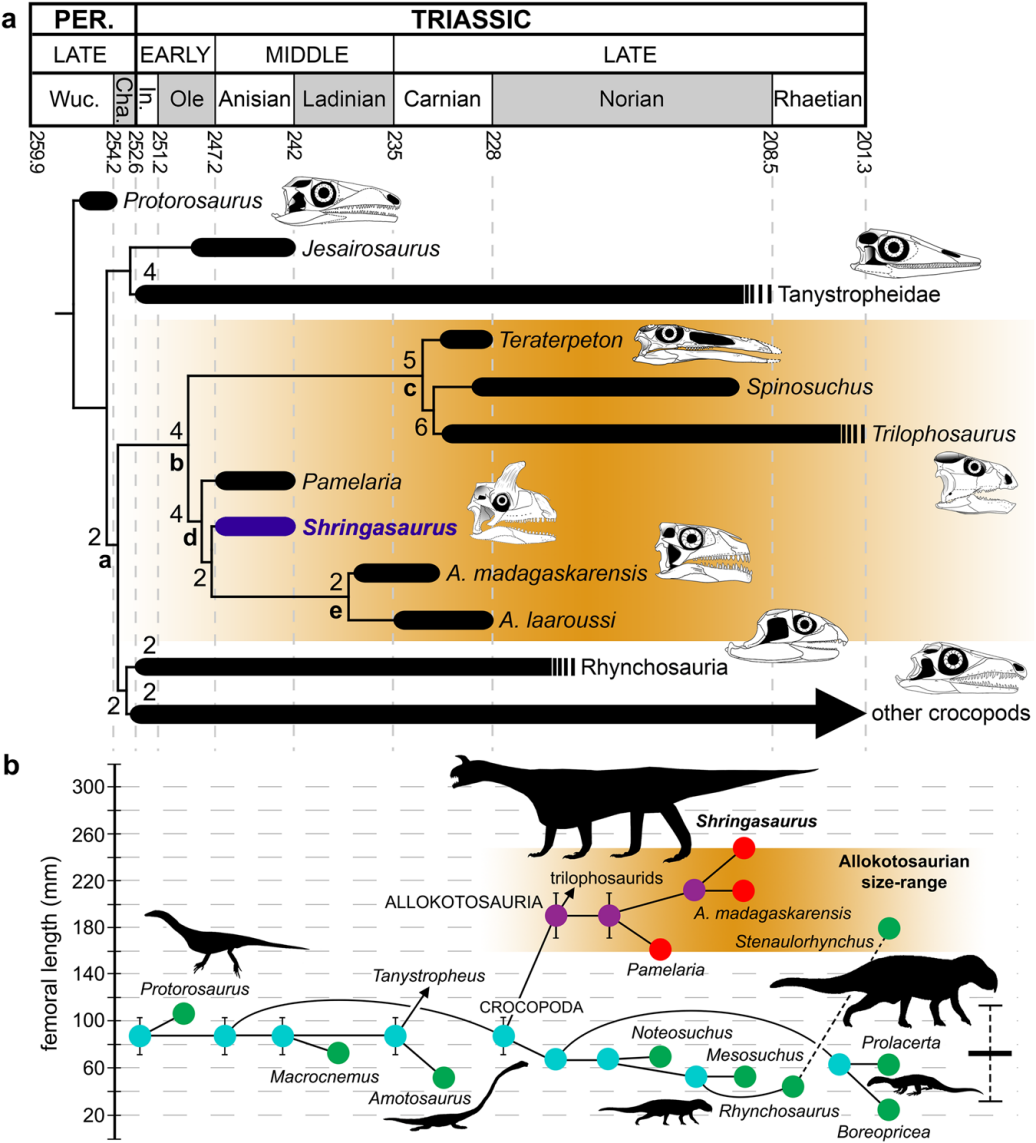
Phylogenetic relationships of *Shringasaurus indicus* gen. et sp. nov. and evolution of body size among early archosauromorphs. (**a**) Time calibrated strict consensus tree found in the data set analysed here (Supplementary Information). Diapsids more basal than *Protorosaurus* are not shown and all clades except Allokotosauria have been collapsed for clarity. Numbers at the nodes are Bremer support values higher than 1. (**b**) Evolution of femoral length (as proxy of body size) optimized as a continuous character using maximum parsimony among non-archosauriform archosauromorphs (Supplementary Information). The horizontal axis represents phylogenetic distance. Green circles represent non-allokotosaurian species, red circles represent allokotosaurians, light blue circles represent non-allokotosaurian ancestral femoral lengths, and violet circles represent allokotosaurian ancestral femoral lengths. The dotted line represents a branch not included in the phylogenetic analysis of this study and the horizontal bar with dotted vertical lines on the right side of the graphic represents the median and standard deviation of Permian to Middle Triassic non-allokotosaurian, non-archosauriform archosauromorph femoral length.﻿ a, Crocopoda; b, Allokotosauria; c, Trilophosauridae; d, Azendohsauridae; e, ﻿*Azendohsaurus﻿*.

A comprehensive phylogenetic analysis focused on Permo-Triassic stem-archosaurs found *Shringasaurus indicus* as a non-archosauriform crocopod^2^, within the clade Allokotosauria (Fig. 4a). Among allokotosaurians, the new species was recovered as an azendohsaurid, together with *Pamelaria dolichotrachela* and both species of *Azendohsaurus*. Several cranial, pectoral and pelvic girdle, and limb synapomorphies support the placement of *Shringasaurus indicus* as an allokotosaurian, azendohsaurid, and the sister-taxon of the genus *Azendohsaurus* (Supplementary Information).

The topology of the strict consensus tree generated from the MPTs is identical to that recovered by Ezcurra^2^ and Nesbitt *et al*.^25^, with exception of the relationships within Allokotosauria (Fig. S4). Contrasting with previous analyses (Ezcurra^2^; Nesbitt *et al*.^20, 25﻿^), *Pamelaria dolichotrachela* was found as the most basal member of Azendohsauridae, and not as the sister-taxon to all other allokotosaurians. The relationships among the other allokotosaurians are congruent with the result of Nesbitt *et al*.^20^, including the taxonomic content of the clade Trilophosauridae, in which *Teraterpeton hrynewichorum* is the sister-taxon of *Spinosuchus caseanus* and *Trilophosaurus buettneri*. Within Azendohsauridae, both species of *Azendohsaurus* are sister-taxa to one another.

Suboptimal alternative relationships for *Shringasaurus indicus* within Allokotosauria and outside this clade were explored using heuristic tree searches under monophyly or non-monophyly constraints. Two additional steps are necessary to force a sister-taxon relationship between the two Indian allokotosaurians *Shringasaurus indicus* and *Pamelaria dolichotrachela*, in which case the clade is sister to all other azendohsaurids. Four extra steps are required to place *Shringasaurus indicus* as the sister-taxon to all other azendohsaurids, in which case *Pamelaria dolichotrachela* is found as the sister-taxon to all other allokotosaurians. Alternatively, five extra steps forces *Shringasaurus indicus* as the most basal azendohsaurid if *Pamelaria dolichotrachela* is the sister-taxon of *Azendohsaurus* spp. Fourteen additional steps forces *Shringasaurus indicus* to be the most basal allokotosaurian and under this constraint *Pamelaria dolichotrachela* is the sister-taxon of Azendohsauridae + Trilophosauridae. Ten extra steps are necessary to find *Shringasaurus indicus* as a trilophosaurid, being the sister-taxon to all other members of the clade. Finally, 26 additional steps are required to place *Shringasaurus indicus* as a non-crocopod archosauromorph (as the sister-taxon of Crocopoda), 25 steps to find it as a rhynchosaur (as the sister-taxon to all other rhynchosaurs), and 29 steps to find it as a crocopod more derived than rhynchosaurs and allokotosaurians (as the sister-taxon to *Boreopricea funerea* plus other archosauromorphs).

The Bremer support of Allokotosauria is relatively low (=2), but it is two times higher for Azendohsauridae (=4). It is interesting to note that all the absolute and GC bootstrap frequencies within Allokotosauria are higher than 50% (Fig. S4), thus indicating a rather robust topology for the group. When *Prolacertoides jimusarensis* and *Azendohsaurus laaroussi* are pruned a posteriori, the Bremer values are of 4 for both Allokotosauria and Azendohsauridae (Fig. S5). As a result, the position of *Shringasaurus indicus* as an azendohsaurid allokotosaurian is very well supported in this dataset.

## Discussion

The most striking feature of *Shringasaurus indicus* is its pair of large supraorbital horns (Figs 2 and 3). These horns should have resulted in a more physiologically costly phenotype than a species with a similar body plan but without such elaborate cranial structures (e.g. *Pamelaria dolichotrachela*, *Azendohsaurus madagaskarensis*) because of the required investment in growth, transport, and maintenance^6, 26^. Horned individuals of *Shringasaurus indicus* of different ontogenetic stages show that the size and robustness of the horns were exacerbated towards the adulthood and possess a distinct variability in their orientation and anterior curvature in large individuals (Fig. 2d–f,h–j). In extant amniotes, the exacerbation of horns and other elaborate cranial structures during ontogeny allows a lower physiological cost to young individuals and their variability is involved in honest quality-signalling^27–31^. The above mentioned traits (i.e. costliness, positive allometry) characterize sexually selected –a subset of natural selection in which the resource at stake is mates^8^– features and, as a result, have been considered as key criteria to recognize secondary sexual characters in the fossil record^6, 7, 32–36^. Besides, the presence of substantial variation in the morphology of the horns of *Shringasaurus indicus* (size and shape) and their potential costliness weakens a species recognition hypothesis as a possible explanation. Species recognition signals have a very low or zero physiological cost (e.g. differences in colour of skin, feathers or fur, vocalisations, chemical signals)^32, 37^ and are likely to exhibit minimal variation within a species because high levels of variation would increase the probability of recognition error^34^. Hence, following the conclusion of previous authors for the elaborate cranial structures of fossil archosaurs (e.g. crests of pterosaurs, hadrosaurids, and oviraptorosarians, horns of ceratopsians)^6, 32, 33^, a non-adaptive, neutral selection, or species recognition hypothesis are not supported as evolutionary drivers for the origin of the horns of the new species (Supplementary Information).

Strong, robust, unbranched, and sub-conical supraorbital horns very similar to those of *Shringasaurus indicus* are found among several amniotes and are mainly used as signals of individual quality and directly as weapons in intraspecific agonistic behaviours (e.g. bovid mammals, chamaeleonid lepidosaurs), usually in male-male combats to get access to receptive females^8, 27–29^. The independent evolution of similar horn shapes and robustness among different groups can be explained as the result of sexual selection acting on the biomechanical performance of weapons^29^. These lines of evidences, including the similarity with the horns of bovids and chamaeleonids, had been used to infer the origin and function of the horns of extinct dinocephalian synapsids and ceratopsian dinosaurs (Fig. 2b) as sexually selected weapons and maybe also used for status signalling^3, 6, 38^, and the same can be interpreted for *Shringasaurus indicus*.

A pair of frontals found in the bone-bed of *Shringasaurus indicus* completely lacks horns, but otherwise is identical to those of horned individuals (Fig. 2g,k). These frontals are approximately of the same size as one specimen with well developed, but still gracile horns (Fig. 2f,j), thus indicating a probable sexual dimorphism. A dimorphic presence/absence of horns is not documented among extinct and extant archosaurs^5, 6^, but occurs in several horned mammals, in which horns are not effective against predators and function only in intraspecific fighting^29^. Thus, the dimorphism is favoured by sexual selection, where females usually lack weapons^8, 27, 28^. In the *Shringasaurus indicus* bone-bed there are at least six horned individuals and only one or two lack horns. Females may be interpreted as those lacking weapons if we consider extant analogues^8, 27^, but this apparent sex ratio can be a result of taphonomic biases because horned skull roofs with fused circumorbital bones are more massive, probably favouring their differential transport and preservation.

*Shringasaurus indicus* attained a relatively large size (3–4 m of total length) that distinctly exceeds the size range of other Early-Middle Triassic archosauromorphs (Fig. 4b). Though there are other probable causes for increases in body size, this could be potentially related with sexual selection because intrasexual competition tends to favour the evolution of larger body sizes^27, 28^. Besides, the new species shows convergences with sauropodomorph dinosaurs, including the shape of marginal teeth, which seems to be related with an herbivorous habit, as previously suggested for *Azendohsaurus* spp.^39^. Thus, it is interpreted that *Shringasaurus indicus* occupied an ecological role as a large primary consumer in its ecosystem, a role previously thought to be restricted to synapsids in Palaeozoic and Early–Middle Triassic terrestrial communities (e.g. edaphosaurids, dinocephalians, anomodonts)^40^, but subsequently successfully exploited by Late Triassic archosauromorphs, such as rhynchosaurs, aetosaurs, and sauropodomorphs^41, 42^. The large size and unusual anatomy of *Shringasaurus indicus* broadens the morphological diversity of Early–Middle Triassic tetrapods and complements the understanding of the evolutionary mechanisms involved in their diversification after the Permo-Triassic mass extinction.

## Methods

### Phylogenetic analysis

The relationships of *Shringasaurus indicus* were analyzed in the most comprehensive phylogenetic dataset available for Permo-Triassic archosauromorphs^2^ as modified by Nesbitt *et al*.^25^. The matrix was analyzed under equally weighted parsimony using TNT 1.5^43, 44^. A heuristic search with 100 replicates of Wagner trees (with a random addition sequence) followed by TBR branch-swapping (holding 10 trees per replicate) was performed. The best trees obtained from the replicates were subjected to a final round of TBR branch swapping. Zero length branches in any of the recovered MPTs were collapsed. Decay indices (=Bremer support values) were calculated and a bootstrap resampling analysis, using 1,000 pseudoreplicates, was performed reporting both absolute and GC (i.e. difference between the frequencies of recovery in pseudoreplicates of the original group and the most frequently recovered contradictory group) frequencies.

We added *Shringasaurus indicus* and three other allokotosaurian species (*Azendohsaurus laaroussi*, “*Spinosuchus* combined”, *Teraterpeton hrynewichorum*) to the original dataset. Two additional terminals were included after splitting the scorings of “*Spinosuchus* combined” into *Trilophosaurus jacobsi* and *Spinosuchus caseanus* in order to test the synonym hypothesis of Nesbitt *et al*.^20^ (Supplementary Information). Two characters were modified, some scorings were changed, and 14 characters were added (601–614; Supplementary Information). The modified data matrix (including “*Spinosuchus* combined”) includes 88 terminals and 620 characters. The following characters were ordered: 1, 2, 7, 10, 17, 19–21, 28, 29, 36, 40, 42, 50, 54, 66, 71, 75, 76, 122, 127, 146, 153, 156, 157, 171, 176, 177, 187, 202, 221, 227, 263, 266, 279, 283, 324, 327, 331, 337, 345, 351, 352, 354, 361, 365, 370, 377, 379, 398, 410, 424, 430, 435, 446, 448, 454, 458, 460, 463, 472, 478, 482, 483, 489, 490, 504, 510, 516, 529, 537, 546, 552, 556, 557, 567, 569, 571, 574, 581, 582, 588.

### Femoral length optimization

Femoral length, as proxy of body size, was optimized as a continuous character^45^ using maximum parsimony in TNT 1.5^43^. Measurements are based on personal observations and published references (Supplementary Information). The median and standard deviation of the femoral length of late Permian to Middle Triassic archosauromorphs (excluding allokotosaurians) were calculated using the software environment R^44^.

### Nomenclatural acts

This published work and the nomenclatural acts it contains have been registered in ZooBank, the proposed online registration system for the International Code of Zoological Nomenclature. The ZooBank LSIDs (Life Science Identifiers) can be resolved and the associated information viewed through any standard web browser by appending the Life Science Identifier to the prefix ‘http://zoobank.org/’. The LSIDs for this publication are urn:lsid:zoobank.org:act:DD9F3C0A-1107-4033-8A6E-8B94A4BD9718 and urn:lsid:zoobank.org:act:2CB56E16-EC8B-4691-99BD-A8F3DF4B26E8﻿.

## Electronic supplementary material

supplemenrary information

data matrix NEXUS

data matrix TNT
