# Supplementary material for: A new horned and long-necked herbivorous stem-archosaur from the Middle Triassic of India
Source: Sci Rep. 2017 Aug 21;7:8366. doi: 10.1038/s41598-017-08658-8 (PMC5567049; doi:10.1038/s41598-017-08658-8)
Supplement: Supplementary file 1 — supplemenrary information [file 41598_2017_8658_MOESM1_ESM.doc]

**Supplementary Information for:**

**A new horned and long-necked herbivorous stem-archosaur from the Middle Triassic of India**

Saradee Sengupta^1,2^*, Martín D. Ezcurra^3^, and Saswati Bandyopadhyay^1^

^1^Geological Studies Unit, Indian Statistical Institute, 203, B. T. Road, Kolkata 700108, India.

^2^Durgapur Govt. College, J. L. N. Road, Durgapur-713214, India.

^3^Sección Paleontología de Vertebrados, CONICET−Museo Argentino de Ciencias Naturales "Bernardino Rivadavia", Avenida Ángel Gallardo 470, Buenos Aires, C1405DJR, Argentina.

*Correspondence to: Saradee Sengupta, Durgapur Govt. College, J. L. N. Road, Durgapur-713214, India. Telephone: +919477609705 E-mail: saradeesengupta@gmail.com.

**Extended Systematic Palaeontology**

Diapsida Osborn, 1903^1^

Archosauromorpha Huene, 1946^2^

Allokotosauria Nesbitt et al., 2015^3^

Azendohsauridae Nesbitt et al., 2015^3^

*Shringasaurus indicus* gen. et sp. nov.

**Differential Diagnosis**—*Shringasaurus indicus* can be distinguished from other archosauromorphs, including other allokotosaurians, by the following autapomorphies: a pair of anterodorsally oriented supraorbital horns; middle-posterior cervicals, dorsals, and at least first two caudals with mammillary processes (a pair of transverse expansions of the distal portion of the neural spine that is not confluent with apex of the spine; Fig. S3a) on the neural spines; and dorsals 1−12 with spinodiapophyseal laminae. In addition, the new genus and species is differentiated from other known allokotosaurian species by the following unique combinations of character-states.

*Shringasaurus indicus* differs from *Pamelaria dolichotrachela* in the presence of a parietal with posterolateral process ventrally inclined in an angle greater than 45º; anteriorly tapering dentary with a Meckelian groove restricted to the ventral border on the anterior half of the bone; cervical and dorsal vertebrae with taller neural spines; dorsal vertebrae with spinoprezygapophyseal laminae; scapula lacking strong curvature or inflexion between the proximal end and the posterior margin of the blade; pubis with apron, in which the symphysis with its counterpart is restricted anteriorly and obscured by the pubic shaft in lateral view; and femoral attachment of the caudofemoralis musculature not convergent with proximal end.

The new genus and species is distinguished from both species of the genus *Azendohsaurus* because multiple maxillary and dentary tooth crowns lack a distinct mesiodistal expansion above the root; the maxilla lacks a prominent anteroposteriorly oriented ridge on the medial surface dorsal to the tooth row; and the dentary tapers more conspicuously anteriorly in lateral view. In addition, *Shringasaurus indicus* differs from *Azendohsaurus madagaskarensis* in the presence of cervical and dorsal vertebrae with taller neural spines; proportionally shorter post-axial anterior and middle cervical vertebrae; and lower olecranon process of the ulna.

*Shringasaurus indicus* differs from *Teraterpeton hrynewichorum* in the presence of considerably shorter and non-edentulous premaxilla, without a prenarial process; a infratemporal fenestra; labiolingually compressed marginal teeth; parietal with posterolateral process ventrally inclined in an angle greater than 45º; proportionally longer anterior and middle cervical vertebrae; and posterior dorsal vertebrae with zygapophyses closer to the median line.

*Shringasaurus indicus* can be differentiated from *Trilophosaurus buettneri* in the presence of a non-edentulous premaxilla, without a prenarial process; an infratemporal fenestra; flat parietals between supratemporal fossae (lacking a sagittal ridge); labiolingually compressed marginal teeth with only one main cusp; parietal with posterolateral process ventrally inclined in an angle greater than 45º; considerably less developed post-glenoid process of the coracoid; scapula lacking strong curvature or inflexion between the proximal end and the posterior margin of the blade; more robust humerus; and lower olecranon process of the ulna.

The new genus and species differs from *Spinosuchus caseanus* (= *Trilophosaurus jacobsi*) in the presence of labiolingually compressed marginal teeth with only one main cusp; an infratemporal fenestra; and proportionally shorter neural spines without an anteroposterior distal expansion.

*Shringasaurus indicus* differs from *Malerisaurus robinsonae* from the Late Triassic of India^4^ and *Malerisaurus* *langstoni* from the Late Triassic of North America^5^ in the presence of leaf-shaped tooth crowns with large denticles (unknown in *M. langstoni*), anterior and middle dorsal vertebrae with distinctly taller neural spines that possess mammillary processes, interclavicle with a well-developed anterior process and T-shaped anterior portion (unknown in *M. robinsonae*), humerus and femur with distinctly more robust shafts, and ilium with a posteriorly oriented postacetabular process.

*Shringasaurus indicus* can be distinguished from putative records of allokotosaurians from the Olenekian of Russia (*Coelodontognathus donensis*, *Coelodontognathus ricovi*, *Doniceps lipovensis*)^6^ and the Late Triassic of the United Kingdom (*Tricuspisaurus thomasi*, *Variodens inopinatus*)^7^ in the presence of labiolingually compressed marginal teeth with only one main cusp; and from the possible allokotosaurian *Arctosaurus osborni*^8^ in the absence of a deep arcuate groove on the ventrolateral surface of the centrum in middle postaxial cervical vertebrae.

**Supplementary Table S1. Referred specimens of *Shringasaurus indicus*, belonging to cranial and postcranial bones of at least seven individuals of different ontogenetic stages.**

| **Registration Number** | **Brief Description** | |
| --- | --- | --- |
| ISIR 780 | Partial skull roof with horn, consists of frontal, prefrontal, postfrontal, and parietal (holotype) | |
| ISIR 781 | Right nasal, frontal, postfrontal, and partial parietal with horn | |
| ISIR 782 | Skull roof with large horn | |
| ISIR 783 | Skull roof with large horn | |
| ISIR 784 | Skull roof with large-sized horn | |
| ISIR 785 | Skull roof with medium-sized horn | |
| ISIR 786 | Horn (medium-sized) | |
| ISIR 787 | Horn (small-sized) | |
| ISIR 788 | Horn (small-sized) | |
| ISIR 789 | Right frontal without horn | |
| ISIR 790 | Left frontal without horn | |
| ISIR 791 | Postfrontal that articulates to ISIR 781 | |
| ISIR 792 | Right nasal (incomplete) | |
| ISIR 793 | Left premaxilla | |
| ISIR 794 | Right premaxilla | |
| ISIR 794A | Broken premaxillary tooth | |
| ISIR 794B | Broken premaxillary tooth | |
| ISIR 795 | Left maxilla | |
| ISIR 795B | Broken maxillary tooth | |
| ISIR 796 | Left quadrate (small) | |
| ISIR 797 | Left quadrate | |
| ISIR 798 | Braincase | |
| ISIR 799 | Braincase (smaller than ISIR 798) | |
| ISIR 800 | Braincase (similar in size to ISIR 798) | |
| ISIR 801 | Left vomer | |
| ISIR 801A | Broken palatal tooth | |
| ISIR 801B | Broken palatal tooth | |
| ISIR 801C | Broken palatal tooth | |
| ISIR 801D | Broken palatal tooth | |
| ISIR 801E | Broken palatal tooth | |
| ISIR 802 | Dentary | |
| ISIR 803 | Nearly complete axis with broken left prezygapophyses | |
| ISIR 804 | Nearly complete axis with broken prezygapophyses | |
| ISIR 805 | Nearly complete axis, broken anterior part of neural spine | |
| ISIR 806 | Axis with broken neural spine | |
| ISIR 807 | Nearly complete third cervical, broken postzygapophyses and neural spine | |
| ISIR 808 | Nearly complete third cervical with distorted postzygapophyses, broken left prezygapophyses | |
| ISIR 809 | Third cervical vertebra, broken prezygapophyses, neural spine, and left postzygapophysis | |
| ISIR 810 | Partial fourth cervical, part of centrum, neural arch, and neural spine present | |
| ISIR 811 | Nearly complete fourth cervical, broken left prezygapophyses | |
| ISIR 812 | Nearly complete fifth cervical, broken right prezygapophyses | |
| ISIR 813 | Nearly complete fifth cervical, right prezygapophysis, postzygapophyses and distal part of neural spine broken | |
| ISIR 814 | Fifth cervical centrum | |
| ISIR 815 | Sixth cervical with broken prezygapophyses and right postzygapophyses | |
| ISIR 816 | Seventh cervical with broken posteroventral part | |
| ISIR 817 | Nearly complete seventh cervical with broken upper part of neural spine, left prezygapophysis, and left postzygapophysis | |
| ISIR 818 | Nearly complete seventh cervical, with broken upper part of neural spine, prezygapophysis, and postzygapophysis | |
| ISIR 819 | Partial seventh cervical with broken centrum, base of the neural arch, and neural spine | |
| ISIR 820 | Complete eighth cervical | |
| ISIR 821 | Nearly complete ninth cervical with partially broken left prezygapophysis | |
| ISIR 822 | Complete first dorsal, transverse processes project laterally form the main body of the centrum | |
| ISIR 823 | Complete first dorsal, neural spine shorter than ISIR 822 and transverse processes project lateroventrally form the main body of the centrum | |
| ISIR 824 | First dorsal centrum | |
| ISIR 825 | Complete second dorsal | |
| ISIR 826 | Second dorsal centrum | |
| ISIR 827 | Second dorsal centrum | |
| ISIR 828 | Distorted second dorsal, centrum and part of postzygapophyses preserved | |
| ISIR 829 | Distorted and partially broken second dorsal | |
| ISIR 830 | Distorted and broken second dorsal neural spine | |
| ISIR 831 | Nearly complete third dorsal, broken left transverse process and upper part of neural spine | |
| ISIR 832 | Nearly complete third dorsal, broken neural spine, prezygapophyses, and postzygapophyses | |
| ISIR 833 | Third dorsal centrum | |
| ISIR 834 | Third dorsal with distorted right lateral side and broken neural spine, prezygapophyses and postzygapophyses | |
| ISIR 835 | Nearly complete fourth dorsal, broken upper part of neural spine | |
| ISIR 836 | Fifth dorsal with broken neural spine, left prezygapophyses, and transverse process | |
| ISIR 837 | Nearly complete fifth dorsal with broken neural spine, left prezygapophyses, and distal part of transverse process | |
| ISIR 838 | Nearly complete fifth dorsal with broken neural spine and transverse process | |
| ISIR 839 | Fifth dorsal centrum | |
| ISIR 840 | Sixth dorsal with broken prezygapophyses and neural spine | |
| ISIR 841 | Nearly complete seventh dorsal, broken upper part of neural spine | |
| ISIR 842 | Partially complete eighth dorsal, broken upper part of neural spine, prezygapophyses, and left transverse process | |
| ISIR 843 | Eighth dorsal with broken neural spine, right lateral part distorted | |
| ISIR 844 | Nearly complete eighth dorsal, broken upper part of neural spine, right prezygapophysis, postzygapophyses, and right transverse process | |
| ISIR 845 | Nearly complete eight dorsal, broken upper part of neural spine | |
| ISIR 846 | Nearly complete ninth dorsal, broken upper part of neural spine, right prezygapophysis, postzygapophyses, and transverse process | |
| ISIR 847 | Tenth dorsal, broken neural spine, postzygapophyses, and left transverse process | |
| ISIR 848 | Broken tenth dorsal centrum | |
| ISIR 849 | Nearly complete eleventh dorsal, broken upper part of neural spine | |
| ISIR 850 | Eleventh dorsal centrum | |
| ISIR 851 | Eleventh dorsal centrum | |
| ISIR 852 | Complete twelfth dorsal | |
| ISIR 853 | Thirteenth dorsal centrum, with broken proximal part of neural spine | |
| ISIR 854 | Thirteenth dorsal centrum | |
| ISIR 855 | Broken neural arch with sub-rounded prezygapophyses of thirteenth dorsal | |
| ISIR 856 | Nearly complete fourteenth dorsal, broken upper part of neural spine | |
| ISIR 857 | Fourteenth dorsal centrum | |
| ISIR 858 | Fourteenth dorsal centrum | |
| ISIR 859 | Broken dorsal centrum, position undetermined | |
| ISIR 860 | Broken neural arch, position undetermined | |
| ISIR 861 | Broken dorsal centrum, position undetermined | |
| ISIR 862 | Broken dorsal centrum, position undetermined | |
| ISIR 863 | Distorted dorsal centrum, position undetermined | |
| ISIR 864 | Articulated first and second sacral | |
| ISIR 865 | Nearly first sacral, broken upper part of neural spine | |
| ISIR 866 | Distorted first sacral, broken upper part of neural spine | |
| ISIR 867 | First sacral centrum with proximal part of transverse process | |
| ISIR 868 | First sacral centrum with proximal part of transverse process | |
| ISIR 869 | Second sacral centrum | |
| ISIR 870 | Second sacral centrum with proximal part of left transverse process | |
| ISIR 871 | Second sacral, right lateral part distorted and broken upper part of neural spine | |
| ISIR 872 | Second sacral centrum | |
| ISIR 873 | Second sacral centrum | |
| ISIR 874 | Second sacral with broken centrum | |
| ISIR 875 | Two articulated anterior caudals | |
| ISIR 876 | Broken anterior caudal centrum | |
| ISIR 877 | Complete anterior caudal | |
| ISIR 878 | Nearly complete anterior caudal, broken upper part of neural spine | |
| ISIR 879 | Nearly complete anterior caudal, broken neural spine and right prezygapophysis | |
| ISIR 880 | Nearly complete anterior caudal with broken neural spine | |
| ISIR 881 | Broken anterior caudal centrum | |
| ISIR 882 | Broken anterior caudal neural arch | |
| ISIR 883 | Complete middle caudal | |
| ISIR 884 | Broken middle caudal centrum | |
| ISIR 885 | Broken middle caudal neural arch | |
| ISIR 886 | Nearly complete middle caudal with broken neural spine | |
| ISIR 887 | Middle caudal centrum with left prezygapophysis and right transverse process | |
| ISIR 888 | Nearly complete distal caudal, broken prezygapophyses and upper part of neural spine | |
| ISIR 889 | Nearly complete distal caudal, broken upper part of neural spine | |
| ISIR 890 | Broken distal caudal with centrum and neural arch | |
| ISIR 891 | Complete distal caudal centrum | |
| ISIR 892 | Complete distal caudal | |
| ISIR 893 | Complete distal caudal, neural spine slanting posteriorly | |
| ISIR 894 | Broken distal caudal | |
| ISIR 895 | Complete distal caudal | |
| ISIR 896 | Broken distal caudal centrum | |
| ISIR 897 | Nearly complete distal caudal | |
| ISIR 898 | Broken distal caudal centrum and neural arch | |
| ISIR 899 | Broken distal caudal centrum | |
| ISIR 900 | Nearly complete distal caudal with broken neural spine | |
| ISIR 901 | Nearly complete distal caudal with broken neural spine | |
| ISIR 902 | Nearly complete distal caudal with broken neural spine | |
| ISIR 903 | Broken neural arch, position undetermined | |
| ISIR 904 | Broken neural arch, position undetermined | |
| ISIR 905 | Broken neural arch, position undetermined | |
| ISIR 906 | Complete cervical rib | |
| ISIR 907 | Broken dorsal rib | |
| ISIR 908 | Broken dorsal rib | |
| ISIR 909 | Broken dorsal rib | |
| ISIR 910 | Broken dorsal rib | |
| ISIR 911 | Broken dorsal rib | |
| ISIR 912 | Broken dorsal rib | |
| ISIR 913 | Broken dorsal rib | |
| ISIR 914 | Broken dorsal rib | |
| ISIR 915 | Broken dorsal rib | |
| ISIR 916 | Broken dorsal rib | |
| ISIR 917 | Broken dorsal rib | |
| ISIR 918 | Rib head | |
| ISIR 919 | Rib head | |
| ISIR 920 | Rib head | |
| ISIR 921 | Rib head | |
| ISIR 922 | Caudal rib | |
| ISIR 923 | Nearly complete chevron | |
| ISIR 924 | Nearly complete chevron | |
| ISIR 925 | Nearly complete chevron | |
| ISIR 926 | Broken chevron | |
| ISIR 927 | Broken chevron | |
| ISIR 928 | Right scapula; complete | |
| ISIR 929 | Left scapula; complete | |
| ISIR 930 | Proximal end of scapula | |
| ISIR 931 | Proximal end of scapula | |
| ISIR 932 | Left nearly complete scapula; broken proximal end | |
| ISIR 933 | Right scapula; slender, crushed, nearly complete | |
| ISIR 934 | Distal end of scapula | |
| ISIR 935 | Left scapula with broken distal end | |
| ISIR 936 | Distal end of scapula | |
| ISIR 937 | Partial scapula | |
| ISIR 938 | Partial scapula | |
| ISIR 939 | Partial scapula | |
| ISIR 940 | Partial scapula | |
| ISIR 941 | Nearly complete left coracoid; broken anterior part | |
| ISIR 942 | Right coracoid with broken proximal end | |
| ISIR 943 | Right coracoid with broken proximal end | |
| ISIR 944 | Left coracoids with broken anteroventral part | |
| ISIR 945 | Proximal part of left coracoid | |
| ISIR 946 | Right coracoid; proximal part only; broken anteroventral portion | |
| ISIR 947 | Left coracoid | |
| ISIR 948 | Complete left clavicle | |
| ISIR 949 | Complete left clavicle | |
| ISIR 950 | Nearly complete interclavicle | |
| ISIR 951 | Complete left humerus | |
| ISIR 952 | Complete left humerus | |
| ISIR 953 | Complete right humerus | |
| ISIR 954 | Nearly complete right humerus with broken proximal end | |
| ISIR 955 | Nearly complete left humerus with broken proximal end | |
| ISIR 956 | Nearly complete left humerus with broken proximal end | |
| ISIR 957 | Nearly complete left humerus with broken proximal end | |
| ISIR 958 | Nearly complete left humerus with broken proximal end | |
| ISIR 959 | Nearly complete right humerus, broken posterior part of distal end | |
| ISIR 960 | Right humerus with broken proximal end | |
| ISIR 961 | Flattened left humerus with broken proximal end | |
| ISIR 962 | Right humerus shaft and distal end | |
| ISIR 963 | Right humerus with broken proximal end | |
| ISIR 964 | Humerus shaft | |
| ISIR 965 | Humerus shaft | |
| ISIR 966 | Humerus shaft | |
| ISIR 967 | Distal end of humerus | |
| ISIR 968 | Left humerus; part of shaft and distal end | |
| ISIR 969 | Broken distal end of right humerus | |
| ISIR 970 | Right humerus, shaft and proximal end | |
| ISIR 971 | Broken distal end of right humerus | |
| ISIR 972 | Broken distal end of right humerus | |
| ISIR 973 | Broken distal end of right humerus | |
| ISIR 974 | Nearly complete humerus | |
| ISIR 975 | Distal end of left humerus | |
| ISIR 976 | Right radius with broken proximal end | |
| ISIR 977 | Left radius with broken distal end | |
| ISIR 978 | Right radius with broken proximal end | |
| ISIR 979 | Left radius with broken distal end | |
| ISIR 980 | Proximal end of radius | |
| ISIR 981 | Distal end of left radius | |
| ISIR 982 | Proximal end of right radius | |
| ISIR 983 | Left ulna | |
| ISIR 984 | Right ulna; broken proximal part | |
| ISIR 985 | Left ulna; broken shaft | |
| ISIR 986 | Distal end of left ulna | |
| ISIR 987 | Proximal end of left ulna | |
| ISIR 988 | Slightly distorted/flattened, broken left ulna | |
| ISIR 989 | Proximal end of left ulna | |
| ISIR 990 | Ulna end | |
| ISIR 991 | Nearly complete left ilium | |
| ISIR 992 | Nearly complete right ilium with broken dorsal blade | |
| ISIR 993 | Nearly complete right ilium with broken dorsal blade | |
| ISIR 994 | Right ilium acetabular region and part of iliac blade | |
| ISIR 995 | Right ilium with broken dorsal part | |
| ISIR 996 | Right ilium acetabular region | |
| ISIR 997 | Broken iliac blade | |
| ISIR 998 | Broken iliac blade | |
| ISIR 999 | Posterior part of iliac blade | |
| ISIR 1000 | Posterior part of iliac blade | |
| ISIR 1001 | Fragment of right iliac blade | |
| ISIR 1002 | Broken right iliac blade | |
| ISIR 1003 | Broken right iliac blade | |
| ISIR 1004 | Broken iliac blade | |
| ISIR 1005 | Left ischium; anteroventral part missing | |
| ISIR 1006 | Broken ischium | |
| ISIR 1007 | Proximal end of left pubis | |
| ISIR 1008 | Proximal end of left pubis | |
| ISIR 1009 | Proximal end of right pubis | |
| ISIR 1010 | Proximal end of left pubis | |
| ISIR 1011 | Proximal end of left pubis | |
| ISIR 1012 | Proximal end of left pubis | |
| ISIR 1013 | Proximal end of right pubis | |
| ISIR 1014 | Proximal end of right pubis | |
| ISIR 1015 | Broken girdle bone | |
| ISIR 1016 | Complete right femur | |
| ISIR 1017 | Complete left femur | |
| ISIR 1018 | Nearly complete right femur with broken proximal end | |
| ISIR 1019 | Right femur distal end | |
| ISIR 1020 | Right femur; shaft and distal end | |
| ISIR 1021 | Femur; both proximal and distal ends missing | |
| ISIR 1022 | Distal end of right femur | |
| ISIR 1023 | Distal end of left femur | |
| ISIR 1024 | Proximal end of femur | |
| ISIR 1025 | Proximal end of right femur | |
| ISIR 1026 | Proximal end of femur | |
| ISIR 1027 | Proximal end of right femur | |
| ISIR 1028 | Right femur; posterior part of distal end | |
| ISIR 1029 | Right femur with broken proximal end | |
| ISIR 1030 | Proximal end of femur | |
| ISIR 1031 | Distorted femoral head | |
| ISIR 1032 | Left tibia with broken proximal end | |
| ISIR 1033 | Complete left tibia | |
| ISIR 1034 | Right tibia with broken proximal end | |
| ISIR 1035 | Proximal end of right tibia | |
| ISIR 1036 | Complete left tibia | |
| ISIR 1037 | Complete right fibula | |
| ISIR 1038 | Partial fibula | |
| ISIR 1039 | Limb end | |
| ISIR 1040 | Limb end | |
| ISIR 1041 | Limb end | |
| ISIR 1042 | Limb end | |
| ISIR 1043 | Limb end | |
| ISIR 1044 | Limb end | |
| ISIR 1045 | Limb end | |
| ISIR 1046 | Limb end | |
| ISIR 1047 | Limb shaft | |
| ISIR 1048 | Limb shaft | |
| ISIR 1049 | Limb shaft | |
| ISIR 1050 | Limb shaft | |
| ISIR 1051 | Limb end | |
| ISIR 1052 | Limb end | |
| ISIR 1053 | Partial radius | |
| ISIR 1054 | Limb end | |
| ISIR 1055 | Limb end | |
| ISIR 1056 | Limb end | |
| ISIR 1057 | Limb end | |
| ISIR 1058 | Limb end | |
| ISIR 1059 | Complete right astragalus and fused lateral centrale | |
| ISIR 1060 | Complete left metatarsal | |
| ISIR 1061 | Carpal/metacarpal | |
| ISIR 1062 | Carpal/tarsal | |
| ISIR 1063 | Phalanx | |
| ISIR 1064 | Phalanx | |
| ISIR 1065 | Phalanx | |
| ISIR 1066 | Phalanx | |
| ISIR 1067 | Phalanx | |
| ISIR 1068 | Phalanx | |
| ISIR 1069 | Phalanx | |
| ISIR 1070 | Carpal/tarsal | |
| ISIR 1071 | Phalanx | |
| ISIR 1072 | Phalanx | |
|  | |  |
|  | |  |

**Additional photographs of the hypodigm of *Shringasaurus indicus***


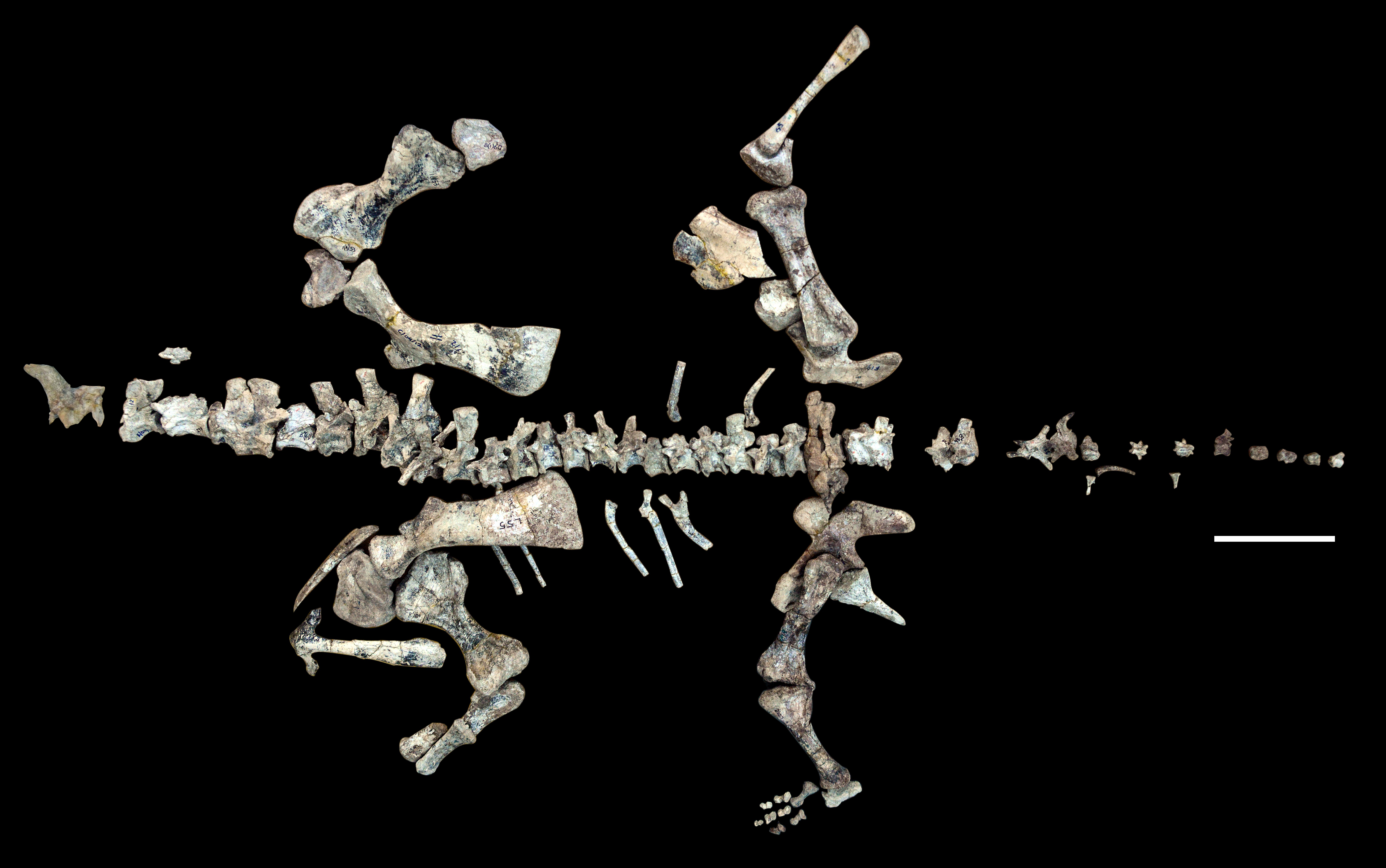


**Figure S1.** **Composite skeleton of *Shringasaurus indicus*.** The bones probably belong to multiple, similar-sized individuals. Scale = 50 cm.


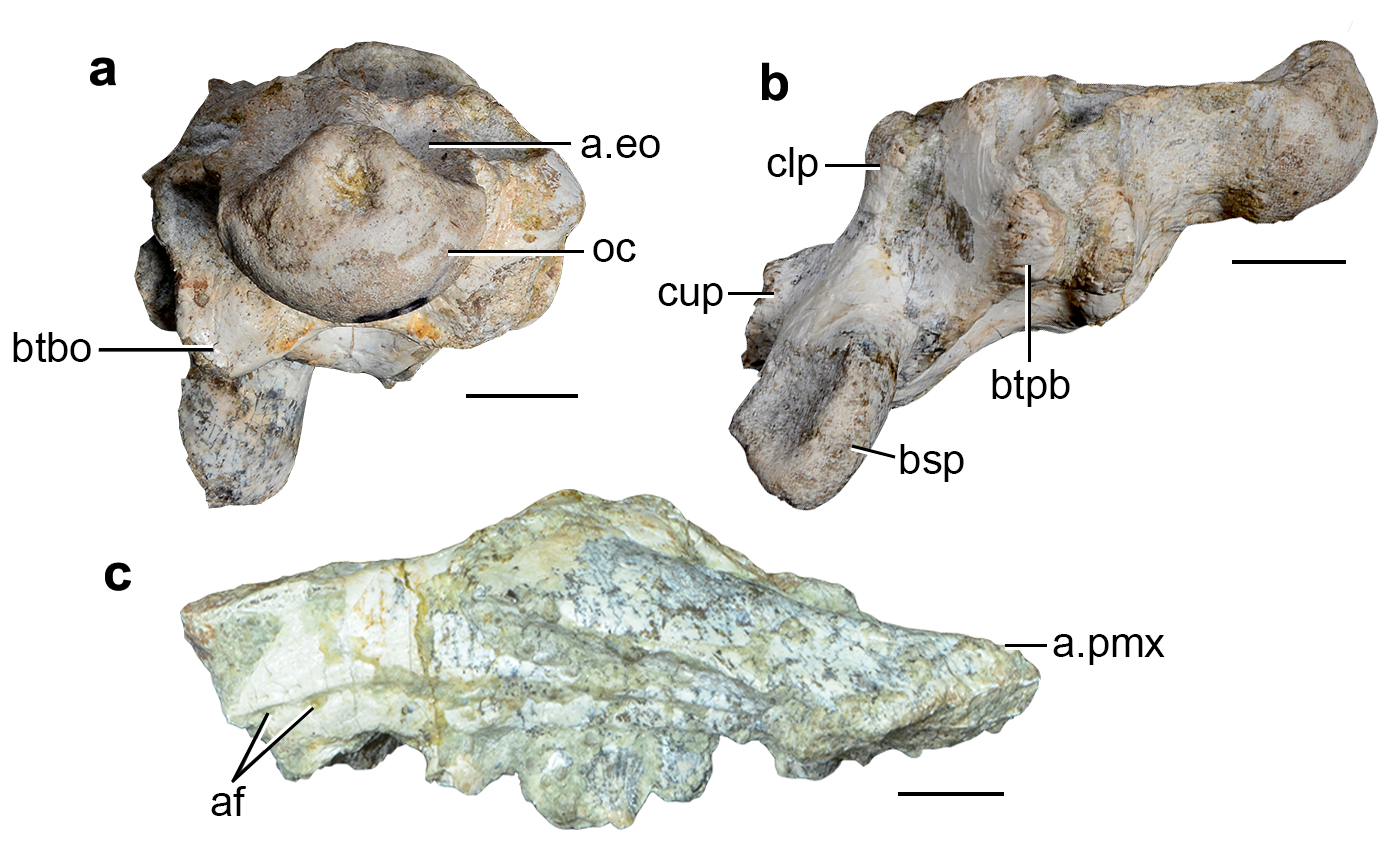


**Figure S2.** Palatal and braincase bones of *Shringasaurus indicus*. **a**, Basioccipital articulated with parabasisphenoid (missing the cultriform process) (ISIR 798) in posterior view. **b**, Basioccipital articulated with parabasisphenoid (missing the cultriform process) (ISIR 798) in left lateral view. **c**, Left vomer in (ISIR 801) in medial view. Scales = 1 cm. a.eo, articular facet for exoccipital; a.pmx, articular facet for premaxilla; af, alveolar foramina; bsp, basipterygoid process; btbo, basal tubera of the basioccipital; btpb, basal tubera of the parabasisphenoid; clp, clinoid process; cup, cultriform process.


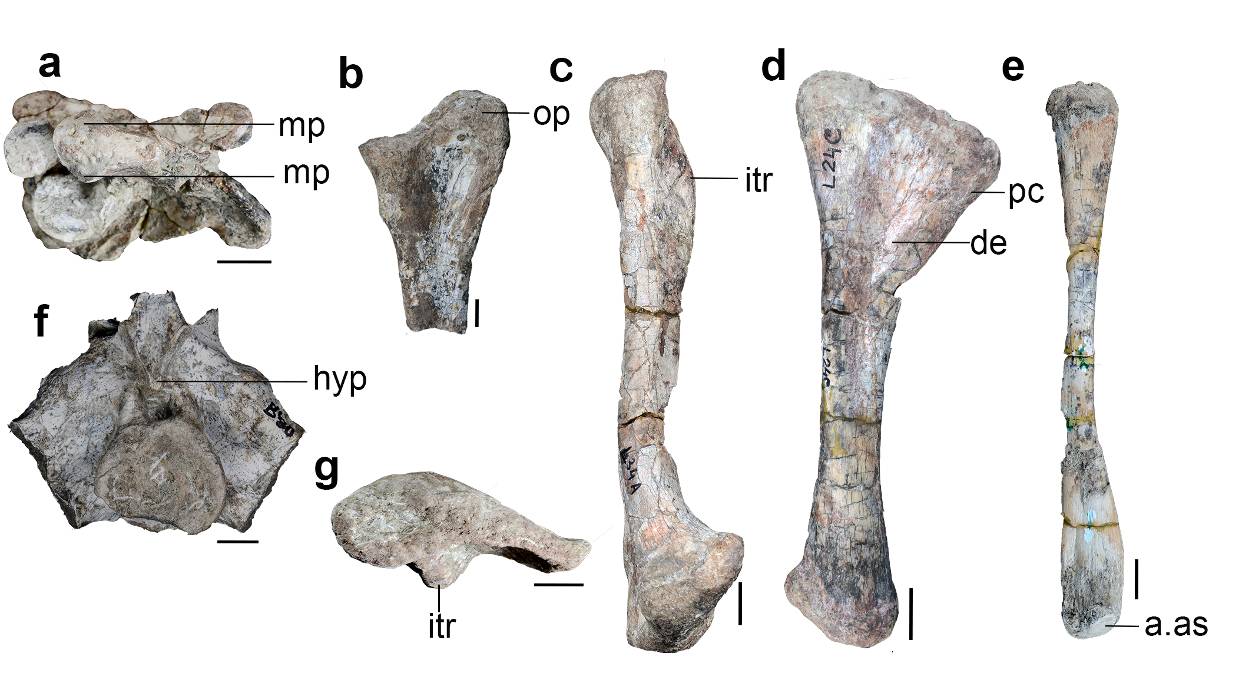


**Figure S3.** Postcranial bones of *Shringasaurus indicus*. **a**, Posterior cervical vertebra (ISIR 820) in dorsal view. **b**, Proximal half of right ulna (ISIR 984) in medial view. **c**, Right femur (ISIR 1016) in medial view. **d**, Left tibia (ISIR 1033) in lateral view **e,** Left fibula (ISIR 1037) in medial view. **f,** First sacral vertebra (ISIR 865) in posterior view. **g,** Right femur (ISIR 1016) in proximal view. Scales = 2 cm. a.as, articular facet for the astragalus; de, depression; hyp, hyposphene; itr, internal trochanter; mp, mammillary processes; op, olecranon process; pc, posterior hemicondyle.

**Supplementary Table S2. Selected measurements of postcranial bones of *Shringasaurus indicus*. The bones may belong to different individuals. All the measurements are in millimetres. The asterisk (*) indicates an incomplete measurement.**

**Fourth or fifth cervical vertebra**

Centrum length 53.5

Centrum anterior height 36.3

**Anterior dorsal vertebra**

Centrum length 54.1

Centrum anterior height 44.0

Length of transverse process 53.0

Neural spine height 118.1

Length across zygapophyses 75.5

**Posterior dorsal vertebra A**

Centrum length 41.7

Centrum anterior height 51.6

Length across zygapophyses 57.5

**Posterior dorsal vertebra B**

Centrum length 39.8

Centrum anterior height 44.3

Neural spine height 65.2

Length across zygapophyses 63.8

**Second caudal vertebra**

Centrum length 34.0

Centrum anterior height 46.0

Neural spine height 37.0

**Scapula**

Blade length 382.8

Blade minimum width 75.0

**Interclavicle**

Posterior stem length 237.2*

Posterior stem width at mid-length 39.9

**Humerus**

Length 272.8

Proximal width 153.7

Length of deltopectoral crest 140.3

Minimum width of shaft 49.3

Distal width 113.6

**Femur**

Length 267.6

Proximal width 82.6

Minimum width of shaft 48.2

Distal width 99.0

**Further details on the phylogenetic analysis**

**1. New and revised characters added to the dataset of Ezcurra (2016)^9^**

601. Maxilla, posterolateral surface: directly adjacent to alveolar margin (0); lateral process of maxilla present, creating distinct space between maxillary alveoli and posterolateral surface of the maxilla (1) (Pritchard et al., 2015: 8^10^).

602. Maxilla, medial surface dorsal to tooth row: smooth (0); prominent anteroposteriorly oriented ridge present (1) (Nesbitt et al., 2015: 201^3^).

603. Teeth, crown height of the upper dentition compared with lower dentition: similar tooth crown height (0); the upper dentition is shorter relative to the taller lower dentition (1) (Nesbitt et al., 2015: 211^3^).

604. Teeth, morphology of crown base: single, pointed crown (0); flattened platform with pointed cusps (1); mesiodistally arranged cusps (2) (Pritchard et al., 2015: 93^10^).

605. Cervical and dorsal vertebrae, shape of posterior articular surface: planar (0); concave (1); convex (2) (Pritchard et al., 2015: 102^10^).

606. Dorsal vertebrae, diapophysis, position: anterior portion of the neural arch/centrum (0); anteroposterior middle of the neural arch/centrum (1) (Nesbitt et al., 2015: 215^3^).

607. Dorsal vertebrae, spinoprezygapophyseal lamina: absent (0); present (1).

608. Dorsal vertebrae, spinopostzygapophyseal lamina: absent (0); present (1) (cf. Nesbitt et al., 2015: 245^3^).

609. Dorsal vertebrae, height of neural spine in anterior dorsals: lower than two times the height of its respective centrum (0); equal or higher than two times the height of its respective centrum (1).

610. Caudal vertebrae, length of the anterior caudal vertebrae (caudal vertebrae 1–10) relative to posterior caudal vertebrae (25+): nearly the same length (0); posterior caudal vertebrae much longer (1) (Nesbitt et al., 2015: 218^3^).

611. Clavicle, portion articulated with the interclavicle, shape: broader than distal portion of clavicle (0); similar in narrowness to the distal portion of the clavicle (1) (Pritchard et al., 2015: 141^10^).

612. Humerus, entepicondyle: exhibits a curved proximal margin (0); exhibits a prominently angled proximal margin (1) (Pritchard et al., 2015: 155^10^).

613. Ilium, postacetabular process in lateral view: squared or rounded (0); subtriangular, tapering posteriorly (1).

614. Pedal digits, penultimate phalanges (last phalanx before ungual): shorter or sub-equal than the more proximal phalanges (0); distinctly longer than the more proximal phalanges (1) (Nesbitt et al., 2015: 235^3^).

Characters 615−620 are those added by Nesbitt et al.^11^.

**2. Characters modified from the dataset of Ezcurra (2016)^9^**

260. Lower jaw, symphysis: formed only by dentary (0); formed only by splenial (1); formed by dentary and also conspicuous participation of splenial (2) (Dilkes, 1998^12^; cf. Pritchard et al., 2015: 78, 85^10^).

305. Teeth, tooth shape at crown base of the marginal dentition: circular (0); labiolingually compressed (1); labiolingually wider than mesiodistally long (2) (Dilkes, 1998^12^; Reisz & Dilkes, 2003:1, 34^13^; Reisz et al., 2010: 4^14^; Ezcurra et al., 2010: 35^15^; Ezcurra et al., 2014: 4^16^; Pritchard et al., 2015: 98^10^).

**3. Scoring changes from the dataset of Ezcurra (2016)^9^**

Character 127: *Trilophosaurus buettneri*, changed from (0) to (2).

Character 167: *Azendohsaurus madagaskarensis*, changed from (0) to (1).

Character 260: *Trilophosaurus buettneri*, changed from (0) to (2).

Character 532: *Protorosaurus speneri*, changed from (2) to (0).

**4. Scorings of the new characters for terminals included in the original data set**

Scorings for the characters added here in the terminals already included by Ezcurra^9^ and Nesbitt et al.^11^:

*Petrolacosaurus kansensis* 00001000000000

*Acerosodontosaurus piveteaui* ?00010000??00?

*Youngina capensis* 0?0010000?1000

*Paliguana whitei* 0??0??????????

*Planocephalosaurus robinsonae* 000010000?100?

*Gephyrosaurus bridensis* 00001000??10??

*Cteniogenys* sp. 000010?????0??

*Simoedosaurus lemoinei* 00?011000?100?

*Aenigmastropheus parringtoni* ????1100???0??

*Protorosaurus speneri* 000010?000?0?0

*Amotosaurus rotfeldensis* 00001???0????0

*Macrocnemus bassanii* 000010?0001010

*Tanystropheus longobardicus* 000[0 2]1100001010

*Jesairosaurus lehmani* 0?00?0000?110?

*Pamelaria dolichotrachela* 00?0100000?010

*Azendohsaurus madagaskarensis* 01101010001010

*Trilophosaurus buettneri* 10012100111111

*Noteosuchus colletti* ?????0??0????0

*Mesosuchus browni* 00001000000000

*Howesia browni* 0?00?00?????0?

*Eohyosaurus wolvaardti* ??00??????????

*Rhynchosaurus articeps* 0000?00000?0?0

*Bentonyx sidensis* 00?0??????????

*Eorasaurus olsoni* ?????0????????

*Prolacertoides jimusarensis* 0??0??????????

*Prolacerta broomi* 000010000010?0

*Kadimakara australiensis* holotype ???0??????????

*Kadimakara australiensis* combined ???0??????????

*Boreopricea funerea* 0??010000??0?0

*Archosaurus rossicus* holotype ??????????????

*'Proterosuchus ferugsi'* holotype ??????????????

*Proterosuchus fergusi* 000010000????0

*Proterosuchus goweri* 00?0??????????

*Proterosuchus alexand*eri 0?0010????0???

'*Chasmatosaurus*' *yuani* 0?0010000001?0

*'Chasmatosaurus ultimus*' 0?00??????????

*Ankistrodon indicus* ???0??????????

*Tasmaniosaurus triassicus* ?00010?1??????

*Exilisuchus tubercularis* ??????????????

*Blomosuchus georgii* ??????????????

*Vonhuenia fredericki* ????1?????????

*Chasmatosuchus rossicus* combined ????1011??????

*Chasmatosuchus magnus* ????1?????????

*Gamosaurus lozovskii* ????1?????????

*Chasmatosuchus magnus* combined ????1?????????

*Chasmatosuchus vjushkovi* ???0??????????

SAM P41754 Long Reef ????11000?????

*Koilamasuchus gonzalezdiazi* ??????????????

*Kalisuchus rewanensis* holotype ?0?0??????????

*Fugusuchus hejiapanensis* 0??0??????????

*Sarmatosuchus otschevi* ???010000?????

*Guchengosuchus shiguaiensis* 00?01010???0??

*Cuyosuchus huenei* ????10?????00?

GHG 7433MI ?0?0?0?????0??

*Garjainia prima* 000010000?000?

*Garjainia madiba* holotype ??????????????

*Garjainia madiba* combined ?0?01000???00?

*Erythrosuchus africanus* 0000100000?01?

*Shansisuchus shansisuchus* 00?01000???010

*Shansisuchus kuyeheensis* ???010????????

*Chalishevia cothurnata* 00?01?????????

*Youngosuchus sinensis* 0?00???????0??

*'Dongusia colorata*' ????11????????

*Uralosaurus* holotype ??????????????

*Uralosaurus* combined ???0??????????

*Vancleavea campi* 0?00100????00?

*Asperoris mnyama* ?0?0??????????

*Euparkeria capensis* 00001000001000

*Dorosuchus neoetus* ????????????1?

*Proterochampsa barrionuevoi* 0??01???0????0

*Proterochampsa nodosa* 0??0??????????

*Tropidosuchus romeri* 0?00?0??00?0?0

*Cerritosaurus binsfeldi* 0??0?0????????

*Gualosuchus reigi* 0000?0??0??-??

*Chanaresuchus bonapartei* 000010000??010

*Pseudochampsa ischigualastensis* 0??0????0?1??0

*Rhadinosuchus gracilis* 0??01?????????

*Archeopelta arborensis* ????????0?????

*Tarjadia ruthae* ????1100??????

*Jaxtasuchus salomoni* 00?0?0??0??0??

*Doswellia kaltenbachi* ???010000?1?0?

*Parasuchus angustifrons* 0?????????????

*Parasuchus hislopi* 0??01000001000

*Nicrosaurus kapffi* 0?0010001?100?

*Smilosuchus* sp. 0?0010000?100?

*Ornithosuchus longidens* 0?001000001?10

*Riojasuchus tenuisceps* 0?0010000??010

*Nundasuchus songeaensis* ???01000???0??

*Turfanosuchus dabanensis* 0??0100000?01?

*Gracilisuchus stipanicicorum* 0000100?????10

*Aetosauroides scagliai* 0??01000??1000

*Batrachotomus kupferzellensis* 000010000?100?

*Prestosuchus chiniquensis* 000010000??00?

*Dimorphodon macronyx* 0?[0 1]01???????01

*Lagerpeton chanarensis* ????????????00

*Marasuchus lilloensis* ?0?0?0??01?010

*Lewisuchus admixtus* 0000?0??0?????

*Asilisaurus kongwe* ???01000?0?01?

*Silesaurus opolensis* 0000110000?000

*Heterodontosaurus tucki* 000011??00?000

*Herrerasaurus ischigualastensis* 0?001100?1?000

*Yarasuchus deccanensis* ????11001??00?

*Dongusuchus efremovi* ??????????????

*Teleocrater* combined 00?01100?0?0??

*Spondylosoma absconditum* ????1100??????

**5. Ratios and angles calculated for the terminals added here to score quantitative discretized characters**

Character 20:

*Shringasaurus*: ca. 0.52

*Teraterpeton* (Sues, 2003: fig. 1^17^): >0.64

Character 21:

*Teraterpeton* (Sues, 2003: fig. 1^17^): 0.63

Character 28:

*Shringasaurus*: 1.15

*Teraterpeton* (Sues, 2003: fig. 1^17^): >10.07

Character 75:

*Shringasaurus*: 10−22

*Teraterpeton*^17^: 15

*Trilophosaurus jacobsi*: 10−22

Character 76:

*Teraterpeton* (Sues, 2003: fig. 2^17^): 1.65

Character 100:

*Teraterpeton* (Sues, 2003: fig. 1^17^): 0.95

Character 177:

*Shringasaurus*: 111.4º

*Teraterpeton* (Sues, 2003: fig. 1^17^): 91º

Character 266:

*Azendohsaurus laaroussii* (MNHN-ALM 351): 0.29

*Trilophosaurus jacobsi* (Spielmann et al., 2008: fig. 93a^18^): 0.22

Character 331:

*Shringasaurus*: 1.47

*Spinosuchus* (Spielmann et al., 2009: table 1^19^): 2.32−2.48

*Teraterpeton* (Sues, 2003: fig. 7^17^): 1.31

Character 351:

*Shringasaurus*: 1.23

*Spinosuchus* (Spielmann et al., 2009: table 1^19^): 1.43−1.67

Character 352:

*Shringasaurus*: 0.81−0.90

*Spinosuchus* (Spielmann et al., 2009: table 1^19^): 1.39, 1-69

Character 379:

*Shringasaurus*: 1.57

*Spinosuchus* (Spielmann et al., 2009: fig. 10k^19^): 3.77

Character 387:

*Shringasaurus*: 5.10

Character 416:

*Shringasaurus*: 0.56

*Trilophosaurus jacobsi* (Spielmann et al., 2008: fig. 100^18^): 0.25

Character 424:

*Shringasaurus*: 0.52

*Trilophosaurus jacobsi* (Spielmann et al., 2008: fig. 100^18^): 0.34

Character 448:

*Trilophosaurus jacobsi* (Spielmann et al., 2008: fig. 104^18^): 0.49

Character 463:

*Shringasaurus*: 0.92

*Trilophosaurus jacobsi* (Spielmann et al., 2008: figs. 105, 106^18^): 0.96

Character 510:

*Shringasaurus*: 0.36

Character 556:

*Trilophosaurus jacobsi* (Spielmann et al., 2008: fig. 110^18^): 0.64

**6. Complete scorings of terminals added here**

*Azendohsaurus laaroussi* 0???????10??0-????????0?????0???1??2????0????0???-?10--?1110?--??0-000?000[1 2]????????????????????????????????????????????????????????????????????????????????????????????????????????????????????????????????????????????????????????????????????????????????????????00???0220010??????000??????????????????1010221001????????????????????????????????????????????????????????????????????????????????????????????????????????????????????????????????????????????????????????????????????????????????????????????????????????????????????????????????????????????????????????????????????????????????????????????????????0110????????????????

*Shringasaurus indicus* 0?0?0-0110010-?????1?0???011[0 1]1001--2000?0200?0???-??0--?1????--????[0 2]???000[1 2]?00?0-0-010--???????-??????????0??000100000-00000??????????????????0??????1????????0010-??11?0?????0120?1011?002?1???????????????????????????????????????????????????????????????????????20-??????1????0-??00??????????????????1??0221000?100101111020--???10??011001001000001?1??1100001111010014?0100010-1??00002?1?00?011010??0101010?00001?10?0110001000211101100?0???0????????????????000?010011010[0 1]10??000[1 2]1?????000?0???10000000100001000?0200????0010000010010?02?010000000??????????????????????01??????????????????1??0----------00001010111?0011?00000

*Trilophosaurus jacobsi* 0?000-01????0-?001???10???????????????????????0-?-??0--??0???--????0??[0 1]000[1 2]??????0-0?0--???0001-00-???-??-0?00?0?00??0-???100020??000010?0-?00???-??11?-??????0?00-2-10??????0????0????????????????????????????????????????????????????????????????????????????????220-?022???0??????1000?????0??0????????10?0202000?1?01????????????11????21001?1100?10???????????????????????????????0???00????0???????????20?00-???????????10000010011110110010????00???????2????1?0?0?0100100?0010??01000-1--??????0??00000000100000000?0?00001?00100000???????2000000000001100000000002?????0???1????????0?????????1?10----------??10012??1?1?10?1??00?

*Spinosuchus caseanus* ?????????????????????????????????????????????????????????????????????????????????????????????????????????????????????????????????????????????????????????????????????????????????????????????????????????????????????????????????????????????????????????????????????????????????????????????????????????????????????100101111000--0??1???0210?1?1?0???0??1???1[0 1]000-11?0?001-?010?0?0-????3????????????????????????????????????????????????????????????????????????????????????????????????????????????????????????????????????????????????????????????????????????????????????????????????????????????????0----------??????21011??????0????

*Spinosuchus combined* 0?000-01????0-?001???10???????????????????????0-?-??0--??0???--????0??[0 1]000[1 2]??????0-0?0--???0001-00-???-??-0?00?0?00??0-???100000??000010?0-?00???-??11?-??????0?00-2-10??????0????0????????????????????????????????????????????????????????????????????????????????220-?022???0??????1000?????0??0????????10?0202000?100101111000--0?11???021001?1100?10??1???1[0 1]000-11?0?001-?010?0?0-?0??300????0???????????20?00-???????????10000010011110110010????00???????2????1?0?0?0100100?0010??01000-1--??????0??00000000100000000?0?00001?00100000???????2000000000001100000000002?????0???1????????0?????????1?10----------??1001210111?10?10?00?

*Teraterpeton hrynewichorum* ??000-0001010-00010[1 2]20000-141??000010000?5--??0-?--10---1010?--00??0101000210000-0-0?1--??10000-00-000-02-010010110100-??00000201000?01010-?00001-0011-----12-01-????101??2-10011001?0???0?01???????????????0??0101011?0?????0?????010????0??????001?????????1???0?00?????2?0??1????1100??10??0?10?10????01010200001?1?00??????00--???0???0[1 2]00?1??10?010-11??1???????1?00???-10???????????????[0 1]??0??011?1????2???0-?0?????????????????????????????????????????????10???????????????????????????????????????????????????????????????????????????????????????????????????????????????????????????????????????0----------??0?0110????1?????????

**7. Taxa excluded a priori from the analysis**

The analysis conducted here is based on the third analysis of Ezcurra^9^. As a result, we excluded a priori from the analysis the following terminals: *Eorasaurus olsini*, *Kadimakara australiensis* holotype, *Archosaurus rossicus*, “*Proterosuchus fergusi*” holotype, “*Chasmatosaurus ultimus*”, *Ankistrodon indicus*, *Exilisuchus tubercularis*, *Blomosuchus georgii*, *Vonhuenia fredericki*, *Chasmatosuchus rossicus* combined, *Chasmatosuchus magnus*, *Gamosaurus lozovskii*, *Chasmatosuchus magnus* combined, *Chasmatosuchus vjushkovi*, SAM P41754 Long Reef, *Kalisuchus rewanensis*, *Garjania madiba* holotype, *Shansisuchus kuyeheensis*, “*Dongusia colorata*”, *Uralosaurus magnus* holotype, and *Uralosaurus* *magnus* combined. See Ezcurra^9^ for the logical basis of the exclusion of these terminals.

In addition, we performed two alternative analyses: one considering *Trilophosaurus jacobsi* as a junior synonym of *Spinosuchus caseanus* sensu Nesbitt et al.^3^ (using *Spinosuchus* combined) and the other including these two species as independent terminals. The first analysis resulted in a total of 88 terminals and the second in a total of 89 terminals.

**8. Results**

The analysis of the dataset (using “*Spinosuchus* combined” as active terminal) recovered two most parsimonious trees (MPTs) of 2788 steps, with a consistency index (CI) of 0.2902 and a retention index (RI) of 0.6313. The best score hit 72 of 100 times. The two MPTs only differ from each other in the position of *Prolacertoides*, being alternatively found as the most basal allokotosaurian or as the sister-taxon of *Jesairosaurus* (Figs. S4, S5).

The following ten synapomorphies support the position of *Shringasaurus* as an allokotosaurian (when *Prolacertoides* is considered a non-crocopodan archosauromorph or is excluded from the analysis): maxilla with 10−22 tooth positions (75: 3->1/2), quadrate with hooked dorsal end in lateral view (180: 0->1), palatine, ventral surface of the anterior ramus of the pterygoid, and vomer with height and diametre of teeth similar to those of the marginal dentition (189: 0->1), maxillary tooth crowns with convex distal edge in at least some anterior tooth crowns in labial view (303: 0->2), posterior cervical and/or anterior dorsal vertebrae with postzygodiapophyseal lamina (318: 0->1), anterior dorsal vertebrae with ratio between transverse width of diapophysis and length of the centrum >0.75 (357: 0->1), coracoid with subglenoid lip lateral extension strongly developed as a shelf-like structure, more developed than the supraglenoid lip on the scapula (400: 0->1), femur with distal articular surface uneven, lateral (= fibular) condyle projecting distally distinctly beyond medial (= tibial) condyle (512: 1->0), distal pedal phalanges with lateral and medial sides converging anteriorly on distal articular portion (585: 0->1), and ilium with postacetabular process subtriangular, tapering posteriorly, in lateral view (613: 0->1).

The position of the new taxon within Azendohsauridae (in both MPTs) is supported by external nares confluent with each other (9: 0->1), quadrate medial ventral condyle distinctly more ventrally projected than the lateral one (183: 0->1), labiolingual compression of the marginal dentition (305: 0->1), middle-posterior dorsal vertebrae with hyposphene-hypantrum accessory intervertebral articulation (359: 0->1), scapula and coracoid lacking fusion between each other in mature individuals (384:0->1), humerus with transverse width of the proximal end versus total length of the bone in mature individuals = 0.44−0.70 (416: 0->1), pubis with prominent tuberosity for the attachment of the ambiens muscle in mature

individuals (474: 1->0), pubic shaft rod-like in lateral view (476: 0->1), and femur with distal transverse width versus total length = 0.26−0.36 (510: 1->2).

The following character-states are recovered as synapomorphies of the *Shringasaurus* + *Azendohsaurus* clade: parietal with posterolateral process ventrally inclined in an angle greater than 45º (167: 0->1), dentary with Meckelian groove restricted to the ventral border

on the anterior half of the bone (270: 0->1), scapula lacking strong curvature or inflexion between the proximal end and the posterior margin of the blade (389: 1->0), pubic apron present, symphysis restricted anteriorly and obscured by the pubic shaft in lateral view (477: 0->1), femoral attachment of the caudofemoralis musculature on the posterior surface of the bone crest-like and with intertrochanteric fossa (= internal trochanter), and not convergent with proximal end (504: 0->1), and dorsal vertebrae with spinoprezygapophyseal lamina (607: 0->1).

Finally, *Shringasaurus* lacks the following synapomorphies of the genus *Azendohsaurus*: multiple maxillary and dentary tooth crowns with distinct mesiodistal expansion above the root (308: 0->1), and maxilla with medial surface dorsal to tooth row with prominent anteroposteriorly oriented ridge (602: 0->1).

An interesting result of this analysis is the position of *Pamelaria* as the most basal azendohsaurid. The following 13 synapomorphies support the position of this taxon within Azendohsauridae: external nares confluent with each other (9: 0->1), maxilla with a distinct ascending process that has a posteriorly concave margin (58: 0->1), postorbital with extension of the ventral process ending close to or at the ventral border of the orbit (131: 0->1), quadrate medial ventral condyle distinctly more ventrally projected than the lateral one (183: 0->1), ectopterygoid lacking contact with maxilla (206: 1->0), labiolingual compression of the marginal dentition (305: 0->1), middle-posterior dorsal vertebrae with hyposphene-hypantrum accessory intervertebral articulation (359: 0->1), scapula and coracoid lacking fusion between each other in mature individuals (384:0->1), humerus with transverse width of the proximal end versus total length of the bone in mature individuals = 0.44−0.70 (416: 0->1), pubis with prominent tuberosity for the attachment of the ambiens muscle in mature individuals (474: 1->0), pubic shaft rod-like in lateral view (476: 0->1), ischium symphysis raised on a distinct low peduncle (486: 0->1), and femur with distal transverse width versus total length = 0.26−0.36 (510: 1->2). Four additional steps are necessary to force the placement of *Pamelaria* as the most basal allokotosaurian, but only two to be found as the most basal trilophosaurid. Thus, the phylogenetic position of *Pamelaria* within Allokotosauria still requires further study.

The results recovered when considering *Trilophosaurus jacobsi* and *Spinosuchus caseanus* as independent terminals were topologically completely congruent with those found in the first analysis (Fig. S6). After the search were found two most parsimonious trees (MPTs) of 2786 steps of CI: 0.2904 and RI: 0.6317. Best score hit 73 of 100 times. *Spinosuchus caseanus* was recovered as more closely related to *Trilophosaurus jacobsi* than to other trilophosaurids (Fig. S6), as it was found by Nesbitt et al.^3^. This result is consistent with the hypothesis of synonym between both species. The Bremer supports closely resemble those of the first analysis, but the resampling frequencies are slightly lower.


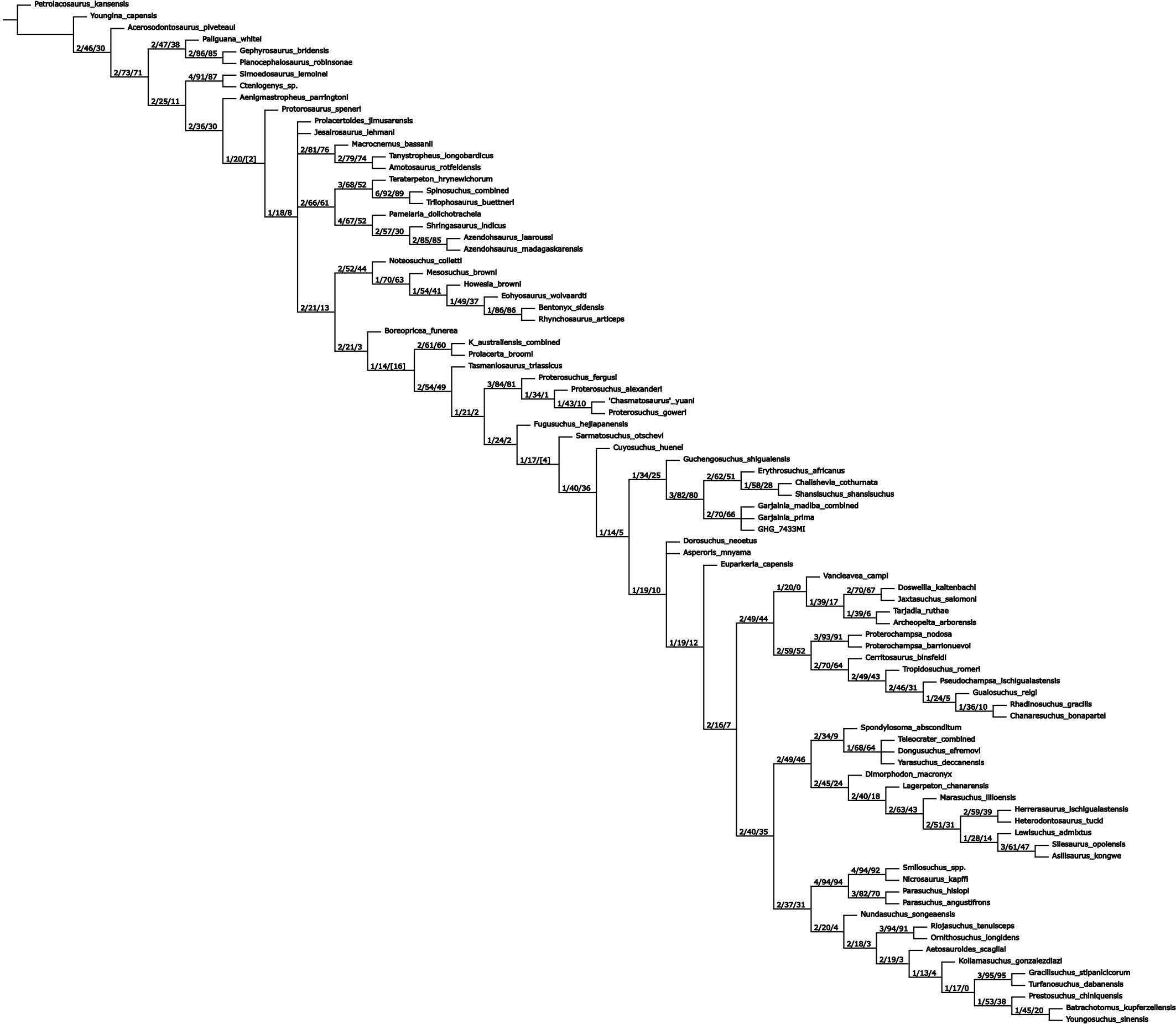


**Figure S4.** Strict consensus tree recovered from the two MPTs using “*Spinosuchus* combined” as active terminal. Numbers above each branch are Bremer values, bootstrap absolute frequencies, and bootstrap GC frequencies, respectively.


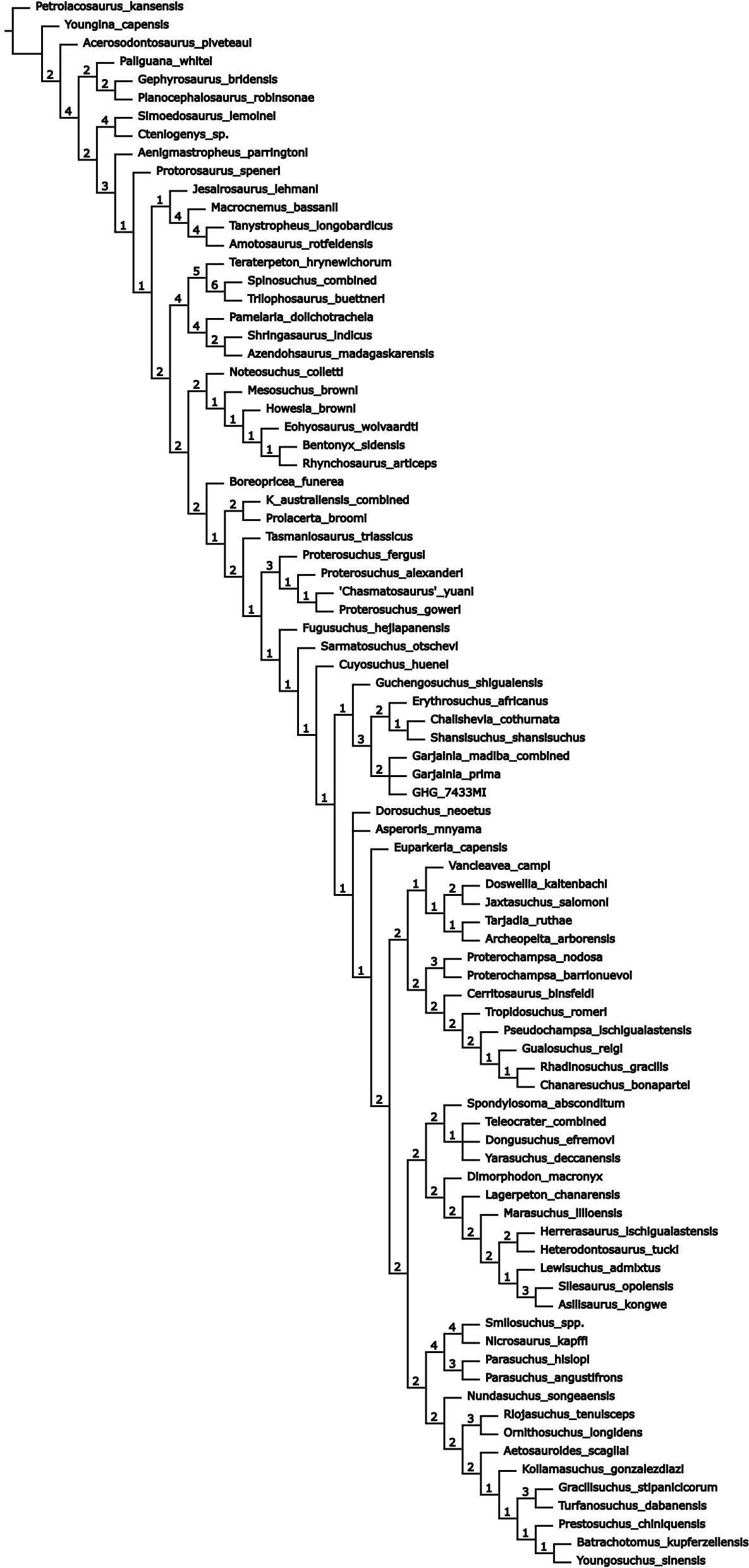


**Figure S5.** Strict reduced consensus tree recovered from the two MPTs after the a posteriori pruning of *Prolacertoides*. Numbers above each branch are Bremer values calculated after the a posteriori pruning of *Prolacertoides* and *Azendohsaurus laaroussi*.


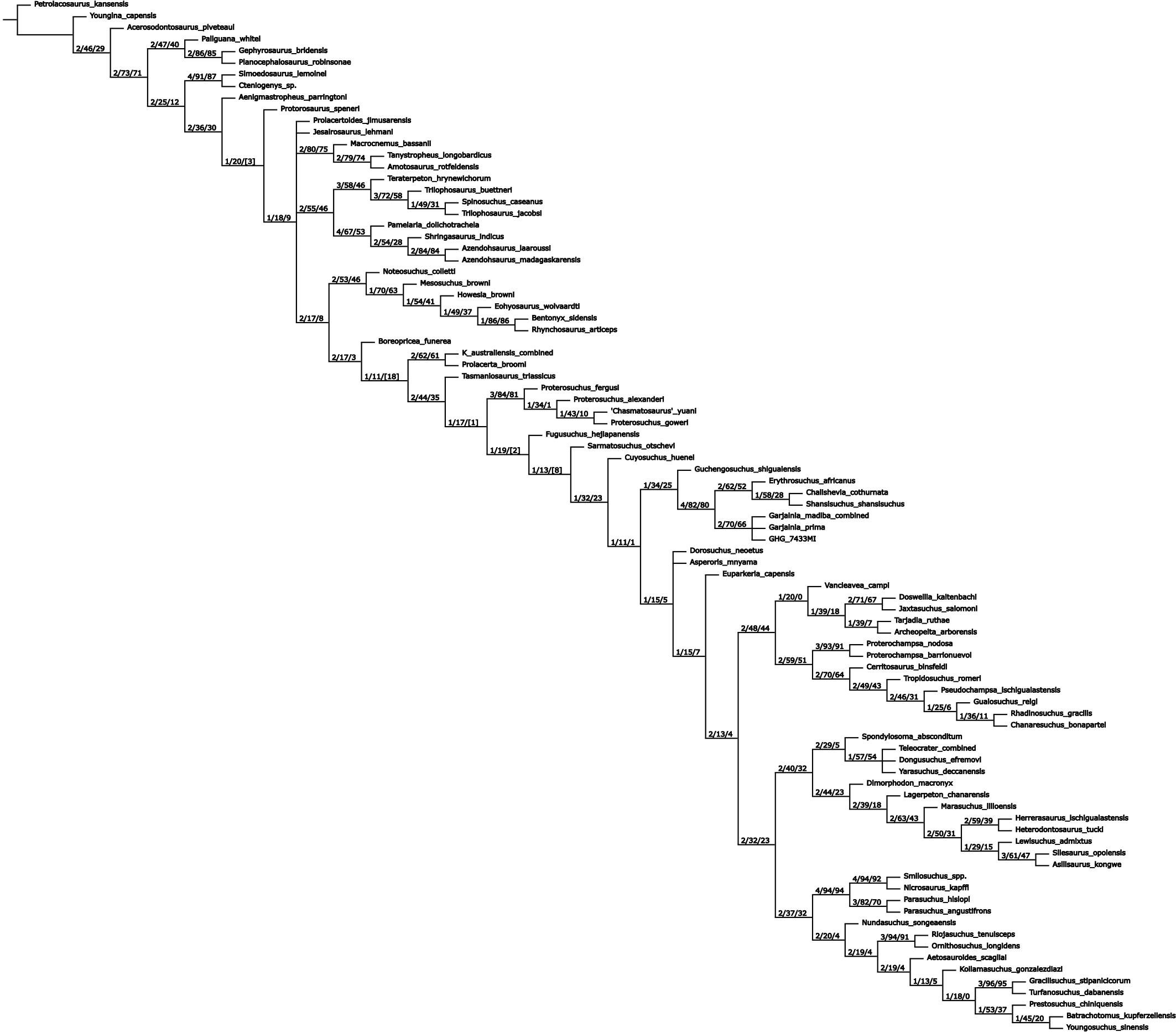
**Figure S6.** Strict consensus tree recovered from the two MPTs using *Spinosuchus* *caseanus* and *Trilophosaurus jacobsi* as independent terminals. Numbers above each branch are Bremer values, bootstrap absolute frequencies, and bootstrap GC frequencies, respectively.

**9. Character support for key nodes**

Character-states present in *Shringasaurus indicus* are highlighted in bold and underlined. All character-states are synapomorphies (unambiguous) present in all the recovered MPTs of the analysis using a combined *Spinosuchus* (i.e. *Trilophosaurus jacobsi* + *Spinosuchus caseanus*).

**Sauria**

146 2🡪0

**160 1🡪0**

**176 0🡪1**

404 0🡪1

**475 2🡪0**

**Archosauromorpha (including choristoderans)**

6 1🡪0

156 0🡪1

**166 0🡪1**

**229 0🡪1**

270 1🡪0

**426 0🡪1**

**Archosauromorpha (excluding choristoderans)**

**313 0🡪1**

**315 0🡪1**

**316 0🡪1**

**317 0🡪1**

***Protorosaurus +* more derived archosauromorphs**

**355 1🡪0**

**429 0🡪1**

**Tanystropheidae *+* Crocopoda**

**36 1🡪2**

**347 0🡪1**

**464 0🡪1**

**491 0🡪1**

557 0🡪1

**Crocopoda**

**8 0/2🡪1**

**37 1🡪0**

168 0🡪1

215 0🡪1

219 0🡪1

255 0🡪1

284 0🡪1

**299 0🡪1** (only in one of the trees)

**532 0🡪2**

**539 1🡪0**

545 0🡪1

**Allokotosauria (in the tree that does not recover *Prolacertoides* as an allokotosaurian)**

21 1🡪2

59 0🡪1

**75 3🡪1/2**

111 0🡪1 (reversed in *Shringasaurus*)

149 0🡪1

**180 0🡪1**

**189 0🡪1**

190 0🡪1

**227 0🡪1**

283 2🡪1

**303 0🡪2**

**318 0🡪1**

**357 0🡪1**

**400 0🡪1**

433 0🡪1

451 0🡪1

453 0🡪1

**512 1🡪0**

**585 0🡪1**

587 0🡪1

**613 0🡪1**

**Azendohsauridae**

**9 0🡪1**

58 0🡪1

131 0🡪1

**183 0🡪1**

206 1🡪0

**305 0🡪1**

**359 0🡪1**

**384 0🡪1**

**416 0🡪1**

**474 1🡪0**

**476 0🡪1** (*Shringasaurus* could have state 1 or 2)

486 0🡪1

**510 1🡪2**

***Shringasaurus + Azendohsaurus***

**167 0🡪1**

**239 0🡪1**

**270 0🡪1**

**389 1🡪0**

**477 0🡪1**

**504 0🡪1**

**607 0🡪1**

***Shringasaurus* (autapomorphies)**

**30 0🡪1**

**111 1🡪0**

**233 0🡪1**

**320 0🡪2**

**332 2🡪1**

**402 0🡪1**

**424 1🡪2**

**466 0🡪1**

**608 0🡪1**

**609 0🡪1**

**611 1🡪0**

***Azendohsaurus***

308 0🡪1

602 0🡪1

**Trilophosauridae**

18 0🡪1

19 1🡪0

42 2🡪5

69 0🡪1

137 0🡪1

343 0🡪1

398 1🡪2

604 0🡪1

***Spinosuchus + Trilophosaurus***

22 0🡪1

95 0🡪1

123 0🡪1

260 0🡪2

305 0🡪2

601 0🡪1

605 1🡪2

606 0🡪1

**Body size analysis**

The optimization of femoral length on a pruned topology based on the results recovered in our phylogenetic analysis required a total of 49.330 steps (Fig. S7). The CI of the character was 0.4918 and the RI: 0.5932. These results were used to biuld Figure 4b, with the addition by hand of *Stenaulorhynchus* as the sister-taxon of *Rhynchosaurus* based on the results of Dilkes^12^.


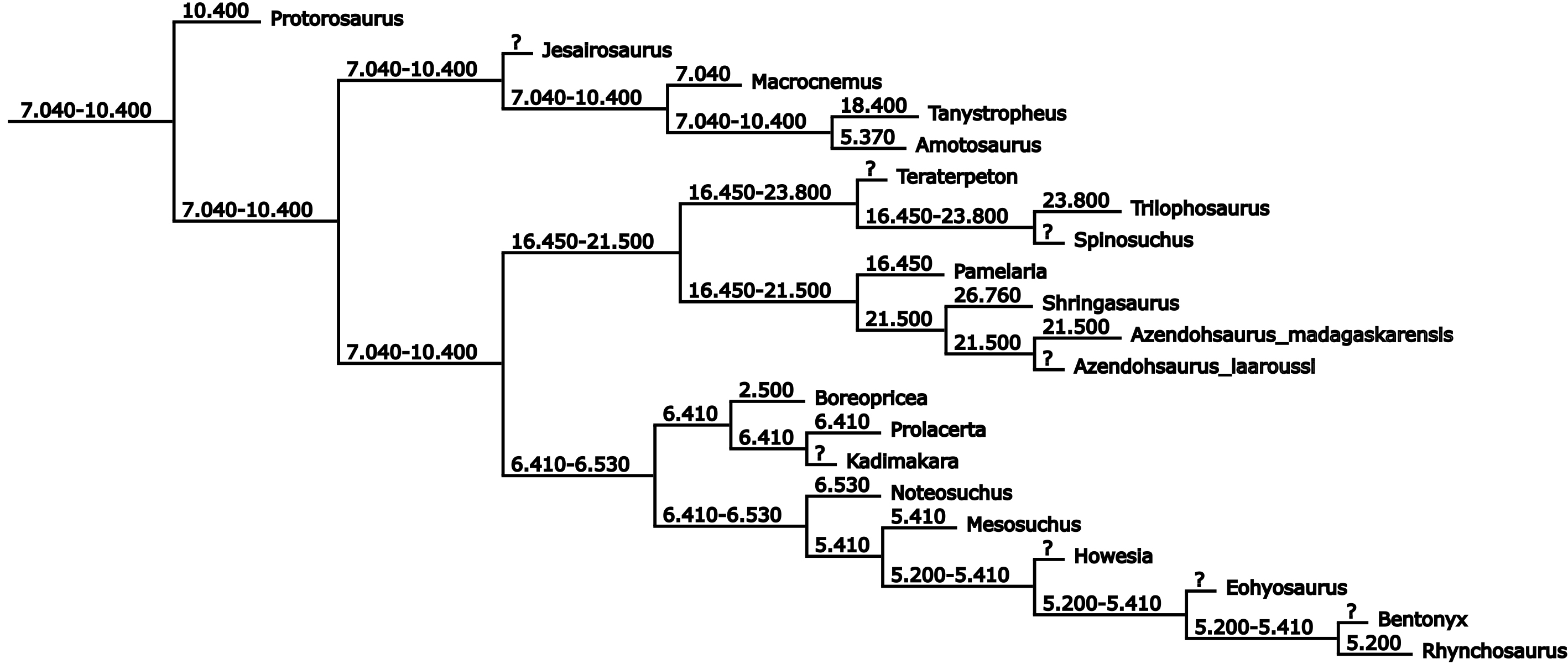


**Figure S7.** Optimization of femoral length as a continuous character using maximum parsimony.

Measurements used in the optimization of femoral length as a continuous character:

*Pamelaria* (ISIR 316/55): 164.5 mm (pers. obs.)

*Shringasaurus* (ISIR 1016): 267.6 mm (this study)

*Azendohsaurus madagaskarensis* (FMNH PR 2799): 215 mm^3^

*Trilophosaurus buettneri* (NMMNH P-31025-694): 238 mm^18^

*Rhynchosaurus* (NHMUK PV R1239): 52 mm^20^

*Noteosuchus* (AM 3591): 65.3 mm (pers. obs.)

*Prolacerta* (BP/1/2676): 64.1 mm (pers. obs.)

*Mesosuchus* (SAM-PK-7416): 54.1 mm (pers. obs.)

*Stenaulorhynchus* (GPIT specimen): 180 mm^21^

*Macrocnemus* (PIMUZ T2472): 70.4 mm^22^

*Amotosaurus* (SMNS 54810): 53.7 mm (pers. obs.)

*Tanystropheus* (SMNS 54621): 184 mm (pers. obs.)

*Protorosaurus* (Nat. Kab. 191): 104 mm^23^

*Boreopricea* (PIN 3708/1): 25 mm^24^

**Further comments on the cranial horns of *Shringasaurus* and the sexual selection hypothesis**

Tobias et al.^25^ defined sexual selection as a subset of social competition in which the resource at stake is mates. These authors included social dominance as a particular case of social selection and this interpretation is followed here. At a broader level, the most accepted view is that social selection is a subset of natural selection^25−28^. This definition of sexual selection agrees with the original proposal of Darwin (1859, p. 156)^28^ that this kind of evolutionary mechanism depends ‘not on a struggle for existence, but on a struggle between the males for possession of the females; the result is not death to the unsuccessful competitor, but few or no offspring.’ Sexual selection usually acts on secondary sexual characters that may or may not be restricted to a single sex, such as ornaments and weapons^26^.

The supraorbital horns of *Shringasaurus* are here interpreted as a secondary sexual character. These structures should have resulted in a physiologically costly phenotype − because of its investment in growth, transport, and maintenance – and show strong positive allometry − smaller individuals possess proportionally smaller and more gracile horns than larger individuals. The maintenance and transport of large horns that approach the height of the rest of the skull below them should have very likely involved a considerable higher amount of energy than the plesiomorphic unhorned phenotype of allokotosaurians. The exacerbation of the size and robustness of the horns during ontogeny also suggests that this phenotype should have been more costly in adult specimens than in juveniles. The presence of a physiologically costly phenotype implies that the horns of *Shringasaurus* were not a non-functional character because it should have reduced the fitness of the individual and, as a consequence, should have been negatively selected under non-sexual natural selection. The above mentioned traits (i.e. costliness, positive allometry) characterize sexually selected features and, as a result, have been considered as key criteria to distinguish secondary sexual characters in the fossil record^29−35^. In addition, the fact that the horns of *Shringasaurus* appear to be intraspecifically dimorphic (probably sexually dimorphic) may bolster the sexual selection hypothesis to explain the origin of these structures^36−40^. The presence of substantial variation in the morphology of the horns of *Shringasaurus* (size and shape) and their costliness weakens a species recognition hypothesis as a possible explanation. Knell and Sampson^29^ stated that sexually selected traits frequently, though not invariably, show condition-dependent expression leading to a great deal of intraspecific variation and strong positive allometry^41−44^.

Berglung et al.^45^ found that in the majority of cases secondary sexual characters serve a dual function as both armament and ornament (e.g. the horns of *Ovis canadensis*) and the frequency of a single function is skewed in favour of the armament function. Signals used in contests are honest because they are relatively more costly to produce for low quality males and because they are constantly tested in combat with other males. Females benefit by subsequently using the information about male quality contained in these male signals to select high-quality males^45^. Conical and non-branched or poorly branched horns are used as weapons by extant mammals, lepidosaurs, and insects^46^. The similarity of the horns of extinct dinocephalian synapsids and ceratopsian dinosaurs to those of bovids and chamaeleonids have been used to infer that these structures were also used as weapons^47,48^. As a result, the conical, robust, and non-branched supraorbital horns of *Shringasaurus* can be interpreted as sexually selected weapons based on their close resemblance to the horns of the above mentioned extant and extinct amniotes and the fact that armaments are common structures among the diversity of secondary sexual characters of extant animals. The presence of a pachyostotic skull roof, with prefrontal, nasal, frontal, and postfrontal fused to each other, in *Shringasaurus* may also support this interpretation.

No cornified (i.e. heavily keratinized) sheath has been found on the preserved supraorbital horns or elsewhere in the *Shringasaurus* bone-bed. However, the preservation of such structure in the Palaeozoic and Mesozoic fossil record is null or restricted to a single possible case. Hatcher et al. (1907, p. 32)^49^ reported that when the ceratopsid *Triceratops* specimen YPM 1821 was discovered, “a portion of the investing horny material was still in place about the left horn core, though in such a decomposed condition that it was impossible to preserve it.” Hieronymus et al.^50^ proposed a series of osteological correlates for the inference of the presence of cornified sheaths in fossil specimens (e.g. ceratopsids) based on extant analogues. Bone surface ornamented by tangential rugosities and dense neurovascular grooves are among the osteological correlates described by these authors and they are present in all the preserved horns of *Shringasaurus*. Thus, this evidence supports the presence of a cornified sheath on the horns of the new Indian species.

Farke^51^ pointed out that the combat behaviour of the extinct ceratopsid dinosaurs cannot be directly inferred from that of bovids because of the disparate phylogenetic origin, unique horn orientation, and other unusual cranial features (such as nasal horns and frills) found in ceratopsids. Here we agree with this statement, but at the same time we consider that it is interesting to do some comparisons between the horn morphology of *Shringasaurus* and that of extant bovids and its relationship with behaviour in order to draw some possible conclusions and generate working hypotheses to be tested in the future. The horns of large-sized specimens of *Shringasaurus* are anteriorly curved, relatively short (in comparison with most bovids), a very low percentage of the length of the horn is occupied by the catching arch [the curved region between stem and stabbing zones; after Kingdon^52^ and Lundrigan^53^], and the tips are directed anterodorsally. Lundrigan^53^ found the following correlations between the horn morphology and combat behaviour in bovids:

- Strongly recurved horns (e.g. mountain sheep, contrasting with the moderately curved horns of e.g. the reedbuck or *Shringasaurus*) are positively correlated with ramming (evidence against ramming for *Shringasaurus*).

- Higher circumference at the base of the horn is positively correlated with ramming (ambiguous for *Shringasaurus*).

- Percentage of the length of the horn occupied by the catching arch is positively correlated with wrestling (more curved horns) and negatively correlated with stabbing (straighter horns). Curves of the catching arch provide a surface for holding and locking horns during wrestling matches^54,55^ (evidence for wrestling and against stabbing for *Shringasaurus*).

- Long horn is positively correlated with fencing and negatively correlated with stabbing (evidence against fencing and possibly for stabbing for *Shringasaurus*).

- Angle formed by palatal plane and stabbing zone is positively correlated with kneeling. Kneeling may be advantageous to species that have horn tips that are directed posteriorly, or posteriorly and ventrally (evidence against kneeling for *Shringasaurus*).

As a result, the morphology of the horns of *Shringasaurus* suggests standing wrestling as a possible combat behaviour, resembling the bovid *Redunca arundinum* (reedbuck)^56,57^. It is interesting to note that a wrestling combat behaviour has been also inferred for the ceratopsid *Triceratops* by Farlow & Dodson^48^. This hypothesis can be tested in the future with quantitative biomechanical analyses (e.g. Finite Elements Analysis) that may explore the capability of the skull and postcranium of *Shringasaurus* for such behaviour.

**Institutional Abbreviations.** AM, Albany Museum, Grahamstown, South Africa; BP, Evolutionary Studies Institute (formerly Bernard Price Institute for Palaeontological Research), University of the Witwatersrand, Johannesburg, South Africa; FMNH, Field Museum of Natural History, Chicago, USA; GPIT, Paläontologische Sammlung der Universität Tübingen, Tübingen, Germany; ISIR, Indian Statistical Institute, Reptile, Kolkata, India; MNHN, Muséum national d'Histoire naturelle, Paris, France; Nat. Kab., Naturalienkabinett und Heimatmuseum, Waldenburg, Germany; NHMUK PV, The Natural History Museum, Palaeontology Vertebrates, London, UK; NMMNH, New Mexico Museum of Natural History, Albuquerque, USA; PIMUZ, Paläontologisches Institut und Museum der Universität Zürich, Zurich, Switzerland; PIN, Paleontological Institute of the Russian Academy of Sciences, Moscow, Russia; ROM, Royal Ontario Museum, Toronto, Ontario, Canada; SAM-PK, Iziko South African Museum, Cape Town, South Africa; SMNS, Staatliches Museum für Naturkunde Stuttgart, Stuttgart, Germany; YPM, Yale Peabody Museum, New Haven, Connecticut, USA.

**Supplementary data**

Supplementary Data SD1: Nexus file of the phylogenetic data matrix used in this study.

Supplementary Data SD2: TNT file of the phylogenetic data matrix used in this study.

**Supplementary references**

1. Osborn, H. F. The reptilian subclasses Diapsida and Synapsida and the early history of the Diaptosauria. *Memoirs of the American Museum of Natural History* **1**, 449−519 (1903).
2. Huene, F. von. Die grossen Stämme der Tetrapoden in den geologischen Zeiten. *Biologisches Zentralblatt* **65**, 268−275 (1946).
3. Nesbitt, S. J. *et al*. Postcranial anatomy and relationships of *Azendohsaurus madagaskarensis*. *Bulletin of the American Museum of Natural History* **398**, 1−126 (2015).
4. Chatterjee, S. *Malerisaurus*, a new eosuchian reptile from the Late Triassic of India. Philosophical Transactions of the Royal Society of London B **291**, 163−200 (1980).
5. Chatterjee, S. *Malerisaurus langstoni*, a new diapsid reptile from the Triassic of Texas. *Journal of Vertebrate Paleontology* **6**, 297−312 (1986).
6. Arkhangelskii, M. S. & Sennikov, A. G. in *Fossil vertebrates of Russia and adjacent countries: Fossil reptiles and birds. Part 1* (eds M.F. Ivakhnenko & E.N. Kurotchkin) 224−243 (GEOS, Moscow, 2008).
7. Robinson, P. L. An unusual sauropsid dentition. *Journal of the Linnean Society of London, Zoology* **43**, 283−293 (1957).
8. Sues, H.-D. *Arctosaurus osborni*, a Late Triassic archosauromorph reptile from the Canadian Arctic Archipelago. *Canadian Journal of Earth Sciences* **54**, 129−133 (2017).
9. Ezcurra, M. D. The phylogenetic relationships of basal archosauromorphs, with an emphasis on the systematics of proterosuchian archosauriforms. *PeerJ* **4**, e1778 (2016).
10. Pritchard, A. C., Turner, A. H., Nesbitt, S. J., Irmis, R. B. & Smith, N. D. Late Triassic tanystropheids (Reptilia, Archosauromorpha) from northern New Mexico (Petrified Forest Member, Chinle Formation) and the biogeography, functional morphology, and evolution of Tanystropheidae. *Journal of Vertebrate Paleontology* **35**, e911186 (2015).
11. Nesbitt, S. J. *et al*. The earliest bird-line archosaurs and the assembly of the dinosaur body plan. *Nature* **544**, 484−487 (2017).
12. Dilkes, D. W. The Early Triassic rhynchosaur *Mesosuchus browni* and the interrelationships of basal archosauromorph reptiles. *Philosophical Transactions of the Royal Society of London B* **353**, 501−541 (1998).
13. Reisz, R. R. & Dilkes, D. W. *Archaeovenator hamiltonensis*, a new varanopid (Synapsida: Eupelycosauria) from the upper Pennsylvanian of Kansas. *Canadian Journal of Earth Sciences* **40**, 667−678 (2003).
14. Reisz, R. R., Laurin, M. & Marjanović, D. *Apsisaurus witteri* from the Lower Permian of Texas: yet another small varanopid synapsid, not a diapsid. *Journal of Vertebrate Paleontology* **30**, 1628−1631 (2010).
15. Ezcurra, M. D., Lecuona, A. & Martinelli, A. A new basal archosauriform diapsid from the Lower Triassic of Argentina. *Journal of Vertebrate Paleontology* **30**, 1433−1450 (2010).
16. Ezcurra, M. D., Scheyer, T. M. & Butler, R. J. The origin and early evolution of Sauria: reassessing the Permian saurian fossil record and the timing of the crocodile-lizard divergence. *PLoS ONE* **9**, e89165 (2014).
17. Sues, H.-D. An unusual new archosauromorph reptile from the Upper Triassic Wolfville Formation of Nova Scotia. *Canadian Journal of Earth Sciences* **40**, 635−649 (2003).
18. Spielmann, J. A., Lucas, S. G., Rinehart, L. F. & Heckert, A. B. 2008. The Late Triassic archosauromorph *Trilophosaurus*. *Bulletin of the New Mexico Museum of Natural History and* *Sciences* **43**, 1−177 (2008).
19. Spielmann, J. A., Lucas, S. G., Heckert, A. B., Rinehart, L. F. & Richards III, H. R. Redescription of *Spinosuchus caseanus* (Archosauromorpha: Trilophosauridae) from the Upper Triassic of North America. *Palaeodiversity* **2**, 283−313 (2009).
20. Benton, M. J. The species of *Rhynchosaurus*, a rhynchosaur (Reptilia, Diapsida) from the Middle Triassic of England. *Philosophical Transactions of the Royal Society of London B* **328**, 213−306 (1990).
21. Huene, F. von. Die fossilen Reptilien des südamerikanischen Gondwanalandes. *Neues Jahrbuch für Mineralogie, Geologie und Paläontologie, Abteilung B* **1938**, 142−151 (1938).
22. Rieppel, O. The hind limb of *Macrocnemus bassanii* (Nopcsa) (Reptilia, Diapsida): development and functional anatomy. *Journal of Vertebrate Paleontology* **9**, 373−387 (1989).
23. Gottmann-Quesada, A. & Sander, P. M. A redescription of the early archosauromorph *Protorosaurus spenseri* (Meyer, 1832) and its phylogenetic relationships. *Palaeontographica Abteilung A* **287**, 123−220 (2009).
24. Benton, M. J. & Allen, J. L. *Boreopricea* from the Lower Triassic of Russia, and the relationships of the prolacertiform reptiles. *Palaeontology* **40**, 931−953 (1997).
25. Tobias, J. A., Montgomerie, R. & Lyon, B. E. The evolution of female ornaments and weaponry: social selection, sexual selection and ecological competition. Philosophical Transactions of the Royal Society B **367**, 2274–2293 (2012).
26. Darwin, C. *The descent of man and selection in relation to sex* (John Murray, London, 1871).
27. Andersson, M. *Sexual selection* (Princeton Univeristy Press, Princeton, 1994).
28. Darwin, C. *The origin of species by means of natural selection* (John Murray, London, 1859).
29. Knell, R. J. & Sampson, S. Bizarre structures in dinosaurs: species recognition or sexual selection? A response to Padian and Horner. *Journal of Zoology* **283**, 18–22 (2011).
30. Hone, D. W. E., Naish, D. & Cuthill, I. C. Does mutual sexual selection explain the evolution of head crests in pterosaurs and dinosaurs? *Lethaia* **45**, 139–156 (2012).
31. Hone, D. W. E. & Naish, D. The ‘species recognition hypothesis’ does not explain the presence and evolution of exaggerated structures in nonavialan dinosaurs. *Journal of Zoology* **290**, 172–180 (2013).
32. Knell, R. J., Naish, D., Tomkins, J. L. & Hone, D. W.E. Sexual selection in prehistoric animals: detection and implications. *Trends in Ecology and Evolution* **28**, 38–47 (2013a).
33. Knell, R. J., Naish, D., Tomkins, J. L. & Hone, D. W. E. Is sexual selection defined by dimorphism alone? A reply to Padian and Horner. *Trends in Ecology and Evolution* **28**, 250–251 (2013b).
34. Borkovic, B. & Russell, A. Sexual selection according to Darwin: a response to Padian and Horner’s interpretation. *Comptes Rendus Palevol* **13**, 701–707 (2014).
35. Hone, D. W. E. & Faulkes, C. G. A proposed framework for establishing and evaluating hypotheses about the behaviour of extinct organisms. *Journal of Zoology* **292**, 260–267 (2014).
36. Padian, K. & Horner, J. R. The evolution of ‘bizarre structures’ in dinosaurs: biomechanics, sexual selection, social selection or species recognition? *Journal of Zoology* **283**, 3–17 (2011a).
37. Padian, K. & Horner, J. R. The definition of sexual selection and its implications for dinosaurian biology. *Journal of Zoology* **283**, 23–27 (2011b).
38. Padian, K. & Horner, J. R. Misconceptions of sexual selection and species recognition: a response to Knell et al and to Mendelson and Shaw. *Trends in Ecology and Evolution* **28**, 249–250 (2013).
39. Padian, K. & Horner, J. R. The species recognition hypothesis explains exaggerated structures in non-avialan dinosaurs better than sexual selection does. *Comptes Rendus Palevol* **13**, 97–107 (2014).
40. Maynard Smith, J. & Harper, D. *Animal signals*. (Oxford University Press, Oxford, 2003).
41. Cotton, S., Fowler, K. & Pomiankowski, A. Do sexual ornaments demonstrate heightened condition-dependent expression as predicted by the handicap hypothesis? *Proceedings of the Royal Society of London, Series B* **271**, 771−783 (2004).
42. Tomkins, J. L., Radwan, J., Kotiaho, J. S. & Tregenza, T. Genic capture and resolving the lek paradox. Trends in Ecology and Evolution **19**, 323–328 (2004).
43. Tomkins, J. L., LeBas, N. R., Witton, M. P., Martill, D. M. & Humphries, S. Positive allometry and the prehistory of sexual selection. *American Naturalist* **176**, 141–148 (2010).
44. Bonduriansky, R. Sexual selection and allometry: a critical reappraisal of the evidence and ideas. *Evolution* **61**, 838–849 (2007).
45. Berglung, A., Bisazza, A. & Pilastro, A. Armaments and ornaments: an evolutionary explanation of traits of dual utility. *Biological Journal of the Linnean Society* **58**, 385–399 (1996).
46. McCullough, E. L., Miller, C. W. & Emlen, D. J. Why sexually selected weapons are not ornaments. *Trends in Ecology and Evolution* ***31***, 742−751 (2016).
47. Barghusen, H. R. A review of fighting adaptations in dinocephalians (Reptilia, Therapsida). *Paleobiology* **1,** 295−311 (1975).
48. Farlow, J. O. & Dodson, P. The behavioral significance of frill and horn morphology in ceratopsian dinosaurs. *Evolution* **29**, 353−361 (1975).
49. Hatcher, J. B., Marsh, O. C. & Lull, R. S. The Ceratopsia. *United States Geological Survey Monograph* **49**, 1−300 (1907).
50. Hieronymus, T. L., Witmer, L. M., Tanke, D. H. & Currie, P. J. The Facial Integument of Centrosaurine Ceratopsids: Morphological and Histological Correlates of Novel Skin Structures. *The Anatomical Record* **292,** 1370−1396 (2009).
51. Farke, A. A. Horn Use in Triceratops (Dinosauria: Ceratopsidae): Testing Behavioral Hypotheses Using Scale Models. *Palaeontologia Electronica* **7**, 1−10 (2004).
52. Kingdon, J. *East African mammals; an atlas of evolution in Africa (Bovids)*. (Academic Press, New York, 1982).
53. Lundrigan, B. Morphology of horns and fighting behavior in the Family Bovidae. *Journal of Mammalogy* **77**, 462−475 (1996).
54. Geist, V. The evolution of horn-like organs. *Behaviour* **27**, 175−214 (1966).
55. Walther, F. Von Waffen und Kampfen bei Horntieren. *Kosmos* **62**, 208−214 (1966).
56. Jungius, H. *The biology and behaviour of reedbuck (*Redunca arundinum *Boddaert 1758) in the Kruger National Park*. (Verlag Paul Parey, Berlin, 1971).
57. Smithers, R. *The mammals of the southern African subregion*. (University of Pretoria, Pretoria, 1983).
