# Supplementary material for: A new horned and long-necked herbivorous stem-archosaur from the Middle Triassic of India
Source: Sci Rep. 2017 Aug 21;7:8366. doi: 10.1038/s41598-017-08658-8 (PMC5567049; doi:10.1038/s41598-017-08658-8)
Supplement: Supplementary file 2 — data matrix NEXUS [file 41598_2017_8658_MOESM2_ESM.doc]

#NEXUS

[written Thu Nov 17 18:17:29 ART 2016 by Mesquite version 2.75 (build 564) at Proterosuchia/192.168.0.103]

BEGIN TAXA;

TITLE Untitled_Taxa_Block;

DIMENSIONS NTAX=111;

TAXLABELS

Petrolacosaurus_kansensis Acerosodontosaurus_piveteaui Youngina_capensis Paliguana_whitei Planocephalosaurus_robinsonae Gephyrosaurus_bridensis Cteniogenys_sp. Simoedosaurus_lemoinei Aenigmastropheus_parringtoni Protorosaurus_speneri Amotosaurus_rotfeldensis Macrocnemus_bassanii Tanystropheus_longobardicus Jesairosaurus_lehmani Pamelaria_dolichotrachela Azendohsaurus_madagaskarensis Azendohsaurus_laaroussi Shringasaurus_indicus Trilophosaurus_buettneri Trilophosaurus_jacobsi Spinosuchus_caseanus Spinosuchus_combined Teraterpeton_hrynewichorum Noteosuchus_colletti Mesosuchus_browni Howesia_browni Eohyosaurus_wolvaardti Rhynchosaurus_articeps Bentonyx_sidensis Eorasaurus_olsoni Prolacertoides_jimusarensis Prolacerta_broomi K_australiensis_holotype K_australiensis_combined Boreopricea_funerea Archosaurus_rossicus_holotype '''Proterosuchus ferugsi''' Proterosuchus_fergusi Proterosuchus_goweri Proterosuchus_alexanderi '''Chasmatosaurus'' yuani' '''Chasmatosaurus ultimus''' Ankistrodon_indicus Tasmaniosaurus_triassicus Exilisuchus_tubercularis Blomosuchus_georgii Vonhuenia_fredericki C_rossicus_combined Chasmatosuchus_magnus Gamosaurus_lozovskii C_magnus_combined Chasmatosuchus_vjushkovi SAM_P41754_Long_Reef Koilamasuchus_gonzalezdiazi Kalisuchus_rewanensis_holotype Fugusuchus_hejiapanensis Sarmatosuchus_otschevi Guchengosuchus_shiguaiensis Cuyosuchus_huenei GHG_7433MI Garjainia_prima Garjainia_madiba_holotype Garjainia_madiba_combined Erythrosuchus_africanus Shansisuchus_shansisuchus Shansisuchus_kuyeheensis Chalishevia_cothurnata Youngosuchus_sinensis '''Dongusia colorata''' Uralosaurus_holotype Uralosaurus_combined Vancleavea_campi Asperoris_mnyama Euparkeria_capensis Dorosuchus_neoetus Proterochampsa_barrionuevoi Proterochampsa_nodosa Tropidosuchus_romeri Cerritosaurus_binsfeldi Gualosuchus_reigi Chanaresuchus_bonapartei Pseudochampsa_ischigualastensis Rhadinosuchus_gracilis Archeopelta_arborensis Tarjadia_ruthae Jaxtasuchus_salomoni Doswellia_kaltenbachi Parasuchus_angustifrons Parasuchus_hislopi Nicrosaurus_kapffi Smilosuchus_spp. Ornithosuchus_longidens Riojasuchus_tenuisceps Nundasuchus_songeaensis Turfanosuchus_dabanensis Gracilisuchus_stipanicicorum Aetosauroides_scagliai Batrachotomus_kupferzellensis Prestosuchus_chiniquensis Dimorphodon_macronyx Lagerpeton_chanarensis Marasuchus_lilloensis Lewisuchus_admixtus Asilisaurus_kongwe Silesaurus_opolensis Heterodontosaurus_tucki Herrerasaurus_ischigualastensis Yarasuchus_deccanensis Dongusuchus_efremovi Teleocrater_combined Spondylosoma_absconditum

;

END;

BEGIN CHARACTERS;

TITLE Untitled_Character_Matrix;

DIMENSIONS NCHAR=620;

FORMAT DATATYPE = STANDARD GAP = - MISSING = ? SYMBOLS = " 0 1 2 3 4 5 6";

CHARSTATELABELS

1 'Skull and lower jaws, interdental plates' / absent 'present, small and well-spaced from each other' 'present, large and close to or contacting with each other', 2 'Skull, total length versus length of the presacral vertebral column' / '0.22-0.38' '0.44-0.72' '0.94-0.98', 3 'Skull, strongly dorsoventrally compressed skull with mainly dorsally facing antorbital fenestrae and orbits' / absent present, 4 'Skull, well-developed nodular prominences on the lateral surface of maxilla, jugal, quadratojugal, squamosal and angular' / absent present, 5 'Skull, dermal sculpturing on the dorsal surface of the skull roof' / absent shallow_or_deep_pits_scattered_across_surface_and_low_ridges 'prominent ridges or tubercles on frontals, parietals, and nasals', 6 'Skull, dorsal surface of nasals and/or frontals ornamented by ridges radiating from centres of growth' / absent present, 7 'Skull, dorsal orbital margin' / orbital_margin_of_the_frontal_level_with_skull_table_or_raised_slightly orbital_margin_of_the_frontal_elevated_above_skull_table 'shelf/ridge elevated above skull table and extends along the lateral surface of the lacrimal, prefrontal, frontal portion of orbital rim, and postorbital', 8 'Skull, dorsal surface of the temporal region' / flat supratemporal_fossa_immediately_medial_or_anterior_to_the_supratemporal_fenestra 'thin, blade-like median sagittal crest', 9 'External nares, confluent' / absent present, 10 'External naris, anteroposterior position in the snout' / 'terminal, on the anterior end of the snout' 'nonterminal, considerably posteriorly displaced, but posterior rim of the naris well anterior to the anterior border of the orbit' 'nonterminal, considerably posteriorly displaced and posterior rim of the naris approximately at level with the anterior border of the orbit', 11 'External naris, directed' / laterally dorsally anteriorly, 12 'External naris, shape' / 'sub-circular' oval, 13 Antorbital_fenestra / absent present, 14 'Antorbital fenestra, anterior margin' / gently_rounded nearly_pointed, 15 'Secondary antorbital fenestra, immediately anterior to the antorbital fenestra' / absent present, 16 'Orbit, shape' / anteroposteriorly_longer_than_tall subcircular dorsoventrally_taller_than_long, 17 'Orbit, elevated rim along the jugal, postorbital, frontal, prefrontal and lacrimal' / absent_or_incipient 'present, restricted to the ascending process of the jugal and sometimes also onto the ventral process of the postorbital' 'present, well-developed along the jugal, postorbital, frontal, prefrontal and lacrimal', 18 Infratemporal_fenestra / present absent, 19 'Posttemporal fenestra, size' / larger_than_or_subequal_to_the_supraoccipital smaller_than_the_supraoccipital developed_as_a_small_foramen_ absent, 20 'Snout, antorbital length (anterior tip of the skull to anterior margin of the orbit) versus total length of the skull' / '0.29-0.40' '0.43-0.62' '0.70-0.76', 21 'Snout, dorsoventral height at the level of the anterior tip of the maxilla versus dorsoventral height at the level of the anterior border of the orbit' / '<=0.33' '0.42-0.52' '0.59-0.80', 22 'Snout, proportions at the level of the anterior border of the orbit' / transversely_broader_than_dorsoventrally_tall_or_subequal dorsoventrally_taller_than_transversely_broad, 23 'Snout, lateral margin of the snout anterior to the prefrontal' / formed_by_the_nasal formed_by_the_nasal_and_maxilla_with_gently_rounded_transition_along_the_maxilla_from_the_lateral_to_dorsal_side_of_rostrum 'formed by the nasal and maxilla with sharp edge along the maxilla between the lateral and dorsal sides of this bone (= box-like snout of Kischlat, 2000)', 24 'Premaxilla-maxilla, suture' / simple_continuous_contact notched_along_the_ventral_margin, 25 'Premaxilla-maxilla, subnarial foramen between the elements' / absent present_and_the_border_of_the_foramen_is_present_on_both_the_maxilla_and_the_premaxilla present_and_the_border_of_the_foramen_is_present_on_the_maxilla_but_not_on_the_premaxilla, 26 'Premaxilla, alveolar margin does not reach the contact with the maxilla and forms a diastema (= subnarial gap)' / absent present, 27 'Premaxilla, main body size' / 'small, the premaxillary body forms less than half of snout in front of the posterior border of the external nares' 'large, the premaxillary body forms half or more than half of snout in front of the posterior border of the external nares', 28 'Premaxilla, anteroposterior length of the main body versus its maximum dorsoventral height' / '0.70-0.73' '1.07-2.00' '2.22-3.80' '4.15-4.68' '>5.00', 29 'Premaxilla, downturned main body' / 'absent, alveolar margin sub-parallel to the main axis of the maxilla' 'slightly, alveolar margin kinked approximately 20º from the alveolar margin of the maxilla' 'strongly, prenarial process obscured by the postnarial process in lateral view (if the postnarial process is long enough) and postnarial process parallel or posteroventrally oriented with respect to the main axis of the premaxillary body', 30 'Premaxilla, angle formed between the alveolar margin and the anterior margin of the premaxillary body in lateral view' / 'acute or right-angled' obtuse, 31 'Premaxilla, longitudinal groove placed approximately at mid-height and extending along most of the length of the lateral surface of the main body of the bone' / absent present 'one longitudinal groove slightly displaced ventrally or at the point of mid-height of the main body', 32 'Premaxilla, narial fossa' / absent_or_shallow expanded_in_the_anteroventral_corner_of_the_naris_, 33 'Premaxilla, peg on the posterior edge of the premaxillary body' / absent present, 34 'Premaxilla, prenarial process length' / less_than_the_anteroposterior_length_of_the_main_body_of_the_premaxilla greater_than_the_anteroposterior_length_of_the_main_body_of_the_premaxilla, 35 'Premaxilla, base of the prenarial process' / anteroposteriorly_shallow anteroposteriorly_deep, 36 'Premaxilla, postnarial process' / absent 'short, ends well anterior to the posterior margin of the external naris' 'well-developed, forms most of the border of the external naris or excludes the maxilla from participation in the external naris', 37 'Premaxilla, postnarial process' / 'wide, platelike' thin, 38 'Premaxilla, sharp dorsal flange at the base of the postnarial process delimiting the posteroventral border of the external naris' / absent present, 39 'Premaxilla, postnarial process' / fits_between_the_nasal_and_the_maxilla_or_lies_on_the_anterodorsal_surface_of_the_maxilla overlaps_the_anterodorsal_surface_of_the_nasal fits_into_slot_of_the_nasal_, 40 'Premaxilla, contact with prefrontal' / absent 'present, marginal' 'present, extensive', 41 'Premaxilla, palatal process on the medial surface' / absent present, 42 'Premaxilla, number of tooth positions' / 10_or_more 5_or_more 4 three 2 1_or_edentulous, 43 'Premaxilla, orientation of the tooth series or the occlusal surface of premaxilla in ventral view' / approximately_parasagittal strongly_transverse_and_anterior_teeth_covering_each_other_in_lateral_view, 44 'Premaxilla, lateroventrally opened anterior alveoli in mature individuals' / absent present, 45 Septomaxilla / present absent, 46 'Maxilla-nasal, maxillo-nasal tuberosity, delimiting anteriorly the antorbital fossa if present' / absent present, 47 'Maxilla-jugal, anguli oris crest' / absent present, 48 'Maxilla-jugal, anterior extension of the anguli oris crest' / restricted_to_the_main_body_of_the_jugal 'extending onto the maxilla, but not the anterior process of the jugal', 49 'Maxilla, anterior extent' / posterior_to_the_anterior_extent_of_the_nasals anterior_to_the_nasals, 50 'Maxilla, length of the portion of the bone anterior to the antorbital fenestra versus the total length of the bone' / '0.12-0.22' '0.29-0.60' '0.64-0.76', 51 'Maxilla, posterior border of the subnarial foramen extending posteriorly as a groove on the lateral surface of the anterior process' / absent present, 52 'Maxilla, anterior maxillary foramen' / absent present, 53 'Maxilla, neurovascular foramina on the lateral surface of the anterior and horizontal processes' / laterally_or_lateroventrally_facing 'lateroventrally facing and extending ventrally as deep, well-defined grooves', 54 'Maxilla, antorbital fossa on the lateral surface of the bone' / absent_or_not_exposed_in_lateral_view 'present on the ascending process of the maxilla, but not along the horizontal process of the maxilla' 'present on the horizontal process of the maxilla, but not reaching the posteroventral corner of the fenestra ' 'present on the horizontal process of the maxilla, reaching the posteroventral corner of the opening', 55 'Maxilla, anteroposterior length of the antorbital fossa anterior to the antorbital fenestra versus length of the antorbital fenestra' / '0.09-0.23' '0.28-0.43' '0.90-0.94' '>2.00', 56 'Maxilla, secondary antorbital fossa anteriorly to the antorbital fossa and adjacent to the dorsal margin of the anterior process' / absent present, 57 'Maxilla, ascending process' / absent present, 58 'Maxilla, ascending process shape' / simply_tapers_to_a_point_dorsally 'the dorsal apex of the maxilla is a separate, distinct process that has a posteriorly concave margin' 'sub-vertical anterior margin of the base of the process', 59 'Maxilla, anterodorsal margin at the base of the ascending process' / convex_or_straight concave, 60 'Maxilla, ascending process remains the same width for its length' / absent present, 61 'Maxilla, contact with prefrontal' / absent present, 62 'Maxilla, ventral margin of the antorbital fossa or fenestra (if the antorbital fossa is absent from the horizontal process of the maxilla) in the horizontal process' / 'mainly sub-parallel to the alveolar margin of the bone' 'diagonal, anteroventrally-to-posterodorsally oriented in an angle close to 45º', 63 'Maxilla, shape of the posterior portion of the bone (ventral to the antorbital fenestra if it is present)' / tapers_posteriorly has_a_similar_dorsoventral_depth_as_the_anterior_portion_ventral_to_the_antorbital_fenestra expands_dorsoventrally_towards_the_distal_end_of_the_horizontal_process_with_a_concave_ventral_margin_of_the_antorbital_fenestra expands_dorsoventrally_towards_the_distal_end_of_the_horizontal_process_with_a_straight_ventral_margin_of_the_antorbital_fenestra, 64 'Maxilla, posterior end of the horizontal process distinctly ventrally deflected from the maxin axis of the alveolar margin' / absent present, 65 'Maxilla, triangular dorsal process with clear dorsal apex formed by discrete expansion of the posterior end of the horizontal process in lateral view' / absent present, 66 'Maxilla, palatal process on the anteromedial surface of the bone' / absent present_and_both_counterparts_do_not_meet_at_the_midline_ present_and_both_counterparts_meet_at_the_midline, 67 'Maxilla, position of the palatal process' / adjacent_to_the_base_of_the_interdental_plates distinctly_dorsally_to_the_base_of_the_interdental_plates, 68 'Maxilla, alveolar margin in lateral view' / 'concave, straight or gently convex' distinctly_convex 'sigmoid, anteriorly concave and posteriorly convex ' 'sigmoid, anteriorly convex, starting close to mid-length, and posteriorly concave', 69 'Maxilla, edentulous anterior portion of the ventral margin of the bone' / absent present, 70 'Maxilla, alveolar margin on the anterior third of the bone (anterior to the level of the anterior border of the antorbital fenestra if present)' / approximately_aligned_to_the_posterior_half_of_the_alveolar_margin abruptly_upturned, 71 'Maxilla, posterior extension in mature individuals' / at_level_or_posterior_to_posterior_orbital_border anterior_to_posterior_orbital_border_but_posterior_to_anterior_orbital_border at_level_or_anterior_to_anterior_orbital_border, 72 'Maxilla, tooth plate' / absent present, 73 'Maxilla, number of tooth rows' / single_row multiple_rows, 74 'Maxilla, location of teeth' / only_on_occlusal_surface on_occlusal_and_lingual_surfaces, 75 'Maxilla, number of tooth positions' / '8-9 ' '10-14 ' '15-22' '23-35' '36-40', 76 'Nasal, total length versus total length of the frontal' / '0.68-0.79' '0.92-2.07' '2.26-3.09', 77 'Nasal, exposure (excluding descending process if present)' / largely_dorsal_element nearly_vertical_contribution_to_the_snout, 78 'Nasal, shape of anterior margin at midline' / strongly_convex_with_anterior_process transverse_with_little_convexity, 79 'Nasal, anterior portion in lateral view' / below_or_at_the_same_level_as_skull_roof 'elevated above skull roof, giving the skull a ?Roman nose? appearance', 80 'Nasal, dorsal surface around posterior margin of external naris' / smooth_or_sculpturing_of_ridges_and_grooves_present depression_around_entire_posterior_margin_that_lacks_sculpturing, 81 'Nasal, descending process, which results from the articulation of the postnasal process of the premaxilla on the anterodorsal surface of the nasal and has an extensive contact with the ascending process of the maxilla' / anteroposteriorly_narrow 'anteroposteriorly very broad, being considerably broader than the ascending process of the maxilla', 82 'Nasal, dorsolateral margin of the anterior portion' / smoothly_rounded distinct_longitudinal_ridge_on_the_lateral_edge, 83 'Nasal, participation in the dorsal border of the antorbital fossa' / absent present, 84 'Lacrimal-postorbital, contact between bones' / absent present, 85 'Lacrimal, participation in the posterior border of the external naris' / present absent, 86 'Lacrimal, exposure on the skull roof in dorsal view' / absent_or_marginal present, 87 'Lacrimal, anterior process forming the entire or almost the entire dorsal border of the antorbital fenestra' / absent present, 88 'Lacrimal, antorbital fossa forming a distinct inset margin to the antorbital fenestra on the lateral surface of the bone' / absent 'present, but strongly restricted anteirorly' present_and_occupies_almost_half_or_more_of_the_anteroposterior_length_of_the_ventral_process_, 89 'Lacrimal, naso-lacrimal duct' / completely_enclosed_by_the_lacrimal enclosed_by_the_lacrimal_and_prefrontal, 90 'Lacrimal, naso-lacrimal duct position' / opens_on_the_posterolateral_edge_of_the_lacrimal opens_on_the_posterior_surface_of_the_lacrimal, 91 'Jugal-quadratojugal, ventral margin in lateral view' / straight_or_convex 'concave, though nowhere dorsal to tooth row', 92 'Jugal, anterior process shape in lateral view' / 'continuously tapering or subrectangular, being lower than the portion of the maxilla underneath it' 'subrectangular or slightly dorsoventrally expanded, being higher than the portion of the maxilla underneath it' with_an_ascending_subprocess_excluding_the_lacrimal_from_the_anteroventral_border_of_the_orbit, 93 'Jugal, anterior process continuously dorsally curved' / 'absent, straight or curved only at its proximal half' present, 94 'Jugal, ventral border of the orbit: ' / gently_concave 'V-shaped', 95 'Jugal, anterior extension of the anterior process' / 'anterior to the level of mid-length of the orbit' 'up to or posterior to the level of mid-length of the orbit', 96 'Jugal, participation of the anterior process in the border of the antorbital fenestra' / present 'absent, excluded by contact between the maxilla and lacrimal', 97 'Jugal, longitudinal ridge or bump(s) on the lateral surface of the main body' / absent present, 98 'Jugal, multiple pits on the lateral surface of the main body' / absent present, 99 'Jugal, ascending process forming the entire anterior border of the infratemporal fenestra' / absent 'present, postorbital excluded from the anterior border of the infratemporal fenestra', 100 'Jugal, length of the posterior process versus the height of its base' / '0.49-1.27' '1.59-3.77' '4.07-5.37', 101 Jugal_posterior_process_with_a_distinct_lateroventral_orientation_with_respect_to_the_sagittal_axis_of_the_snout / absent present, 102 'Jugal, distal half of the posterior process' / tapering subrectangular, 103 'Jugal, posterior process forms entirely or almost entirely the ventral border of the infratemporal fenestra (it also applies in the lower temporal bar is incomplete)' / absent present, 104 'Jugal, base of the posterior process with a semi-elliptical, ventral expansion in lateral view' / absent present, 105 'Jugal, posterior process' / lies_dorsal_to_the_anterior_process_of_the_quadratojugal lies_ventral_to_the_anterior_process_of_the_quadratojugal splits_the_anterior_process_of_the_quadratojugal is_splited_by_the_anterior_process_of_the_quadratojugal_, 106 'Jugal, posterior termination of the posterior process' / anterior_to_or_at_level_with_the_posterior_border_of_the_infratemporal_fenestra posterior_to_the_infratemporal_fenestra, 107 'Prefrontal, contact its counterpart in the median line of the skull roof' / absent present, 108 'Prefrontal, suture with the nasal' / 'parasagittal, at least in its posterior third, or anterolateral' anteromedial, 109 'Prefrontal, subtriangular medial process' / 'absent, nasal-frontal suture transversely broad' 'present, nasal-frontal suture strongly transversely reduced', 110 'Prefrontal, groove on the lateral surface of the main body opening into the orbital border' / absent present, 111 'Prefrontal, lateral surface of the orbital margin' / smooth_or_slight_grooves_present rugose_sculpturing_present, 112 'Frontal, frontals fused to one another' / absent present, 113 'Frontal, suture with the nasal' / transverse 'oblique, forming an angle of at least 60° with long axis of the skull and frontals entering between both nasals' 'oblique and nasals entering considerably between frontals in a non-interdigitate suture', 114 'Frontal, orbital border' / absent_or_anteroposteriorly_short anteroposteriorly_long_and_forms_most_of_the_dorsal_edge_of_the_orbit, 115 'Frontal, dorsal surface' / flat_or_slightly_depressed with_longitudinal_ridge_along_midline, 116 'Frontal, suture with parietal' / 'mostly transverse or parietals slightly entering between frontals on the median line, forming an obtuse-angled suture' 'parietals strongly entering between both frontals, forming an acute-angled suture' 'W-shaped suture', 117 'Frontal, participates on the anteromedial corner of the supratemporal fossa' / absent present, 118 'Frontal, dorsal surface adjacent to sutures with the postfrontal (if present) and parietal' / flat_to_slightly_concave possesses_a_longitudinal_and_deep_depression, 119 'Frontal, longitudinal groove' / longitudinally_extended_along_most_of_the_surface_of_the_frontal 'anterolaterally-to-posteromedially extended along the posterior half of the frontal', 120 'Frontal, ventral surface' / 'hourglass-shaped median longitudinal canal for the passage of the olfactory duct and olfactory bulb moulds on the anterior end of the bone' 'median longitudinal canal for the passage of the olfactory duct only slightly constricted, no olfactory bulb moulds and distinct semilunate posteromedially-to-anterolaterally oriented ridge on the orbital roof, extending onto the prefrontal', 121 'Frontal, olfactory tract on the ventral surface of the frontal' / maximum_transverse_constriction_point_well_posterior_to_the_moulds_of_the_olfactory_bulbs_and_posterolateral_margin_of_the_bulbs_delimited_by_a_low_ridge 'maximum transverse constriction of the olfactory bulbs immediately posterior to the moulds of the olfactory bulbs and posterolateral margin of the bulbs well delimited by a thick, tall ridge', 122 Postfrontal / equivalent_in_size_to_postorbital reduced_to_approximately_less_than_half_the_size_of_the_postorbital absent, 123 'Postfrontal, participation in the border of the supratemporal fenestra' / absent present, 124 'Postfrontal, shape of dorsal surface' / flat_or_slightly_concave_towards_raised_orbital_rim depression_with_deep_pits, 125 'Postorbital-jugal, postorbital bar' / composed_by_both_jugal_and_postorbital_in_nearly_equal_proportion composed_mostly_by_the_postorbital, 126 'Postorbital-squamosal, upper temporal bar' / 'located approximately at level of mid-height of the orbit' located_approximately_aligned_to_the_dorsal_border_of_the_orbit, 127 'Postorbital-squamosal, contact' / restricted_to_the_dorsal_margin_of_the_elements 'continues ventrally for much or most of the ventral length of the squamosal, but squamosal does not contact jugal' continues_ventrally_for_much_or_most_of_the_ventral_length_of_the_squamosal_and_squamosal_contacts_jugal, 128 'Postorbital, lateral boss adjacent to orbital margin' / absent present, 129 'Postorbital, supratemporal fossa extending onto the ascending process' / absent present, 130 'Postorbital, posterior process extends close to or beyond the level of the posterior margin of the supratemporal fenestrae' / absent present, 131 'Postorbital, extension of the ventral process' / ends_much_higher_than_the_ventral_border_of_the_orbit ends_close_to_or_at_the_ventral_border_of_the_orbit, 132 'Postorbital, ventral process in lateral view' / continuously_anteriorly_curved_or_straight distinctly_anteriorly_flexed, 133 'Postorbital, depression on the lateral surface of the ventral process' / absent present, 134 'Postorbital, anteriorly projecting, rounded spur on the anterior edge of the ventral process indicating the lower delimitation of the eyeball' / absent present, 135 'Squamosal, completely covering the quadrate in lateral view' / present absent, 136 'Squamosal, overhanging quadrate laterally' / absent present, 137 'Squamosal, anterior process forms more than half of the lateral border of the supratemporal fenestra' / absent present, 138 'Squamosal, anteroventral process' / absent present, 139 'Squamosal, transition between the anterior and ventral processes' / 'sharp, squared posterodorsal border of the infratemporal fenestra' 'gentle, widely rounded posterodorsal border of the infratemporal fenestra', 140 Squamosal_medial_process / 'short, forming approximately half or less of the posterior border of the supratemporal fenestra' 'long, forming entirely or almost entirely the posterior border of the supratemporal fenestra', 141 'Squamosal, posterior process' / does_not_extend_posterior_to_the_head_of_the_quadrate extends_posterior_to_the_head_of_the_quadrate, 142 'Squamosal, posterior process shape' / straight_ ventrally_curved, 143 'Squamosal, ventral process' / present absent, 144 'Squamosal, ventral process shape' / 'anteroposteriorly broad and plate-like' 'anteroposteriorly narrow and strap-like', 145 'Squamosal, ventral process orientation' / 'posteroventrally directed, vertical, or more than 45º from the vertical' anteroventrally_directed_at_45º_or_less, 146 'Squamosal, contribution of the ventral process to the posterior border of the infratemporal fenestra' / forms_less_than_half_of_the_border_of_the_fenestra 'forms more than half of the border, but quadratojugal or quadrate broadly participates in the border of the fenestra' forms_almost_completely_the_border_of_the_fenestra, 147 'Squamosal, posterodorsally-to-anteroventrally oriented tuck on the lateral surface of the ventral process' / absent present, 148 'Squamosal, longitudinal ridge on the lateral surface of the ventral process' / absent present, 149 'Squamosal, posterodorsal portion with a supratemporal fossa' / absent present, 150 Quadratojugal / absent_or_fused_to_the_quadrate present, 151 'Quadratojugal, shape' / 'L-shaped or strip-like bone' subtriangular, 152 'Quadratojugal, infratemporal fossa marked by a sharp edge' / absent present, 153 'Quadratojugal, anterior process' / 'absent, anteroventral margin of the bone rounded' 'incipient, short anterior prong on the anteroventral margin of the bone' 'distinctly present, in which the lower temporal bar is complete, but process terminatesnwell posterior to the base of the posterior process of the jugal' 'distinctly present, in which the lower temporal bar is complete and participates in the posteroventral border of the infratemporal fenestra, and process terminates close to the base of the posterior process of the jugal', 154 'Quadratojugal, widely concave notch on the anterior margin of the ascending process' / absent present, 155 'Quadratojugal, depression along the posterior half of the ascending process up to the exposed lateral surface of its distal tip' / absent present, 156 'Quadratojugal, posterior extension of the ventral end' / 'absent, without a posteriorly arched quadratojugal' 'limited, ventral condyles of the quadrate broadly visible in lateral view ' 'strongly developed, overlapping completely or almost completely the ventral condyles of the quadrate in lateral view ', 157 Supratemporal / broad_element 'slender, in parietal and squamosal trough' absent, 158 'Supratemporal, bifurcated medial border, in which a ventromedial process extends underneath the posterolateral process of the parietal' / present absent, 159 'Parietal, median contact between both parietals' / suture_present fused_with_loss_of_suture, 160 'Parietal, extension over interorbital region' / absent_or_slight present, 161 'Parietal, supratemporal fossa medial to the supratemporal fenestra' / well_exposed_in_dorsal_view_and_mainly_dorsally_or_dorsolaterally_facing poorly_exposed_in_dorsal_view_and_mainly_laterally_facing, 162 'Parietal, pineal fossa on the median line of the dorsal surface' / absent present, 163 'Parietal, position of the pineal fossa' / restricted_to_the_parietal extended_along_frontal_and_parietal, 164 'Parietal, pineal foramen' / large 'reduced to a small, circular pit' absent, 165 'Parietal, position of the pineal foramen in dorsal view' / 'completely enclosed by parietals in the anterior half of the bone (excluding posterolateral processes of the parietals)' 'completely enclosed by parietals close to mid-length or in the posterior half of the bone (excluding posterolateral processes of the parietals)' enclosed_by_both_frontals_and_parietals, 166 'Parietal, distinct transverse emargination adjacent to the posterior margin of the bone in late ontogeny' / absent present, 167 'Parietal, posterolateral process' / nearly_vertical ventrally_inclined_greater_than_45º, 168 'Parietal, posterolateral process height' / 'dorsoventrally low, usually considerably lower than the supraoccipital' 'dorsoventrally very deep, being plate-like in occipital view and subequal to the height of the supraoccipital', 169 'Parietal, posterolateral process with a strongly transversely convex dorsal margin elevated from the median line of the posterior margin of the skull roof' / absent present, 170 'Parietal, tuberosity on the posterior surface of the base of the posterolateral process' / absent present, 171 'Postparietal, size (pair of postparietals if they are not fused to each other)' / 'sheet-like, not much narrower than the suproccipital' 'small, splint-like' absent_as_a_separate_ossification, 172 'Postparietal, fusion between counterparts' / absent 'present, forming an interparietal', 173 Tabular / present absent, 174 'Palpebral/s' / absent present, 175 'Neomorphic bone (= septomaxilla of phytosaurs), separate ossification anterior to nasals and surrounded by the premaxilla on the dorsal surface of the snout' / absent present, 176 'Quadrate, shape' / straight_posteriorly shallowly_emarginated with_conch, 177 'Quadrate, angle between the posterior margins of the dorsal and ventral ends' / '41-47º' '91-96º' '106-137º' '143-158º', 178 'Quadrate, dorsal head' / does_not_have_a_sutural_contact_with_the_paroccipital_process_of_the_opisthotic has_a_sutural_contact_with_the_paroccipital_process_of_the_opisthotic, 179 'Quadrate, dorsal head' / partially_exposed_laterally completely_covered_by_the_squamosal, 180 'Quadrate, dorsal end hooked posteriorly in lateral view' / absent present, 181 'Quadrate, foramen on the medial wall of the quadrate foramen' / absent present, 182 'Quadrate, posterior margin of the ventral half in lateral view' / concave convex, 183 'Quadrate, ventral condyles' / subequally_distally_extended medial_condyle_distinctly_more_distally_projected_than_the_lateral_one, 184 'Neomorph ossification, present between the pterygoid, quadrate and skull roof' / 'absent, the quadrate flange of the pterygoid meets the quadrate but remains free of the skull roof' present, 185 'Vomer, shape' / 'broad, plate-like bone, at least as transversely broad as the internal naris' 'stick-like bone, transversely narrower than the internal naris', 186 'Vomer, contact with maxilla' / absent present, 187 'Vomer, teeth' / 'present, more than one row or no rows are distinguishable ' 'present, mainly in a single row, but multiple teeth present immediately anterior to the contact with the pterygoid' 'present, single row along entire extension ' absent, 188 'Palatine-pterygoid, teeth on the palatine and ventral surface of the anterior ramus of the pterygoid' / present absent, 189 'Palatine-pterygoid, height and dimatre of teeth on the palatine, ventral surface of the anterior ramus of the pterygoid and vomer' / considerably_smaller_than_those_of_the_marginal_dentition_ similar_to_those_of_the_marginal_dentition, 190 'Palatine, transverse extension' / 'narrow, subequal contribution of the palatine and pterygoid to or pterygoid main component of the palate posteriorly to the choanas' 'broad, the palatine is the main component of the palate posteriorly to the choanas', 191 'Palatine, anterior processes forming the posterior border of the choana' / subequal_in_anterior_extension_or_anterolateral_process_longer anteromedial_process_longer single_process, 192 'Pterygoids, contact with each other' / 'present, anteriorly ' 'absent, remain separate along their entire length', 193 'Pterygoid, anterior ramus (= palatal process)' / extends_anterior_to_the_anterior_limit_of_the_palatine forms_oblique_suture_with_palatine_but_process_ends_before_reaching_anterior_limit_of_palatine forms_transverse_suture_with_palatine, 194 'Pterygoid, anterior ramus (= palatal process) shape' / 'transversely broad at its base, converging gradually with the transverse ramus' 'transversely narrow along its entire extension, converging in a right or acute angle with the transverse ramus and the bone acquires an overall L-shape contour in ventral or dorsal view', 195 'Pterygoid, teeth on the ventral surface of the anterior ramus (= palatal process), excluding tiny palatal teeth if present' / 'present in two distinct fields (= T2 and T3 of Welman, 1998)' 'present in three distinct fields (= T2, T3a and T3b)' 'present in three distinct fields (= T2a, T2b and T3)' 'present in one field that occupies most of the transverse width of the ramus (= T2 + T3)' 'present in only one posteromedially-to-anterolaterally oriented field (= T2)' 'present in only one field adjacent to the medial margin of the ramus (= T3)' absent, 196 'Pterygoid, number of rows on palatal tooth field T2' / more_than_two_or_do_not_dispose_on_distinct_rows two_rows_parallel_to_each_other single_row, 197 'Pterygoid, number of rows on palatal tooth field T3' / more_than_two_or_not_disposed_in_distinct_rows two_parallel_rows single_row, 198 'Pterygoid, most lateral row of teeth on the ventral surface of the anterior ramus (tooth field T2) raised on a thick, posteromedially-to-anterolaterally oriented ridge' / absent present, 199 'Pterygoid, a row of fang-like teeth on the medial edge of the anterior ramus (= palatal process) (= T4 of Welman, 1998)' / absent present, 200 'Pterygoid, orientation of the lateral ramus' / 'posterolaterally, forming an obstuse angle with the anterior ramus' 'laterally or anterolaterally, forming a right or acute angle with the anterior ramus', 201 'Pterygoid, lateral margin of the lateral ramus in dorsal or ventral view' / posterolateral_margin_with_an_acute_corner posterolateral_margin_merges_smoothly_into_anterolateral_margin_forming_a_smoothly_convex_lateral_outline, 202 'Pterygoid, teeth on the lateral ramus' / 'present, more than a single row or no rows recognizable' 'present, single row on the posterior edge (= T1 of Welman, 1998)' absent, 203 'Ectopterygoid, body' / arcs_anteriorly arcs_anterodorsally, 204 'Ectopterygoid, articulation with pterygoid' / simple_overlap_of_ectopterygoid_and_pterygoid complex_overlap_between_ectopterygoid_and_pterygoid, 205 'Ectopterygoid, shape along suture with pterygoid' / does_not_reach_the_posterolateral_corner_of_the_transverse_flange reaches_the_posterolateral_corner_of_the_transverse_flange, 206 'Ectopterygoid, contact with maxilla' / absent present, 207 'Ectopterigoid, posterior expansion in contact with jugal' / absent present, 208 'Supraoccipital, shape in occipital view' / 'plate-like' 'inverted V-shape', 209 'Supraoccipital, participation in the dorsal border of the foramen magnum' / absent present, 210 'Supraoccipital, posterior surface' / smooth_or_with_a_low_median_ridge 'with a prominent median, vertical ridge', 211 'Otoccipital, fusion between opisthotic and exoccipital' / absent_or_partial present, 212 'Opisthotic, contact between paroccipital process and parietal immediately lateral to supraoccipital' / absent present, 213 'Opisthotic, paroccipital processes orientation' / extend_laterally_forming_aproximately_a_90°_angle_with_the_parasagittal_plane deflected_posterolaterally_at_an_angle_of_more_than_20°_from_the_transverse_plane_of_the_skull, 214 'Opisthotic, paroccipital process attachment' / ends_freely 'contacts supratemporal or proximal end of quadrate and/or squamosal' sutured_to_the_pterygoid_and_the_pterygoid_wing_of_the_quadrate, 215 'Opisthotic, paroccipital process morphology' / unflattened_and_tapered_ 'anteroposteriorly-flattened distally ', 216 'Opisthotic, fossa immediately lateral to the foramen magnum' / absent present, 217 'Opisthotic, ventral ramus shape' / 'club-shaped ' 'pyramidal, with a tapering distal end' 'rod-like, with a cylindrical distal end and relatively thin' 'rod-like and very robust' 'plate-like', 218 'Opisthotic, ventral ramus' / extends_further_laterally_than_the_lateralmost_edge_of_the_exoccipital_in_posterior_view covered_by_the_lateralmost_edge_of_the_exoccipital_in_posterior_view, 219 'Exoccipital, morphology of the dorsal end' / 'exoccipital columnar throught dorsoventral height, forming transversely narrow dorsal contact with more dorsal occipital elements' dorsal_portion_of_exoccipital_exhibits_dorsomedially_inclined_process_that_forms_transversely_broad_contact_with_more_dorsal_occipital_elements, 220 'Exoccipital, lateral surface' / 'without subvertical crest (= metotic strut)' 'with clear crest (= metotic strut) lying anterior to both external foramina for hypoglossal nerve (CN XII) ' 'with clear crest (= metotic strut) present anterior to the more posterior external foramina for hypoglossal nerve (CN XII)', 221 'Exoccipital, medial margin of their distal ends' / no_contact_with_its_counterpart 'contact with its counterpart to exclude basioccipital from the floor of the endocranial cavity and diverge from each other on the occipital condyle, exposing the basioccipital dorsally' 'contact with its counterpart along the entire dorsal surface of the basioccipital, excluding the basioccipital from the floor of the endocranial cavity and the dorsal surface of the occipital condyle', 222 'Exoccipital, number of foramina for the passage of the hypoglossal nerve (CN XII)' / two one, 223 'Pseudolagenar recess, between the ventral surface of the ventral ramus of the opisthotic and the basal tubera' / present absent, 224 'Lagenar/cochlear recess' / absent_or_short_and_strongly_tapered present_and_elongated_and_tubular, 225 'Basioccipital-parasphenoid/parabasisphenoid, contact with each other in mature individuals' / 'loose, overlapping suture' 'tightly sutured, sometimes by an interdigitated suture, or both bones fused to each other', 226 'Basioccipital-parasphenoid/parabasisphenoid, basal tubera' / absent present, 227 'Basioccipital-parasphenoid/parabasisphenoid, basal tubera shape' / clearly_separated partially_connected medially_expanded_and_nearly_or_completely_connected, 228 'Basioccipital, position of the posterior margin of the occipital condyle' / even_with_craniomandibular_joint_ anterior_to_craniomandibular_joint posterior_to_craniomandibular_joint, 229 'Basioccipital, articular surface of the occipital condyle' / concave hemispherical, 230 'Basioccipital, notochordal scar on the occipital surface of the occipital condyle' / absent_or_developed_as_a_small_subcircular_pit 'developed as a vertical furrow or a large sub-circular fossa that occupies approximately half of the height of the occipital surface of the condyle', 231 'Basioccipital, occipital neck' / 'present, distinctly separating the occipital condyle from the basioccipital body' absent_or_extremely_short, 232 'Basioccipital, shape of the basal tubera' / rounded_and_anteroposteriorly_elongated bladelike_and_anteroposteriorly_shortened, 233 'Basioccipital, orientation of the basal tubera' / 'lateroventral, basal tubera divergent from each other' 'ventral, basal tubera parallel with each other', 234 'Parasphenoid-basisphenoid/parabasisphenoid, exposure on the median line of the endocranial cavity floor' / present absent, 235 'Parasphenoid/parabasisphenoid, orientation' / horizontal_ 'oblique, main axis posterodorsal-to-anteroventrally oriented', 236 'Parasphenoid/parabasisphenoid, posterodorsal portion' / incompletely_ossified completely_ossified, 237 'Parasphenoid/parabasisphenoid, intertuberal plate' / absent present_and_straight present_and_arched_anteriorly, 238 'Parasphenoid/parabasisphenoid, semilunar depression on the posterolateral surface of the bone' / absent present, 239 'Parasphenoid/parabasisphenoid, recess (= median pharyngeal recess, = hemispherical sulcus, = hemispherical fontanelle)' / absent present, 240 'Parasphenoid/parabasisphenoid, position of the foramina for entrance of the cerebral branches of the internal carotid artery leading to the pituitary fossa' / ventral posterolateral anterolateral, 241 'Parasphenoid/parabasisphenoid, position of the foramina for the entrance of the cerebral branches of the internal carotids on the ventral surface of the bone' / immediately_medial_or_posteromedial_to_the_base_of_the_basipterygoid_process close_to_the_suture_between_basioccipital_and_parabasisphenoid, 242 'Parasphenoid/parabasisphenoid, shape of the cultriform process in lateral view' / 'continuously tapering anteriorly, without dorsoventral constriction at its base ' dorsoventrally_compressed_at_its_base_, 243 'Parasphenoid/parabasisphenoid, base of the cultriform process' / relatively_dorsoventrally_short 'tall, with the dorsal edge extending up between clinoid processes and ventral parts of the crista prootica ', 244 'Parasphenoid/parabasisphenoid, dentition on cultriform process' / present absent, 245 'Basisphenoid/parabasisphenoid, anterior tympanic recess on the lateral side of the braincase' / absent present, 246 'Basisphenoid/parabasisphenoid, parasphenoid crests' / absent_so_that_there_is_no_ventral_floor_for_the_vidian_canal present_as_a_pair_of_thick_crests_running_along_the_ventrolateral_border_of_the_basisphenoid_body_and_framing_the_ventromedial_floor_of_the_vidian_canal, 247 'Basisphenoid/parabasisphenoid, basipterygoid processes' / 'moderately short, finger-like and with short articulating facets' 'long, with hemispherical articulating facets' very_short_and_subcylindrical, 248 'Basisphenoid/parabasisphenoid, orientation of basipterygoid processes' / anterolateral_or__lateral posterolateral, 249 'Prootic-supraoccipital, floccular (= auricular) recess' / largely_restricted_to_the_prootic extends_onto_internal_surface_of_the_supraoccipital, 250 'Prootic-basisphenoid/parabasisphenoid, position of the external foramina for passage of the abducens nerves (CN VI)' / within_the_dorsum_sellae 'track between the dorsum sellae and prootic, grooving the articular facets' within_the_prootic, 251 'Prootic-basisphenoid/parabasisphenoid, orientation of the external foramina for passage of the abducens nerves (CN VI)' / open_anteriorly open_dorsally, 252 'Prootic, extensive contact with parietal' / absent present, 253 'Prootic, contact with its counterpart on the median line of the floor of the endocranial cavity' / absent present, 254 'Prootic, lateral surface' / continuous_and_slightly_convex crista_prootica_present, 255 'Prootic, anterior inferior process' / 'absent or developed as a small, peg-like projection' well_developed, 256 'Prootic, ridge on the lateral surface of the inferior anterior process ventral to the trigeminal foramen' / present absent, 257 'Prootic, vestibule on the medial surface' / incompletely_ossified almost_completely_ossified, 258 'Laterosphenoid, ossification' / absent present, 259 'Laterosphenoid, anterodorsal channel' / absent present, 260 'Lower jaw, symphysis' / formed_largely_by_dentary formed_only_by_splenial, 261 'Lower jaw, distinct dorsal process behind the alveolar margin' / 'absent, with a slightly convex dorsal margin behind the alveolar portion' 'present, formed by a dorsally well-developed surangular' 'present, formed by a dorsally well-developed posterodorsal ramus of the dentary and sometimes a dorsally well-developed coronoid bone', 262 'Lower jaw, external mandibular fenestra' / absent present, 263 'Lower jaw, anteroposterior length of the external mandibular fenestra versus anteroposterior length of the dentary anterior to the fenestra' / '0.07-0.36' '0.44-0.53' '0.71-0.88', 264 'Lower jaw, Meckelian fossa orientation' / dorsomedially 'mostly dorsally due to greatly expanded prearticular resulting in a ventral border of the fossa situated dorsal to the half-height of the lower jaw at that level', 265 'Dentary-splenial, mandibular symphysis length' / positioned_distally 'present along one-third of the lower jaw', 266 'Dentary, minimum height of the bone versus length of the alveolar margin (including edentulous anterior end if present)' / '0.05-0.14' '0.16-0.19' '0.22-0.29' '0.34-0.36', 267 'Dentary, shape of the tooth bearing portion' / mostly_straight distinctly_dorsally_curved_during_all_or_most_of_its_extension ventrally_curved_or_deflected, 268 'Dentary, large foramina aligned in two distinct rows starting on the anteroventral corner of the bone' / absent present, 269 'Dentary, longitudinal groove approximately centred dorsoventrally on the lateral surface' / absent present, 270 'Dentary, position of the Meckelian groove on the anterior half of the bone' / dorsoventral_centre_of_the_dentary restricted_to_the_ventral_border, 271 'Dentary, anterior portion' / 'unexpanded, dorsal margins of the anterior and posterior portions of the bone in the same plane' 'dorsally expanded, whole dorsoventral height of the anterior portion is greater than that of the posterior portion', 272 'Dentary, posterodorsal process, in which its dorsal margin is confluent with the dorsal margin of the lower jaw' / absent present, 273 'Dentary, posterocentral process, in which its margins are not confluent with the dorsal or ventral margin of the lower jaw' / absent present, 274 'Dentary, distal end of the posterocentral process (process that contributes to the anterodorsal border of the external mandibular fenestra)' / tapering rounded, 275 'Dentary, posteroventral process, in which its ventral margin is confluent with the ventral margin of the lower jaw' / absent present_and_excluded_from_the_anteroventral_border_of_the_external_mandibular_fenestra present_and_contributing_to_the_anteroventral_border_of_the_external_mandibular_fenestra_, 276 'Dentary, posteroventral process length' / 'extended posteriorly to the level of the posterodorsal and/or posterocentral processes ' 'extended posteriorly beyond the level of the posterodorsal and/or posterocentral processes', 277 Posteriormost_dentary_teeth / on_the_anterior_half_of_lower_jaw on_the_posterior_half_of_lower_jaw, 278 'Dentary, alveolar margin' / present_along_entire_length_of_the_dentary absent_in_the_anterior_portion, 279 'Dentary, number of tooth rows' / one_ two more_than_two_, 280 'Dentary, occlusion with upper teeth' / 'single-sided overlap' flat_occlusion blade_and_groove, 281 'Surangular-angular, suture' / even_with_lateral_surface_of_hemimandible elevated_and_separates_dorsal_concave_area_on_surangular_from_concave_area_on_angular, 282 'Surangular-angular, suture along the anterior half of the bones in lateral view' / anteroposteriorly_convex_ventrally anteroposteriorly_concave_ventrally, 283 'Surangular/articular, retroarticular process' / absent 'anteroposteriorly short, being poorly developed posteriorly to the glenoid fossa' 'anteroposteriorly long, extending considerably posteriorly to the glenoid fossa', 284 'Surangular-articular, retroarticular process' / not_upturned upturned, 285 'Surangular, anterior extension' / beyond_coronoid_eminence posterior_to_reaching_the_anterior_border_of_the_coronoid_eminence, 286 'Surangular, lateral shelf' / absent 'present, low ridge near dorsal margin' 'present, presence of laterally or ventrolaterally projecting shelf with straight or gently convex lateral edge' 'present, presence of laterally projecting shelf with strongly convex lateral edge', 287 'Surangular, dorsal margin in lateral view' / straight_or_gently_convex strongly_convex, 288 'Surangular, anterior surangular foramen on the lateral surface of the bone, near surangular-dentary contact' / absent present, 289 'Surangular, posterior surangular foramen on the lateral surface of the bone, positioned directly anterolateral to the glenoid fossa' / absent present, 290 'Angular, dorsoventral exposure on the lateral surface of the lower jaw' / wide narrow, 291 'Angular, ventrolateral surface' / continuous_with_lateral_surface_of_angular laterally_projecting_ridge_present_that_separates_lateral_and_ventral_sides_of_the_angular, 292 'Angular, posteroventral surface' / ridged_or_keeled transversely_convex, 293 'Articular, fused to the prearticular' / absent present, 294 'Articular, foramen on the medial side' / absent present, 295 'Articular, ventromedially directed process' / absent present, 296 'Stapes, shape' / 'robust, with thick shaft' 'slender, rod-like shaft', 297 'Stapes, stapedial foramen piercing the columellar process' / present absent, 298 'Teeth, posterior extent of mandibular and maxillary tooth rows' / subequal maxillary_teeth_extending_further_posteriorly, 299 'Teeth, tooth attachment' / 'subthecodont (= protothecodont)' ankylothecodont pleurodont acrodont thecodont, 300 'Teeth, maxillary and/or dentary tooth crowns' / generally_homodont 'markedly heterodont (gross change in morphology) ', 301 'Teeth, enlarged caniniform region in maxilla' / present absent, 302 'Teeth, maxillary tooth crowns in labial view' / all_the_tooth_crowns_possess_a_rather_similar_distal_edge_morphology_along_the_entire_alveolar_margin 'the distal edge of the posterior tooth crowns possess a distinct different morphology from those of the anterior tooth crowns, usually the posterior edge becomes convex', 303 'Teeth, distal edge of the maxillary tooth crowns in labial view' / concave_in_all_tooth_crowns straight_or_gently_sigmoid convex_in_at_least_some_anterior_tooth_crowns, 304 'Teeth, serrations on the maxillary/dentary crowns' / absent 'distinctly present on the distal margin and usually apically restricted, low or absent on the mesial margin' present_and_distinct_on_both_margins_, 305 'Teeth, labiolingual compression of the marginal dentition' / only_distally_or_nowhere_ present, 306 'Teeth, multiple maxillary or dentary tooth crowns with longitudinal labial or lingual striations or grooves' / absent present, 307 'Teeth, multiple maxillary and dentary tooth crowns with extensive wear facets' / absent present, 308 'Teeth, multiple maxillary and dentary tooth crowns distinctly mesiodistally expanded above the root' / absent present, 309 'Hyoid apparatus, length and orientation of the ceratobranchial' / 'short, directed to quadrate region' 'long, directed posteriorly and extending posteriorly beyond the quadrate condyles', 310 'Cervical, dorsal, sacral and caudal vertebrae, notochordal canal piercing completely the centrum' / present_throughout_ontogeny absent_in_adults, 311 'Cervical and dorsal vertebrae, anteroposterior compression of centra in the cervico-dorsal transition (= pectoral centra)' / moderate 'very strong, being considerably anteroposteriorly shorter than tall', 312 'Cervical and dorsal vertebrae, neurocentral sutures' / close_in_adults 'remain open in sub-adults and adults', 313 'Cervical and dorsal vertebrae, at least one or more cervical or anterior dorsal with parallelogram-shaped centra in lateral view, in which the anterior articular surface is situated higher than the posterior one' / absent present, 314 'Cervical and dorsal vertebrae, one or more vertebrae with an accessory rib articular facet between the diapophysis and parapophysis in the cervico-dorsal transition' / absent present, 315 'Cervical and dorsal vertebrae, anterior centrodiapophyseal lamina or paradiapophyseal lamina in posterior cervicals and anterior dorsals' / absent present, 316 'Cervical and dorsal vertebrae, posterior centrodiapophyseal lamina in cervicals and anterior dorsals' / absent present, 317 'Cervical and dorsal vertebrae, prezygodiapophyseal lamina in posterior cervicals and anterior dorsals' / absent present, 318 'Cervical and dorsal vertebrae, postzygodiapophyseal lamina in posterior cervicals and anterior dorsals' / absent present, 319 'Cervical and dorsal vertebrae, thick, mainly vertical tuberosity immediately below the transverse process, but both structures are not connected with each other, in posterior cervicals and anterior dorsals' / absent present, 320 'Cervical and dorsal vertebrae, gradual transverse expansion of the distal half of the neural spine' / absent 'present, but lacking distinct mammillary processes on the lateral surface of the neural spine' 'present, with distinct mammillary processes on the lateral surface of the neural spine', 321 'Cervical and dorsal vertebrae, spine table in the distal end of the postaxial neural spines (not mammillary process)' / absent_ 'present in cervicals, but not in dorsals' 'present in dorsal, but not in cervicals' present_in_both_cervicals_and_dorsals, 322 'Cervical and dorsal vertebrae, distal surface of transverse expansion of the neural spine' / convex_ flat_, 323 'Cervical and dorsal vertebrae, outline of the spine tables in dorsal view' / suboval_or_subrectangular 'subtriangular or heart-shaped', 324 'Cervical vertebrae, number of vertebrae in the neck' / fewer_than_eight eight_or_nine more_than_ten, 325 'Cervical vertebrae, atlantal articulation facet on the axial intercentrum' / 'saddle-shaped' concave_with_upturned_lateral_borders, 326 'Cervical vertebrae, centrum of atlas in mature individuals' / separate_from_axial_intercentrum fused_to_axial_intercentrum, 327 'Cervical vertebrae, ventral surface of the centrum on anterior cervicals' / transversely_convex with_a_low_median_longitudinal_keel with_a_median_longitudinal_keel_that_extends_ventral_to_the_centrum_rims_in_at_least_one_anterior_cervical_, 328 'Cervical vertebrae, height of the neural spine of the axis' / dorsoventrally_tall strongly_dorsoventrally_low, 329 'Cervical vertebrae, shape of the neural spine of the axis' / expanded_posterodorsally_or_the_height_of_the_anterior_portion_is_equivalent_to_the_posterior_height expanded_anterodorsally, 330 'Cervical vertebrae, dorsal margin of the neural spine of the axis' / dorsally_convex mostly_straight_or_dorsally_concave, 331 'Cervical vertebrae, lengths of the fourth and fifth cervical centra versus the height of their anterior articular surface' / '0.63-2.67' '2.92-4.12' '6.09-6.80' '14.16-14.33', 332 'Cervical vertebrae, diapophysis and parapophysis of anterior to middle cervical postaxial vertebrae' / single_facet_or_both_situated_on_the_same_process situated_on_different_processes_and_well_separated situated_on_different_processes_and_nearly_touching, 333 'Cervical vertebrae, position of diapophysis or dorsal margin of synapophysis in anterior postaxial cervicals' / at_or_near_dorsoventral_level_of_pedicles 'near the dorsoventral mid-point of the centrum', 334 'Cervical vertebrae, longitudinal lamina or tuberosity extended posteriorly from the base of the transverse process in postaxial anterior and middle cervicals' / 'absent or poorly developed, not well laterally developed' 'strongly developed, flaring laterally as a prominent and thick, wing-like shelf', 335 'Cervical vertebrae, posterior portion of the neural arch ventral to the postzygapophysis in postaxial cervicals' / smooth 'with a shallow, posterolaterally facing fossa', 336 'Cervical vertebrae, epipophysis in postaxial cervicals' / absent present_in_at_least_the_third_to_fifth_cervical_vertebrae, 337 'Cervical vertebrae, excavation immediately lateral to the base of postaxial cervical neural spines' / absent_ shallow_ represented_by_a_deep_pocket_or_pit, 338 'Cervical vertebrae, anterior cervical vertebrae (presacral vertebrae 3?5) postzygapophyses' / separated_posteriorly 'connected through a horizontal lamina (= transpostzygapophyseal lamina) with a notch at the midline', 339 'Cervical vertebrae, shape of the postaxial neural spines in lateral view' / 'sub-triangular' rectangular, 340 'Cervical vertebrae, distinct longitudinal lamina extending along the lateral surface of the centrum at mid-height in postaxial anterior and middle cervical vertebrae' / absent present, 341 'Cervical vertebrae, longitudinal lamina connecting the prezygapophysis and postzygapophysis in the third cervical neural arch' / absent present, 342 'Cervical vertebrae, shape of postaxial anterior cervical neural spines' / 'tall, with height and length approximately equal or height larger' 'long and low, with height lower than length', 343 'Cervical vertebrae, anterior and middle postaxial cervical neural spines with an anterior overhang' / absent present, 344 'Cervical vertebrae, relative location of dorsal margin of mid-cervical neural spines' / spines_are_equivalent_in_height_and_length_to_other_cervical_neural_spines_ 'spines are dorsoventrally depressed at their anteroposterior midpoints, leaving them little more than midline dorsal ridges', 345 'Cervical vertebrae, position of the mammillary processes of the neural spines along the neck' / present_from_the_fourth_presacral present_from_the_fifth_presacral present_from_the_sixth_or_seventh_presacral present_from_the_eighth_or_ninth_presacral, 346 'Cervical vertebrae, postaxial cervical intercentra' / present absent, 347 'Cervical and dorsal ribs, tuberculum in posterior cervical or anterior dorsal ribs' / short long_and_distinct, 348 'Cervical and dorsal ribs, at least one rib of the cervico-dorsal transition with a thin lamina webbing tuberculum and capitulum' / absent present, 349 'Cervical ribs, shape' / 'short, being less than two times the length of its respective vertebra, and tapering at a high angle to the neck ' 'short, being less than two times the length of its respective vertebra, and shaft parallel to the neck ' 'very long, being two times the length of its respective vertebra, and parallel to the neck', 350 'Cervical ribs, accessory process on anterolateral surface of anterior cervical ribs' / absent present, 351 'Dorsal vertebrae, length versus height of the centrum in anterior dorsals' / '0.45-1.10' '1.18-2.00' '2.19-2.74', 352 'Dorsal vertebrae, length versus height of the centrum in posterior dorsals' / '0.66-1.39' '1.48-1.86' '1.95-2.04' '2.39-2.46', 353 'Dorsal vertebrae, ventral surface of middle and posterior centra' / transversely_convex 'ridged, with slightly swollen sides' single_keel double_keel, 354 'Dorsal vertebrae, lateral fossa on the centrum below the neurocentral suture' / absent_ 'present, but not well-rimed' 'present and well-rimed', 355 'Dorsal vertebrae, subcentral foramen in the lateral surface of the centra' / absent present, 356 'Dorsal vertebrae, diapophysis and parapophysis in anterior dorsals' / close_to_the_body_of_the_midline expanded_on_stalks, 357 'Dorsal vertebrae, ratio between transverse width of diapophysis and length of the centrum in anterior dorsals' / '<0.70' '>0.75', 358 'Dorsal vertebrae, development of the transverse processes in middle and posterior dorsals' / short moderately_long 'extremely long, being considerably broader than its respective centrum', 359 'Dorsal vertebrae, hyposphene-hypantrum accessory intervertebral articulation in middle-posterior dorsals' / absent present, 360 'Dorsal vertebrae, zygosphene-zygantrum articulation' / absent present, 361 'Dorsal vertebrae, dorsally opened pit lateral to the base of the neural spine' / absent shallow_ developed_as_a_deep_pit_, 362 'Dorsal vertebrae, anterior and middle dorsal neural spines' / 'subrectangular, with the anterior margin vertical, anterodorsally or slightly posterodorsally inclined' 'subtriangular, with the anterior margin strongly posterodorsally oriented', 363 'Dorsal vertebrae, fan-shaped neural spine in lateral view' / absent present, 364 'Dorsal vertebrae, position of middle dorsal neural spines' / 'situated at mid-length between the zygapophyses' 'displaced posteriorly from mid-length between the zygapophyses', 365 'Dorsal vertebrae, position of the mammillary processes of the neural spines in the trunk' / extend_up_to_the_tenth_presacral extend_up_to_the_eleventh_presacral extend_up_to_the_twelfth_presacral extend_up_to_the_thirteenth_presacral extend_up_to_the_sixteenth_presacral_or_beyond, 366 'Dorsal vertebrae, intercentra' / present absent, 367 'Dorsal ribs, angle between heads and shaft in anterior dorsal ribs' / close_to_90º 'low, gentle posteroventral bowing of the base of the shaft', 368 'Dorsal ribs, proximal end of middle dorsal ribs' / dichocephalous holocephalous, 369 'Sacral vertebrae-sacral ribs, ratio between the width of the neural arch + ribs of the first primordial sacral and the length of the neural arch across the zygapophyses' / less_than_three_times three_times_or_more, 370 'Sacral vertebrae, number' / two three four_or_more, 371 Sacral_ribs / almost_entirely_restricted_to_a_single_sacral_vertebra shared_between_two_sacral_vertebrae, 372 'Sacral ribs, anteroposterior length of the first primordial sacral rib versus the second primordial sacral rib in dorsal view' / primordial_sacral_rib_one_is_longer_anteroposteriorly_than_primordial_sacral_rib_two primordial_sacral_rib_two_is_about_the_same_length_or_longer_anteroposteriorly_than_primordial_sacral_rib_one, 373 'Sacral ribs, second rib shape' / single_unit bifurcates_distally_into_anterior_and_posterior_processes, 374 'Sacral ribs, morphology of posterior process' / pointed_bluntly truncated_sharply, 375 'Sacral and caudal vertebrae, transverse processes and ribs of sacral and/or anterior caudal vertebrae in mature individuals' / sutured_to_the_vertebra fused_to_the_vertebra, 376 'Caudal vertebrae, autotomic septa within the centrum' / absent present, 377 'Caudal vertebrae, length of the transverse process + rib versus length across zygapophyses in anterior caudal vertebrae' / '0.29-0.41' '0.62-1.20' '1.51-1.68' '2.20-2.72', 378 'Caudal vertebrae, distal end of the transverse processes + ribs of anterior caudals in dorsal or ventral view' / tapering_or_squared anteroposteriorly_expanded, 379 'Caudal vertebrae, neural spine height versus anteroposterior length at its base in anterior caudals' / '0.66-2.21' '2.36-2.65' '2.92-3.05' '3.42-3.54', 380 'Caudal vertebrae, accessory laminar process on the anterior face of the neural spine on middle caudals' / absent present, 381 'Caudal vertebrae, prezygapophysis of posterior caudals' / not_elongated_ elongated_more_than_a_quarter_of_the_adjacent_centrum, 382 'Chevrons, distal anteroposterior width of anterior and middle haemal spines in lateral view' / equivalent_to_proximal_length tapering_distally_ 'longer than proximal width (= paddle-like haemal spine)', 383 Gastralia / 'present, forming an extensive ventral basket with closely packed elements' 'present, well separated' absent, 384 'Scapulacoracoid, both bones fused with each other in mature individuals' / present absent, 385 'Scapulacoracoid, notch on the anterior margin at level of the suture between both bones' / absent present, 386 'Scapulacoracoid, glenoid fossa orientation' / posterolateral_ posteroventral_, 387 'Scapula, total length of the scapula versus minimum anteroposterior width of the scapular blade' / '1.23-6.73' '7.92-11.31', 388 'Scapula, large fenestra between scapula and coracoid immediately anterior to the glenoid region' / absent present, 389 'Scapula, strong curvature or inflexion of the posterior margin of the scapular blade' / absent 'present, the angle formed is close to 90º', 390 'Scapula, anterior margin of the scapular blade in lateral view' / straight_or_convex_along_entire_length distinctly_concave, 391 'Scapula, constriction distal to the glenoid' / anteroposterior_length_greater_than_half_the_proximodistal_length_of_the_scapula anteroposterior_length_less_than_half_the_proximodistal_length_of_the_scapula, 392 'Scapula, supraglenoid foramen' / absent present, 393 'Scapula, lateral tuber on the posterior edge, just dorsal of the glenoid fossa' / absent present, 394 'Scapula, diagonal ridge adjacent to the anterior margin on the medial surface of the scapular blade' / abent present, 395 'Scapula, acromion process' / in_about_the_same_plane_as_the_ventral_edge_of_the_scapula distinctly_raised_above_the_ventral_edge_of_the_scapula, 396 'Scapula, acromion process' / gently_raised_from_the_anterior_margin_of_the_scapular_blade sharply_raised_in_an_angle_close_to_90º_from_the_anterior_margin_of_the_scapular_blade, 397 'Coracoid, anterior border in lateral view' / rounded distinctly_hooked, 398 'Coracoid, posterior border in lateral view' / unexpanded_posteriorly moderately_expanded_posteriorly 'strongly expanded posteriorly - the entire border, not only the posteroventral region as is the case in the postglenoid process - and, as a result, the scapular girdle acquires an L-shape in lateral view', 399 'Coracoid, subglenoid lip posteror extension' / as_developed_as_or_less_developed_than_the_supraglenoid_lip_on_the_scapula more_extended_than_the_supraglenoid_lip_on_the_scapula, 400 'Coracoid, subglenoid lip lateral extension' / 'poorly developed, resembling the development of the supraglenoid lip on the scapula' 'strongly developed as a shelf-like structure, more developed than the supraglenoid lip on the scapula', 401 'Coracoid, biceps process' / absent_or_small large, 402 'Coracoid, postglenoid process separated from the glenoid fossa by a notch' / absent present, 403 'Coracoid, postglenoid process shape in lateral view' / rounded_posterior_margin tapering_posterior_margin, 404 Cleithrum / present absent, 405 Interclavicle / present absent, 406 'Interclavicle, anterior process' / present absent, 407 'Interclavicle, anterior margin with a median notch' / absent present, 408 'Interclavicle, lateral processes' / well_developed reduced_or_absent, 409 'Interclavicle, webbed between lateral and posterior processes' / 'present, proximal half of the bone subtriangular or diamond-shaped' 'absent, rather sharp angles between processes', 410 'Interclavicle, transverse width at mid-length of the posterior process versus the length of the posterior process' / '0.07-0.14' '0.20-0.27', 411 'Interclavicle, posterior ramus' / little_change_in_width_along_entire_length gradual_transverse_expansion_present, 412 'Clavicle, articulation with interclavicle' / on_the_anteroventral_surface_of_the_interclavicle on_the_anterodorsal_surface_of_the_interclavicle_ 'into a deep, anteriorly facing socket', 413 Sternum / not_mineralized 'mineralized (bone or calcified cartilage)', 414 'Forelimb-hindlimb, length ratio' / '>0.55' '<0.55', 415 'Humerus, torsion between proximal and distal ends' / approximately_45°_or_more_ 35°_or_less_, 416 'Humerus, transverse width of the proximal end versus total length of the bone in mature individuals' / '0.20-0.41' '0.44-0.70', 417 'Humerus, proximal articular surface in proximal view' / 'subrectangular to crescent-shape' 'sub-oval', 418 'Humerus, proximal articular surface' / continuous_with_the_deltopectoral_crest separated_by_a_gap_from_the_deltopectoral_crest, 419 'Humerus, proximal end in anterior view' / approximately_symmetric 'medially expanded, being asymmetric', 420 'Humerus, conical process on the proximal surface, placed immediately adjacent to the base of the deltopectoral crest' / absent present, 421 'Humerus, internal tuberosity distinctly separated proximally from the articular surface' / absent present, 422 'Humerus, shape of the deltopectoral crest in lateral view' / rounded_or_subtriangular_ subrectangular_or_trapezoidal_, 423 'Humerus, ventral margin of the deltopectoral crest developed as a thick subcilindrical tuberosity that is well differentiated from the thinner dorsal margin' / present absent, 424 'Humerus, length of the deltopectoral crest relative to total length of the bone in mature individuals' / '0.16-0.18' '0.24-0.49' '0.52-0.55', 425 'Humerus, entepicondyle size in mature individuals' / moderately_large strongly_developed, 426 'Humerus, entepicondylar foramen' / present absent, 427 'Humerus, ectepicondylar region' / foramen_present 'foramen absent, supinator process and groove present' 'supinator process, groove or foramen absent', 428 'Humerus, capitellum (radial condyle) and trochlea (ulnar condyle)' / 'ball-shaped structures distinct from the ectepicondyle and entepicondyle' absent_or_incipient, 429 'Humerus, trochlea (ulnar condyle) situated approximately at mid-width on the distal end of the bone' / present 'absent, being considerably laterally displaced ', 430 'Ulna, olecranon process' / absent_or_low prominent_but_lower_than_its_anteroposterior_depth_at_base 'strongly developed, being higher than its anteroposterior depth at base', 431 'Ulna, olecranon process in lateral view' / tapering_towards_the_proximal_tip_of_the_bone subrectangular_or_slightly_expanded_towards_the_proximal_tip_of_the_bone, 432 'Ulna, olecranon process as a separate ossification' / absent present, 433 'Ulna, lateral tuber (= radius tuber) on the proximal portion' / absent present, 434 'Ulna, distal end in posterolateral view' / rounded_and_convex_ squared_off_where_the_distal_surface_is_nearly_flat, 435 'Radius, total length versus total length of the humerus' / '0.62-0.66' '0.69-0.92' '0.95-0.97' '1.12-1.17', 436 'Radius, length in comparisons with that of the ulna' / shorter_ longer_or_subequal, 437 'Radius, shape' / straight twisted_in_lateral_view, 438 'Radius, distal end' / unexpanded_or_poorly_anteroposteriorly_expanded strongly_anteroposteriorly_expanded, 439 'Carpals, intermedium' / present absent, 440 'Carpals, perforating foramen between intermedium and ulnare' / present absent, 441 'Carpals, medial centrale' / present absent, 442 'Carpals, lateral centrale' / large small_or_absent, 443 'Carpals, pisiform' / present absent, 444 'Carpals, distal carpal five' / absent present, 445 'Manus, longest metacarpal + digit' / longer_than_humeral_length_ subequal_to_shorter_than_humeral_length_, 446 'Metacarpus, length of the longest metacarpal versus length of the longest metatarsal' / '0.34-0.39' '0.43-0.45' '0.54-0.98', 447 'Metacarpus, proximal ends' / overlap_ abut_one_another_without_overlapping, 448 'Metacarpus, width of the distal end of the metacarpal I versus its total length' / '0.26-0.33' '0.36-0.45' '0.48-0.53' '0.58-0.64' '0.73-0.75', 449 'Metacarpus, extensor pits on the dorsodistal portion of the metacarpals I-III' / absent_or_shallow_and_symmetrical deep_and_asymmetrical, 450 'Metacarpus, metacarpal IV' / longer_than_metacarpal_III equal_or_shorter_than_metacarpal_III, 451 'Manual digits, unguals length' / about_the_same_length_or_shorter_than_the_last_phalanx_of_the_same_digit distinctly_longer_than_the_last_phalanx_of_the_same_digit, 452 'Manual digits, unguals of manual digits I-III' / blunt_on_at_least_digits_II_and_III 'trenchant on digits I-III', 453 'Manual digits, second phalanx of manual digit II' / shorter_than_the_first_phalanx_of_manual_digit_II_ longer_than_the_first_phalanx_of_manual_digit_II, 454 'Manual digits, number of phalanges in digit IV' / five four three_or_less, 455 'Pelvic girdle, acetabulum' / completely_closed perforated, 456 'Pelvic girdle, acetabulum orientation' / mainly_laterally_facing lateroventrally_or_mainly_ventrally_facing, 457 'Pelvic girdle, acetabular antitrochanter' / absent present, 458 'Ilium, maximum height of the acetabulum versus length of the femur' / '0.12-0.17' '0.21-0.47' '0.54-0.57', 459 'Ilium, laterally deflected dorsal blade' / absent present, 460 'Ilium, preacetabular process' / absent_or_incipient 'present, being considerably anteroposteriorly shorter than its dorsoventral height' 'present, being longer than two thirds of its height and not extending beyond the level of the anterior margin of the pubic peduncle' present_and_extending_beyond_the_level_of_the_anterior_margin_of_the_pubic_peduncle, 461 'Ilium, preacetabular process' / semicircular_ 'subtriangular or finger-like', 462 'Ilium, lateral crest dorsal to the supraacetabular crest/rim' / absent present_and_divides_the_preacetabular_process_from_the_postacetabular_process confluent_with_the_anterior_extent_of_the_preacetabular_process, 463 'Ilium, length of the postacetabular process versus anteroposterior length of the acetabulum' / '0.31-0.63' '0.79-1.24' '1.31-1.37' '1.49-1.55', 464 'Ilium, main axis of the postacetabular process in lateral or medial view' / posterodorsally_oriented mainly_posteriorly_oriented, 465 'Ilium, caudifemoralis brevis muscle origin on the lateroventral surface of the postacetabular process' / not_dorsally_or_laterally_rimed_by_a_brevis_shelf 'dorsally rimed by a brevis shelf, but lacking a brevis fossa' dorsolaterally_rimed_by_a_brevis_shelf_and_with_a_lateroventrally_facing_brevis_fossa laterally_rimed_by_a_brevis_shelf_and_with_a_ventrally_facing_brevis_fossa, 466 'Ilium, dorsal margin of the iliac blade' / convex mostly_straight concave, 467 'Ilium, angle between anterior margin of the pubic peduncle and longitudinal axis across pubic and ischiadic peduncles' / lower_than_45º equal_or_higher_than_45º_, 468 'Ilium, posteriorly projected heel on the posterior margin of the ischiadic peduncle in lateral view' / absent 'present, with its dorsal margin settled at 45º or lower to the longitudinal axis of the bone', 469 'Ilium, acetabulum shape' / 'irregular, marked by posterodorsal invasion by finished bone ' 'roughly circular, no posterodorsal invasion by finished bone', 470 'Pubis-ischium, contact' / present_and_extended_ventrally present_and_reduced_to_a_thin_proximal_contact, 471 'Pubis-ischium, thyroid fenestra:' / absent present, 472 'Pubis, total length versus anteroposterior length of the acetabulum' / '1.15-2.58' '2.84-3.43' '3.94-4.87', 473 'Pubis, anterior and posterior portions of the acetabular margin' / continuous_ recessed_, 474 'Pubis, tuberosity for the attachment of the ambiens muscle in mature individuals' / prominent incipient_or_absent, 475 'Pubis, shaft orientation' / anteroventral vertical_or_posteroventral_, 476 'Pubis, form of the shaft (= pubic tubercle, = pectineal tuberosity)' / 'plate-like' 'rod-like and curved posteriorly' 'rod-like and straight', 477 'Pubis, anterior apron' / 'absent, symphysis extended along the ventral margin of the pelvic girdle and visible in lateral view' 'present, symphysis restricted anteriorly and obscured by the pubic shaft in lateral view', 478 'Pubis, transverse width of conjoined aprons versus total length of the bone' / '0.27-0.59' '0.77-0.97' '1.12-1.28' '1.48-1.94', 479 'Pubis, pectineal process' / absent present, 480 'Pubis, distal end in lateral or medial view' / unexpanded_or_gently_expanded_anteroposteriorly 'sharply expanded anteroposteriorly, forming a distinct pubic boot', 481 'Pubis, transverse width of the distal portion' / nearly_as_broad_as_the_proximal_width significantly_narrower_than_the_proximal_width, 482 'Ischium, total length versus anteroposterior length of the acetabulum' / '1.04-1.24' '1.55-2.50' '2.72-3.53' '4.31-4.48', 483 'Ischium, proximal articular surface' / articular_surface_with_the_ilium_and_pubis_continuous articular_surfaces_with_the_ilium_and_pubis_continuous_but_separated_by_a_fossa articular_surfaces_with_the_ilium_and_pubis_separated_by_a_nonarticulating_concave_surface, 484 'Ischium, longitudinal groove on the dorsal surface of shaft' / absent present, 485 'Ischium, medial contact with antimere' / restricted_to_the_medial_edge extensive_contact_but_the_dorsal_margins_are_separated, 486 'Ischium, symphysis raised on a distinct low peduncle' / absent present, 487 'Ischium, cross-section of the distal portion' / platelike semicircular_or_subtriangular, 488 'Ischium, shape of posterior margin' / linear_posterior_margin posterior_process_extends_from_posterodorsal_ischiadic_margin, 489 'Femur, total length versus total length of the humerus' / '0.92-0.97' '1.09-1.56' '1.62-1.74' '1.86-1.96', 490 'Femur, minimum transverse width versus minimum transverse width of the humerus' / '0.95-1.01' '1.08-1.32' '1.46-1.80' '1.93-2.00', 491 'Femur, proximal articular surface' / 'well ossified, being flat or convex' 'partially ossified, being concave and sometimes with a circular pit', 492 'Femur, femoral head' / not_distinctly_offset_from_the_shaft distinctly_offset_from_the_shaft, 493 'Femur, femoral head orientation (long axis of the femoral head angle with respect to the transverse axis through the femoral condyles Parrish, 1986)' / 'anterior (60º-90º)' 'anteromedial (20º-60º)', 494 'Femur, proximal articular surface (= posterolateral portion of the head sensu Nesbitt 2011)' / limited_to_the_proximal_surface_of_the_bone extends_under_the_proximal_surface_of_the_bone, 495 'Femur, proximal surface' / rounded_and_smooth_ transverse_groove_present, 496 'Femur, posteromedial tuber (= anteromedial tuber of Nesbitt, 2011) on the femoral head' / absent present, 497 'Femur, posterior tuber on the femoral head' / present absent, 498 'Femur, anterior tuber (= anterolateral tuber of Nesbitt, 2011) on the femoral head' / present_as_an_expansion absent_, 499 'Femur, fossa trochanterica (sensu Novas, 1996) on the ventral/posterior surface of the proximal end' / present absent, 500 'Femur, dorsolateral trochanter on the anterolateral surface of the proximal end' / absent present, 501 'Femur, transition between femoral head and shaft' / smooth_ notch_ concave_emargination, 502 'Femur, anterior trochanter (= lesser or minor trochanter) (= iliofemoralis cranialis muscle insertion)' / absent present, 503 'Femur, trochanteric shelf' / absent present_in_mature_individuals, 504 'Femur, attachment of muscle caudifemoralis on the posterior surface of the bone' / 'crest-like and with intertrochanteric fossa (= internal trochanter), and convergent with proximal end' 'crest-like and with intertrochanteric fossa (= internal trochanter), and not convergent with proximal end' 'crest-like and without intertrochanteric fossa (= fourth trochanter), and not convergent with proximal end', 505 'Femur, shape of the process for the attachment of the caudifemoralis musculature' / 'mound-like and rounded' sharp_flange, 506 'Femur, process for the attachment of the caudifemoralis musculature in medial or lateral view' / 'symmetrical, with the proximal and distal margins forming similar low-angle slopes to the shaft' 'asymmetrical, with the distal margin forming a steeper angle to the shaft', 507 'Femur, proximodistal extension of the process for the attachment of the caudifemoralis musculature' / restricted_to_the_proximal_half_of_the_shaft_and_low 'distally extended beyond mid-shaft and well posteriorly developed', 508 'Femur, bone wall thickness at or near midshaft' / 'thickness/diameter >0.3' 'thin, thickness/diameter <0.3 (1)', 509 'Femur, shaft' / diameter_constant_or_widening_distally diameter_distally_narrowed, 510 'Femur, distal transverse width versus total length' / '0.08-0.11' '0.13-0.24' '0.26-0.36' '0.39-0.41', 511 'Femur, distal condyles' / 'prominent, strong dorsoventral expansion (in sprawling orientation) restricted to the distal end' not_projecting_markedly_beyond_shaft_and_expand_gradually_if_there_is_any_expansion, 512 'Femur, distal articular surface' / 'uneven, fibular condyle projecting distally distinctly beyond tibial condyle' both_condyles_prominent_distally_and_approximately_at_same_level 'both condyles do not project distally (distal articular surface concave or almost flat)', 513 'Femur, anterior extensor groove' / 'absent, anterior margin of the bone straight or convex in distal view' 'present, anterior margin of the bone concave in distal view', 514 'Femur, surface between the lateral condyle and crista tibiofibularis on the distal surface' / smooth_ deep_groove, 515 'Femur, shape of lateral condyle in distal view' / 'lateral surface is rounded and mound-like' lateral_surface_is_triangular_and_sharply_pointed_, 516 'Tibia, total length versus total length of the femur' / '0.46-0.51' '0.60-0.65' '0.70-1.27' '1.41-1.46', 517 'Tibia, distinctly anteriorly projected process beyond the articular portion for the femur on the proximal end (= cnemial crest)' / absent present_and_anteriorly_straight_ present_and_curved_anterolaterally_, 518 'Tibia, proximal surface of the lateral condyle' / convex_or_flat depressed_, 519 'Tibia, lateral posterior condyle of the proximal end' / offset_anteriorly_from_the_medial_posterior_condyle level_with_the_medial_posterior_condyle_at_its_posterior_border, 520 'Tibia, lateral surface of the proximal half' / smooth 'with a longitudinal crest (= fibular crest)', 521 'Tibia, posterolateral process (= lateral malleolus) on the distal end' / absent present, 522 'Tibia, posterior surface of the distal end' / rounded distinct_proximodistally_oriented_ridge_present, 523 'Tibia, posterior side of the distal portion' / smooth_and_featureless dorsoventrally_oriented_groove_or_gap, 524 'Tibia, lateral side of the distal portion' / 'smooth/rounded' proximodistally_oriented_groove, 525 'Fibula, proximal end in proximal view' / round_or_slightly_elliptical transversely_compressed, 526 'Fibula, anterior edge of the proximal portion' / rounded_ tapers_to_a_point_and_arched_anteromedially, 527 'Fibula, proximal portion in lateral view' / symmetrical_or_nearly_symmetrical posterior_part_expanded_posteriorly, 528 'Fibula, transverse width at mid-length' / subequal_to_transverse_width_of_the_tibia distinctly_narrower_than_transverse_width_of_the_tibia, 529 'Fibula, area of attachment of the iliofibularis muscle' / not_on_a_prominent_process_ 'on a low, distinct tubercle' on_a_hypertrophied_tubercle, 530 'Fibula, location of the attachment site of the iliofibularis muscle' / near_the_proximal_portion near_the_midpoint_between_the_proximal_and_distal_ends, 531 'Fibula, distal end in lateral view' / 'angled anterodorsally (asymmetrical)' 'rounded or flat (symmetrical)', 532 'Proximal tarsals, articulation between astragalus and calcaneum' / roughly_flat concavoconvex_with_concavity_on_the_calcaneum concavoconvex_with_concavity_on_the_astragalus, 533 'Proximal tarsals, foramen for the passage of the perforating artery between the astragalus and calcaneum (= perforating foramen)' / present absent, 534 'Astragalus, crural facets' / 'separated by a non-articular surface' continuous, 535 'Astragalus, margin between tibial and fibular facets' / grades_smoothly_into_anterior_hollow separated_by_a_prominent_ridge_from_anterior_hollow_, 536 'Astragalus, tibial facet' / 'concave, flat or flexed' divided_into_distinct_posteromedial_and_anterolateral_basins, 537 'Astragalus, ascending process (= anterior ascending process)' / absent 'present, occupying most of the anteroposterior depth of the astragalus' 'present, restricted to the anterior half of the astragalar depth ', 538 'Astragalus, anterior hollow' / shallow_depression 'reduced to a foramen (= extensor canal) or absent', 539 'Astragalus, posterior groove' / present absent, 540 'Astragalus, anteromedial corner in proximal view' / obtuse acute, 541 'Astragalus, dorsolateral margin' / overlaps_the_anterior_and_posterior_portions_of_the_calcaneum_equally posterior_corner_dorsally_overlaps_the_calcaneum_much_more_than_the_anterior_portion_, 542 'Astragalus, articulation with distal tarsal 4' / poorly_defined well_defined, 543 'Calcaneum, articular facet for the astragalus' / lies_completely_medial_to_the_fibular_facet lies_partially_ventral_to_the_fibular_facet, 544 'Calcaneum, development of lateral margin' / calcaneum_terminating_in_unthickened_margin roughened_tuberosity_present_laterally, 545 'Calcaneum, calcaneal tuber (= expansion of the lateral margin of the bone)' / absent_or_incipient_ prominent, 546 'Calcaneum, orientation of calcaneal tuber' / 'lateral, between 0º?35º ' 'posterolateral, deflected between 36º?70º' 'posterior, between 71º?90º', 547 'Calcaneum, proportions of calcaneal tuber at the midshaft' / taller_than_broad about_the_same_or_broader_than_tall just_short_twice_the_transverse_width_of_the_fibular_facet, 548 'Calcaneum, calcaneal tuber distal end' / rounded_and_unexpanded 'flared, dorsally and/or ventrally', 549 'Calcaneum, calcaneal tuber distal end in proximal or distal view' / tapering_or_squared expanded, 550 'Calcaneum, distal surface of calcaneal tuber with a vertical median depression' / absent present, 551 'Calcaneum, ventral notch between the main body and the calcaneal tuber' / absent present, 552 'Calcaneum, ventral articular surface for distal tarsal 4 and the distal end of the calcaneal tuber' / continuous_ separated_by_a_clear_gap separated_by_a_gap_with_a_laterally_and_medially_delimited_ventral_fossa, 553 'Calcaneum, fibular facet' / slightly_convex_or_flat_ hemicylindrical_ concave_, 554 'Calcaneum, articular facets for the fibula and astragalus' / connected_by_a_continuous_surface separated, 555 'Calcaneum, articular surfaces for fibula and distal tarsal 4' / 'separated by a non-articular surface' continuous_, 556 'Calcaneum, transverse width of the distal articular surface versus transverse width of the astragalus' / '0.28-0.33' '0.42-0.48' '0.54-1.22', 557 'Distal tarsals, medial pedal centrale' / present_and_does_not_contact_tibia present_and_contacts_tibia absent_as_a_separate_ossification, 558 'Distal tarsals, distal tarsal 1' / present absent, 559 'Distal tarsals, distal tarsal 2' / present absent, 560 'Distal tarsals, distal tarsal 4 transverse width' / broader_than_distal_tarsal_3 subequal_to_distal_tarsal_3_, 561 'Distal tarsals, articular facet for metatarsal V on distal tarsal 4' / more_than_half_of_the_lateral_surface_of_the_bone less_than_half_of_the_lateral_surface_of_the_bone, 562 'Distal tarsals, proximal surface of distal tarsal 4' / flat 'distinct, proximally raised region on the posterior portion (= heel of Sereno and Arcucci, 1994)', 563 'Distal tarsals, distal tarsal 5' / present absent, 564 'Pes, foot length (articulated fourth metatarsal and digit) versus tibia-fibula length' / '>1' '<1', 565 'Metatarsus, configuration' / metatarsals_diverging_from_ankle_ 'compact, metatarsals I?IV tightly bunched', 566 'Metatarsus, metatarsals overlapping proximally' / absent present, 567 'Metatarsus, length of the longest metatarsal versus length of the tibia' / '0.20-0.23' '0.29-0.32' '0.37-0.59' '0.62-0.65', 568 'Metatarsus, metatarsals I and V mid-shaft diameters' / subequal_or_greater_than_those_of_metatarsals_II_to_IV lower_than_those_of_metatarsals_II_to_IV, 569 'Metatarsus, length of metatarsal I versus metatarsal III' / '0.17-0.21' '0.27-0.33' '0.38-0.42' '0.46-0.79' '0-93-0.97', 570 'Metatarsus, anteromedial portion of the shaft of metatarsal I' / smooth_or_slight_ridge 'distinct, rugose ridge present', 571 'Metatarsus, length of the metatarsal II versus length of the metatarsal IV' / '0.52-0.56' '0.60-0.85' '0.90-1.02' '1.06-1.15', 572 'Metatarsus, metatarsal II midshaft diameter' / 'less than or equal to the midshaft diameter of the metatarsals I-IV' more_than_the_midshaft_diameter_of_metatarsal_I, 573 'Metatarsus, metatarsal IV mid-shaft diameter' / subequal_to_that_of_metatarsal_III lower_than_that_of_metatarsal_III, 574 'Metatarsus, length of metatarsal IV versus length of metatarsal III' / '0.85-1.00' '1.04-1.08' '1.11-1.28' '1.31-1.34', 575 'Metatarsus, distal articulation surface of the metatarsal IV' / 'broader than deep (nearly symmetrical)' 'broad as deep or deeper than broad (asymmetrical)', 576 'Metatarsus, dorsal prominence separated from the proximal surface by a concave gap in metatarsal V' / absent present, 577 'Metatarsus, metatarsal V with a hook-shaped proximal end' / 'absent, articular face for distal tarsal 4 aligned to the medial margin of the shaft' 'present, with a gradually medially curved proximal process' 'present, with an abruptly medially flexed proximal process and, as a result, the metatarsal acquires a L-shape in dorsal or ventral view', 578 'Metatarsus, metatarsal V outer process on the proximal lateral margin' / 'absent, smooth curved margin' 'present, prominent pointed process', 579 'Metatarsus, metatarsal V lateral plantar tubercle in mature individuals' / absent present, 580 'Metatarsus, metatarsal V medial plantar tubercle in mature individuals' / absent present, 581 'Pedal digits, length of digit III versus length of digit IV' / '0.64-0.77' '0.82-0.83' '0.87-1.44', 582 'Pedal digits, phalanges on pedal digit V' / 'present and ''fully'' developed first phalanx' 'present and ''poorly'' developed first phalanx' absent, 583 'Pedal digits, ratio of lengths of pedal digits V and I' / '0.30-0.85' '1.37-3.07', 584 'Pedal digits, phalanx V-1' / 'subequal to or shorter than other non-ungual phalanges' 'metatarsal-like, considerably longer than other non-ungueal phalanges', 585 'Pedal digits, distal articular portion of distal pedal phalanges' / lateral_and_medial_sides_parallel_or_near_parallel_ lateral_and_medial_sides_converging_anteriorly, 586 'Pedal digits, pedal unguals' / 'weakly transversely compressed, rounded and triangular in cross-section' dorsolaterally_compressed 'strongly transversely compressed, with a sharp dorsal keel', 587 'Pedal digits, ventral tubercle in unguals' / absent_or_small well_developed_and_extended_ventral_to_the_articular_portion_of_the_ungual, 588 'Osteoderms, dorsal osteoderms' / absent 'present, one row ' 'present, two rows' 'present, more than two rows', 589 'Osteoderms, sculpture on their external surface' / absent present, 590 'Osteoderms, coarse and incised ornamentation composed of central regular pits of subequal size and contour on the external surface of the dorsal osteoderms' / absent present, 591 'Osteoderms, dorsal prominence on the external surface of paramedian osteoderms' / absent 'longitudinal keel, extending along all or most of the anteroposterior length of the osteoderm as a transversely compressed flange' 'blunted, anteroposteriorly restricted eminence', 592 'Osteodemrs, paramedian osteoderms' / thin very_thick, 593 'Osteoderms, relation between paramedian dorsal osteoderms and presacral vertebrae' / 'one to one (includes pairs)' more_than_one_osteoderm, 594 'Osteoderms, dorsal osteoderm alignment dorsal to the dorsal vertebrae' / staggered_ one_to_one, 595 'Osteoderms, dimensions of presacral dorsal osteoderms' / 'square-shaped, about equal dimensions' longer_than_wide wider_than_long_, 596 'Osteoderms, unornamented anterior articular lamina on paramedian osteoderms' / absent present, 597 'Osteoderms, anterior edge of paramedian presacral osteoderms' / straight_or_rounded with_a_distinct_anterior_process, 598 'Osteoderms, presacral paramedian osteoderms with a distinct longitudinal bend near the lateral edge' / absent present, 599 'Osteoderms, appendicular osteoderms' / absent present, 600 'Osteoderms, ventral osteoderms' / absent 'present, scattered, not forming a carapace ' 'present, forming a carapace ', 601 'Maxilla, posterolateral surface: directly adjacent to alveolar margin (0), lateral process of maxilla present, creating distinct space between maxillary alveoli and posterolateral surface of the maxilla (1)', 602 'Maxilla, medial surface dorsal to tooth row: smooth (0), prominent anteroposteriorly oriented ridge present (1)', 603 'Teeth, crown height of the upper dentition compared with lower dentition: similar tooth crown height (0), the upper dentition is shorter relative to the taller lower dentition (1)', 604 'Teeth, morphology of crown base: single, pointed crown (0), flattened platform with pointed cusps (1), mesiodistally arranged cusps (2)', 605 'Cervical and dorsal vertebrae, shape of posterior articular surface: planar (0), concave (1), convex (2)', 606 'Dorsal vertebrae, diapophysis, position: anterior portion of the neural arch/centrum (0), anteroposterior middle of the neural arch/centrum (1)', 607 'Dorsal vertebrae, spinoprezygapophyseal lamina: absent (0), present (1)', 608 'Dorsal vertebrae, spinopostzygapophyseal lamina: absent (0), present (1)', 609 'Dorsal vertebrae, height of neural spine in anterior dorsals: lower than two times the height of its respective centrum (0), equal or higher than two times the height of its respective centrum (1)', 610 'Caudal vertebrae, length of the anterior caudal vertebrae (caudal vertebrae 1?10) relative to posterior caudal vertebrae (25+): nearly the same length (0), posterior caudal vertebrae much longer (1)', 611 'Clavicle, portion articulated with the interclavicle, shape: broader than distal portion of clavicle (0), similar in narrowness to the distal portion of the clavicle (1)', 612 'Humerus, entepicondylar crest: exhibits a curved proximal margin (0), exhibits a prominently angled proximal margin (1)', 613 'Ilium, postacetabular process in lateral view: squared or rounded (0), subtriangular, tapering posteriorly (1)', 614 'Pedal digits, penultimate phalanges (last phalanx before ungual): shorter or sub-equal than the more proximal phalanges (0), distinctly longer than the more proximal phalanges (1)', 615 'Primordial sacral vertebra two, sacral rib' / 'consist of a single body in one plane (could contain a lateral notch)' has_a_separate_posterolateral_process_positioned_dorsal_and_posterior_to_the_main_body_of_the_sacral_rib, 616 'Scapula, posterior edge of the blade just dorsal to the glenoid region' / smoothly_transversely_convex 'with a distinct, longitudinal sharp ridge', 617 'Ilium, ventral portion, ischial peduncle, lateral view' / nearly_straight_or_slightly_concave 'distinct notch (=dorsal expansion) between the posterior and anterior ends', 618 'Femur, distal end, medial condyle in posterior view' / smooth_surface_or_a_small_depression well_defined_proximodistally_oriented_scar_extending_from_the_posterior_portion_of_the_condyle_well_proximally, 619 'Fibula, anterior edge' / gently_rounded distinct_ridge_paralleling_the_shaft, 620 'Proximal tarslas, fusion between astragalus and calcaneum' / absent present ;

MATRIX

Petrolacosaurus_kansensis 0000110000010-0000101000000200000100---00(1 2)10000-0--10---0---0--000-0001000300000-0-000--0000000-00000-?03?0100001101-0-000000000-000000-0000--000?0001??3???1101-0-0100000000000-0-0?-10001000000000000110?0000010001000?00000??00-00-10?00?2?0????0?100?--??????0-000-0000101010-0-1000010-00100000?00000000000010000000?0000020--00010110100?010100100100-0010200000??2(0 1)0110010000??0030000?00?0?010010100?20000-0001000000000?1000000001012?00011000000010?02000000000?0000100111000000020-10-20?00001?0000?????00001000??2001??200?00000??000?000000000??0000-------00020000100001203?0002?101??00100000----------0000001000000000??0000

Acerosodontosaurus_piveteaui 0?0?????????0-?0?0???????????????????????????????-???--??????--0???0??10004???????-010--000?000-??000000?0?????0?1?1???000??00????00?0????????0002?????????????1???????????????0-??0?-????????????????????0???0?????????????????????????????????????????????????????00-???2???????????00??10??0?????000??100?00000?000000?0000000--??0?????????00?1???0?-?0?0011?01000000001-001??????1???????0????????????????????0????????????????????101000-?00?110000001??0100????00?100-010000010?001020-00-?????0??1?000??-??00000000?0??1????0?????????0????????????????????????????????????????????????????????????0----------00?00010000??00???0???

Youngina_capensis ?0000-0000010-000011?00000010100?11110-01300000-1--00---0-0-0--00??0001000310000-0-010--??10100-10010000000000001101-0-000100100-1000010101000000200010020001101-0-01000000000002000?-00000000100000100101000?00100011001000011001000-1000002-0000010100?--0000000-000-00?010?(0 1)10-0-?0000110?00000010?00010010(0 1)0(0 1)000?0000?0000000--0??0????0000000100000-00-0?11000000000001-001?00?11(0 1)0100001201000100?0?00020000-?000010001000000000?1101000-00?11100?00110201000000000100-0000011100001020-00-001000011000000-0100??0000?00111?0200?00??????0??1000000????0000-------0002000010000120(2 3)01002?00100001000010-001-200-000?0010000?10000?0?00

Paliguana_whitei ??000-00????0-?000???0?????????????????0?????????-???--??????--???????1?????0?????-0??--???0100-?00??????????00??10?00-??10000?010000010????????10?00???????2-??-????000002-1??22?0???0????????0?????????2?????????????????????????0?????????????????????????????0-??0-?0??00?????????00???0?0????????????????200000????????????????????????????????????????????????????????????????????????????????????????????????????????????????????????????????????????????????????????????????????????????????????????????????????????????????????????????????????????????????????????????????????????????????????????????????????0??0????????????????

Planocephalosaurus_robinsonae 0?000-0000000-0000?01000000201000100---00210?00-?--10---10011--000-0001000200000-0-010--??00001-00-10010?0000001010100-0011000200000001000010001010000------2-10-0-0100?002-10022000?00?000001101012-00002000?0???0??????????0??01010-10??0?0?000????110???????????020-?02200?00110-0000--2010000-011????1301020(0 1)000?0000?0000000--??1?001?000?00?1???0??00?00?2?00000010101?001?00?1111???00??0000001101001020000-101?01??0??0???0?????100???????????????????????????000?00-0100101101000000-0--10???01????0???-???0000000?0?1????????0???????????-1000001??1?00-------0?0????????????????????01111????????????????????000010000?100?0?00?1

Gephyrosaurus_bridensis 0?00110000010-0000?00000000101000100---00110?00-1--10---10001--000-0001000400000-0-010--0000001-00-10000-0000001110000-001100020000000100010100000000(0 1)000--02-10-0-0100?0?2-10021000?000000001101011-0010200010???0???????0000??01010-10000?0?000??10110?01????????020-00020010011{1 2}11000012010010101100??12010200010?0000?00000??????1000?00000000000?0??00?00?3001000010101?001?0011011?0??01{0 1}0?0?01?0110???200?0-100001??0??0???0?????100???????????????????????????0?0?00-0?00000101???000-1--???0000??00?0?????00001000?0?1????????0?????0?000?-100000?0?1?00-------0?0?????00?????????????11111?{0 1}??1?1?????????????00001000??10??0?0?01

Cteniogenys_sp. 0?10100010??0-??00???00?{0 1}??{3 4}?000?000---00{1 2}00??0-0--10--?1{0 1}101--000-00010003?0?????-010--0100000-000100?00?1-0000-102-0-0?10010?0-000001???????0???0??10?3??1??00-0-2-?0?0?2-???130?0000101?000?0??01000???0??10???0??2????0000??010?1000000?0?000?0?0120?01?0??????000-?0?01000?11???00001????0???????????0010000100?101??00000?????002????000?????0??????????110010??????????????????0????00???????????????????????????????????0000000?1111-1?00?????????????????????0?0?00-0?000101?????????????????????00?000-0100000000?????????????????????????????????????????????????????????????????????????????????????????????000010?????0????(0 1)???

Simoedosaurus_lemoinei 0?100-0010200-000030?000011?0?000--0---00301?00-0--?0---10001--000-00000004101?0-0-010--??00000-00-11000201-0000-102-0-002--1000-0000011000010000000110020022-00-0-2-101102-1001?0100?01010000?0103--0011000010110001200101000101101101000000-0000010120?01?000-0??000-00010000110{1 2}01000011000000101010???0010100000?101000000010(0 1)-100000001000000100000-???0010001000000001-?0111010-0?1000?1?1100011101000020000-?01101010??001000100111(0 1)00(1 2)1000?110????????????????000?0000110000100?01000-0--?000000??00000000100001000?0111001?00100000000?0?0?????????????????????????????????????????????????????????????????????00?011000?100?00000?

Aenigmastropheus_parringtoni ?????????????????????????????????????????????????????????????????????????????????????????????????????????????????????????????????????????????????????????????????????????????????????????????????????????????????????????????????????????????????????????????????????????????????????????????????????????????????????00010111000{0 2}?????????????000?1???????????1???100?000?0?????????????????????????????????????????????????????????????111002000???????????????????????????????????????????????????????????????????????????????????????????????????????????????????????????????????????????????????????????????????????????1100???0????????

Protorosaurus_speneri ?0000-0200010-000011??0000020100?10110-0?300?00-1--10---10???--00??0001000310000-0-01?--0000000-0000??00-00000000100-0-??0000000-0000010001?100000000???????2-(0 1)0-0-{0 1 2}?10000??10012?00?0?0??200?????{0 1}????????????010?0110010?????????1???????????????????????????????000-?0?2000010-{1 2}010000020000?00010?????0010(0 1)00000?100101111020--0?0{0 1}0011?1000(0 1)?100110310?2110?100010010010101?0000-000000?100000011011?00011010-?0000000?0010?000000111101201?011000000101202?00010????0{0 1}?0?000???00??1?20-?0-????0011?00?0??-???0??0000?1101???20???000???010??00000001??0000-------00020000001001(0 2)030100(0 2 3)?11100(0 1)0100000----------00000010?000?0?00??0?0

Amotosaurus_rotfeldensis 0-?0100?????????10????{0 1}0?0??????????????0110?00-????0???1110???00??000?0003????????0???????0000?00?0?000-00?0?001?0???????????????????????????????????????????????????????????????????????2000??003--00102??????????????????????0?????????0?0?0????1?000???????????000-00?200?010-0-?0000??0000???0??????100101001?011001?1100?00--1???1112210??0?10?111-1??211(1 2)00?001??0011-(0 1)01000111??20????01?00110000?00010000-?????????1??0?0000?1?0??1-1?(0 1)?0?100???????000??0???0???0100111000101001100-0--10??001???0???????00000000?1??1???20?????????01????0???0????0??0-------00022101??1111203?1002?011??1?110{0 1}00----------0000001???0????00?0?0?

Macrocnemus_bassanii 00000-0200010-0000?10?0000120000?0021000010??00-?--1?---11101--00??00010003?0?0?-?-0?0--???0000-000???????000000110100-01000100000100010?00???????0?0?????????01-0-2-10?0????0?1???0?0??0?0010???001200???????????????0????????????????????????????????????????????000-?0?0?000??????0000????00?00????????001000000011001?1?10000--1???1111210?10?111110-(0 1)??21130?0001000011-1010101111020?00101?0011?000?0??10000-10110000000?0??000???0111-0-000(1 2)100??????110?0000000?01011011101(0 1)101001020-00?10??0011(1 2)?0?0?????0000----?1??1???2???00??0??010??00000001??0000-------00021100001111203?1002?12011?0100000----------00000010?00010100?0??0

Tanystropheus_longobardicus 0-000-0200010-0{0 1}1001?00000120000?0021000010??00-0--00---11101--000-3001000110000-0-010--0000100-0001?000-0001000111000-01110(0 1)01000(0 1)0001000-1001-----00------??11-0-0(0 2)1000?2-10011000?0?0002(0 1)1010005-2-010200??0010101?00??0?00?00??01?10?????????0?1?000?11??10-?0-000-?002000010-0-10000120000000010?01110(0 1)1(0 1)(0 1 2)0010(0 1)1100101111000--2?0111132100101110111-11?212?200001000011-10100010-10{1 2 3}100010110011{0 1}000?00010000-10???0?0?0010??00001?0111-0-0?0(0 1)10000111010010100010?01011021120(0 1)101001100-0--10??00111100000-0100000000?110110120???000???000?100000001??1010-------00022110001111203?2000?0111020110000----------00000(0 2)11000010100??0?0

Jesairosaurus_lehmani 0?000-00????0-?000{1 2 3}1?000?01?0????????????100?00-?-?10--?1000?--00??00010002???????-010--??00001-00-10000-00????01101-0-??0100020-1100?100010000000000?????????01-0-000000?2-10?12?00?0?????00??0?03--00102?????0?0??11??????????0??1??1???0?0?0????????????????????000-00?210?010-0-???00020000??001??????{0 4}01010???01?00??10?0?00--{0 1}??{1 2}0000????00?1???00-?????200(0 1)0000??(0 1)001-10?00????1?100???00?010?11???0002??00-10110001?1?10??0?0???0101-??????????????????????????0??0(1 2)(0 1)0?10000100?01100-0--?0?0?00???0???????????----?0??????{0 1 2}????????1?110??????????????????????????????????????????????????????????0----------000?00?0000?110???????

Pamelaria_dolichotrachela 0?000-0110??0-0{0 1}10{0 1}???{0 1}??01??0???--20??0?20??00-?-???---111??--00??0??1000{1 2}??????0-010--??00000-001?0?00-0000010?00?0?-0000000001?100010????00??????11??????2-0?-????101002-10?120011110000011?0??1?-?0??????0?0100011?0?0100?10011110000?11?10000010000???0?1?????020-0012110010-0-01000?110???0?01?00???10?0211000?1001011110100-100100?12100000100100-?1??1100(0 1)0111101001-00100011010??0002?1100011001?00000100-?0????????01100010001111010001011000?00??12000011?0000101001100001000000102000100010010100000-0100000000?020010120010000010011102001000000001100000100001100000??01203?10020120??????1010----------0?00?0100000?010000000

Azendohsaurus_madagaskarensis 0?000-0110010-0110112000001100001--200000200?00-0--10---11(0 1)00--100-00010001?000?-0-01?----00000-00110000-00??0101100011??00000001110001000?0?000000011000-01??0000-02111002-10?12001011000201111?02-1001010000001010011020100010111010000?11211????10?0??1?0?11??10000-0022001010-0-10000?1??01?10010?01111010221001?100101111000--1010001(0 1)21001?0100110-11?{1 2}110000101101001-10000000-1010000111100001101000010100-?00001010?01100000001111011001(0 1)??00000010??02011110000?020011000(0 1)100?000111000?0?0100??00000000100001000?0200101?00100000100?0?020010000000011000000000021000001?01?030100101201020101010----------0001101010001010000000

Azendohsaurus_laaroussi 0???????10??0-????????0?????0???1??2????0????0???-?10--?1110?--??0-000?000{1 2}????????????????????????????????????????????????????????????????????????????????????????????????????????????????????????????????????????????????????????????????????????????????????????00???0220010??????000??????????????????1010221001????????????????????????????????????????????????????????????????????????????????????????????????????????????????????????????????????????????????????????????????????????????????????????????????????????????????????????????????????????????????????????????????????????????????????????????????????0110????????????????

Shringasaurus_indicus 0?0?0-0110010-?????1?0???011{0 1}1001--2000?0200?0???-??0--?1????--????{0 2}???000{1 2}?00?0-0-010--???????-??????????0??000100000-00000??????????????????0??????1????????0010-??11?0?????0120?1011?002?1???????????????????????????????{0 1}?1?111?10001?11011???0?0000????????????20-??????1????0-??00??????????????????1??0221000?100101111020--???10??011001001000001?1??1100001111010014?0100010-1??00002?1?00?011010??0101010?00001?10?0110001000211101100?0???0????????????????000?010011010(0 1)10??000{1 2}1?????000?0???10000000100001000?0200????0010000010010?02?010000000??????????????????????01??????????????????1??0----------00001010111?001?000000

Trilophosaurus_buettneri ??000-0200010-0{0 1}010?21001-1200010?02000?150-000-0-010---10101--10??01010001?1000-0-010--???0001-00-?0?-??-001010110000-??01000201?00001010-1001-----11?-?-??2-00-0-??101002-10012001?100???1-1???1----0??????1?0101001?030100010111110000?1101000??1?000?11????????220-102200?0?????110001111000100?0?01111010202000?100100011000--0?11000021001?1100010-01??11(0 1)000-11000001-00100000-101000012000{0 1}011101000020100-100000011?010000110011110110010110000?0001202001110000{2 3}00-0110{0 1}00100001000-1--10000001100000100100001000?010000120010000010010?02000000??00011000000000021000001001203?1002?120??00101010----------0010012100111111000000

Trilophosaurus_jacobsi 0?000-01????0-?001???10???????????????????????0-?-??0--??0???--????0??{0 1}000{1 2}??????0-0?0--???0001-00-???-??-0?00?0?00??0-???100020??000010?0-?00???-??11?-??????0?00-2-10??????0????0????????????????????????????????????????????????????????????????????????????????220-?022???0??????1000?????0??0????????10?0202000?1?01????????????11????21001?1100?10???????????????????????????????0???00????0???????????20?00-???????????10000010011110110010????00???????2????1?0?0?0100100?0010??01000-1--??????0??00000000100000000?0?00001?00100000???????2000000000001100000000002?????0???1????????0?????????1?10----------??10012??1?1?1?1??00?0

Spinosuchus_caseanus ?????????????????????????????????????????????????????????????????????????????????????????????????????????????????????????????????????????????????????????????????????????????????????????????????????????????????????????????????????????????????????????????????????????????????????????????????????????????????????100101111000--0??1???0210?1?1?0???0??1???1(0 1)000-11?0?001-?010?0?0-????3????????????????????????????????????????????????????????????????????????????????????????????????????????????????????????????????????????????????????????????????????????????????????????????????????????????????0----------??????21011?????0?????

Spinosuchus_combined 0?000-01????0-?001???10???????????????????????0-?-??0--??0???--????0??{0 1}000{1 2}??????0-0?0--???0001-00-???-??-0?00?0?00??0-???100000??000010?0-?00???-??11?-??????0?00-2-10??????0????0????????????????????????????????????????????????????????????????????????????????220-?022???0??????1000?????0??0????????10?0202000?100101111000--0?11???021001?1100?10??1???1(0 1)000-11?0?001-?010?0?0-?0??300????0???????????20?00-???????????10000010011110110010????00???????2????1?0?0?0100100?0010??01000-1--??????0??00000000100000000?0?00001?00100000???????2000000000001100000000002?????0???1????????0?????????1?10----------??1001210111?1?10?00?0

Teraterpeton_hrynewichorum ??000-0001010-00010{1 2}20000-141??000010000?5--??0-?--10---1010?--00??0101000210000-0-0?1--??10000-00-000-02-010010110100-??00000201000?01010-?00001-0011-----12-01-????101??2-10011001?0???0?01???????????????0??0101011?0?????0?????010????0??????001?????????1???0?00?????2?0??1????1100??10??0?10?10????01010200001?1?00??????00--???0???0{1 2}00?1??10?010-11??1???????1?00???-10???????????????{0 1}??0??011?1????2???0-?0?????????????????????????????????????????????10???????????????????????????????????????????????????????????????????????????????????????????????????????????????????????????????????????0----------??0?0110????1?????????

Noteosuchus_colletti ?????????????????????????????????????????????????????????????????????????????????????????????????????????????????????????????????????????????????????????????????????????????????????????????????????????????????????????????????????????????????????????????????????????????????????????????????????????????????????1?0???????0{0 1}??????????????????????????????0000??1???001?0?1000111?0200?0?0??????????????????????????????????????????????????0????00001??1?2?0100?0??100-0110(0 1)0010000??2????01??0?00??100000-0100000000?0201101200100000??01???200?000?0000110000000000?{0 1}000001?01202?1002?12010?0?000?0----------00?????0??0????00?00?0

Mesosuchus_browni 0000100110210-0110101000201111001--200010400?00-0-010---10001--00100101010-00000-0-010--0100000-01010000-00000001000010??10101001000101000000000010001000-02001010-00101002-100120000000011000000000000112010110100011112010?01?110110100?0101000??10100?1???110?0-000-00?000?01????002100210110010100???01010-00010?1001?0000000--1?010000110010?100000-1???110000?01??1001-0010001101020(2 3)??00000{0 1}011100?00010000-10100001000??????????1111-0-00??10000???0??0200??0?00020100100001100001010-0000000?00??100000-0100000000?020110120010000010?10?1200?0000?00011000000000021000001?0120201002?120?0?0?00000----------00000010000000000?0000

Howesia_browni 0?00100210?????{0 1}10{0 1}??00????????????????1????????????????1???1??00??1??1111-?0100?0?010????00000?010?0000-00000001000-10001010?00-000?01000?00000000?0???????0?10-0-1??0?0?2-1?0120?0?0?0???000?0{0 1}001000102010010100?1?11??1??0???101???00?0?0?000??1?000?????????0-?{0 1}0-???????????????21??21??1?01??0?????10?0-00010?1?0?????????????0{1 2}000?????????????????????0?00??1??1????0??000?101??0(1 2)0?0011??00?000??0010?????01001?????1?????????01????????????????????????????0?0?0100100000100001020-00010???00???0?000-0??0000000?0?011?1?00100000?0010?1200?00?0?000110?0000000021000001?01?0???00??120???0?0???0----------??0?00?00?????0?0?0000

Eohyosaurus_wolvaardti ??00??02???????110{0 1}???????????????????????????11???????????????0???1??1111-??????????????100000?01110000-0??????????-??00{0 1}??0100-100101000000000011001001002001?-????1?1?02-1??12000000?0?201??0??{0 2 5}???0????????0?0??11??????????????????????????????????????????????00-???1????10-{1 2}0??210021011?1101??????1???-000101?????????????????????????????????????????????????????????????????????????????????????????????????????????????????????????????????????????????????????????????????????????????????????????????????????????????????????????????????????????????????????????????????????????????????????????????????????00????????????????

Rhynchosaurus_articeps 0000100210210-00200010-12-1-2-000--20002?5--?0111-010---10001--00??1101111-10100-0-010--0100100-00111000-00000000000-11??1000100-1001010000100000000010010020110-0-2-100002-1001?00000000?31-0?000----0102??1111??00111????????0?1?1??0???01??000???0000???0?1?????110-?0?10000?????0122002?0?100101000??01010-000101100??0000000--1??????0?10?00?10000?-11???1?000?0100000?-1??00011110?0(0 1)0?0010000011?0?0001000111010000100010?00000011111-0?000110000????1?01?00?010?0?0100?001(0 1)0100?010?0-0??10???00?2?0????????000000000??{0 1}???20???0000???1???2?0?00?0??0?110?0??00000?100?0??001202?1002?110?01??000?0----------000000?00000?0?0000?00

Bentonyx_sidensis 0?000-0210210-00200010-12-1-2-000--20002?5--?0101-010---1000?--00??1?01111-10100-0-010--01?1000-001?1?????0000000000-11??1010100-100101000?10?00????0???????1110-0-2-100002-1001?000???00131-0?000----01?2?????1???0111?{0 2}0????1?110110000?010?1??????100???????????110-?0?100?01?????122????0?1???????????1?1?-00010????????????????????????????????????????????????????????????????????????????????????????????????????????????????????????????????????????????????????????????????????????????????????????????????????????????????????????????????????????????????????????????????????????????????????????????????????00?0????????????????

Eorasaurus_olsoni ?????????????????????????????????????????????????????????????????????????????????????????????????????????????????????????????????????????????????????????????????????????????????????????????????????????????????????????????????????????????????????????????????????????????????????????????????????????????????????1001?11100?{0 2}??????????1?000(0 1)?11???0?011??1?????1????????????????????????????????????????????????????????????????????????????????????????????????????????????????????????????????????????????????????????????????????????????????????????????????????????????????????????????????????????????????????????0??????????????

Prolacertoides_jimusarensis 0??0??0?00?10-???????00????????????????0?????00-?-???--?10000--0???2?010002?00?0?0-010--???0000-??????????00100?????????????????????????????????????????????????????????????????????????0?31-0?0?0????01??000?0???????????????????????????????????????????????????????????????????????????????????????????{0 4}???2000??????????????????????????????????????????????????????????????????????????????????????????????????????????????????????????????????????????????????????????????????????????????????????????????????????????????????????????????????????????????????????????????????????????????????????????????????????0??0????????????????

Prolacerta_broomi 00000-0(1 2)00010-0(0 1)10111001201210?00?0210001100000-?-010---100?0--000-000100031000?-0-010--0000100-010100(0 1)0-0000000110100-0010000000100001010000000000001000-011101(0 1)0-(0 1 2)0101002-1001(1 2)00001101000001100000011010110?01000111000110000010110(0 1)000002100010101000110011000-000-0000100010-0-000000210001110100011110100010001100111010020--1001101121000(0 1)(0 1)1011103011{1 2}111000001001001200100011010100?0??0000010000000011000-101100010?000000100010111-0-0??11000??0??1?010000?000010100{1 2 3}01001100001020-00-1000001??100000-0100000000001001012001000001??10?120010000?0001100000?00002100000110120301002?120??20100000----------00000010000010?0000000

K_australiensis_holotype ???00??1?????????01????????????????????????????????????????????????????????????????????????????????????????????0???100-??{0 1}00?00?0?????101000?0000{0 1}00?1000-01{0 1}?0111000101002-1?????0???????????????????????????????0???10????????0?????????0?2?000????100????????????????????????????????0?2??0??1??????????????????????????????????????????????????????????????????????????????????????????????????????????????????????????????????????????????????????????????????????????????????????????????????????????????????????????????????????????????????????????????????????????????????????????????????????????????????????????0????????????????

K_australiensis_combined ???00-?1????0-???01???????????????????????????0-?-???--?1000?--0???{0 3}??{0 1}00????????0-???--???????-??????????00???0???100-??{0 1}00?00?0?????101000?0000{0 1}00?1000-01{0 1}?0111000101002-1?????0???????????????????????????????0???10????????0?????????0?2?000????100????????????????????????????????0?2??0??1??????????????????????????????????????????????????????????????????????????????????????????????????????????????????????????????????????????????????????????????????????????????????????????????????????????????????????????????????????????????????????????????????????????????????????????????????????????????????????????0????????????????

Boreopricea_funerea ??000-0?????0-?0?0{0 1}??00????????????2???011???00-?-??0---1000?--00??0??100?3?0????0-010--??-??00-0?-0------0000000100?0-0?000?000??000010????00???????1000-?1???????1010?0?2-10?12000?10?1?{0 1 2}0???1??????1????????????????????????????????????????00????1?????????????000-00?200??????????00????0????01??????{0 2 4}01000?0?0?1001?1000020--???0???0210010010??00{1 2}1???11?00000100100?{2 3 4}??1????????????0??100001?0000000???????0??00???100?0000000?0111-0-0??1100???????20?0?00?0??????????????????????????????????12?0????????????????010????2?0??0???1?01??02???000???00110000000000?1??000??01?0?0?001012010?0?0000?????????????0??010000??0?0?0??00

Archosaurus_rossicus_holotype ????????00?1???????????????220010?1201??1101??????????????????????????????????????????????????????????????????????????????????????????????????????????????????????????????????????????????????????????????????????????????????????????????????????????????????????????????????????????????????????????????1?????????????????????????????????????????????????????????????????????????????????????????????????????????????????????????????????????????????????????????????????????????????????????????????????????????????????????????????????????????????????????????????????????????????????????????????????????????????????????????????????

'''Proterosuchus ferugsi''' ??0?????????100???????{0 1}?????????????????????0??????????-1100?020???0?0?0??{2 3}?0????????????????0?????{1 2}0??0????????????????????????????????????????????????????????????????????????????????1?{1 2}0001100000?100101101????????????????????????????????????????????????????????????????????????0??????????????????10100????01???????????????????????????????????????????????????????????????????????????????????????????????????????????????????????????????????????????????????????????????????????????????????????????????????????????????????????????????????????????????????????????????????????????????????????????????????????????????????????

Proterosuchus_fergusi {0 1}?000-01000110021021110121122001011201001101000-010(0 1)00--110000200100000000320000-0-0101100001000000201100(0 1)000000000000-??100010001000010101000000100010(0 1)(0 1)-02110(0 1)(0 1)11(1 2)010100011001200001001??0001100000010010110100001111000101?00010110101?01210000010101?111?11?01?0010001110?01110-00000111010110010101111010011000110011100(0 1)020--100(0 1)0010110(0 1)0(1 2)0100000201?210?01001110(1 2)001(0 2)001??????1?????0?0?????????????????????01101100?????????????????01000?1000100???20200?0??????????????????????????????????????100010-010000000000201101?001????????????2001000000001100?????000?1000001?01?03010020?2??0?0?00000??--------00000010000????0???0?0

Proterosuchus_goweri {0 1}?000-010001100210{2 3}1{0 1}00121122?010?1??1?01101000-010?00--11?0002001021000003?00???0-01011000??0??00120?10??0000000?0000-00100?1000??00010?01???000?0001????????000111010100011??130000100112000?1000{0 1}?01???01?0101001111030101?0?010110101?01210000010101???1?110?10????????????????????????????????????11?1?????1??0????????????????????????????????????????????????????????????????????????????????????????????????????????????????????????????????????????????????????????????????????????????????????????????????????????????????0010????100?0?0?????????????????????????????????????????????????????????????????????00?0??????????????0?

Proterosuchus_alexanderi ?0001001?0??1002102??{0 1}01????2??????????0??????0-?1?????-11?00?20???0?0?0003{1 2}0????0?0?0?????01000???20110-0000000000000-??10001000100?01010100000010001000-0211000112-101000110?1300001001??00????000001001?11?101001111000101?0?010110101?0?2?0??????101???1?11??10001000011?001110-00000111000?10??010??1{0 4}010011000110011?000020--1?000010110?0101000001011210??1?011?010014?0?000111101001?10?000010000?00010000-1011011000???0?0?000??????010?0????????????????????000?0100?1??00100?01020-00-?000?00??10?010-01000000000????????????0000???1??120010000000011000001000011000001?01?0?0?00??12000?0?0???0----------?00?0010????0???0????0

'''Chasmatosaurus'' yuani' 1?000-010001100210?11001?11(2 3)2001011201001101?00-011000--110000200100100000320000-0-01011??00100000120110-1000000000000-??1000100010000101010??00020001000-02???000-2-1010?{0 1}110?130?001??1?2000?1?0{0 2}?0??001?11?1?????111?30??1?0??11110000?0?210??????001???????????001?001110001110-?000011102?110010????1(1 4)010011000?100??1000120--???{1 2}?????????0?1???0?{0 1}???{1 2}?100(0 1)0??1?0{1 2}001310100010-1030010?{0 1}00000100???00010000-1010011000001000001011011-(0 1)10001100????????0?0?10??000?0100110000100?01020-00-?0?0?0011100010-0100000000?0201101(1 2)0010000010010?120???0?0??00110011000000?{0 1}000001?01?????????120??????00000---------000?0010000001?00??0?0

'''Chasmatosaurus ultimus''' 2??0????????1??????????????????????????????????????003???????010?210?1?0001?????????????????????????????????????????????????????????????????????????????????????????????????????????????11???1110?????0??????11????????????????????????????????????????????????????00???001010111?0-?000?????????????????140100{1 2}1000????????????????????????????????????????????????????????????????????????????????????????????????????????????????????????????????????????????????????????????????????????????????????????????????????????????????????????????????????????????????????????????????????????????????????????????????????0?00????????????????

Ankistrodon_indicus {0 1}?????????????????????????????????????????????????????????????????????????????????????????????????????????????????????????????????????????????????????????????????????????????????????????????????????????????????????????????????????????????????????????????????????????????????????????????????????????1???0110?0???????????????????????????????????????????????????????????????????????????????????????????????????????????????????????????????????????????????????????????????????????????????????????????????????????????????????????????????????????????????????????????????????????????????????????????????????????0????????????????

Tasmaniosaurus_triassicus 0?0?????????100???????????1?10?????20????10??????{1 2}?????-110??0100??0???000{2 3}???????????1????????????????????????0?1?0???00?????????????????????????????????????0????2-?0???01??????????????????????????1????????????????????????????????????????????????????????????0????0?0??001100-?000?????????????????110?00{1 2}10?0?100??1010????????????????????????????????1??1??????10???????????????????1{0 1}?????????????????????01100010???????????????????????????????????????????????????????????????????????????????????????????????????????{0 1 2}??????????????????????????????????????????????????{2 3}0???????020????????0??????????????00010?1????????????

Exilisuchus_tubercularis ????????????????????????????????????????????????????????????????????????????????????????????????????????????????????????????????????????????????????????????????????????????????????????????????????????????????????????????????????????????????????????????????????????????????????????????????????????????????????????????????????????????????????????????????????????????????????????????????????????????????????????????????????????????????????????????????????????0?0110??1?001???????????????????????????????????????????????????????????????????????????????????????????????????????????????????????????????????????????????????????

Blomosuchus_georgii ????????????????????????????????????????????????????????????????????????????????????????????????????????????????????????????????????????????????????????????????????????????????????????????????????????????????????????????????0??????????121000?0?0101?11?????????????????????????????????????????????????????????????????????????????????????????????????????????????????????????????????????????????????????????????????????????????????????????????????????????????????????????????????????????????????????????????????????????????????????????????????????????????????????????????????????????????????????????????????????????????????

Vonhuenia_fredericki ?????????????????????????????????????????????????????????????????????????????????????????????????????????????????????????????????????????????????????????????????????????????????????????????????????????????????????????????????????????????????????????????????????????????????????????????????????????????????????1001110110101-???????????0?1?1?????-?????????????????0?????????????????????????????????????????????????????????????????????????????????????????????????????????????????????????????????????????????????????????????????????????????????????????????????????????????????????????????????????????????????1???????????????

C_rossicus_combined ?????????????????????????????????????????????????????????????????????????????????????????????????????????????????????????????????????????????????????????????????????????????????????????????????????????????????????????????????????????????????????????????????????????????????????????????????????????????????????100111111020--???1???0111(0 1)02010?000?0?????001???1001?0?????????????????????????????????????????????????????????????????????????????????????????????????????????????????????????????????????????????????????????????????????????????????????????????????????????????????????????????????????????????????1011????????????

Chasmatosuchus_magnus ?????????????????????????????????????????????????????????????????????????????????????????????????????????????????????????????????????????????????????????????????????????????????????????????????????????????????????????????????????????????????????????????????????????????????????????????????????????????????????1?01???1?????????1????111?02010?000-???????????????????????????????????????????????????????????????????????????????????????????????????????????????????????????????????????????????????????????????????????????????????????????????????????????????????????????????????????????????????????????????????1???????????????

Gamosaurus_lozovskii ?????????????????????????????????????????????????????????????????????????????????????????????????????????????????????????????????????????????????????????????????????????????????????????????????????????????????????????????????????????????????????????????????????????????????????????????????????????????????????1?01?11111???????1???01110020?0????????????????????????????????????????????????????????????????????????????????????????????????????????????????????????????????????????????????????????????????????????????????????????????????????????????????????????????????????????????????????????????????????????1???????????????

C_magnus_combined ?????????????????????????????????????????????????????????????????????????????????????????????????????????????????????????????????????????????????????????????????????????????????????????????????????????????????????????????????????????????????????????????????????????????????????????????????????????????????????1?01?11111???????1???0111002010?000-???????????????????????????????????????????????????????????????????????????????????????????????????????????????????????????????????????????????????????????????????????????????????????????????????????????????????????????????????????????????????????????????????1???????????????

Chasmatosuchus_vjushkovi {0 1}????????00????????????????220010?0201??110???????????????????????????????????????????????????????????????????????????????????????????????????????????????????????????????????????????????????????????????????????????????????????????????????????????????????????????????????????????????????????????????1????{1 2}1??????????????????????????????????????????????????????????????????????????????????????????????????????????????????????????????????????????????????????????????????????????????????????????????????????????????????????????????????????????????????????????????????????????????????????????????????????????0????????????????

SAM_P41754_Long_Reef ?????????????????????????????????????????????????????????????????????????????????????????????????????????????????????????????????????????????????????????????????????????????????????????????????????????????????????????????????????????????????????????????????????????????????????????????????????????????????????1?0???????1{2 3}10?????????????????????????????210??1002001-???????????????????????????????????????????????????????????????????????????????????????????????????????????????????????????????????????????????????????????????????????????????????????????????????????????????????????????????????????????????11000???????????

Koilamasuchus_gonzalezdiazi ????????????????????????????????????????????????????????????????????????????????????????????????????????????????????????????????????????????????????????????????????????????????????????????????????????????????????????????????????????????????????????????????????????????????????????????????????????????????????????????????{0 1}?????????????????????????1??????20???????0???????????????????????????????????????????????????1???00????0??1-?????????????????????????0???021?{0 1}1??00???????????????????????????????????????????????????????????????????????????????????????????????????????????????????????{2 3}0-10?11010??????????????????0???

Kalisuchus_rewanensis_holotype 2?00????????10??????????????2??????{1 2}?????????0???{0 1}?000-?1{1 2}???0???10000?000{2 3 4}???????-???????????????????????????????????????????????????????????????????????????????????????????????????????????????????????????????????????????????????????????????????????????????????????????????????????????????????????1???0210?0?????????????????????????????????????????????????????????????????????????????????????????????????????????????????????????????????????????????????????????????????????????????????????????????????????????????????????????????????????????????????????????????????????????????????????????????????????0?0????????????????

Fugusuchus_hejiapanensis ??000-00?0??100210{2 3}1?10????????????????0?????00-?{1 2}??00--110000200??0?010002?0?0?-000?011??00000010010011??00000?200?-0-??100010001000010?01??00001000???????????-0-2-1010?011?????0??????????01???????????????100011111100-01100011110001?01111001010101??1??110??????????????????????????????????????????10100?10?0?????????????????0???????????????????????????????????????????????????????????????????????????????????????????????????????0?00??100????????????00????????????????????????????????????????????????????????????????????????????????????????????????????????????????????????????????????????????????????0??0????????????????

Sarmatosuchus_otschevi 2?000-0?0001?????0{2 3}???????1220010?0201??110???????????????????????????0???{1 2}?????????????????00??00?{1 2}0??1???????0??0????00?????0???????10?01?00000{1 2}0001???????????????1?1???????12000110????0001??00?000002?????0?0111?1000-01?00111110000?1111000?0?0000??1??110???0{0 1}???0211001??????000??????????????????10???21000?1001110111101-{1 2}??1001011000(0 1)0100000-01????0001??110?00?-??????????????0????100010000000010000-??????????????????????????01000?????????????????????????????????????????????????????????????????????????????????????????????????????????????????????????????????????????????????????????????????????????010000??????0????

Guchengosuchus_shiguaiensis ??001000?00?101???{2 3}??1{0 1}????????????2??20?????0??010010-011-??020?10001{0 1}0001?0000000?1??????????????????????????0??00-0-00{0 1}?0????-?????????????????????????????00-0-2-10110{0 1}?1??????????????1-0???0----00?2?????0?1?11?10??-?????????????????????????????0??1?1100???????????????????????0?1??20?????0?1???1010021000?10011?111?100-???1????{1 2}10001010?010-?11{1 2}1????????10200?-?00???????????????1?00?01?00?????????????????????1?????????011??0?0???101??????????????????????????????????????????????????????????????????????????????????????????????????????????????????????????????????????????????????????????????????00?01010???0????????

Cuyosuchus_huenei ???0?????????????0??????????????????????????????????????????????????????????????????????????????10?20??0??????????????????????????????????????????????????????????????????????????????????????????????????????1??????????????????????????????????????????????????????????????????????????????????????????????????????1001111110101-????????1?0001?10??00-?1???10010011?01001-??00?01111?10(0 1)0??{0 1}1?0?00?0????0010000-???????????10000000?10111-0100???0?????????????????0?0{0 1}0110110100100000021?0001?00?0?{1 2 3}110?000001?0???????0{0 1 2}11100{0 1 2}001000001001111?????????????????????????????????????????????????????????10?????????{1 2}????10?????00?00000?

GHG_7433MI 2?000-01????1????????????????????????????????????????????????130?100?1?0001{1 2}?????0?????????????????????????????0{0 1}?0000-??100?10101?0??????????????????????????00111??10?1?????????????????????????????000??????????????????????????????????????????????????????????????0??{0 1}00?1??????000??????????????????4???02100011?01???????????????????????1?1?????????????010??1??1?0??10???????????????0??????????????????????????????01?00000????121-010????01?1?????00??????????????????????00?0?021?000?000?00????????????????????????????001????????????01???????????????????????????????01?03?1010?????????????0?---------0??0?0?0?????0???????0

Garjainia_prima 2?000001000110021021210120111010000200201100110-011011--1200?1300100111000120000100010011-020101100100100000010?001000-1-100010101000110001000000100010020022-?0111??10110{1 2}?100120000010???(0 1)00???0022000020111100011111130-010001101100001111100000?0101021111100110010002100011101?00000111030000010?1??1(1 4)0100210?0?1001111111101-1??200101101010110000-?1??100010111001001-?0000010-0??00???{0 1}1100001100100010000-?0???0110??11000001011111-01001??01????????????????000?0100110101100000021200010?0000??100010-0100000000?0202100?0010000010010?101?????????0110000010000?????????01?????????????????????0?---------??000010000?000???0000

Garjainia_madiba_holotype ??00????????????10?????????????????????????????????????????????????????????????????0????????????100100?00????????????0-??10001?10?0001???????????????1??{2 3}??????????????????????????????????????????????????????0?0??1?11??-?????????????????????????????????????????????????????????????????????????????????????????????????????????????????????????????????????????????????????????????????????????????????????????????????????????????????????????????????????????????????????????????????????????????????????????????????????????????????????????????????????????????????????????????????????????????????????????????????????????????????

Garjainia_madiba_combined 2?00??{0 1}100011???10???????01210100?020(0 1)??1100????????12??1{1 2}???????100?1?000???0?????0??????0?????1001001000???10??????????10001?10?0001???????????{0 1}???100{2 3}002????1????1?111??1????0?0?11????????????????????????0?0??1?1130-?1100010?1000011111000?0?0101????111?0??0????021000(0 1)??????0000?11?{2 3}??1?01?11???(1 4)0?0021000?1001110100101-???{1 2}???0110????11????-?1???00?10??1??1001-?0???010-?????1???1?00001100110?00000-???????????11000001011111-01001????????????????????0?0?0000110?01100?001{1 2}1????1000000??100010-010000000000201100?00100000?????????????????????????????????????????????????????????????????????????????0?01000???00?0?00??

Erythrosuchus_africanus 2?000-00000111021021110010111000100200201100?10-0100120-12000020011311200011000000101001??0201011001001(0 1)010001000000-0-1-1000101-1000010000000000210010020122-00-112-1011111100120001000???1-0?000----00020?10100011111041-0211011111000011?1100001?000002011111011001100?00001110100000011103001001011??14010021000?1101111110101-10?2???01101010100000-0111100(0 2)10011102001-00000010-00?0?00(0 1)01100001100110010000-??????????011000001021111-010111101????????040?????000?0110110(0 1)0110000102110001000000??1000100010000100000302100?0010000010011?0011-0000000011000100000022110001?01?0?03000002110???000?00---------??0000100000?01?000000

Shansisuchus_shansisuchus 2?000-00000110121031210??01110001002(0 1)0201100?10-0110120112-00110011011000012000010101000??0{1 2}000?10010010010000001000-0-1-1000101-?000110?00?000002100100{2 3}012??00-102-101001110012?00?10????1-0???0----01?2?????001111?1041-?2110?11110?00?11?1?0??1?0?00?2011111?11001?0020000111010?0000?110300?0010?????4010(0 1)21000?1101?11?1?101-????0010?10?00?10??0?-?1?2?0001011100?001-000?00?0-0??????0?1100001100?10010000-???????????1100000?0101{1 2}1-0?011??01????????030??00?0?0?0110210010100?0102120?0?0?0000??000??000?00002000??2111?0?00100000101???10101000?0000110011000000?????????01?????????021??????0000?---------??00?01000???010??0??0

Shansisuchus_kuyeheensis ???????????????????????120?110001?0??????10???????10???????????????{0 1 3}?1?00??????????????????????????????????????????????????????????????????????????????????????????????????????????????????????????????????????????????????????????????????????????????????????????????????00?1??????000??????????????????{0 2 4}????????0?1?0??11??????????{1 2}???0110?????0??????????0???00?1?0???1???0???????????????1?0000110???0010000-???????????1100000?0101?1-??????????????????????????????????????????????????????????????????????????????????????????????????????????????????????????????????????????????????????????????????????????????010??????????????

Chalishevia_cothurnata 2?000-???00?101???????{0 1}1{1 2}??????????2??20?????1??0?1012?112-??12??10211?0001??00010??1????????????????????????????????????????????????????????????????1?????2??????????????????01?0?0010??????????????????????????????????????????????????????????????????????????????????????????????????????00?0?????????4???021000?110??110?0????????????1?0?00??0??????????0?010????0????????????????????????????????????????????????????????????????????????????????????????????????????????????????????????????????????????????????????????????????????????????????????????????????????????????????????????????????????????????????00?01???????????????

Youngosuchus_sinensis ??000-010001100210{1 2 3}111000001100000011000?200?00-01-012--111000101??001100?120011-0001002??0101011001001000000000101000-??100010110001010000010000100010020022-?010-2-101102-10012000001???????????????????0???10?1??111????????????1???????????????????????????????0010?0200??11????000001110200000??????1{0 2 4}010021000?10011??11?0{0 2}??1???00?0110?0101??000-1112?????????????0????????????????????110{0 1}001101??00100110???????????100000001101{1 2}1-0?0?12100??????????????????????????????????????????????????????????????????????????????????????????????????????????????????????????????????????????????????????????????????0?00???????0???0????

'''Dongusia colorata''' ?????????????????????????????????????????????????????????????????????????????????????????????????????????????????????????????????????????????????????????????????????????????????????????????????????????????????????????????????????????????????????????????????????????????????????????????????????????????????????1?0??11110???????????????????????????????0??2000?1010??????????????????????????????????????????????????????????????????????????????????????????????????????????????????????????????????????????????????????????????????????????????????????????????????????????????????????????????????????????????????11??????????????

Uralosaurus_holotype ???????????????????????????????????????????????????????????????????????????????????????????????????????????????????????????????????????????????????????????????????????????????????????????1-0???0----0002??????????????????????????????????????????????????????????????????????????????????????????????????????????????????????????????????????????????????????????????????????????????????????????????????????????????????????????????????????????????????????????????????????????????????????????????????????????????????????????????????????????????????????????????????????????????????????????????????????????????????????????????????

Uralosaurus_combined 2?????????????????????????????????????????????????????????????????????????1????????????????????????????????????????????????????????????????????????????????????????????????????????????????1-0???0----0002?????????????????????????????????????????????????????????0????0210001??????000??????????????????4????21??????????????????????????????????????????????????????????????????????????????????????????????????????????????????????????????????????????????????????????????????????????????????????????????????????????????????????????????????????????????????????????????????????????????????????????????????????????0????????????????

Vancleavea_campi 2000100-00110-0100101?110011000001020000?1???00-0--00---11001--0-1?010000?100000-0------??00000-01010000?0000000-?02-0-??2--0100--000010-01110000000-10020022-00-0-??00?002-1011001000?????????????????????????0?1??111???100??0010110000??????????????????1??1??1?00???03200?010-{1 2}10000001100???001?????14001-21000?1?0????00?00--???1????1???10?10???0-1????1?3100?100{0 1}00?-??0?00????????00?21100001100010?????????????????000?00000110121-1?0000100???????2?101????000?00-001010010??0????????10?0?00210000000010000----00111100010100000100???101010001000011000000000012110??1?0?30(3 4)0????0001?????????30-101?001?120?00100????00??00000

Asperoris_mnyama 2?001000?0011?0?1????1{0 1}0001100000?0200201200?0????-01{0 1}?-110??0??011{0 3}00?000??00?000?01?????????????0???????00?010{0 1}002-0-00??0????-?????????????????????????????00-0-{1 2}?10?01{0 1}-??????????????????????????????????????????????????????????????????????????????????????????????????????????????????????????????4?????1??0?????????????????????????????????????????????????????????????????????????????????????????????????????????????????????????????????????????????????????????????????????????????????????????????????????????????????????????????????????????????????????????????????????????????????????????????????????0?0????????????????

Euparkeria_capensis 2000(0 1)0010000100110112100(0 2)0110100010200001200100-000(0 1)010-110000200100001000110000000010111-0000000001001000000000110100-??100010000000010000010010000010020022-0100-2-10100011001200000001020011110020001020100101010011040-00000110110000?110100010101000101?11001?0010001100001102?0000002102011101010??1401002100011001011110101-10?10000100?010100000-010211(0 1)010001??1001-00000010-10??(0 1)00?01100001100?00010000-10????000001(0 1)00000?110121-000001100????????0(2 3)01?0??00??0110110100100001011?00020000001(1 2)0010000010000200000(1 2)11000200100000001?0?0211-0000000011111001000012110001?012030(1 2)00(0 1)?010?0?0000{0 1}020-101010110000001000001000000000

Dorosuchus_neoetus ???????????????????????????????????????????????????????????????????????????????????????????????????????????????????????????????????????????????????????????????????????????????????????????????????????????????0111?1?1040-01?0?111?10000?110?000???00000?1?11100??????????????????????????????????????1?????????????1???????????????????????????????????????????????????????????00???1???????????????????????????????????????????????????????????????????????????????0?0101101101001?????????????????????00100000100002000?0211101200100000????????????????????????????????????????????????????????????????????????????????????????1???00??

Proterochampsa_barrionuevoi ?21(0 1)202000111001?0311010011210010?021000?100100-?2-0?12-1100100001?0?020001{1 2}000000001000?-0000000001000000000000(0 1)100-0-??2--0100-0000010001000000100010020022-00-0-2-101002-10?1?0?00000102000?01042-10102?101101011111040-0?01?110111001?0?20001??100000????????1?001?00?0001?10-110?000?0-031000000?0???40100{1 2}10?0?1000?0000?00--{0 1}?021000110??0?10?000-11?{1 2}??1000?1100000?-10000??0-1????0??00?0?001100??00100?0-??????????01000000?1101{1 2}1-0-???11??????????????????0??????????????00??????????????????1001?????1?0002?00?01121?02100000011??10??2?1-00?0??0???????????????????????1{2 3}?????????????????0000----------000??01???0????0???000

Proterochampsa_nodosa 2?1120200011100110312010?11?10?1??021000?100?00-?????0--110010000??0?0200012000100-01000??000000000?0?00?000000??100-0-?????0100-?000010001?00?00?0?0100{2 3}0022-?0-????10100??1001?00001001?2??0???0????0????????0???1111????????????????????????????????????????????001000?000?????1??0?00?0-031??0000?0???40100?00?0????????????????????????????????????????????????????????????????????????????????????????????????????????????????????????????????????????????????????????????????????????????????????????????????????????????????????????????????????????????????????????????????????????????????????????????????????0??0????????????????

Tropidosuchus_romeri ?000202100111(0 1)0010{1 2}1?0100???00010102?000?100?00-11-0000-110010100??0?0100?1?0001?0001010??0000011012000000000000010000-??2--01000010101010(0 1)000000100110121022-0?00-2-10100??1001(2 3)0000000???00011{0 1}002210102?????0?0??111040?0??1?110111000?0?2?000??1?000???????????0011?0?201?0??????0?01?2??3????110?0???{0 2 4}0100{1 2}1000?1001?0000000--100200?0110?00?100010-11???110100?1??0011-10?00010-10{1 2}1000??11000011??0?00000?0-?0?????????10??000?1?0111-0?0??11??????????????????000?0110????00100001011300010?0?001?0010000??00002000?01111?02100000011011101211-0001000011101000000012110001?01201011100000???2--00010-101-0??-0?0?00?0??00?0?000?000

Cerritosaurus_binsfeldi ??0020200011110110{1 2 3}11010??1110?1?10210?0?1???00-12?0011-110?10100??000100?1?0001?0001002??000000100100000000000001??-0-??2--0100-010101010??110001001100310?2-0?-0-2-1010???10013000?00????????????????????????0???1111??????????????????????????????????????????????11???2????????????00?11?3???001??????{0 2 4}010(0 1)01000???????????????1???????????000?????0?????????????1??0?0???0????????????????????????????????????????????????????????????????????????????????????????????????????????????????????????????????????????????????????????????????????????????????????????????????????????????????????????????10-???-1??-??0??0?0??????????????

Gualosuchus_reigi 2?0021210011110210{2 3}1002000121001010210001100?00-11-000--110010300100101000110001-0-01000??01000010(0 1)1000000000000010000-002--01000000111010(0 1)?11000100110021022-0010-2-101002-10012000000010200011?002210102011110?01?1110?0-?0???110111000?0?2?0????1?000???????????0012000201?010-1100001?1003000011000??14010001000?1001?0000000--10020000110?000100000-11?2?0(0 1)0100?1??0001-100??????????????0100{0 1}00110001(0 1)010010-???????????10?0100?1?0121-????????????????????????????????????????00?????????????0?001200100000100002001?0{1 2}1110121000000110110?1?????????????????????0????????????1???????????????????0?10-011-100-0?0000?0??0??-???0?00?

Chanaresuchus_bonapartei 21(0 1)0212(0 1)0011100210310020001(2 3)1001010210001100100-1(1 2)-000--110010100100101000210001-0-01002?-0100001001000000000000010200-??2--010000001(0 1)10100011000100110121022-0010-2-101002-10012000000010200001(0 1)002210002?101101011111020-00?1011011100000121000??1?000???1?????1?00110002010010-1100001?110300(0 1)011000??14010011000?100001100000--100200(0 1)0110?00?100000-11?2?(0 1)0010011000001-10000110-102100?0?1101001100010010010-??????????010001000110121-?????1???????????????????0001011010010010000101130001000000(1 2)300100000100002001001111?021000000110110?1211-00000000111010000000?2111?0110120201110?000??22--00010-011-000-00000010000??010000000

Pseudochampsa_ischigualastensis ?11021210011110?10{1 2 3}1?020001210010?02100??10??00-?2-000--110??0100??0101000{2 3}20001?0-0???0??0100001001000000000000010000-??2--010000?01110101000000100110121022-?010-2-101?0??1001?00000001??000???002210102????1?????1110???0??1?110111000?0?1?000????000?????1?????00???0??0????????0000??0-???????10?0??14010001000??00??0000??{0 1}??1??{1 2}?????10?0?0?0?????11?2?11010??10000?1-100?0????1011?0000000?00?100??00000?0-1?????????????000001????????????????????????????????????1???0??????????011?000??????????0????????0002000????????21??00?011?110?1????0000??0?11{0 1}????0????????????101201?2110?000??????00010-01?-100-000??0????0?1??0?0????

Rhadinosuchus_gracilis ??0021??00111?0???????2???1?1??1?10????0?{1 2}0??0??11??02?-110??0100??0?0?00???0001?00?1??2???{0 1}?????????????????????????????????????????????????????????10?{2 3}1?{1 2}????????????????????????????????????????????????????????1?1?40???0???????????????????????????????1?????0????00211?0??????000?????????????????14??0(0 1)21000?1??????????????????????????????????????{1 2}1????????????????????????????????0?????????????????????????????????????????????????????????????????????????????????????????????????????????????????????????????????????????????????????????????????????????????????????01?????????????????????10-01??000-??0??01???????????????

Archeopelta_arborensis ???????????????????????????????????????????????????????????????????????????????????????????????????????????????????????????????????????????????????????????????????????????????????????????????????????????????0111?0??041-00?1?110?10100?110?000???0000?????110?????????????????????????????????????????????????????1?0???????00--???????????????????????????00000?1????001-1??1?????0?????????????????????????????????????????1010101???????????????????????????????0??111???0?00??00?????????????0?00??00100001100002000?02011?0?101????????????????????????????????????????????????????????????????????{2 3}110110210???????????0????????0??

Tarjadia_ruthae ????20?1???????????????????????????????????????????????????????????????????????????????????????????????????????0??0000-0?100??????????????????????????????????001????101????1??????????????????????????????????010????????????????????????????????????????????????????????????????????????10?3????????0??????????????1?0??10000?????????????????????????????????010??20000???????????????????????????????????????????????????????????????????????????????????????????????????????????????????????????????????????????0020000???????????????????????????????????????????????????????????????????????????????{2 3}1101??2101??????1100????????????

Jaxtasuchus_salomoni {0 1}??020??????1????0????{0 1}??????????????????????0???100-1??1100?000010000?0002????????????????????????????????????????????????????????0?????????????????????????????????????????????????????????0???0????0????????0?11???????-?01??????110??????????????????????????????1???????????????????1???????0?1??????40?0001100?1?01??????0{0 2}??1??{0 1}???1{1 2}10000010?110-???{1 2}?1?(2 3)10?????????-11?????????????0?????????????????????????????????10001010110111-0-??0?000???????2?300??????????????????????????????????????2300100?00??0??2000?0?101002?0?00?011?00001????????????????????????????0????0120???00??00100?1?0?0?311201?01011000?0?0??0??0?????0??

Doswellia_kaltenbachi 2?1020{0 1}0????????-13???????????????????????????0-???????????????0??????0000{3 4}????????0??????11?0??00--0--0--?????0???0-0-??2---100-0-0-0100-1110000-0001----022-00-0-??001002-1??120000000???000???0002101120101101111111020-0011?110011000?010?001??1?000???????????000-?00200001?????0000120001000010001114???0?0????1001?00000101-1???10?01100000110110-10?{1 2}11(1 2)010012000000-11011110-??3000?1{0 1}?????????????????????00000012??????????????????????????????????????????000?111010000010000101130001000000???01???????0???????011?1??????????????????????????????????????????????????????????????????????????31120?1(0 2)101?0???010000?1?0?0?00??

Parasuchus_angustifrons ??10201001111000001??000001?0??-00--1-001{0 1 2}0?100-1?-0?20-1101002001020020002?0000-0001002??10000110020000110000101???-0-????00100-0101010100011000100110020022-0?-0-2-101002-1011210001001131-12011----01020111101010111040-???1?110110000?0?0?1{1 2}-??1?000?????????1??????????????????????????????????????????????????????????????????????????????????????????????????????????????????????????????????????????????????????????????????????????????????????????????????????????????????????????????????????????????????????????????????????????????????????????????????????????????????????????????????????????????????????0???????????????????

Parasuchus_hislopi 2110201001111?00001210000014000-00--1-001000100-12-0020-110100200102002000210000-0001002??10?00?10020000110000100100-0-001010100-0101010100?11000100110020022-00-????101002-1011210000001131-12??????????????11010101110??-1111?1111100100010001-???0001???1011?01?0010?10001?110-2?10000?11010?01010?1??140?1-(0 2)1000?1001011110031(0 1)10010000110000?10000?-11?2?10010111000001-10000010-1???????00000001100?00100000?1000101120010101010111111-{1 2}?000{0 1}100????????0?01100100010110110200100001021?00?10???001100100100100002000?01101001001000101001200111-000000001111110011012211000100120311001011???201002021020011010000??01000001000000000

Nicrosaurus_kapffi 2?10101001111000001230000012000-00--0-001000?00-11-000--110100200102002000220?10-0-(0 1)1000??100-0100020000110000100100-0-??1000100-1100010000011001000011020022-00-0-(1 2)1101002-1011210000001131-12011----00020111101010111040-01??0111110000?000?0{1 2}-??1?000?????11????01111100010110-2010000010120110010?1??04011-(1 2)(0 1)000?1?01?111100310{1 2}??1???0110000?1??000-1????0??1011?10000?-1????????1??02?????????????????????????00?1011??010001001111111-{1 2}?000????????????????????????????????????????????????????????00100001100002000?0?101?0?00100000101?210?????????????????????????????????????????????????????????????????????0?0010001?100?00000?

Smilosuchus_spp. ??1010100111100000122000001(2 3)000-00--0-001000?00-11-0-0--110000100102002000{2 3}10000-0-11000??100-01000(1 2)0010110000000100-0-001000100-1100010000011011100011020022-00-0-2-101002-10112100?0001131-12011----00020111101010111040-0101?112010000?010001-0110000?100?11??1?01111100010110-20100000110200010101?1?140?1-20100?1101011110031(0 1)1?01001011000001??000-11?2100010111100001-10010010-1???110?01000001100100111000-?00010?1??010001010111111-1?000?100????????????????000?011011010010000102120001000000??0010000010000200000110100?00100000001?210111-000000001111110011012211??01?0??0?1????00?????????2?210000?0000?00?0010000?100??00000

Ornithosuchus_longidens 2?00100100011002?011110001121100000210101300?00-01-0031-1101001002?000100001000000001101???20000000{1 2}0010000-0000010000-??100010111000010001011000100110030022-0100-2-101002-10?1{2 3}00000??1131-110?1----0002011110?0?0111??????????????????????????????????????????1?001?01?201?0??????0000?1?030?1???0?????4010021000?100??11110031?????????1?0?00?10??0?-11????10101?1?0?00?-1?0?10????0??010?01001001100?10011011110?????1?001??010101?0121-000??1100????????00??0???10010110110100100211011000022?0?00??00101100100102000?0110?0???11000????1?2?02????0??????112?110??00???????????1?0???00???????????02020-1001100(0 1)000?001000001?10000000

Riojasuchus_tenuisceps 2?00?-000001110210112100011221?0000210101300?00-01-0?31-1101000002?000100001001000001101??02000000010010000000002100-0-??100010000000010000011000100010030022-01-0-2-001002-1001200000001131-110{0 1}1----00020?1110?010111040-???1?111110000?01001?????0000???1?1???1?001201321??010-2100000010030?10010?11114010021000?1000000000031010020010110000?100000-1??{1 2}?00000111000001-100011???1??0?0???100?00?100?11?110111??????????011001010110121-100111??101??????010?????1001011(0 2)(2 3)102001001110110000?20?????201100100100102000?0(1 2)100002111000100011210211-1000010011211101100122110011?0120302000001000?{0 1}?002020-000120000?0?0010000??010?00000

Nundasuchus_songeaensis 2??????????????????????????????????????????????????????????????????????????????????????????????????????????????????????????????????????????????????????????????????????????????????????????00?????3--001?2?????????????????????????????????????????????????????????0????0{0 1}11100??????000??21?20?0?????????4????21000?1?01?110100311?00????01?0?0??10??0?-11????0(0 3)10??110?001-1?00?010-1?????0101?00011100?100100110?01010??1??11001010110111-??????????????????????????1??????????????0??10112000???????1100101?0010000200000210100201100010001?200111-0000000011111100110122???01??012031?00?0110???0?0?2?20-10100011?0???01000???0??00?0?0

Turfanosuchus_dabanensis ?0001001?001100010?1{1 2}?0000110100000210201100?00-00?0020-110000001??0011000{1 2}{1 2}001001101111??010001100100100?000000111100-??100010110011010010?11001000010?20??2-0010-??10?00??100120000000???001???13--?01?2????????1?1??040-2?01?110?10000?1100100???0000?10??110???0010000101?0?10??00000111020?0001??????40??021000?10010?11100310???{1 2}000??10000?10??10-?1??????20??1?01011-???00010-1???0000??????0???1?????????????????????10000000110111-100????0?????????????????00?1021020010?1?01010110000???????12001?0100?00002000?0110100?01100010101????111-10010?0011221110{1 2}101?????????01?0???????010?????????20-101?0011??0??0100000?01?0000?0

Gracilisuchus_stipanicicorum 2?001(0 1)11000110001011{1 2}0000011010000021020?200?00-00-0020-11(0 1)00010120001100021000001101111??01000110-10-1031000000110000-??10001111100101100-011001-00110020022-0110-2-10100{1 2}?100130100000???1-1?0?1----01020110101010111040-?(0 1)???010???????1?001{1 2}-????000?????1???1?001000?10100?10??0000011102000001010??14010(0 1)21000?1?01?11110031010?10?10110102?100010-11?11100200?1001011-100000?0-1?100000{0 1}???{0 1}??1????????????????????????10000001110121-??????????????????????????001021011000010000102100001?00000?000100100?00002000000111002{1 2}?100010???1100111-1000000011221110210122???01??0120302000001000?0?002020-10100011??0000100?????100??000

Aetosauroides_scagliai 2?0011000001100??????100001{2 3 4}0?0?00?21000?100?0??0{0 1}-0030-1110001?1??000?000?{1 2}0001-10?110{1 2}???????1??????????000000{0 1}100-0-??{0 1}???0??-?????????????????????????????00-????11?0????????????????????1???1??-?01????????????????????0????10?1000????????????????????????????21???01?10?10-{1 2}??100?????????????????14???0210???100?01111002101?0100?0100?00?101000-11?{1 2}?(0 1)(0 1)020011000001-10000010-10{1 2 3}????1?1?0000?100?10??00????01010000?01(0 1)000010111111-100111100????????0???????01010310000100100001011100010000002200100100100002000002101002011000101011210111-1000000011221110111122110011?0120302000001000???????21020012000120??01000??1000000000

Batrachotomus_kupferzellensis 2?001001?0011002102?2100101101010?0110001200?10-0{0 1}00130-1110001?02100110001?0011-11010011-01000?10010?00?00000?0101000-001000101100110100?0011000?01010020?22-0010-2-101012-1?012000000??1?1-?1???----000201101011101?1040-11101110?100100110012-???0000121?11111??0010001001011102100000111020010??011??140??021000?1001111110031(0 1)???200101100000100000-11?{1 2}1(0 1)0020001101001-?00010?0-1???31?001?00001101010?100110??????????010000010110111-11011??00????????????????010?0211110101110?010210010?001000??0010110010000200000110100?01100010001?21011??1??????111221100?111?????????01?0???????12000?????2?20-10011011??000010000?100?000000

Prestosuchus_chiniquensis 200010010001110210212100001(0 1)0100000210001200?00-01-0020-11010010121001100012001100001001??0100010001001001000000000000-??100010110000110110011000001010020022-0010-2-101012-100110000000???1-110?1----01020?1?101010111040-11(0 1)1?110110000?110012-0010000??01?111?1?0010002000011102(0 1)00000111020010010111114010021000?1001?111100310100?00001100000100000-11?{1 2}100020001100001-100000?0-1??001?0011000011010100100110?0101011???10000010110111-1101???00????????????????0101021?110201110001021001110010101200101100100002000?01101002011000100011210111-10000000112211002111221100?1?0120?0?00?01{1 2}????0?0?2?20-1011001100000010000??00?000000

Dimorphodon_macronyx ?{1 2}00??0?0001100210?22?0000-3000001111000?200?00-11-0?20-111000100??000200?0??00?0??01002???20000000-------???????1??????????0000??00?010?01?1-00000??10?2-?1?????????????????001???0?0?????????????????????????????????????????????????????????????????????????????00??0001?1001????1000??????0??????????0{0 2 4}110(0 1)01000?1?0???????00--???1?????????????????-11????(0 1)010??1?0????-10????????????01??0001000100?10?21010-??????????010?110101101201???????????????0?0-0101110?10031?10021010000112??00?10???000?01100100?0000----10111?00300?0-00-??110?0-11-?0111???00-------??1?2111101?1?10403000111100?0?00010----------000?(0 1)01???????01???1?1

Lagerpeton_chanarensis ?????????????????????????????????????????????????????????????????????????????????????????????????????????????????????????????????????????????????????????????????????????????????????????????????????????????????????????????????????????????????????????????????????????????????????????????????????????????????????1?0????????0--????????????????????????????(1 3)?????????????1??00010-1???0???????????????????????????????????????????????????????????????????????????0011021?10020010000001130001000?00??01110101100002100?01111102101000001011101-11-1011100-00-------2-1-21111110112000000200000022--000?????????????????????????00?????1

Marasuchus_lilloensis {0 1}???????????10???????????????????????????????????{0 1}??????11?0??0????0?0?0001????????????????????????????????????????????????????????????0????10?????????????????????????????????12000?0?????????????????????????0?01?????41-1101?110???000?010002-?0?00{0 1}0?????1110?????????????????????????????????????????4010021??0?100101000000--10020010110?000100100-1????(1 2)10100????0011-1?000010-1011?000?001?001100?100010110??????????01?1?1010110121-1001?{1 2 3}10??????????????????011021210020011011001100001100?001200110100000112000?011100021000100110110?0211-011010001120000010011211111111120302000000000?2--00?0----------0??0?0?0??01?010??0??0

Lewisuchus_admixtus {0 1}?00????????11??00{1 2}???0?0????????????????????0???{0 1}-002??110??0000{1 2}0000?0002????????0????????00??10010-0031??????????????????0??01?001110????10000000?1002002???????????????????120000000???00????05-2-0102101?10111?111040-100111101100000010002-00110000??1?11101?????????????????????????????????????1??40?0(0 1)21000?1001?1111000--11010000110?00?100??0-110??12010001??0011-10???????????0????00110011000100110111???????????10?010?01{1 2}0?21-???????????????????????????????????????????????????????????????????????????????????????10?01001???????????????????????????????????????????????????????????????10-000-10?-0?0000?0??0??????1111?

Asilisaurus_kongwe 0?0?????????1?????????0?????????????????????????????????????????????????????0????0?01????????????????????????00????????????????????????????????????????????????????????????????1?0?????????????????????????????????????????????????????????????????????????????????0????0????0???????100??21????1????00???1????20001?1001?1111000--?1?2????1100000?0?1?0-1??2?1?0100?11000?1-1???01????????00???01?001100?10?110110?????????????0010101?0121-?????????????????????????000?0{1 2 3}?2100?00110?11011??0??1?1?0???0010110001111200010110??0?10001001111????111-0110100011210000100112???????1???????0?1??????????100----------?????01000?0?01?111110

Silesaurus_opolensis 00000-11?00?10??002????0001100000??2100?1200?0???1-103??1????00002100010001???00-0?011?2??1{1 2}000?10010??0??0?0000110100-00???0?0???011010?00??0010100?1002102??0010-2-111002-1??12000000????1-???{0 1}0----0102?01??0?11?111040-1?011110110000?01000000011(0 1)001201011101?00100010001111?1101000121010?1001000???10?0220111?1001?1111000--110(1 2)(0 1)0101100000100100-11?2110010001100001-10001110-10100111{0 1}0?110011000100110111??????????010001000110121-000003100????????????????000{1 2}02121021001102100(1 2)1000021110001?001011(0 1)0011112000101110002101110011111101211-0211100000-------20112???1???1021?020001000???2--0100----------000000110000?000111110

Heterodontosaurus_tucki 00000-02?00110000020210101110001000200011300100-01-00(2 3)3-110000000200101000110000-1001012??0100011000001000000000110010-002--110010001010000011001000011020022-00-0-2-111002-11?1200001001?31-???1?----010210???0111?111041-1001?110210000?010002-??11000???1?111?1?02100021001010-101001012012111001000??0411012(0 1)110?1001?0000000--11?00010100?10?100000-11??0?1?10111?00001-100?20???10?0000121?11001100?10?0??10-11-------0110?110?0110121-1001011001-100110101101121011031011001011020112--00-32?1?0011011?0?0??-?102110?0?1?0?022001110?1101101-11-?2111?0-00-------201?2111101010213020001000??22--0000----------00000011??00?000??-101

Herrerasaurus_ischigualastensis 2?000-0100011011002121001011000100020000?200?00-01001200110000000{1 2}130010002?0000-0001102??01000010010-0030000000?10010-002--0{0 1}00110010100000(0 1)0000000010020022-0010-2-101002-10?130000000???1-1???0----01?210?0101110111040-?0???111110000?010?12-????000???1?????1?001100(1 2)0011011010000001(1 2)1020010010?(0 1)1114010021000?1?01?1111002001102001011001(0 1)0100100-11?2?(0 1)00100?1100001-10000010-101??01000?11000100011?110111??????????110011011120121-1001011001-1111001(1 2)11111210110212111200110(1 2)10121001112110103?01110100012112010?01110(0 1)0220(0 1)011011101101211-0211100000-------2010211110101121302000110000?1000000----------000?001100?1?00010-100

Yarasuchus_deccanensis ?????????????????????????????????????????????????????????????????????????????????????????????????????????????????????????????????????????????????????????????????????????????????????????????????????????????????????????????????????????????????????????????????????????????????????????????????????????????????????100111111000--{1 2}??1???(0 1)2100110100010-?1???10010111001001-1???0010-1???30???1?0?00??00????00000-???????????10000000110111-00010????????????????????00000??21100001?010001100002011000210010100010010200000112100200100000???????11???0?????0011010001001??????????????????????????????????????????-??????11001??00?1?1??0

Dongusuchus_efremovi ??????????????????????????????????????????????????????????????????????????????????????????????????????????????????????????????????????????????????????????????????????????????????????????????????????????????????????????????????????????????????????????????????????????????????????????????????????????????????????????????????????????????????????????????????????????????????????????????????????????????????????????????????????????????????????????????????????????????????????????????????????????0010100010010200000112100?????????????????????0????????????????????????????????????????????????????????????????????????????????1??

Teleocrater_combined 2?000-0?????100?0???????0????????????????????????{0 1}?0020-1?0??010021000?0001????????????????????????????????????0?10110-00??????????????????????????????????????????????????????130?0100????????????????????????0?0??1?10???110???10?10000????????????????????1????????????????????????????????????????????4????21????100111111000--??01001?210010010?010-1???11001?101101001-1???00?0-1010?00????01?01100010??????????????????10000000110111-00010??00????????????????000?0212????0010??0?????????011000??0010100010010200000112100200100000101110011???0?????0011010001001?????????????????????????????????????????????00?01100?0?0??111110

Spondylosoma_absconditum ?????????????????????????????????????????????????????????????????????????????????????????????????????????????????????????????????????????????????????????????????????????????????????????????????????????????????????????????????????????????????????????????????????????????????????????????????????????????????????1?01?1111000--??????????0??1?10??10-1????1??100?110???1?1??0101??1????????1????011000??????????????????????00101?????????????????????????????????????????????????0?01?{1 2}1???????????????????????????????0?11100?????????????????????????????????????????????????????????????????????????????????????????1100??????11????

;

END;

BEGIN NOTES;

TEXT TAXA = Untitled_Taxa_Block TAXON = 32 TEXT = 'Scorings of added characters based on UMZC and BPI specimens (MDE, 12 September 2012).';

TEXT TAXA = Untitled_Taxa_Block TAXON = 35 TEXT = 'The scorings were based on the description of Tatarinov (1978), descrption and figures of Benton and Allen (1997) and personal observations on the specimen. The figures of Tatarinov (1978) were not considered because of discrepancies with the photographs of Benton and Allen (1997) and strange morphology when compared with other basal archosauromorphs (MDE, 2 April 2015).';

TEXT TAXA = Untitled_Taxa_Block TAXON = 46 TEXT = 'Include the holotype PIN 1025 348 and the referred specimen (ex Vonhuenia fredericki referred specimen) PIN 1025 14 (MDE, 4 April 2014).';

TEXT TAXA = Untitled_Taxa_Block TAXON = 47 TEXT = 'I scored the holotye and the unambiguously referred vertebra PIN 1025 419 (MDE, 20 October 2014).';

TEXT TAXA = Untitled_Taxa_Block TAXON = 48 TEXT = 'Include holotype PIN 2252 381 and referred specimens PIN 3200 212, 3200 217, 3200 472, 2243 167, 2252 384 and 2252 386 (MDE, 4 April 2014).';

TEXT TAXA = Untitled_Taxa_Block TAXON = 51 TEXT = 'Chasmatosuchus magnus plus the holotype of Gamosaurus PIN 3361 13 and referred specimens PIN 3361 14, 3361 94, 3361 183, 3361 213 and 3361 214 (MDE, 9 September 2014).';

TEXT TAXA = Untitled_Taxa_Block TAXON = 53 TEXT = 'The two published vertebrae are reinterpreted as middle dorsals (MDE, 20 June 2015).';

TEXT TAXA = Untitled_Taxa_Block TAXON = 75 TEXT = 'The tentatively referred specimens of Sookias et al. (2014) (partial pterygoid and lower jaw) were not included here because there is no possitive evidence for this referral. Sookias et al. (2014) stated that "These referrals are, however, plausible because no other archosauromorphs have been found at the locality", but this argument is circular (MDE, 18 August 2014).';

TEXT TAXA = Untitled_Taxa_Block TAXON = 90 TEXT = 'Nicrosaurus kapffi: NHMUK R38036, 38037, 42743, 42744, 42745, multiple SMNS specimens (SMNS specimens with ambiguous identification were not considered), Huene 1923, Hungerbühler 1998, 2000 (MDE, 19 August 2014).';

TEXT TAXA = Untitled_Taxa_Block TAXON = 91 TEXT = 'UCMP 26699, UCMP 27200, MCZ 1029, USNM 18313 and Camp (1930) (MDE, 6 May 2014).';

TEXT TAXA = Untitled_Taxa_Block TAXON = 92 TEXT = 'Huene 1914, Walker 1964, Sereno 1991 (MDE, 20 August 2014).';

TEXT TAXA = Untitled_Taxa_Block TAXON = 108 TEXT = 'The specimens that were not associated with the holotype and do not possess overlapping features with bones collected in the type excavation site are not included in thes scorings. These non-associated bones usually are of bigger animals and sometimes possess non-congruent characters with those expected for the animal of the type excavation site. The very poorly preserved maxilla and supposed attached premaxilla were not included because they seem to belong to a bigger animal than the holotype. It was not included here the premaxilla-maxilla (ISI R334/1), right jugal (reinterpreted as an indeterminate bone, ISI R334/3), left quadrate-quadratojugal (reinterpreted as an indeterminate bone, ISI R334/4), right squamosal (reinterpreted as a right postorbital, ISI R334/5), right pterygoid (the indentification of this bone as a pterygoid is very tentative, ISI R334/7), and left pterygoid (ISI R334/6) (MDE, 16 March 2015).';

TEXT TAXA = Untitled_Taxa_Block TAXON = 109 TEXT = 'I scored only the holotype and unambiguously referred femora (MDE, 20 October 2014).';

TEXT TAXON = 44 CHARACTER = 1 TEXT = 'The absence of interdental plates is particularly evident in the anterior end of the right dentary (UTGD 54655) (MDE, 12 March 2014).';

TEXT TAXON = 74 CHARACTER = 1 TEXT = 'Based on UMZC T6921 (MDE, 28 March 2014).';

TEXT TAXON = 90 CHARACTER = 1 TEXT = 'Nicrosaurus kapffi: NHMUK R38036, 38037, 42743, 42744, 42745, Huene 1923, Hungerbühler 1998, 2000; Nicrosaurus sp. Hungerbühler, 1998 (MDE';

TEXT TAXON = 93 CHARACTER = 5 TEXT = 'The dorsal surface of the skull of available specimens is too damaged to determine the state of this character (MDE, 7 May 2014).';

TEXT TAXON = 10 CHARACTER = 8 TEXT = 'The parietals have a median, longitudinal sagital crest, and the surface immediately lateral to it are not depressed as it should be the case in a supratemporal fossa (NMK S 180) (MDE, 13 February 2014).';

TEXT TAXON = 25 CHARACTER = 9 TEXT = 'Dilkes (1998: character 10) scored Mesosuchus has having confluent external nares and the condition is clearly visible in SAM-PK-6536 (MDE, 27 August 2012).';

TEXT TAXON = 78 CHARACTER = 14 TEXT = 'In the holotype the anterior border of the antorbital fenestra is pointed, whereas in PVL 4606 is gently rounded (MDE, 7 July 2014).';

TEXT TAXON = 106 CHARACTER = 15 TEXT = 'The secondary antorbital fenestra occurs is defined by the premaxilla, maxilla and sometimes nasal. In Heterodontosaurus the accesory antorbital fenestra is completely enclosed by the maxilla within the antorbital fossa and, as a result, is not considered homologous to the former (MDE, 27 August, 2014).';

TEXT TAXON = 82 CHARACTER = 16 TEXT = 'The skull is strongly dorsoventrally compressed beause of taphonomic processes and the character cannot be confidently scored (MDE, 18 August 2014). ';

TEXT CHARACTER = 19 TEXT = 'Not_quantified_because_morphological_gaps_seem_to_be_apparent._(DM)';

TEXT TAXON = 10 CHARACTER = 19 TEXT = 'Based on Gottman-Quesada and Sander (2009: fig. 9) (MDE, 12 February 2014).';

TEXT TAXON = 25 CHARACTER = 19 TEXT = 'See discussion of Modesto and Sues (2004: 347) regarding the scoring of this character in Mesosuchus (MDE, 16 September 2012).';

TEXT TAXON = 26 CHARACTER = 19 TEXT = 'See discussion of Modesto and Sues (2004: 347) regarding the scoring of this character in Howesia (MDE, 16 September 2012).';

TEXT TAXON = 35 CHARACTER = 19 TEXT = 'Based on Tatarinov (1978: 508) (MDE, 25 March 2014).';

TEXT TAXON = 38 CHARACTER = 19 TEXT = 'In SAM-PK-K10603 the posttemporal fenestra cannot be observed, but it may be a result of the strong dorsoventral artificial compression suffered by the skull. In RC 846 the posttemporal fenestra is present as a foramen (MDE, 5 September 2014).';

TEXT TAXON = 76 CHARACTER = 19 TEXT = 'Based on Dilkes and Arcucci (2012: 17) (MDE, 13 April 2014).';

TEXT TAXON = 3 CHARACTER = 21 TEXT = 'The skulls that preserve the anterir tip of the maxilla are strongly dorsoventrally compressed and, as a result, the scoring is codified as missing data (MDE, 7 February 2014).';

TEXT TAXON = 6 CHARACTER = 22 TEXT = 'Based on Evans (1980: fig. 1) (MDE, 20 September 2012).';

TEXT TAXON = 10 CHARACTER = 22 TEXT = 'The condition of this character cannot be confidently assessed because the skulls of Protorosaurus are hevely crushed and compressed (MDE, 3 September 2012).';

TEXT TAXON = 12 CHARACTER = 22 TEXT = 'All the skulls of Macrocnemus bessanii are strongly compressed to assess the state of this character (MDE, 30 August 2012).';

TEXT TAXON = 64 CHARACTER = 22 TEXT = 'Based on BP/1/5207 and NHMUK R5392 (MDE, 21 June 2012).';

TEXT TAXON = 64 CHARACTER = 25 TEXT = 'Based on the right side of BP/1/5207 (MDE, 21 March 2014).';

TEXT TAXON = 74 CHARACTER = 25 TEXT = 'SAM-PK-6048 lacks a subnarial foramen, but SAM-PK-6047 possesses a subnarial foramen formed by a notch on the maxilla (MDE, 31 March 2014).';

TEXT TAXON = 95 CHARACTER = 25 TEXT = 'Based on Butler et al. (2014) (MDE, 22 August 2014).';

TEXT TAXON = 3 CHARACTER = 27 TEXT = 'Based on Gow (1975: fig. 1) (MDE, 15 October 2012).';

TEXT TAXON = 55 CHARACTER = 29 TEXT = 'The palatal process of the maxilla is distinctly anteroventrally oriented and in other basal archosauriforms the palatal process of the premaxilla forms a ventrally facing obtuse angle with the alveolar margin of the bone. As a result, the premaxilla of Kalisuchus should have been downturned, resembling the condition in proterosuchids and Sarmatosuchus (MDE, 18 September 2014).';

TEXT TAXON = 64 CHARACTER = 29 TEXT = 'Based on the articulated skull BP/1/5207 (MDE, 21 June 2012).';

TEXT TAXON = 66 CHARACTER = 33 TEXT = 'Based on Gower (2003: 15) (MDE, 25 September 2014).';

TEXT TAXON = 3 CHARACTER = 34 TEXT = 'Based on GHG K 106 (MDE, 7 February 2014).';

TEXT TAXON = 12 CHARACTER = 34 TEXT = 'Based on Peyer (1937: fig. 32) (MDE, 17 February 2014).';

TEXT TAXON = 12 CHARACTER = 35 TEXT = 'Based on Peyer (1937: fig. 32) (MDE, 17 February 2014).';

TEXT TAXON = 3 CHARACTER = 36 TEXT = 'Based on BP/1/2871 (MDE, 27 March 2015).';

TEXT TAXON = 19 CHARACTER = 36 TEXT = 'The dorsolateral process of the premaxilla is well extended beyond the posteiror margin of the external naris (Spielmann et al. 2008: fig. 20). Accordingly, the scoring of this character was changed from (?) to (1) (MDE, 4 September 2012).';

TEXT TAXON = 35 CHARACTER = 36 TEXT = 'Based on Tatarinov (1978: 508) (MDE, 2 April 2015).';

TEXT TAXON = 73 CHARACTER = 37 TEXT = 'The posterior margin of the base of the postnarial process is broken off, but the facet for its reception on the maxilla shows that it was plate-like (MDE, 1 October 2014).';

TEXT TAXON = 26 CHARACTER = 40 TEXT = 'Based on Dilkes (1995: 669) (MDE, 10 September 2012).';

TEXT TAXON = 3 CHARACTER = 42 TEXT = 'Based on Broom (1922:273) and Gow (1975) (MDE,12 February 2014).';

TEXT TAXON = 6 CHARACTER = 42 TEXT = 'Based on Evans (1980: 223) (MDE, 20 September 2012).';

TEXT TAXON = 10 CHARACTER = 42 TEXT = 'Based on Gottman-Quesada and Sander (2009: 140) (MDE, 3 September 2012).';

TEXT TAXON = 25 CHARACTER = 42 TEXT = 'Mesosuchus has two premaxillary teeth (Dilkes, 1998: 511) (MDE, 27 August 2012).';

TEXT TAXON = 35 CHARACTER = 42 TEXT = 'Based on Tatarinov (1978: 510) (MDE, 2 April 2015).';

TEXT TAXON = 78 CHARACTER = 42 TEXT = 'Based on the left premaxilla of PVL 4601 (MDE, 7 July 2014).';

TEXT TAXON = 79 CHARACTER = 42 TEXT = 'Based on Trotteyn et al. (2013) (MDE, 9 July 2014).';

TEXT TAXON = 57 CHARACTER = 44 TEXT = 'The holotype of Sarmatosuchus seems to be non-fully grown specimen because of the presence of an open neurocentral suture in the dorsal centrum (MDE, 9 March 2014).';

TEXT TAXON = 82 CHARACTER = 44 TEXT = 'The premaxillary teeth are lateroventrally oriented, but it may be a result of the strong dorsoventral compressio suffered by the skull (PVSJ 567) (MDE, 28 April 2014).';

TEXT TAXON = 3 CHARACTER = 45 TEXT = 'Based on SAM-PK-K7578 (MDE, 6 September 2014).';

TEXT TAXON = 6 CHARACTER = 45 TEXT = 'The presence of a septomaxilla is equivocal (Evans 1980: 220) (MDE, 20 September 2012).';

TEXT TAXON = 13 CHARACTER = 45 TEXT = 'Following Dilkes (1998) and Nosotti (2007) (MDE, 2 September 2012).';

TEXT TAXON = 19 CHARACTER = 45 TEXT = 'Based on Gregory (1945: 277) (MDE, 25 February 2014).';

TEXT TAXON = 32 CHARACTER = 45 TEXT = 'The septomaxilla of Prolacerta broomi possesses a curved lateral lip (Modesto and Sues, 2004) and, as a result, is not a completely flat sheet of bone. Accordingly, the scording of this character was changed from (2) to (1) (MDE, 11 August 2013).';

TEXT TAXON = 3 CHARACTER = 49 TEXT = 'Based on GHG K 106 (MDE, 7 February 2014).';

TEXT TAXON = 7 CHARACTER = 49 TEXT = 'Based on Evans (1990: 208) (MDE, 4 April 2015).';

TEXT TAXON = 65 CHARACTER = 51 TEXT = 'Based on Wang et al. (2013: fig 2a) (MDE, 27 March 2014).';

TEXT TAXON = 1 CHARACTER = 52 TEXT = 'Based on Reisz (1977: fig. 2) (MDE, 18 September 2012).';

TEXT TAXON = 10 CHARACTER = 52 TEXT = 'Based on Modesto and Sues (2004) and Gottman-Quesada and Sander (2009) (MDE, 16 September 2012).';

TEXT TAXON = 12 CHARACTER = 52 TEXT = 'Based on PIMUZ T4822 (MDE, 16 September 2012).';

TEXT TAXON = 74 CHARACTER = 52 TEXT = 'The anterior maxillary foramen is absent in SAM-PK-13665 but it is present in other specimens (Gow 1970) (MDE, 16 September 2012).';

TEXT TAXON = 91 CHARACTER = 54 TEXT = 'Based on UCMP 27200 and Nesbitt (2011: 96) (MDE, 6 May 2014).';

TEXT TAXON = 12 CHARACTER = 59 TEXT = 'Based on PIMUZ T4355 (MDE, 29 August 2014).';

TEXT TAXON = 96 CHARACTER = 59 TEXT = 'Both character-states are present in MCZ 4117 (MDE, 18 September 2014).';

TEXT TAXON = 13 CHARACTER = 61 TEXT = 'Based on WIld (1973: fig. 5, 6) (MDE, 20 June 2012).';

TEXT TAXON = 39 CHARACTER = 63 TEXT = 'Most of the suture between maxilla and jugal cannot be diserned possibly because of the partial fusion between both bones (NMQR 880) (MDE, 24 March 2014).';

TEXT TAXON = 74 CHARACTER = 63 TEXT = 'Based on SAM-PK-6047A and the left side of SAM-PK-5867 (MDE, 18 September 2014).';

TEXT TAXON = 99 CHARACTER = 65 TEXT = 'The dorsal process is present in UFRGS-PV-0152-T and the dorsal prong of the anterior tip of the jugal figured by Parrish 1993 for UFRGS-PV-0156-T might be a dorsal proess extending from the maxilla (MDE, 4 September 2014).';

TEXT TAXON = 13 CHARACTER = 66 TEXT = 'Based on Wild (1973: fig. 26) (MDE, 7 March 2014).';

TEXT TAXON = 25 CHARACTER = 66 TEXT = 'Based on Dilkes (1998: 505) and SAM-PK-6536 (MDE, 3 March 2014).';

TEXT TAXON = 38 CHARACTER = 66 TEXT = 'Based on CT data of BSPG 1934 VIII 514 (MDE, 5 September 2014).';

TEXT TAXON = 74 CHARACTER = 66 TEXT = 'Based on UMZC T692 and Gow (1970) (MDE, 28 March 2014).';

TEXT TAXON = 92 CHARACTER = 66 TEXT = 'Based on Walker (1964: 72) (MDE, 20 August 2014).';

TEXT TAXON = 96 CHARACTER = 66 TEXT = 'Based on Lecuona (2013) and Butler et al. (2014) (MDE, 22 August 2014).';

TEXT TAXON = 74 CHARACTER = 67 TEXT = 'Based on SAM-PK-6050 (MDE, 21 October 2014).';

TEXT TAXON = 26 CHARACTER = 68 TEXT = 'Based on Dilkes (1998: character 16) (MDE, 10 September 2012).';

TEXT TAXON = 74 CHARACTER = 68 TEXT = 'The ventral margin of the maxilla is slightly convex in SAM-PK-5867 (MDE, 11 September 2012).';

TEXT TAXON = 6 CHARACTER = 75 TEXT = 'Based on Evans (1980: 225) (MDE, 20 September 2012).';

TEXT TAXON = 10 CHARACTER = 75 TEXT = 'Gottman-Quesada and Sander (2009: 141) described 28 (+-1) tooth positions in the maxilla of NMK S 180 (MDE, 3 September 2012).';

TEXT TAXON = 26 CHARACTER = 75 TEXT = 'I think that this character is not applicable for Howesia and more derived rhynchosaurs (MDE, 3 September 2012).';

TEXT TAXON = 3 CHARACTER = 76 TEXT = 'Based on GHG K106 (MDE, 20 November 2012).';

TEXT TAXON = 6 CHARACTER = 76 TEXT = 'Based on Evans (1980: fig. 1c) (MDE, 20 September 2012).';

TEXT TAXON = 13 CHARACTER = 76 TEXT = 'Based on Wild (1973) (MDE, 2 September 2012).';

TEXT TAXON = 19 CHARACTER = 76 TEXT = 'No skull of Trilophosaurus buettneri preserves both antorbital and postorbital regions (MDE, 4 September 2012).';

TEXT TAXON = 78 CHARACTER = 76 TEXT = 'The suture between the frontals and parietals cannot be confidently traced in available specimens (MDE, 8 July 2014).';

TEXT TAXON = 4 CHARACTER = 77 TEXT = 'The snout of AM 3585 is broad and the natural moulds of the nasals indicate that these bones were dorsal elements (MDE, 3 September 2012).';

TEXT TAXON = 19 CHARACTER = 77 TEXT = 'The snout of Trilophosaurus buettneri is narrow and tall, and the nasal has a strong vertical contribution to the snout (Spielmann et al. 2008: figs. 20-22) (MDE, 4 September 2012).';

TEXT TAXON = 13 CHARACTER = 78 TEXT = 'Based on PIMUZ T2484 (MDE, 11 September 2012).';

TEXT TAXON = 64 CHARACTER = 83 TEXT = 'Based on the left side of BP/1/5207 (MDE, 21 March 2014).';

TEXT CHARACTER = 85 TEXT = 'Made_reproducible_after_deBraga_&_Reisz_1995._(DM)';

TEXT TAXON = 13 CHARACTER = 85 TEXT = 'Based on Wild (1973: fig. 1: f.n.) (MDE, 20 June 2012). ';

TEXT TAXON = 78 CHARACTER = 87 TEXT = 'Based on PVL 4606 (MDE, 8 July 2014).';

TEXT TAXON = 92 CHARACTER = 87 TEXT = 'Based on Walker (1964: fig. 3a) (MDE, 20 August 2014).';

TEXT TAXON = 3 CHARACTER = 89 TEXT = 'The foramen for the opening of the nasolacrimal duct could not be located in any of the studies specimens (e.g. BP/1/2459, 3859, GHG K 106, GHG RS 160, TM 1490, 4095) (MDE, 11 August 2013).';

TEXT TAXON = 16 CHARACTER = 89 TEXT = 'No foramen for the lacrimal duct is present (Flynn et al., 2010: 677) (MDE, 18 February 2014).';

TEXT TAXON = 19 CHARACTER = 89 TEXT = 'The new interpretations of the skull of Trilophosaurus buettneri strongly differs from those of Gregory (1945) regarding the shape and position of the lacrimal and the foramen showed by this author should not be positioned within the lacrimal based on Parks (1969) and Spielmann et al. (2008). However, the latter author did not give information about the passage of the lacrimal duct in Trilophosaurus buettneri (MDE, 14 September 2012).';

TEXT TAXON = 38 CHARACTER = 89 TEXT = 'Based on RC 846 (MDE, 5 September 2014).';

TEXT TAXON = 74 CHARACTER = 89 TEXT = 'Based on Senter (2003) (MDE, 28 March 2014).';

TEXT TAXON = 98 CHARACTER = 89 TEXT = 'Based on Gower (1999: 39) (MDE, 22 April 2014).';

TEXT TAXON = 2 CHARACTER = 90 TEXT = 'The lacrial duct opens laterally on the lacrimal, close to the posterior margin of the bone (MNHN 1908-32-57) (MDE, 10 August 2013).';

TEXT TAXON = 3 CHARACTER = 90 TEXT = 'The foramen for the opening of the nasolacrimal duct could not be located in any of the studies specimens (e.g. BP/1/2459, 3859, GHG K 106, GHG RS 160, TM 1490, 4095) (MDE, 11 August 2013).';

TEXT TAXON = 6 CHARACTER = 90 TEXT = 'Based on Evans (1980: 212) (MDE, 20 September 2012).';

TEXT TAXON = 13 CHARACTER = 90 TEXT = 'Based on Wild (1973: fig. 1: f.n.) (MDE, 20 June 2012). ';

TEXT TAXON = 16 CHARACTER = 90 TEXT = 'No foramen for the lacrimal duct is present (Flynn et al., 2010: 677) (MDE, 18 February 2014).';

TEXT TAXON = 19 CHARACTER = 90 TEXT = 'The new interpretations of the skull of Trilophosaurus buettneri strongly differs from those of Gregory (1945) regarding the shape and position of the lacrimal and the foramen showed by this author should not be positioned within the lacrimal based on Parks (1969) and Spielmann et al. (2008). However, the latter author did not give information about the passage of the lacrimal duct in Trilophosaurus buettneri (MDE, 14 September 2012).';

TEXT TAXON = 25 CHARACTER = 90 TEXT = 'Based on Dilkes (1998: 505) (MDE, 13 September 2012).';

TEXT TAXON = 27 CHARACTER = 90 TEXT = 'Based on the natural mould of the passage of the nasolacrimal duct (MDE, 8 September 2014).';

TEXT TAXON = 38 CHARACTER = 90 TEXT = 'Based on RC 846 (MDE, 5 September 2014).';

TEXT TAXON = 3 CHARACTER = 91 TEXT = 'The ventral margin of the ventral temporal bar (i.e. formed by the posterior process of the jugal and the anterior process of the quadratojugal) is concave in BP/1/3859 (MDE, 11 August 2013).';

TEXT TAXON = 19 CHARACTER = 91 TEXT = 'The ventral margin of the postorbital region of the skull is severely damaged in TMM 31025-140 and the condition of the character cannot be confidently assessed (Spielmann et al. 2008: fig. 19) (MDE, 4 September 2012).';

TEXT TAXON = 14 CHARACTER = 92 TEXT = 'Based on ZAR 08 (MDE, 1 May 2014).';

TEXT TAXON = 3 CHARACTER = 95 TEXT = 'Based on BP/1/3859 (MDE, 7 February 2014).';

TEXT TAXON = 12 CHARACTER = 95 TEXT = 'Based on PIMUZ T4822 (MDE, 10 October 2012).';

TEXT TAXON = 14 CHARACTER = 95 TEXT = 'Based on ZAR 08 (MDE, 1 May 2014).';

TEXT TAXON = 78 CHARACTER = 96 TEXT = 'Based on PVL 4606 (MDE, 8 July 2014).';

TEXT TAXON = 91 CHARACTER = 96 TEXT = 'Based on UCMP 27200, MCZ 1029 and Camp (1930: fig. 11) (MDE, 6 May 2014).';

TEXT TAXON = 16 CHARACTER = 100 TEXT = 'The height of the base of the posterior process is measured orthogonally to the main axis of the process (MDE, 18 February 2014).';

TEXT TAXON = 32 CHARACTER = 103 TEXT = 'State 0 in SAM-PK-K10797 and state 1 in BP/1/3575 and BP/1/5375 (MDE, 5 March 2014).';

TEXT TAXON = 64 CHARACTER = 104 TEXT = 'In BP/1/5207 the base of the posterior process of the jugal seems to be semi-elliptical, but it is not the case in BP/1/3893 (MDE, 10 April 2014).';

TEXT TAXON = 2 CHARACTER = 108 TEXT = 'The morphology of the suture between the prefrontal and nasal cannot be confidently determined and, particularly, because the skull is exposed in medial view (MNHN 1908-32-57) (MDE, 11 August 2013).';

TEXT TAXON = 3 CHARACTER = 108 TEXT = 'The suture between the prefrontal and nasal is anterolaterally oriented along its entire extension (BP/1/3859, GHG K 106) (MDE, 11 August 2013).';

TEXT TAXON = 13 CHARACTER = 108 TEXT = 'Based on Nosotti (2007: fig. 44) (MDE, 2 Septermber 2012).';

TEXT TAXON = 12 CHARACTER = 113 TEXT = 'Based on PIMUZ T4822 (MDE, 10 October 2012).';

TEXT TAXON = 19 CHARACTER = 114 TEXT = 'The orbital border of the frontal is broad in TMM 31025-207 (Spielmann et al. 2008: fig. 20e, f). Accordingly, the scoring of this character was changed from (0) to (1) (MDE, 4 September 2012).';

TEXT TAXON = 64 CHARACTER = 114 TEXT = 'The orbital margin of the frontal is narrow (MDE, 22 June 2012).';

TEXT TAXON = 13 CHARACTER = 115 TEXT = 'Based on PIMUZ T2189 (MDE, 7 March 2014).';

TEXT TAXON = 5 CHARACTER = 116 TEXT = 'The posterolateral process of the frontal is long and the suture with the parietal forms an acute angle with the parasagittal plane (Fraser, 1982: fig. 1c). Accordingly, the scoring of this character was changed from (1) to (2) (MDE, 11 August 2013).';

TEXT TAXON = 13 CHARACTER = 116 TEXT = 'Based on PIMUZ T2189 (MDE, 7 March 2014).';

TEXT TAXON = 35 CHARACTER = 116 TEXT = 'Based on Tatarinov (1978: 508) (MDE, 2 April 2015).';

TEXT TAXON = 38 CHARACTER = 116 TEXT = 'In large specimens of Proterosuchus fergusi (e.g. SAM-PK-K10603) the fr-p suture forms a right to obtuse angle to parasagittal plane, but in smaller specimens (e.g. RC 59) the fr-p suture forms an acute angle, similar to Prolacerta (Modesto & Sues, 2004: fig. 4). Thus, this feature seems to be related to intraspecific variation (probably ontogenetic) in Proterosuchus fergusi (MDE, 19 June 2012).';

TEXT TAXON = 35 CHARACTER = 120 TEXT = 'Based on Tatarinov (1978: 508) (MDE, 25 March 2014).';

TEXT TAXON = 12 CHARACTER = 121 TEXT = 'Based on PIMUZ T2472 (MDE, 17 February 2014).';

TEXT TAXON = 26 CHARACTER = 121 TEXT = 'The maximum constriction of the olfactory tract mould is situated well posteriorly to the broken anterior margin of the frontals (SAM-PK-5885) (MDE, 7 March 2014).';

TEXT CHARACTER = 123 TEXT = 'Merger_of_48_and_101_(identical!_--_A1)_and_54_(B3)._(DM)';

TEXT TAXON = 2 CHARACTER = 123 TEXT = 'The anterior end of the anterior process of the preserved postorbital and the parietals are missing. As a result, it is not possible to determine if the postfrontal participated in the supratermporal fenestra or not (i.e. if it was a contact between the postorbital and parietal) (MNHN 1908-32-57) (MDE, 10 August 2013).';

TEXT TAXON = 13 CHARACTER = 123 TEXT = 'Based on Nosotti (2007: 81) (MDE, 11 August 2013).';

TEXT TAXON = 16 CHARACTER = 123 TEXT = 'Based on Flynn et al. (2010: 679) (MDE, 14 February 2014).';

TEXT TAXON = 35 CHARACTER = 125 TEXT = 'The condition is not consistent between Tatarinov (1978) and Benton & Allen (1997) (MDE, 25 March 2014).';

TEXT TAXON = 2 CHARACTER = 128 TEXT = 'Only the mould of the medial surface of the postorbital is preserved and it cannot be determined the morphology of the lateral surface of the bone (MNHN 1908-32-57). Accordingly, the state of this character was changed from (0) to (?) (MDE, 10 August 2013).';

TEXT CHARACTER = 130 TEXT = 'Definition_of_state_1:_if_temporal_fenestrae_absent:_reaches_supratemporal,_if_at_least_one_fenestra_present:_extends_beyond_caudal_margin_of_fenestrae._This_avoids_inapplicability_and_the_need_for_quantification._(DM)';

TEXT TAXON = 12 CHARACTER = 130 TEXT = 'The posterior process of the quadratojugal does not reach the posterior border of the supratemporal fenesrtra defined by the squamosal (PIMUZ T4822) (MDE, 30 August 2012).';

TEXT TAXON = 38 CHARACTER = 130 TEXT = 'The posterior process of the postorbital extends beyond the posterior margin of the supratemporal fenestra in Proterosuchus fergusi (e.g. BP/1/4016 and SAM-PK-K10603) (MDE, 19 June 2012).';

TEXT TAXON = 82 CHARACTER = 131 TEXT = 'The skull is strongly dorsoventrally compressed beause of taphonomic processes and the character cannot be confidently scored (MDE, 18 August 2014). ';

TEXT TAXON = 16 CHARACTER = 137 TEXT = 'Based on Flynn et al. (2010: fig. 1A) (MDE, 18 February 2014).';

TEXT TAXON = 32 CHARACTER = 137 TEXT = 'Based on UMZC 2003.41R (MDE, 5 April 2014).';

TEXT TAXON = 35 CHARACTER = 137 TEXT = 'The condition is not consistent between Tatarinov (1978) and Benton & Allen (1997) (MDE, 25 March 2014).';

TEXT CHARACTER = 138 TEXT = 'Coded_as_inapplicable_in_the_absence_of_an_infratemporal_fenestra._(DM)';

TEXT TAXON = 6 CHARACTER = 138 TEXT = 'The condition is almost identical to that of Planocephalosaurus (compare Evans 1980: fig. 1 and Fraser 1982: fig. 1) (MDE, 20 September 2012).';

TEXT TAXON = 35 CHARACTER = 139 TEXT = 'The condition is not consistent between Tatarinov (1978) and Benton & Allen (1997) (MDE, 25 March 2014).';

TEXT TAXON = 74 CHARACTER = 141 TEXT = 'Based on the left squamosal of SAM-PK-5867 (MDE, 28 March 2014).';

TEXT TAXON = 35 CHARACTER = 145 TEXT = 'The condition is not consistent between Tatarinov (1978) and Benton & Allen (1997) (MDE, 25 March 2014).';

TEXT TAXON = 35 CHARACTER = 146 TEXT = 'The condition is not consistent between Tatarinov (1978) and Benton & Allen (1997) (MDE, 25 March 2014).';

TEXT TAXON = 35 CHARACTER = 148 TEXT = 'The condition is not consistent between Tatarinov (1978) and Benton & Allen (1997) (MDE, 25 March 2014).';

TEXT CHARACTER = 150 TEXT = 'States_0_and_1_exchanged_so_the_character_can_be_ordered._(DM)';

TEXT TAXON = 2 CHARACTER = 150 TEXT = 'It is not clear if the quadratojugal was absent or not (Bicklemann et al. 2009) (MDE, 10 August 2013).';

TEXT TAXON = 6 CHARACTER = 150 TEXT = 'Based on Evans (1980: 216) (MDE, 20 September 2012).';

TEXT TAXON = 10 CHARACTER = 150 TEXT = 'The presence of a quadratojugal is doubtful in Protorosaurus (MDE, 3 September 2012).';

TEXT TAXON = 12 CHARACTER = 150 TEXT = 'The quadratojugal is probably absent in Macrocnemus (PIMUZ T4822), but I cannot assess the condition confidently (MDE, 30 August 2012).';

TEXT TAXON = 13 CHARACTER = 150 TEXT = 'The quadratojugal is absent (Wild, 1973, Nosotti, 2007) (MDE, 22 June 2012).';

TEXT TAXON = 19 CHARACTER = 150 TEXT = 'The quadratojugal of TMM 31025-140 is broken and the anterior extent of this bone cannot be assessed with the currently available specimnes (Spielman et al. 2008: fig. 19). As a result, the scoring of this character has been changed from (2) to (?) (MDE, 4 September 2012). ';

TEXT TAXON = 39 CHARACTER = 150 TEXT = 'Based on the facet on the quadrate (NMQR 1484) (MDE, 24 March 2014).';

TEXT TAXON = 10 CHARACTER = 153 TEXT = 'The presence of a quadratojugal is doubtful in Protorosaurus (MDE, 3 September 2012).';

TEXT TAXON = 12 CHARACTER = 153 TEXT = 'I couldn''t recognize a quadratojugal in Macrocnemus specimens (MDE, 30 August 2012).';

TEXT TAXON = 13 CHARACTER = 153 TEXT = 'Tanystropheus lacks of a quadratojugal (Wild, 1973: figs. 8?10) and Nosotti (2007) (MDE, 20 June 2012).';

TEXT TAXON = 92 CHARACTER = 153 TEXT = 'Based on the facet along the entire ventral margin of the posterior process of the jugal present in NHMUK R3142 (MDE, 20 August 2014).';

TEXT TAXON = 6 CHARACTER = 157 TEXT = 'Based on Evans (1980: 212) (MDE, 20 September 2012).';

TEXT TAXON = 13 CHARACTER = 157 TEXT = 'This character is scored as unkown based on Nosotti (2007) (MDE, 2 September 2012).';

TEXT TAXON = 25 CHARACTER = 157 TEXT = 'The supratemporal of SAM-PK-6536 is considerably broader than the slit-like bone of Proterosuchus fergusi. Accordingly, it is scores as condition (0) (MDE, 27 August 2012).';

TEXT TAXON = 32 CHARACTER = 157 TEXT = 'However, the specimen that I identified as Prolacerta broomi in the GHG collection (431) seems to lack supratemporals and a supratemporal facet in both squamosals (MDE, 19 June 2012). Is this specimen actually Prolacerta broomi?';

TEXT TAXON = 38 CHARACTER = 157 TEXT = 'Both supratemporals are clearly present in SAM-PK-K10603 and SAM-PK-K140 (MDE, 19 June 2012).';

TEXT TAXON = 10 CHARACTER = 159 TEXT = 'Gottman-Quesada and Sander (2009: 142) (MDE, 10 September 2012).';

TEXT TAXON = 13 CHARACTER = 159 TEXT = 'In small individuals the parietals are not fused to each other, whereas the opposite occurs in large individuals (Nosotti 2007: 51). We have considered here only the adult condition (MDE, 11 September 2012).';

TEXT TAXON = 19 CHARACTER = 160 TEXT = 'The parietals do not extend over the interorbital region in TMM 31025-140 (Spielmann et al. 2008: fig. 19) (MDE, 4 September 2012).';

TEXT TAXON = 38 CHARACTER = 160 TEXT = 'Proterosuchus fergusi possesses intraspecific variation for this character, in large specimens (e.g. SAM-PK-K10603) the parietal does not extend over the interorbital region, but in smaller specimens (e.g. RC 59) the parietal considerably extends over the interorbital region. Probably it is related to an ontogenetic variation (MDE, 19 June 2012).';

TEXT CHARACTER = 164 TEXT = 'Why_did_you_replace_the_position_character_with_this_size_character?_These_are_two_independent_characters._I_have_added_the_position_character_back._(DM)';

TEXT TAXON = 4 CHARACTER = 164 TEXT = 'The dorsal surface of the parietals are too damaged to determine confidently the size and shape of the pineal foramen (MDE, 14 February 2014).';

TEXT TAXON = 5 CHARACTER = 164 TEXT = '25.5% based on Fraser (1982: fig. 1c). Accordingly, the scoring of this character was changed from (1) to (0) (MDE, 11 August 2013).';

TEXT TAXON = 6 CHARACTER = 164 TEXT = 'Ratio=21.67% based on Evans (1980: fig. 9a) (MDE, 20 September 2012).';

TEXT TAXON = 10 CHARACTER = 164 TEXT = 'Gottman-Quesada and Sander (2009: 142) described that the presence of a pineal foramen seems to be a variable condition in Protorosaurus (MDE, 3 September 2012). ';

TEXT TAXON = 13 CHARACTER = 164 TEXT = 'The size of the pineal foramen seems to range between 26.90% to 18.09% (Wild, 1973: fig. 1) (MDE, 20 June 2012).';

TEXT TAXON = 25 CHARACTER = 164 TEXT = 'Ratio=18.89% based on SAM-PK-6536 (Dilkes, 1998: fig. 5) (MDE, 27 August 2012).';

TEXT TAXON = 26 CHARACTER = 164 TEXT = 'Based on Dilkes (1995: 670) (MDE, 7 March 2014).';

TEXT TAXON = 35 CHARACTER = 164 TEXT = 'Based on Tatarinov (1978: 508) (MDE, 2 April 2015).';

TEXT TAXON = 38 CHARACTER = 164 TEXT = 'In only one specimen of Proterosuchus fergusi I have seen a very small pineal foramen (BP/1/3993). In other well-preserved specimens the pineal foramen is definitely absent (e.g. RC 59, SAM-PK-K10603). So, this character seems to be polymorphic, as in Prolacerta broomi (MDE, 19 June 2012).';

TEXT TAXON = 64 CHARACTER = 164 TEXT = 'In well-preserved specimens of Erythrosuchus africanus (e.g. NM QR 1473) the pineal foramen is absent (MDE, 22 June 2012).';

TEXT CHARACTER = 165 TEXT = 'Inapplicable_in_the_absence_of_a_pineal_foramen._--_The_states_should_probably_be_defined_differently._(DM)';

TEXT TAXON = 1 CHARACTER = 165 TEXT = 'Borderline_--_depends_on_left_vs_right_parietal..._(DM)';

TEXT TAXON = 3 CHARACTER = 165 TEXT = 'Ratio_suture_anterior/posterior_to_foramen_in_Carroll_1981:_fig._9:_7.15/5.58_(ML)';

TEXT TAXON = 13 CHARACTER = 165 TEXT = 'In different specimens the pineal foramen is positioned at the frontal-parietal suture or it is completely enclosed by the parietals (Nosotti 2007: 51). As a result, this character was scored as polymorphic for Tanystropheus longobardicus (MDE, 2 September 2012).';

TEXT TAXON = 38 CHARACTER = 165 TEXT = 'Scoring based on BP/1/3993 (MDE, 19 June 2012).';

TEXT TAXON = 73 CHARACTER = 170 TEXT = 'Nesbitt et al. (2013: 11) (MDE, 25 September 2014).';

TEXT TAXON = 1 CHARACTER = 171 TEXT = 'Borderline._(DM)';

TEXT TAXON = 19 CHARACTER = 171 TEXT = 'The postparietal cannot be discerned in Trilophosaurus buettneri (Gregory 1945: 278) (MDE, 4 September 2012).';

TEXT TAXON = 25 CHARACTER = 171 TEXT = 'Dilkes (1998: character 29) scores Mesosuchus as lacking of a postparietal (MDE, 27 August 2012).';

TEXT TAXON = 32 CHARACTER = 171 TEXT = 'The postparietals are absent in Prolacerta broomi (Modesto and Sues, 2004). Accordingly, the scording of this character was changed from (1/2) to (2) (MDE, 11 August 2013).';

TEXT TAXON = 26 CHARACTER = 176 TEXT = 'Based on SAM-PK-5885 (MDE, 3 September 2012).';

TEXT TAXON = 35 CHARACTER = 183 TEXT = 'Based on Tatarinov (1978: 510) (MDE, 2 April 2014).';

TEXT TAXON = 12 CHARACTER = 185 TEXT = 'Based on Peyer (1937: 55) (MDE, 17 February 2014).';

TEXT TAXON = 35 CHARACTER = 185 TEXT = 'Based on Tatarinov (1978: 509) (MDE, 2 April 2015).';

TEXT TAXON = 13 CHARACTER = 186 TEXT = 'Based on Wild (1973).';

TEXT TAXON = 25 CHARACTER = 186 TEXT = 'Based on Dilkes (1998: character 38) (MDE, 11 September 2012).';

TEXT TAXON = 32 CHARACTER = 186 TEXT = 'Based on the desarticulated vomer of BPI/1/2675 (MDE, 13 September 2012).';

TEXT TAXON = 10 CHARACTER = 187 TEXT = 'Based on Gottman-Quesada and Sander (2009: 146) (MDE, 13 February 2014).';

TEXT TAXON = 74 CHARACTER = 187 TEXT = 'Based on Gow (1970) (MDE, 14 February 2014).';

TEXT TAXON = 13 CHARACTER = 188 TEXT = 'The condition of this character seems to be variable within Tanytropheus longobardicus (Wild 1973, Nosotti 2007: 80) (MDE, 11 September 2012). Based on Wild (1973) (MDE, 10 March 2014).';

TEXT TAXON = 12 CHARACTER = 189 TEXT = 'Based on Besano II (BSPG 1973 I 86)';

TEXT TAXON = 3 CHARACTER = 192 TEXT = 'Based on UC 1528 (MDE, 6 September 2014).';

TEXT TAXON = 4 CHARACTER = 192 TEXT = 'Based on AM 3585 (MDE, 16 September 2012).';

TEXT TAXON = 5 CHARACTER = 192 TEXT = 'Based on Fraser (1982: 718) (MDE, 16 September 2012).';

TEXT TAXON = 26 CHARACTER = 192 TEXT = 'Based on Dilkes (1995: 670) (MDE, 7 March 2014).';

TEXT TAXON = 28 CHARACTER = 192 TEXT = 'Based on Benton (1990: 231) (MDE, 3 April 2014).';

TEXT TAXON = 35 CHARACTER = 192 TEXT = 'Based on Benton and Allen (1997: 936) (MDE, 2 April 2014).';

TEXT TAXON = 64 CHARACTER = 192 TEXT = 'Based on Gower (2003: 31) (MDE, 16 September 2012).';

TEXT TAXON = 76 CHARACTER = 192 TEXT = 'Based on Dilkes and Arcucci (2012: 16) (MDE, 14 April 2014).';

TEXT TAXON = 3 CHARACTER = 193 TEXT = 'Based on UC 1528 (MDE, 6 September 2014).';

TEXT TAXON = 10 CHARACTER = 195 TEXT = 'Gottman-Quesada and Sander (2009:146) (MDE, 10 September 2012).';

TEXT TAXON = 12 CHARACTER = 195 TEXT = 'Based on Peyer (1937: 60) and Besano II (MDE, 6 September 2012).';

TEXT TAXON = 32 CHARACTER = 196 TEXT = 'Based on BP/1/5066 (MDE, 5 March 2014).';

TEXT TAXON = 3 CHARACTER = 197 TEXT = 'Based on Gow (1975: 94) (MDE, 10 February 2014).';

TEXT TAXON = 32 CHARACTER = 197 TEXT = 'Based on BP/1/5066 (MDE, 5 March 2014).';

TEXT TAXON = 35 CHARACTER = 199 TEXT = 'Based on Tatarinov (1978: 510) (MDE, 2 April 2014).';

TEXT TAXON = 74 CHARACTER = 202 TEXT = 'Based on SAM-PK-13664 (MDE, 27 August 2012).';

TEXT TAXON = 25 CHARACTER = 204 TEXT = 'Based on Dilkes (1998: 508) (MDE, 3 March 2014).';

TEXT TAXON = 26 CHARACTER = 204 TEXT = 'Based on Dilkes (1995: 670) (MDE, 10 September 2012).';

TEXT TAXON = 32 CHARACTER = 205 TEXT = 'Based on BP/1/5066 (MDE, 5 March 2014).';

TEXT TAXON = 64 CHARACTER = 205 TEXT = 'Based on Gower (2003) (MDE, 21 March 2014).';

TEXT TAXON = 19 CHARACTER = 206 TEXT = 'Based on Spielmann et al. (2008: 27) (MDE, 13 November 2012).';

TEXT TAXON = 26 CHARACTER = 206 TEXT = 'Based on Dilkes (1995: 671) (MDE, 7 March 2014).';

TEXT TAXON = 28 CHARACTER = 206 TEXT = 'Based on NHMUK R1236 (MDE, 11 September 2014).';

TEXT TAXON = 26 CHARACTER = 207 TEXT = 'Based on DIlkes (1998: character 39) (MDE, 10 September 2012).';

TEXT TAXON = 26 CHARACTER = 209 TEXT = 'Based on Dilkes (1995: 671) (MDE, 7 March 2014).';

TEXT TAXON = 29 CHARACTER = 209 TEXT = 'The occipital surface of hte braincase is very damaged (MDE, 9 September 2014).';

TEXT TAXON = 32 CHARACTER = 209 TEXT = 'Based on SAM-PK-K10018 and Gow (1975: 105) (MDE, 5 September 2014).';

TEXT TAXON = 38 CHARACTER = 209 TEXT = 'Based on BSPG 1934 VIII 514 and SAM-PK-K1603 (MDE, 5 September 2014).';

TEXT TAXON = 64 CHARACTER = 209 TEXT = 'Based on Gower (1997: 560) (MDE, 21 March 2014).';

TEXT TAXON = 72 CHARACTER = 209 TEXT = 'Based on Nesbitt et al. (2009: 826) (MDE, 25 April 2014).';

TEXT TAXON = 99 CHARACTER = 209 TEXT = 'Based on Mastrantonio et al. (2013) (MDE, 4 September 2014).';

TEXT TAXON = 13 CHARACTER = 211 TEXT = 'Based on Nosotti (2007: 12) (MDE, 10 March 2014).';

TEXT TAXON = 72 CHARACTER = 211 TEXT = 'Based on Nesbitt et al. (2009: 826) (MDE, 25 April 2014).';

TEXT TAXON = 74 CHARACTER = 211 TEXT = 'Based on SAM-PK-7696 (MDE, 28 March 2014).';

TEXT TAXON = 74 CHARACTER = 213 TEXT = 'Based on SAM-PK-7696 (MDE, 28 March 2014).';

TEXT TAXON = 10 CHARACTER = 214 TEXT = 'Based on Gottman-Quesada and Sander (2009:146) (MDE, 3 September 2012).';

TEXT TAXON = 19 CHARACTER = 214 TEXT = 'The paroccipital processes of Trilophosaurus buettneri have a weak contact with the squamosal (Spiealmann et al. 2008: fig. 18) (MDE, 14 September 2012).';

TEXT TAXON = 32 CHARACTER = 214 TEXT = 'In Prolacerta broomi the paroccipital processes possess a weak contact with the posterior temporal region of the skull (BPI/1/471) (MDE, 14 September 2012). ';

TEXT TAXON = 74 CHARACTER = 214 TEXT = 'Based on SAM-PK-5867 (MDE, 14 September 2012).';

TEXT TAXON = 4 CHARACTER = 217 TEXT = 'Based on AM 3585 (MDE, 16 September 2012).';

TEXT TAXON = 10 CHARACTER = 217 TEXT = 'Based on BSPG 1995 I 5 (MDE, 16 September 2012).';

TEXT TAXON = 19 CHARACTER = 217 TEXT = 'Based on Spielmann et al. (2008: figs. 25, 26) (MDE, 16 September 2012).';

TEXT TAXON = 32 CHARACTER = 217 TEXT = 'Based on BP/1/2675 (MDE, 5 March 2014).';

TEXT TAXON = 65 CHARACTER = 218 TEXT = 'Based on Gower and Sennikov (1996) (MDE, 26 March 2015).';

TEXT TAXON = 32 CHARACTER = 220 TEXT = 'Based on BP/1/5066 (MDE, 5 March 2014).';

TEXT TAXON = 98 CHARACTER = 220 TEXT = 'Based on Gower (2002) (MDE, 19 September 2014).';

TEXT TAXON = 99 CHARACTER = 220 TEXT = 'Based on Mastrantonio et al. (2013) (MDE, 19 September 2014).';

TEXT TAXON = 25 CHARACTER = 221 TEXT = 'Cannot be determined bcause of the fusion between exoccipitals and basioccipital (MDE, 3 March 2014).';

TEXT TAXON = 32 CHARACTER = 221 TEXT = 'Based on BP/1/5066 (MDE, 5 March 2014).';

TEXT TAXON = 74 CHARACTER = 221 TEXT = 'Based on Gower and Weber (1998: 374) (MDE, 18 August 2014).';

TEXT TAXON = 76 CHARACTER = 221 TEXT = 'The contact between exoccipitals on the floor of the endocranial cavity is uncertain (Trotteyn and Haro 2010, Dilkes and Arcucci 2012) (MDE, 14 April 2014).';

TEXT TAXON = 103 CHARACTER = 221 TEXT = 'Based on Bittencourt et al. (2014: 8) (MDE, 21 August 2014).';

TEXT TAXON = 3 CHARACTER = 222 TEXT = 'Based on Evans (1987: 195) (MDE, 7 September 2014).';

TEXT TAXON = 13 CHARACTER = 222 TEXT = 'Based on PIMUZ T2189 (MDE, 10 March 2014).';

TEXT TAXON = 19 CHARACTER = 222 TEXT = 'Based on Spielmann et al. (2008: 32) (MDE, 25 February 2014).';

TEXT TAXON = 32 CHARACTER = 222 TEXT = 'Based on BP/1/5066 (MDE, 5 March 2014).';

TEXT TAXON = 57 CHARACTER = 222 TEXT = 'Although scored in the data matrix of Gower and Sennikov (1997: character 34), they say in the text that no trace of the hypoglosal foramina can be detected (2 April 2014).';

TEXT TAXON = 64 CHARACTER = 222 TEXT = 'Based on Gower (1996: 560) (MDE, 21 March 2014).';

TEXT TAXON = 72 CHARACTER = 222 TEXT = 'Based on Nesbitt et al. (2009: 826) (MDE, 25 April 2014).';

TEXT TAXON = 3 CHARACTER = 225 TEXT = 'Based on Gardener et al. (2010: fig. 3) (MDE, 15 October 2012).';

TEXT TAXON = 6 CHARACTER = 225 TEXT = 'Based on Evans (1980: 227) (MDE, 14 February 2014).';

TEXT TAXON = 26 CHARACTER = 225 TEXT = 'SAM-PK-5885 seems to not be a fully mature individual (MDE, 7 March 2014).';

TEXT TAXON = 76 CHARACTER = 225 TEXT = 'Based on PVL 2063 (MDE, 14 April 2014).';

TEXT TAXON = 63 CHARACTER = 227 TEXT = 'Based on BP/1/6232aa (MDE, 29 April 2014).';

TEXT TAXON = 6 CHARACTER = 228 TEXT = 'Based on Evans (1980: fig. 1) (MDE, 21 September 2012).';

TEXT TAXON = 26 CHARACTER = 229 TEXT = 'The posterior surface of the occipital condyle of SAM-PK-5885 is strongly damaged (MDE, 7 March 2014).';

TEXT TAXON = 32 CHARACTER = 231 TEXT = 'The occipital neck is extremely short in BP/1/2675 and anteroposteriorly long in BP/1/5066 (MDE, 5 March 2014).';

TEXT TAXON = 3 CHARACTER = 234 TEXT = 'Based on Evans (1987: fig. 2a) (MDE, 7 September 2014).';

TEXT TAXON = 74 CHARACTER = 237 TEXT = 'Based on SAM-PK-5867 and SAM-PK-7696 (cf. Gower and Weber, 1998, contra Nesbitt, 2011) (MDE, 19 April 2014).';

TEXT TAXON = 74 CHARACTER = 238 TEXT = 'Based on Gower and Weber (1998) (MDE, 22 August 2014).';

TEXT TAXON = 76 CHARACTER = 238 TEXT = 'Based on Dilkes and Arcucci (2012: 18) (MDE, 19 April 2014).';

TEXT TAXON = 81 CHARACTER = 238 TEXT = 'Based on Nesbitt (2011: 87) (MDE, 19 April 2014).';

TEXT TAXON = 1 CHARACTER = 240 TEXT = 'Based on Reisz (1981: 24) (MDE ,10 February 2014).';

TEXT TAXON = 3 CHARACTER = 240 TEXT = 'Based on Evans (1987: 196) (MDE, 7 September 2014).';

TEXT TAXON = 26 CHARACTER = 240 TEXT = 'Although Dilkes (1998: character 45) scored this character as (?) in Howesia the foramina for the exit of the carotids are clearly ventrally situated in the parashpenoid in SAM-PK-5885 (MDE, 10 September 2012).';

TEXT TAXON = 74 CHARACTER = 240 TEXT = 'The foramina for the entrance of the internal carotid are positioned ventrally in the basisphenoid in UMZC T692 (MDE, 11 September 2012).';

TEXT TAXON = 76 CHARACTER = 240 TEXT = 'Based on Dilkes and Arcucci (2012: 18) (MDE, 19 April 2014).';

TEXT TAXON = 84 CHARACTER = 240 TEXT = 'The entrance for the internal carotid seems to have been labelled as CN VI by Desojo et al. (2011) (MDE, 19 April 2014).';

TEXT TAXON = 38 CHARACTER = 246 TEXT = 'Based on BP/1/3993 (MDE, 23 September 2014).';

TEXT CHARACTER = 247 TEXT = 'Was_"basal_tubera",_but_the_basal_tubera_are_something_else_and_don''t_articulate_with_anything!_(DM)';

TEXT TAXON = 6 CHARACTER = 247 TEXT = 'Based on Evans (1980: 227) (MDE, 20 September 2012).';

TEXT TAXON = 32 CHARACTER = 247 TEXT = 'Not_absolutely_sure.__Some_specimens_might_be_closer_to_1,_and_apparently_variable_(Evans,_1986:_186-187_and_figures_4,_5).';

TEXT TAXON = 76 CHARACTER = 248 TEXT = 'Based on Dilkes and Arcucci (2012: 18) (MDE, 14 April 2014).';

TEXT TAXON = 74 CHARACTER = 249 TEXT = 'Based on Gower and Weber (1998: 379) (MDE, 31 March 2014).';

TEXT TAXON = 89 CHARACTER = 249 TEXT = 'The suture between supraoccipital and prootic cannot be discerned (MDE, 22 January 2015).';

TEXT TAXON = 3 CHARACTER = 250 TEXT = 'Based on Evans (1987: 201) (MDE, 31 March 2015).';

TEXT TAXON = 57 CHARACTER = 250 TEXT = 'Based on scoring of Gower and Sennikov (1997: character 21) (MDE, 31 March 2014).';

TEXT TAXON = 65 CHARACTER = 250 TEXT = 'Based on Gower and Sennikov (1996: 896) (MDE, 31 March 2014).';

TEXT TAXON = 74 CHARACTER = 250 TEXT = 'Based on Gower and Weber (1998: 377) (MDE, 31 March 2014).';

TEXT TAXON = 99 CHARACTER = 250 TEXT = 'Based on Mastrantonio et al. (2013) (MDE, April 12 2015).';

TEXT TAXON = 32 CHARACTER = 252 TEXT = 'Based on BPI/1/2675 and GHG 431 (MDE, 12 October 2012).';

TEXT TAXON = 64 CHARACTER = 252 TEXT = 'Based on Gower (1997: fig. 4a) (MDE, 23 March 2014).';

TEXT TAXON = 25 CHARACTER = 254 TEXT = 'Based on Dilkes (1998: 509) (MDE, 3 March 2014).';

TEXT TAXON = 25 CHARACTER = 255 TEXT = 'Based on Dilkes (1998: character 48) (MDE, 3 March 2014).';

TEXT TAXON = 25 CHARACTER = 256 TEXT = 'Following Nesbitt (2011: character 94) (MDE, 5 March 2014).';

TEXT TAXON = 74 CHARACTER = 256 TEXT = 'Based on SAM-PK-7696 (MDE, 16 March 2015).';

TEXT TAXON = 89 CHARACTER = 256 TEXT = 'Poorly preserved in the inferior anterior process (ISI, MDE 22 September 2015).';

TEXT TAXON = 3 CHARACTER = 257 TEXT = 'Based on Gardner et al. (2010: 8) (MDE, 9 February 2014).';

TEXT TAXON = 74 CHARACTER = 257 TEXT = 'Based on Gower and Weber (1998: 379) (MDE, 31 March 2014).';

TEXT TAXON = 35 CHARACTER = 258 TEXT = 'The sphenethmoid described by Tatarinov (1978: 510) seems to have been too anterior to be a laterophenoid (MDE, 2 May 2014).';

TEXT TAXON = 96 CHARACTER = 258 TEXT = 'Based on the right side of MCZ 4117 (MDE, 22 August 2014).';

TEXT TAXON = 105 CHARACTER = 258 TEXT = 'Inferred from the well-preserved and defined facet on the ventral surface of the skull roof of ZPAL Ab IIII 1223 (MDE, April 20 2015).';

TEXT TAXON = 12 CHARACTER = 261 TEXT = 'Based on PIMUZ T2472 (MDE, 17 February 2014).';

TEXT TAXON = 35 CHARACTER = 261 TEXT = 'Based on Tatarinov (1978: 510) (MDE, 2 April 2014).';

TEXT TAXON = 14 CHARACTER = 264 TEXT = 'Based on the right side of ZAR 08 (MDE, 2 May 2014).';

TEXT TAXON = 35 CHARACTER = 264 TEXT = 'Based on Tatarinov (1978: 510) (MDE, 2 April 2014).';

TEXT TAXON = 35 CHARACTER = 267 TEXT = 'Based on Benton and Allen (1997) (MDE, 19 September 2014).';

TEXT TAXON = 82 CHARACTER = 267 TEXT = 'The specimen is too artificially compressed to determine the character-state (MDE, 19 September 2014).';

TEXT TAXON = 1 CHARACTER = 268 TEXT = 'Based on Reisz (1981: fig. 11) (MDE, 10 February 2014).';

TEXT TAXON = 32 CHARACTER = 268 TEXT = 'Based on BP/1/2675 (MDE, 5 March 2014).';

TEXT TAXON = 107 CHARACTER = 270 TEXT = 'Based on MACN-Pv 18060 (MDE, 26 August 2014).';

TEXT TAXON = 3 CHARACTER = 271 TEXT = 'Based on GHG K 106 (dorsally expanded) and SAM-PK-K8565 (mostly horizontal) (MDE, 9 February 2014).';

TEXT TAXON = 10 CHARACTER = 272 TEXT = 'In at least two specimens (NMK S 180 and the Simon and Bartholomaeus specimen) seem to be two posterior processes in the dentary (MDE; 31 March 2015).';

TEXT TAXON = 74 CHARACTER = 272 TEXT = 'The base of a posterodorsal process seems to be present in SAM-PK-5867 (MDE, 31 March 2014).';

TEXT CHARACTER = 283 TEXT = 'Split,_see_65,_and_ordered._(DM)';

TEXT TAXON = 16 CHARACTER = 283 TEXT = 'Flynn et al. (2010: 683) (MDE, 18 February 2014).';

TEXT TAXON = 19 CHARACTER = 284 TEXT = 'Scoring changed from that of PhD from (0) to (1) (MDE, 30 January 2015).';

TEXT TAXON = 96 CHARACTER = 284 TEXT = 'Based on MCZ 4116 (MDE 5 October 2014).';

TEXT TAXON = 19 CHARACTER = 288 TEXT = 'Based on Spielmann et al. (2008: fig. 27) (MDE, 16 September 2012).';

TEXT TAXON = 106 CHARACTER = 288 TEXT = 'Based on Sereno (2012: 107) (MDE, 27 August 2014).';

TEXT TAXON = 19 CHARACTER = 289 TEXT = 'Based on Pritchard et al. 2015 (MDE, 28 March 2015).';

TEXT TAXON = 81 CHARACTER = 289 TEXT = 'The posterior surangular foramen is present in MCZ 4037 but it seems to be absent in PULR 07 and PVL 4586 (MDE, 22 April 2014).';

TEXT TAXON = 90 CHARACTER = 289 TEXT = 'Based on NHMUK R38036, 42744 (MDE, 16 March 2015).';

TEXT TAXON = 106 CHARACTER = 289 TEXT = 'Based on Sereno (2012: 107) (MDE, 27 August 2014).';

TEXT TAXON = 5 CHARACTER = 290 TEXT = 'The postdentary bones of Planocephalosaurus are fused with each other (Fraser, 1982) and, as a result, it is not possible to determine the degree of exposition of the angular in lateral view (MDE, 11 August 2013).';

TEXT TAXON = 19 CHARACTER = 290 TEXT = 'The exposition of the angular in lateral view is wide in TMM 31025-5 (Spielmann et al. 2008: fig. 28). Accordingly, the scoring was changed from (?) to (0) (MDE, 4 September 2012).';

TEXT TAXON = 26 CHARACTER = 290 TEXT = 'Dilkes (1995) said that the mould of the angular indicates that it had a limited lateral exposure (MDE, 3 September 2012).';

TEXT TAXON = 1 CHARACTER = 292 TEXT = 'Based on Reisz (1981: 26) (MDE, 10 February 2014).';

TEXT TAXON = 38 CHARACTER = 294 TEXT = 'Based on RC 846 (MDE, 5 September 2014).';

TEXT TAXON = 58 CHARACTER = 294 TEXT = 'There is an area filled with matrix that may represent a medial foramen on the articular, but it cannot be determined confidently (MDE, 12 April 2014).';

TEXT TAXON = 63 CHARACTER = 294 TEXT = 'Based on NMQR 3051 (MDE, 26 August 2014).';

TEXT TAXON = 64 CHARACTER = 294 TEXT = 'Based on Gower (2003: 35) (MDE, 26 August 2014).';

TEXT TAXON = 74 CHARACTER = 294 TEXT = 'Based on UMZC T6921 (MDE, 31 March 2014).';

TEXT TAXON = 96 CHARACTER = 294 TEXT = 'Based on MCZ 4116 (MDE 5 October 2014).';

TEXT TAXON = 32 CHARACTER = 295 TEXT = 'Based on BP/1/2675 (MDE, 23 September 2014).';

TEXT TAXON = 38 CHARACTER = 295 TEXT = 'e.g. BSPG 1934 VIII 514 (MDE, 23 September 2014).';

TEXT TAXON = 96 CHARACTER = 295 TEXT = 'Based on MCZ 4116 (MDE 5 October 2014).';

TEXT TAXON = 13 CHARACTER = 298 TEXT = 'Based on comparisons between the maxillae and dentaries several specimens (MDE, 10 March 2014).';

TEXT TAXON = 3 CHARACTER = 299 TEXT = 'Based on Gow (1975) and pers. obs. of BP/1/3859 and GHG K 106 (MDE, 11 August 2013).';

TEXT TAXON = 4 CHARACTER = 299 TEXT = 'In AM 3585 it couldn''t be discern the presence of alveoli in the tooth bearing bones and the tooth seem to be ankylosed to the bone, but the poor state of preservation of the specimen prevents assessing the condition confidently (MDE, 3 September 2012).';

TEXT TAXON = 6 CHARACTER = 299 TEXT = 'Based on Evans (1980: 236) (MDE, 20 September 2012).';

TEXT TAXON = 10 CHARACTER = 299 TEXT = 'The height of the maxillary tooth crowns in labial and linguals views is subequal in the Simon Bartholomaeus specimen (SMNS 55387). This suggests that the tooth implantation is subthecodont (MDE, 15 February 2015).';

TEXT TAXON = 12 CHARACTER = 299 TEXT = 'The teeth are implanted into deep alveoli and not ankylosed to the tooth-bearing bone. The lingual margen of the alveoli seems to be lower than the labial margen, thus, the character is scores as subthecodont based on PIMUZ T2472, T4355 and T4822 (MDE, 30 August 2012).';

TEXT TAXON = 13 CHARACTER = 299 TEXT = 'Wild (1973) says that the kind of tooth implantation of Tanystrophaeus is subthecodont (page 48) (MDE, 20 June 2012).';

TEXT TAXON = 32 CHARACTER = 299 TEXT = 'Modesto_&_Sues_2004._(DM)';

TEXT TAXON = 64 CHARACTER = 299 TEXT = 'It was added the fifth state. '' thecodont'' to that of the data matrix of Reisz et al. (2010) for Erythrosuchus africanus (MDE, 20 June 2012).';

TEXT CHARACTER = 303 TEXT = 'Removed_the_mention_of_compression_because_it''s_already_a_separate_character_(28,_now_4)._Also_note_that_I_exchanged_states_0_and_1_so_the_character_can_be_ordered._(DM)';

TEXT TAXON = 1 CHARACTER = 303 TEXT = 'Based on Reisz (1981: 27) (MDE, 10 February 2014).';

TEXT TAXON = 10 CHARACTER = 303 TEXT = 'Gottman-Quesada and Sander (2009: 148) described the crowns of Protorosaurus as straight. However, in BSPG 1995 I 5 (cast of WMsN P47361) a few maxillary teeth are slightly distally curved. As a result, I scored this character as polymorphic in Protorosaurus (MDE, 3 September 2012).';

TEXT TAXON = 13 CHARACTER = 303 TEXT = 'Tanystropheus is strongly heterodont and the crowns possess a strong to slight distal curvature or completely lack it (MDE, 20 June 2012).';

TEXT TAXON = 19 CHARACTER = 303 TEXT = 'This scoring was changed from (1) to (2) because Trilophosaurus buettneri lacks of a distal curvature in the margin teeth (Spielmann et al. 2008: figs. 18, 20, 21) (MDE, 4 September 2012).';

TEXT TAXON = 31 CHARACTER = 303 TEXT = 'Young (1973: fig. 1) illustrates a tooth crown with a convex mesial margin that it is not currently preserved in the specimen (MDE, April 7 2015).';

TEXT TAXON = 3 CHARACTER = 305 TEXT = 'Crowns are labiolingually compressed in GHG RS 160 and GHG K106, but not compressed in TM 1490 (MDE, 9 February 2014).';

TEXT TAXON = 5 CHARACTER = 305 TEXT = 'Based on Fraser (1982: 714, 719) (MDE, 3 March 2014).';

TEXT TAXON = 35 CHARACTER = 305 TEXT = 'Based on personal observation of the cast of the skull (MDE, April 7 2015).';

TEXT TAXON = 6 CHARACTER = 307 TEXT = 'Based on Evans (1980: 237) (MDE, 15 February 2014).';

TEXT TAXON = 3 CHARACTER = 309 TEXT = 'The hyoids were not recognized in the studied specimens and neither mentioned by Gow (1975) (MDE, 12 August 2013).';

TEXT TAXON = 13 CHARACTER = 309 TEXT = 'Based on Nisotti (2007, fig. 12) (MDE, 2 September 2012).';

TEXT TAXON = 32 CHARACTER = 309 TEXT = 'Based on SAM-PK-K10797 (MDE, 5 March 2014).';

TEXT TAXON = 10 CHARACTER = 310 TEXT = 'Gottman-Quesada and Sander (2009: 153) said that the presence of a notochordal canal in the vertebrae of the Protorosaurus specimens cannot be assessed. However, in the cast MB R2173 (original probably destroyed during WWII) the surface of the posterior facet of the centrum is concave and clearly not notochordal (MDE, 24 September 2012).';

TEXT TAXON = 24 CHARACTER = 310 TEXT = 'Based on Carroll (1976: 42) (MDE, 18 September 2012).';

TEXT TAXON = 12 CHARACTER = 313 TEXT = 'Based on PIMUZ T4355 (MDE, 2 June 2013).';

TEXT TAXON = 25 CHARACTER = 313 TEXT = 'Based on SAM-PK-6536 (MDE, 3 March 2014).';

TEXT TAXON = 38 CHARACTER = 314 TEXT = 'Based on SAM-PK-11208 (MDE, 5 September 2014).';

TEXT TAXON = 12 CHARACTER = 315 TEXT = 'Based on PIMUZ T2472 (picture 386) (MDE, 10 September 2012).';

TEXT TAXON = 32 CHARACTER = 315 TEXT = 'Based on BPI/1/2675 (MDE, 13 September 2012).';

TEXT TAXON = 38 CHARACTER = 315 TEXT = 'Based on SAM-PK-K140 and GHG 363 (MDE, 12 September 2012).';

TEXT TAXON = 12 CHARACTER = 316 TEXT = 'Although a centrodiapophyseal lamina seems to be present in PIMUZ T4822, the strongly compressed nature of the Macrocnemus specimens turns difficult to assess the condition of this character (MDE, 10 September 2012).';

TEXT TAXON = 32 CHARACTER = 316 TEXT = 'Based on BPI/1/2675 (MDE, 13 September 2012).';

TEXT TAXON = 12 CHARACTER = 317 TEXT = 'Based on PIMUZ T4822 (10 September 2012).';

TEXT TAXON = 32 CHARACTER = 317 TEXT = 'Based on BP/1/2675 (MDE, 13 September 2012).';

TEXT TAXON = 32 CHARACTER = 318 TEXT = 'Based on BPI/1/2675 (MDE, 13 September 2012).';

TEXT TAXON = 38 CHARACTER = 318 TEXT = 'SAM-PK-11208 has a thick postzygodiapophyseal lamina but other specimens (e.g. SAM-PK-K140 lack this lamina) (MDE, 12 September 2012).';

TEXT TAXON = 74 CHARACTER = 318 TEXT = 'Based on SAM-PK-6047B (MDE, 2 April 2014).';

TEXT TAXON = 32 CHARACTER = 320 TEXT = 'The posterior cervical and anterior dorsal vertebrae of BPI/1/2675 have mammillary processes on the lateral surface of the neural spines. Accordingly, the scoring of this character was changed from (0) to (1) (MDE, 14 September 2012).';

TEXT TAXON = 54 CHARACTER = 320 TEXT = 'Proterosuchids lack a transverse expansion of the neural spine posteriorly to the vertebrae with mammillary processes and, as a result, Koilamasuchus is scored with a question mark (MDE, 5 April 2014).';

TEXT TAXON = 3 CHARACTER = 324 TEXT = 'Based on Smith and Evans (1996) (MDE, 11 February 2014).';

TEXT TAXON = 19 CHARACTER = 324 TEXT = 'In the eighth postaxial vertebra the capitular rib faet is lost (Spielmann et al., 2008: 41) (MDE, 25 February 2014).';

TEXT TAXON = 25 CHARACTER = 324 TEXT = 'Based on SAM-PK-5882 and SAM-PK-6536 (MDE, 3 March 2014).';

TEXT TAXON = 1 CHARACTER = 325 TEXT = 'Based on Reisz (1981: 30) (MDE, 10 February 2014).';

TEXT TAXON = 56 CHARACTER = 326 TEXT = 'The atlantal intercentrum is preserved, based on unpublished photographs (MDE, 3 April 2015).';

TEXT TAXON = 14 CHARACTER = 327 TEXT = 'A keel is present on the axis of ZAR 07 (MDE, 2 May 2014).';

TEXT TAXON = 25 CHARACTER = 327 TEXT = 'Based on SAM-PK-5882 (MDE, 3 March 2014).';

TEXT TAXON = 26 CHARACTER = 327 TEXT = 'At least in the axis of SAM-PK-5885 (MDE, 3 September 2012).';

TEXT TAXON = 35 CHARACTER = 327 TEXT = 'Based on Benton & Allen (1997: 936) (MDE, 25 March 2014).';

TEXT TAXON = 64 CHARACTER = 327 TEXT = 'Based on SAM-PK-3028 (MDE, 24 March 2014).';

TEXT TAXON = 95 CHARACTER = 327 TEXT = 'Based on Wu and Russell (2001: 44) (MDE, 22 April 2014).';

TEXT TAXON = 40 CHARACTER = 330 TEXT = 'Based on a photograph in Cruickshank (1972), the neural spine of the axis is currently broken in NMQR 1484 (MDE, 11 May 2015).';

TEXT TAXON = 78 CHARACTER = 330 TEXT = 'The dorsal margin of the neural spine of the axis is damaged in PVL 4601 and PVL 4602 (MDE, 19 October 2014).';

TEXT TAXON = 81 CHARACTER = 330 TEXT = 'The dorsal margin of the neural spine of the axis is mostly straight in MCZ 4037 and dorsally convex in PVL 4575 (MDE, 19 October 2014).';

TEXT CHARACTER = 331 TEXT = 'Only_mammalimorphs,_Thrinaxodon,_crocodiles,_and_Eldeceeon_have_a_difference_between_thoracic_and_lumbar_vertebrae._(DM)';

TEXT TAXON = 1 CHARACTER = 331 TEXT = 'Ratio=2.02 in the fourth cervical vertebra based on Peabody (1952: fig. 3) (MDE, 18 September 2012).';

TEXT TAXON = 5 CHARACTER = 331 TEXT = 'The exact position of the cervical vertebra illustrated by Fraser and Walkden (1984: fig. 5) (MDE, 12 September 2012).';

TEXT TAXON = 6 CHARACTER = 331 TEXT = 'Ratio=ca. 1.22 based on Evans (1981: fig. 4c) (MDE, 21 September 2012).';

TEXT TAXON = 12 CHARACTER = 331 TEXT = 'Ratio: 3.76-3.83 in PIMUZ T4822 and this ratio is higher in larger specimens such as PIMUZ T4355 (MDE, 6 September 2012).';

TEXT TAXON = 13 CHARACTER = 331 TEXT = 'Ratio=14.25 in the fourth cervical of PIMUZ T2818 (MDE, 11 September 2012).';

TEXT TAXON = 19 CHARACTER = 331 TEXT = 'Some cervical vertebrae are longer then the posterior dorsals and others shorter (Spielman et al. 2008: appendix 10). Accordingly, the scoring of this character was changed from (0) to (0&1) (MDE, September 2012). Ratio=1.84-2.5 in the fourth and fifth cervical vertebrae based on Spielmann et al. (2008: appendix 10) (MDE, 13 September 2012).';

TEXT TAXON = 32 CHARACTER = 331 TEXT = 'Ratio=3.26 in the fourth cervical 3.68 in the fifth cervical vertebrae of BPI/1/2675 (MDE, 13 September 2012).';

TEXT TAXON = 1 CHARACTER = 332 TEXT = 'Based on Reisz (1981: 37) (MDE, 28 March 2015).';

TEXT TAXON = 5 CHARACTER = 332 TEXT = 'Based on Fraser and Walkden (1984: 576) (MDE, 3 March 2014).';

TEXT TAXON = 6 CHARACTER = 332 TEXT = 'Based on Evans (1981: 84) (MDE, 15 February 2014).';

TEXT TAXON = 7 CHARACTER = 332 TEXT = 'Based on Evans (1991: 190) (MDE, 5 April 2015).';

TEXT TAXON = 10 CHARACTER = 332 TEXT = 'The specimens are too transversely compressed to determine the condition of this character (MDE, 13 February 2014).';

TEXT TAXON = 13 CHARACTER = 332 TEXT = 'Based on Pritchard et al. (2015) (MDE, 28 March 2015).';

TEXT TAXON = 86 CHARACTER = 332 TEXT = 'The degree of separation between parapophyses and diapophyses is not clear because of deformation (SMNS 91083) (MDE, 28 April 2014).';

TEXT TAXON = 5 CHARACTER = 333 TEXT = 'Based on Fraser and Walkden (1984: 576) (MDE, 31 March 2015).';

TEXT TAXON = 13 CHARACTER = 336 TEXT = 'Based on PIMUZ T2189 (MDE, 10 March 2014).';

TEXT CHARACTER = 337 TEXT = 'In_Mesenosaurus_and_Heleosaurus,_only_the_cervicals_have_excavations_(Reisz_&_Modesto_2007:737).';

TEXT TAXON = 2 CHARACTER = 337 TEXT = 'The cervicals lack a depression or pit lateral to the base of the neural arch (MNHN 1908-32-57). Accordingly, the state of this character was changed from (1) to (0) (MDE, 11 August 2013).';

TEXT TAXON = 10 CHARACTER = 337 TEXT = 'The seventh cervical of WMsN P47361 has a shallow excaviation next to the base of the neural spine, but the more anterior cervical vertebrae lack such depression (MDE, 3 September 2012).';

TEXT TAXON = 26 CHARACTER = 337 TEXT = 'At least in the axis of SAM-PK-5885 (MDE, 3 September 2012).';

TEXT TAXON = 38 CHARACTER = 337 TEXT = 'The fifth to eight cervical vertebrae of Proterosuchus fergusi possess an excavation lateral to the neural spine, which is shallow in the middle cervicals and very deep in posterior cervicals, particularly in the eight cervical (BP/1/3993). These excavations are also present in Prolacerta broomi (MDE, 20 June 2012).';

TEXT TAXON = 32 CHARACTER = 338 TEXT = 'A transpostzygapophyseal lamina is present in the third cervical, but not in the fourth and fifth cervicals (BP/1/2675) (MDE, 2 April 2015).';

TEXT TAXON = 12 CHARACTER = 340 TEXT = 'Based on PIMUZ T4822 (MDE, 16 September 2012).';

TEXT TAXON = 13 CHARACTER = 340 TEXT = 'Based on PIMUZ T2818 (MDE, 11 September 2012).';

TEXT TAXON = 13 CHARACTER = 341 TEXT = 'Based on PIMUZ T2818 (MDE, 11 September 2012).';

TEXT TAXON = 26 CHARACTER = 341 TEXT = 'Based on the axis of SAM-PK-5885 (MDE, 10 September 2012).';

TEXT TAXON = 64 CHARACTER = 343 TEXT = 'Based on NHMUK R3592 (MDE, 12 September 2012).';

TEXT TAXON = 5 CHARACTER = 346 TEXT = 'Based on Fraser and Walkden (1984: 580) (MDE, 12 September 2012).';

TEXT TAXON = 12 CHARACTER = 346 TEXT = 'Intercentra are present in the cervical series of PIMUZ T4822, but absent in PIMUZ T2472 and T4355 (MDE, 11 September 2012).';

TEXT TAXON = 19 CHARACTER = 346 TEXT = 'Based on Spielmann et al. 2008 (fig. 30) (MDE, 13 September 2012).';

TEXT TAXON = 32 CHARACTER = 346 TEXT = 'Based on Gow (1975: 107) (MDE, 5 March 2014).';

TEXT TAXON = 65 CHARACTER = 346 TEXT = 'I am not sure if the elements described by Wang et al. (2013) in the neck of S. shansisuchus are actually intercentra. They are too large and overlap considerably the lateral surface of the centra (MDE, 27 March 2014).';

TEXT TAXON = 74 CHARACTER = 346 TEXT = 'Based on Ewer (1965: 406) (MDE, 31 March 2014).';

TEXT TAXON = 74 CHARACTER = 349 TEXT = 'Based on SAM-PK-13665 (MDE, 31 March 2014).';

TEXT CHARACTER = 350 TEXT = 'Not_sure_what_that_is._(DM)';

TEXT TAXON = 2 CHARACTER = 350 TEXT = 'Based on Currie (1980) (MDE, 6 February 2014).';

TEXT TAXON = 25 CHARACTER = 350 TEXT = 'Based on SAM-PK-5882 (MDE, 27 August 2012).';

TEXT TAXON = 35 CHARACTER = 350 TEXT = 'Based on Tatarinov (1978: 511) (MDE, 2 April 2015).';

TEXT TAXON = 38 CHARACTER = 350 TEXT = 'The presence of this accesory process is clear in BSPG 1934 VIII 514 (MDE, 20 June 2012).';

TEXT TAXON = 1 CHARACTER = 353 TEXT = 'Based on Reisz (1981: 33, 34) (MDE, 6 March 2014).';

TEXT TAXON = 2 CHARACTER = 353 TEXT = 'The condition of this character cannot be confidently determined in the holotype of Acerosodontosaurus (MNHN 1908-32-57). As a result, it is preferred here to score the character as (?) rather than (1) (MDE, 11 August 2013).';

TEXT TAXON = 13 CHARACTER = 353 TEXT = 'Based on SMNS 55341 (MDE, 09 August 2013).';

TEXT TAXON = 24 CHARACTER = 353 TEXT = 'Based on AM 3591 (MDE, 18 September 2012).';

TEXT TAXON = 28 CHARACTER = 353 TEXT = 'Based on NHMUK R1238 (MDE, 11 September 2014).';

TEXT TAXON = 38 CHARACTER = 353 TEXT = 'Scoring based on SAM-PK-K140 (MDE, 20 June 2012).';

TEXT TAXON = 64 CHARACTER = 353 TEXT = 'Based on NHMUK 3592 (MDE, 24 August 2012).';

TEXT TAXON = 9 CHARACTER = 354 TEXT = 'The condition cannot be determiend in most of the dorsal vertebrae (MDE, 10 March 2014).';

TEXT TAXON = 92 CHARACTER = 354 TEXT = 'Based on Walker (1964: 82) (MDE, 20 August 2014).';

TEXT TAXON = 12 CHARACTER = 355 TEXT = 'Although in at least one dorsal vertebra of PIMUZ T4822 there is a circular foramen in the lateral surface of the centrum, I couldn''t recognize this feature in any other vertebra of other specimens (MDE, 10 October 2012).';

TEXT TAXON = 65 CHARACTER = 356 TEXT = 'Based on Young (1964: fig. 21f) (MDE, 27 March 2014).';

TEXT TAXON = 1 CHARACTER = 357 TEXT = 'The ratio is lower than 0.5 based on Reisz (1981: 34) (MDE, 2 April 2013).';

TEXT TAXON = 2 CHARACTER = 357 TEXT = 'This ratio should be similar to Youngina because of the very short transverse processes (MDE, 6 February 2014).';

TEXT TAXON = 3 CHARACTER = 357 TEXT = 'Ratio=0.46 based on BP/1/3859 vertebrae 9?12 (MDE, 2 April 2013).';

TEXT TAXON = 5 CHARACTER = 357 TEXT = 'Ratio=0.18?0.25 based on Fraser and Walkden (1984: figs. 5, 6) (MDE, 2 April 2013).';

TEXT TAXON = 6 CHARACTER = 357 TEXT = 'Ratio=0.15?0.30 based on Evans (1981: figs. 5, 6) (MDE, 2 April 2013).';

TEXT TAXON = 10 CHARACTER = 357 TEXT = 'Ratio=0.38?0.45 based on postaxial vertebrae 8, 9 and 11 of BSPG 1995-I-5 cast of WMSN P47361 (MDE, 2 April 2013).';

TEXT TAXON = 12 CHARACTER = 357 TEXT = 'Ratio=0.56 based on PIMUZ T2472 (MDE, 2 April 2013).';

TEXT TAXON = 13 CHARACTER = 357 TEXT = 'Ratio=0.46 based on Wild (1973: fig. 52) (MDE, 3 April 2013).';

TEXT TAXON = 14 CHARACTER = 357 TEXT = 'Based on ZAR 08 (MDE, 2 May 2014).';

TEXT TAXON = 16 CHARACTER = 357 TEXT = 'Ratio=0.49 in UA 8-26-98-265 (MDE, 18 February 2014).';

TEXT TAXON = 19 CHARACTER = 357 TEXT = 'Ratio=0.84 based on Spielmann et al. (2009: fig. 37) (MDE, 3 April 2013).';

TEXT TAXON = 25 CHARACTER = 357 TEXT = 'Ratio=ca. 0.5 based on Dilkes (1998: 513) (MDE, 2 April 2013).';

TEXT TAXON = 28 CHARACTER = 357 TEXT = 'Based on SHYMS 2 (MDE, 11 September 2014).';

TEXT TAXON = 30 CHARACTER = 357 TEXT = 'Ratio=0.70 based on PIN 156/110 (MDE, 2 April 2013).';

TEXT TAXON = 32 CHARACTER = 357 TEXT = 'Ratio=0.55 based on BP/1/2675 vertebra 10 (MDE, 2 April 2013, 6 March 2014).';

TEXT TAXON = 64 CHARACTER = 357 TEXT = 'Ratio=0.85 based on NHMUK R3592 (MDE, 2 April 2013).';

TEXT TAXON = 65 CHARACTER = 357 TEXT = 'ratio=1.13 based on Young (1964: fig. 21f) (MDE, 27 March 2014).';

TEXT TAXON = 74 CHARACTER = 357 TEXT = 'Ratio=0.48 based on Ewer (1965: fig. 7g, h) (MDE, 2 April 2013).';

TEXT TAXON = 105 CHARACTER = 357 TEXT = 'Based on ZPAL Ab III 1930 (MDE, April 23 2015)';

TEXT CHARACTER = 358 TEXT = 'Quantify._(DM)Cannot_from_this_end.R';

TEXT TAXON = 13 CHARACTER = 358 TEXT = 'Based on PIMUZ T2818 (MDE, 2 September 2012).';

TEXT TAXON = 24 CHARACTER = 358 TEXT = 'Based on AM 3591 (MDE, 18 September 2012).';

TEXT TAXON = 28 CHARACTER = 359 TEXT = 'Based on BATGM M20a (MDE, 30 October 2014).';

TEXT TAXON = 38 CHARACTER = 359 TEXT = 'Based on SAM-PK-K140 y GHG 363 (MDE, 5 September 2014).';

TEXT TAXON = 57 CHARACTER = 359 TEXT = 'Hyposphene is present in the ninth presacral and an isolated dorsal neural arch (MDE, 13 March 2014).';

TEXT TAXON = 64 CHARACTER = 359 TEXT = 'Based on NHMUK R3592 (MDE, 24 March 2014).';

TEXT TAXON = 94 CHARACTER = 359 TEXT = 'Based on Nesbitt et al. (2014: 1364) (MDE, 11 November 2014).';

TEXT TAXON = 28 CHARACTER = 360 TEXT = 'Based on BATGM M20a (MDE, 30 October 2014).';

TEXT TAXON = 24 CHARACTER = 361 TEXT = 'Dilkes (1998: 530) reported the absence of pits lateral to the neural spines in Noteosuchus. This condition is right for the preserved posterior dorsal vertebrae of AM 3591, but it could not be assessed in the anterior dorsal vertebrae. Although the pits are prensent in the posterior dorsal vertebrae of Howesia (SAM-PK-5886), in Mesosuchus the posterior dorsal vertebrae also lack of the pit, but it is present in the anterior dorsal vertebrae (SAM-PK-6046). Accordingly, the condition of this character is scored as missing data in Noteosuchus (MDE, 18 September 2012).';

TEXT TAXON = 11 CHARACTER = 363 TEXT = 'Based on SMNS 54783 (MDE, 22 September 2014).';

TEXT TAXON = 80 CHARACTER = 364 TEXT = 'Based on Dilkes and Arcucci (2012) character 55 (MDE, 18 August 2014).';

TEXT TAXON = 1 CHARACTER = 365 TEXT = 'Based on Reisz (1981: 34) (MDE, 12 February 2014).';

TEXT TAXON = 3 CHARACTER = 366 TEXT = 'Based on Gow (1975: 95) (MDE, 15 October 2012).';

TEXT TAXON = 11 CHARACTER = 366 TEXT = 'There is an intercentrum at least between the last two dorsals in SMNS 90600 (MDE, 29 August 2014).';

TEXT TAXON = 35 CHARACTER = 366 TEXT = 'Intercentra were probably absent in the dorsal series, but the condition cannot be determined confidently (MDE, April 12 2015).';

TEXT TAXON = 74 CHARACTER = 366 TEXT = 'Based on Ewer (1965: 406) (MDE, 31 March 2014).';

TEXT TAXON = 2 CHARACTER = 368 TEXT = 'Currie (1980) described that the dorsal ribs are holochepahlous and agrees with personal observation of MNHN 1908-32-57 (MDE, 6 February 2014).';

TEXT TAXON = 10 CHARACTER = 368 TEXT = 'Based on Gottman-Quesada and Sander (2009: 158) (MDE, 3 September 2012).';

TEXT TAXON = 13 CHARACTER = 368 TEXT = 'Based on Wild (1973: fig. 35) (MDE, 20 June 2012).';

TEXT TAXON = 8 CHARACTER = 369 TEXT = 'Based on MNHN.F.BR12154 (MDE, April 10 2015).';

TEXT TAXON = 5 CHARACTER = 370 TEXT = 'Second sacral bifurcated, contrasting with the first one (Fraser Walkden, 1984) (MDE, 12 August 2013).';

TEXT TAXON = 12 CHARACTER = 370 TEXT = 'I have observed the presence of a dorsosacral vertebra in which a rib contacts the medial surface of the anterior end of the iliac blade in PIMUZ T2472 and T4822 (MDE, 30 August 2012).';

TEXT TAXON = 19 CHARACTER = 370 TEXT = 'Trilophosaurus buettneri has two unequal sacral vertebrae (Spielmann et al. 2008) and, as a result, the scoring of this character was changed from (?) to (0) (MDE, 4 September 2012).';

TEXT TAXON = 95 CHARACTER = 370 TEXT = 'Based on Wu and Russell (2001: 45) (MDE, 22 April 2014).';

TEXT TAXON = 102 CHARACTER = 371 TEXT = 'Based on Bonaparte (1975: fig. 6) (MDE, 19 April 2014).';

TEXT TAXON = 10 CHARACTER = 373 TEXT = 'Based on SMNS 55387, cast of the Simon Bartholomeus specimen (MDE, 13 February 2014).';

TEXT TAXON = 12 CHARACTER = 373 TEXT = 'Based on PIMUZ T2472 (MDE, 10 September 2012).';

TEXT TAXON = 21 CHARACTER = 373 TEXT = 'Based on a second primordial sacral previously referred to Trilophosaurus jacobsi (Spielmann et al. 2009: 285) (Spielmann et al. 2008, fig. 96) (MDE, 23 August 2016).';

TEXT TAXON = 24 CHARACTER = 373 TEXT = 'Based on AM 3591 (MDE, 18 September 2012).';

TEXT TAXON = 25 CHARACTER = 373 TEXT = 'Based on Dilkes (1998: character 87) (MDE, 11 September 2012).';

TEXT TAXON = 26 CHARACTER = 373 TEXT = 'Based on Dilkes (1998: character 87) and SAM-PK-5886 (MDE, 10 September 2012, 7 March 2014).';

TEXT TAXON = 28 CHARACTER = 373 TEXT = 'Based on SHYMS 5 (MDE, 11 September 2014).';

TEXT TAXON = 74 CHARACTER = 373 TEXT = 'The sacral rib of the second sacral of Euparkeria is not bifurcated (SAM-PK-7696) (MDE, 11 September 2012).';

TEXT TAXON = 76 CHARACTER = 373 TEXT = 'Based on Trotteyn (2011: 433) (MDE, 14 April 2014).';

TEXT TAXON = 10 CHARACTER = 375 TEXT = 'Based on Gottman-Quesada and Sander (2004: 159) (MDE, 10 October 2012).';

TEXT TAXON = 105 CHARACTER = 380 TEXT = 'Based on ZPAL Ab III 1975 (MDE, 30 April 2015).';

TEXT TAXON = 1 CHARACTER = 381 TEXT = 'Based on Reisz (1981: 36) (MDE, 12 February 2014).';

TEXT TAXON = 105 CHARACTER = 381 TEXT = 'Based on ZPAL Ab III 1975 (MDE, 30 April 2015).';

TEXT TAXON = 6 CHARACTER = 382 TEXT = 'Based on Evans (1981: fig. 11b) (MDE, 21 September 2012).';

TEXT TAXON = 12 CHARACTER = 382 TEXT = 'Based on PIMUZ T2472 (MDE, 22 September 2014).';

TEXT TAXON = 13 CHARACTER = 382 TEXT = 'Based on PIMUZ T2817 (MDE, 11 September 2012).';

TEXT TAXON = 1 CHARACTER = 383 TEXT = 'Based on Peabody (1952: 22) and Reisz (1981: 4) (MDE, 12 February 2014).';

TEXT TAXON = 3 CHARACTER = 383 TEXT = 'Based on Smith and Evans (1996: fig. 4c, d) (MDE, 3 September 2013).';

TEXT TAXON = 6 CHARACTER = 383 TEXT = 'Based on Evans (1981: 91) (MDE, 10 October 2012).';

TEXT TAXON = 19 CHARACTER = 383 TEXT = 'Based on the scoring of Pritchard et al. (2015: character 137) (MDE, 29 March 2015).';

TEXT TAXON = 26 CHARACTER = 383 TEXT = 'Based on Dilkes (1995: 676) (MDE, 7 March 2014).';

TEXT TAXON = 107 CHARACTER = 383 TEXT = 'Based on PVL 2054 (MDE, 26 August 2014).';

TEXT TAXON = 57 CHARACTER = 384 TEXT = 'The type specimen of Sarmatosuchus is not a mature individual (MDE, 13 March 2014).';

TEXT TAXON = 28 CHARACTER = 385 TEXT = 'Based on NHMUK R1239 (MDE, 12 September 2014).';

TEXT TAXON = 94 CHARACTER = 385 TEXT = 'The condition of this character cannot be confidently determined because the anterior margin of the coracoid is misssing (MDE, 11 November 2014).';

TEXT TAXON = 26 CHARACTER = 387 TEXT = 'The scapular blade of SAM-PK-5885 was apparently damaged (MDE, 7 March 2014).';

TEXT TAXON = 13 CHARACTER = 390 TEXT = 'The anterior margin of the scapular blade is not distintly concave, but neither continuously convex (MDE, 12 March 2014).';

TEXT TAXON = 26 CHARACTER = 390 TEXT = 'The scapular blade of SAM-PK-5885 was apparently damaged (MDE, 7 March 2014).';

TEXT TAXON = 6 CHARACTER = 392 TEXT = 'Based on Evans (1981: 95) (MDE, 21 September 2012).';

TEXT TAXON = 26 CHARACTER = 392 TEXT = 'Based on Broom (1906: plate 15) (MDE, 3 September 2012). ';

TEXT TAXON = 32 CHARACTER = 392 TEXT = 'The supraglenoid foramen is absent in BP/1/2675. Accordingly, the scoring of this character was changed from (?) to (0) (MDE, 11 August 2013).';

TEXT TAXON = 35 CHARACTER = 397 TEXT = 'Based on Tatarinov (1978: 511) (MDE, 25 March 2014).';

TEXT TAXON = 3 CHARACTER = 404 TEXT = 'The presence of cleithrum is uncertain in Youngina capensis (Smith and Evans, 1996: 296) (MDE, 3 September 2013).';

TEXT TAXON = 10 CHARACTER = 404 TEXT = 'The presence of a cleithrum is ambiguous in Protorosaurus Gottman-Quesada and Sander (2009) (MDE, 3 September 2012).';

TEXT TAXON = 64 CHARACTER = 404 TEXT = 'Since none of the preserved specimens of Erythrosuchus possess an articulated pectoral girdle I consider that the presence or absence of a cleithrum cannot be determined (MDE, 24 August 2012).';

TEXT TAXON = 35 CHARACTER = 405 TEXT = 'Based on Tatarinov (1978: 511) (MDE, 25 March 2014).';

TEXT TAXON = 26 CHARACTER = 406 TEXT = 'Based on Broom (1906: plate 15) (MDE, 3 September 2012). ';

TEXT TAXON = 32 CHARACTER = 406 TEXT = 'The interclavicle of Prolacerta lacks an anterior process (BP/1/2675). Accordingly, the scoring of this character was changed from (0) to (1) (MDE, 11 August 2013).';

TEXT TAXON = 74 CHARACTER = 406 TEXT = 'I consider that the anterior portion of the interclavicle of the holotype specimen is not enough preserved to assessed the condition of the character (MDE, 24 August 2012).';

TEXT TAXON = 14 CHARACTER = 407 TEXT = 'Based on ZAR 06 (2 May, 2014)';

TEXT TAXON = 38 CHARACTER = 407 TEXT = 'Based on GHG 363 (MDE, 12 September 2012).';

TEXT TAXON = 3 CHARACTER = 409 TEXT = 'The anterior half of the interclavicle is T-shaped, with sharp angles between lateral and posterior processes (Gow, 1975: 9c; Smith and Evans, 1996: 6c) (MDE, 3 September 2013).';

TEXT TAXON = 19 CHARACTER = 409 TEXT = 'Gregory_1945:_pl._26._(DM)';

TEXT TAXON = 25 CHARACTER = 409 TEXT = 'Although DIlkes (1998) described the interclavicle of Mesosuchus as T-shaped its anterior end is diamond-shaped (MDE, 27 August 2012).';

TEXT TAXON = 26 CHARACTER = 409 TEXT = 'Based on Broom (1906: plate 15) (MDE, 3 September 2012). ';

TEXT TAXON = 35 CHARACTER = 409 TEXT = 'Based on Tatarinov (1978: 511) (MDE, 25 March 2014).';

TEXT TAXON = 74 CHARACTER = 409 TEXT = 'I consider that the anterior portion of the interclavicle of the holotype specimen is not enough preserved to assessed the condition of the character (MDE, 24 August 2012).';

TEXT CHARACTER = 410 TEXT = 'Was_"Interclavicle_wide_(0),_narrow_(1)"._The_coding_was_incomprehensible._So_I_made_several_characters_of_it._(DM)';

TEXT TAXON = 5 CHARACTER = 410 TEXT = 'The posterior ramus of the interclavicle is not known (Fraser and Walkden 1984) (MDE, 12 August 2013).';

TEXT TAXON = 3 CHARACTER = 412 TEXT = 'Based on Gow (1975: 95) (MDE, 15 October 2012).';

TEXT TAXON = 6 CHARACTER = 412 TEXT = 'Evans (1981: fig. 15) clearly shows that the clavicle articulates with the ventral surface of the interclavicle (MDE, 10 October 2012).';

TEXT TAXON = 12 CHARACTER = 412 TEXT = 'Based on PIMUZ T4355 (MDE, 10 October 2012).';

TEXT TAXON = 19 CHARACTER = 412 TEXT = 'Based on Gregory (1945: 301) (MDE, 13 November 2012).';

TEXT TAXON = 3 CHARACTER = 413 TEXT = 'The presence of mineralized sternal plates in Youngina was confirmed by Smith and Evans (1996) (MDE, 4 September 2013).';

TEXT TAXON = 35 CHARACTER = 413 TEXT = 'Based on Tatarinov (1978: 511) (MDE, 25 March 2014).';

TEXT TAXON = 64 CHARACTER = 413 TEXT = 'Since none of the preserved specimens of Erythrosuchus possess an articulated pectoral girdle I consider that the presence or absence of a mineralized sternum cannot be determined (MDE, 24 August 2012).';

TEXT TAXON = 106 CHARACTER = 413 TEXT = 'The bones interpreted by Sereno (2012) as sternal plates are likely uncinate processes (MDE, 27 August 2014).';

TEXT TAXON = 3 CHARACTER = 414 TEXT = '50.9 mm estimated forelimb in SAM-PK-K7710a based on BP/1/3859. 67 hindlimb of SAM-PK-K710a. Ratio= 0.76 (MDE, 22 September 2014).';

TEXT TAXON = 10 CHARACTER = 414 TEXT = 'Length of the forelimb of BSPG 1995 I 5, cast of WMsN P 47361: 203.8 mm. Lenth of the hindlimb of BSPG 1995 I 5, cast of WMsN P 47361, based on extrapolations with NHMW 1974 1635: 318.3 mm. Ratio: 0.64 (MDE, 22 September 2014).';

TEXT TAXON = 3 CHARACTER = 415 TEXT = 'Based on BPI/1/3859 (MDE, 15 October 2012).';

TEXT TAXON = 12 CHARACTER = 415 TEXT = 'The condition of the character is not clear in the available specimens due to the strong degree of compression of the materials (MDE, 10 September 2012).';

TEXT TAXON = 76 CHARACTER = 422 TEXT = 'Trotteyn (2011) described that the shape of the deltopectoral crest cannot be determined because of postmortem deformation (MDE, 14 April 2014).';

TEXT CHARACTER = 425 TEXT = 'Quantify._(DM)';

TEXT TAXON = 5 CHARACTER = 425 TEXT = 'In Planocephalosaurus the entepicondyle is strongly developed in comparison with the shaft width at mid-length (Fraser and Walkden 1984: fig. 14). Accordingly, the scoring of this character was changed from (0) to (1) (MDE, 14 September 2012).';

TEXT TAXON = 9 CHARACTER = 425 TEXT = 'The condition seems to be more similar to that of Youngina capensis (BP/1/3859) than to that of Prolacerta broomi (BP/1/2675) (MDE, 20 June 2012).';

TEXT TAXON = 10 CHARACTER = 425 TEXT = 'The degree of development of the entepicondyle in Protorosaurus is more similar to Youngina than to Prolacerta (WMsN P47361) (MDE, 3 September 2012).';

TEXT CHARACTER = 427 TEXT = 'I_have_exchanged_states_0_and_1_so_the_character_can_be_ordered_(absent-groove-foramen)._(DM)';

TEXT TAXON = 3 CHARACTER = 427 TEXT = 'A supinator process and an ectopicondylar groove are present is Youngina (BP/1/3859). Accordingly, the scoring of this character was changed from (?) to (1) (MDE, 11 August 2013).';

TEXT TAXON = 74 CHARACTER = 427 TEXT = 'In the holotype of Euparkeria there is no supinator ridge neither a deep groove in the distal end of the right humerus (MDE, 24 August 2012).';

TEXT TAXON = 2 CHARACTER = 428 TEXT = 'Based on MNHN 1908-32-57 (MDE, 2 June 2013).';

TEXT TAXON = 10 CHARACTER = 428 TEXT = 'IN BSPG 1995 I 5 there are a well-developed condyle next to the ectepicondyle and it is very likely that its degree of development is understimated due to the strong dorsoventral degree of compression that suffered the bone. The same condition or even more developed distal condyles are observed in other individuals and in BSPG AS VII 1207 there is evidence of at least three distinct distal condyles (MDE, 13 September 2012).';

TEXT TAXON = 25 CHARACTER = 428 TEXT = 'Based on SAM-PK-6536 (MDE, 11 September 2012).';

TEXT TAXON = 26 CHARACTER = 428 TEXT = 'The condition of this character cannot be confidently assessed based on Broom (1906) drawing and the humerus is currently lost (DIlkes 1998) (MDE, 11 September 2012).';

TEXT TAXON = 3 CHARACTER = 430 TEXT = 'Youngina lacks an olecranon process in the ulna (Gow 1975: fig. 9a) (MDE, 11 August 2013).';

TEXT TAXON = 6 CHARACTER = 430 TEXT = 'Based on Evans (1981: 97) and the presence of a distinct and large posterior depression in the distal end of the humerus (MDE, 21 September 2012).';

TEXT TAXON = 19 CHARACTER = 430 TEXT = 'In Trilophosaurus buettneri the olecranon process is prominent but it is not ossified separately (Spielmann et al. 2008: figs. 69, 70). As a result, the scoring of this character was changed from (0) to (1) (MDE, 5 September 2012).';

TEXT TAXON = 25 CHARACTER = 433 TEXT = 'Based on SAM-PK-6046 (MDE, 4 March 2014).';

TEXT TAXON = 3 CHARACTER = 434 TEXT = 'The distal end is concave in BP/1/3859, but possibly because of the lack of complete ossification of the distal end (MDE, 11 February 2014).';

TEXT CHARACTER = 435 TEXT = 'Rationale_for_new_limits_explained_in_spreadsheet_(ML).';

TEXT TAXON = 3 CHARACTER = 436 TEXT = 'Based on Gow (1975: 97) (MDE, 15 October 2012).';

TEXT TAXON = 12 CHARACTER = 436 TEXT = 'Based on PIMUZ T4355 (MDE, 10 October 2012).';

TEXT TAXON = 13 CHARACTER = 436 TEXT = 'Based on Nosotti (2007: table 4, 6) (MDE, 11 October 2012).';

TEXT TAXON = 13 CHARACTER = 440 TEXT = 'Based on Nosotti (2007: 30, fig. 23) (MDE, 12 March 2014).';

TEXT TAXON = 38 CHARACTER = 440 TEXT = 'Based on SAM-PK-K140 (MDE, 6 September 2014).';

TEXT TAXON = 3 CHARACTER = 441 TEXT = 'Based on Gow (1975: 95) (MDE, 29 March 2015).';

TEXT TAXON = 13 CHARACTER = 441 TEXT = 'Based on Nosoti (2007: fig. 23) (MDE, 20 June 2012).';

TEXT TAXON = 13 CHARACTER = 442 TEXT = 'Based on Nosoti (2007: fig. 23) (MDE, 20 June 2012).';

TEXT TAXON = 107 CHARACTER = 444 TEXT = 'This scorings is based on the reinterpretation of the distal carpals preserved in PVSJ 373 as dc2-5 instead of dc1-4 (MDE, April 13 2015).';

TEXT TAXON = 10 CHARACTER = 445 TEXT = 'Based on BSPG 1995 I 5 (MDE, 10 September 2012).';

TEXT TAXON = 19 CHARACTER = 445 TEXT = 'In TMM 31025-140 the lengh of manus is 16.8 cm based on Spielmann et al. (2008: fig. 74) and length of humerus is 17.0-17.6 based on Spielmann et al. (2008: appendix 10) (MDE, 13 September 2012).';

TEXT TAXON = 25 CHARACTER = 445 TEXT = 'No specimen preserves both complete humerus and manus (MDE, 11 September 2012).';

TEXT TAXON = 72 CHARACTER = 447 TEXT = 'Based on Nesbitt et al. (2009: 831) (MDE, 27 April 2014).';

TEXT TAXON = 74 CHARACTER = 450 TEXT = 'Based on SAM-PK-13666 (MDE, 11 October 2012).';

TEXT TAXON = 74 CHARACTER = 452 TEXT = 'Based on SAM-PK-13666 (MDE, 31 March 2014).';

TEXT TAXON = 28 CHARACTER = 454 TEXT = 'Based on SHYMS 6 (MDE, 12 Septemebr 2014).';

TEXT TAXON = 35 CHARACTER = 454 TEXT = 'Based on Tatarinov (1978: 511) (MDE, 25 March 2014).';

TEXT TAXON = 3 CHARACTER = 455 TEXT = 'The pelvic girdle is solid in Youngina (BP/1/3859). Accordingly the scoring of this character was changed from (1) to (0) (MDE, 11 August 2013).';

TEXT TAXON = 5 CHARACTER = 455 TEXT = 'The acetabulum is completely closed (Fraser and Walkden 1984: fig. 16) (MDE, 12 August 2013).';

TEXT TAXON = 12 CHARACTER = 455 TEXT = 'The acetabular wall appears to be partially open in PIMUZ T4822 probably because of lack of preservation (MDE, 18 February 2014).';

TEXT TAXON = 94 CHARACTER = 456 TEXT = 'Based on the orientation of the sacral rib facets and in agreement with the scoring of Nesbitt et al. (2014: character 270) (MDE, 11 November 2014).';

TEXT TAXON = 93 CHARACTER = 462 TEXT = 'In PVL 3828 there is a crest cofluent with the anterior border of the preacetabular process, but it is absent in PVL 3826 (MDE, 20 August 2014).';

TEXT TAXON = 10 CHARACTER = 463 TEXT = 'Based on Gottman-Quesada and Sander (2009:165) (MDE, 10 September 2012).';

TEXT TAXON = 10 CHARACTER = 464 TEXT = 'Based on Gottman-Quesada and Sander (2009: 165) (MDE, 26 February 2014).';

TEXT TAXON = 93 CHARACTER = 465 TEXT = 'The depression on the ventral surface of the base of the postacetabular process in PVL 3827 does not reach the distal end of the process, contrasting with the condition in dinosauriforms (MDE, 20 August 2014).';

TEXT TAXON = 105 CHARACTER = 466 TEXT = 'Based on ZPAL Ab III 364 (MDE, April 23 2015)';

TEXT TAXON = 3 CHARACTER = 471 TEXT = 'Based on Smith and Evans (1996: 297) (MDE, 3 September 2013).';

TEXT TAXON = 10 CHARACTER = 471 TEXT = 'In the Simon/Bartholomaeus specimen the thyroid fenestra is absent (SMNS cast, MDE pers. obs.) (MDE, 9 August 2013)';

TEXT TAXON = 19 CHARACTER = 471 TEXT = 'Based on Gregory (1945: fig. 9) (MDE, 25 February 2014).';

TEXT TAXON = 24 CHARACTER = 471 TEXT = 'Based on Carroll (1976: 47) (MDE, 10 October 2012).';

TEXT TAXON = 92 CHARACTER = 473 TEXT = 'Based on NHMUK R2410 and Walker 1964: fig. 11g (MDE, 20 August 2014).';

TEXT TAXON = 93 CHARACTER = 473 TEXT = 'The pubis has a groove in PVL 3827 (MDE, 20 August 2014).';

TEXT TAXON = 1 CHARACTER = 474 TEXT = 'Based on Reisz (1981: 42): a second, smaller process extends ventrolaterally from the ridge connecting the lateral pubic tubercle to the acetabulum. This process ... probably served as the origin of the ambiens and pubotibialis muscles (MDE, 12 February 2014).';

TEXT TAXON = 28 CHARACTER = 477 TEXT = 'Based on BATGM M20a, b (MDE, 30 October 2014).';

TEXT TAXON = 3 CHARACTER = 479 TEXT = 'Based on Smith and Evans (1996: 297) (MDE, 3 September 2013).';

TEXT TAXON = 105 CHARACTER = 485 TEXT = 'Based on Nesbitt (2011: 140) (MDE, 24 April 2014).';

TEXT TAXON = 10 CHARACTER = 488 TEXT = 'Based on Simon and Bartholomaeus specimen (MDE, 31 March 2015).';

TEXT TAXON = 10 CHARACTER = 490 TEXT = 'The humeri and femora are strongly compressed and the diameter of the bones cannot be measured (MDE, 3 September 2012).';

TEXT TAXON = 10 CHARACTER = 491 TEXT = 'Based on BSPG AS VII 1207 (MDE, 13 February 2014).';

TEXT TAXON = 85 CHARACTER = 502 TEXT = 'Based on MCZ 4077 (MDE, 28 April 2014).';

TEXT TAXON = 85 CHARACTER = 503 TEXT = 'Based on MCZ 4077 (MDE, 28 April 2014).';

TEXT TAXON = 85 CHARACTER = 504 TEXT = 'Based on MCZ 4077 (MDE, 28 April 2014).';

TEXT TAXON = 85 CHARACTER = 505 TEXT = 'Based on MCZ 4077 (MDE, 28 April 2014).';

TEXT TAXON = 85 CHARACTER = 506 TEXT = 'Based on MCZ 4077 (MDE, 28 April 2014).';

TEXT TAXON = 3 CHARACTER = 508 TEXT = 'BP/1/3859 was prepared with acid and, as a result, the thickness of the bone wall may look artificially thinner (MDE, 11 February 2014).';

TEXT TAXON = 85 CHARACTER = 508 TEXT = 'Based on MCZ 4077 (MDE, 28 April 2014).';

TEXT TAXON = 94 CHARACTER = 508 TEXT = 'Based on Nesbitt et al. (2014: character 323) (MDE, 11 November 2014).';

TEXT TAXON = 10 CHARACTER = 509 TEXT = 'Based on SMNS 55387 cast of Simon and Bartholomeus specimen (MDE, 13 February 2014).';

TEXT TAXON = 25 CHARACTER = 509 TEXT = 'Based on the femur of SAM-PK-7416 (MDE, 11 September 2012).';

TEXT CHARACTER = 510 TEXT = 'I_changed_this_character._Petrolacosaurus_clearly_has_a_chunky_broad_femur,_as_does_Youngina._4/1_is_the_new_ratio,_not_3/1_(RR)._Problem:_then_Youngina_has_1_and_Hyperodapedon_and_Apsisaurus_0._State_1_redefined_slightly_(ML).';

TEXT TAXON = 86 CHARACTER = 510 TEXT = 'The femur of SMNS 91002 seems to be strongly artificially compressed (MDE, 28 April 2014).';

TEXT TAXON = 10 CHARACTER = 511 TEXT = 'Based on SMNS 55387 cast of Simon Bartholomaeus specimen (MDE, 12 March 2014).';

TEXT TAXON = 13 CHARACTER = 511 TEXT = 'Based on SMNS 54626 (MDE, 12 March 2014).';

TEXT TAXON = 94 CHARACTER = 523 TEXT = 'Probably miscored by Nesbitt et al. (2014: character 337) (MDE, 11 November 2014).';

TEXT TAXON = 94 CHARACTER = 529 TEXT = 'Although scored as a low crest-shaped structure by Nesbitt et al. (2014: character 339), the tubercle is considerably better developed than in non-pseudosuchian archosauromorphs (MDE, 11 November 2014).';

TEXT TAXON = 3 CHARACTER = 533 TEXT = 'It seems that it was present in adult specimens (Broom, 1921; Smith and Evans, 1996: fig. 8d) (MDE, 3 September 2013).';

TEXT TAXON = 13 CHARACTER = 533 TEXT = 'Based on Wild (1974: 116) and Nosotti (2007: 35) (MDE, 11 October 2012).';

TEXT TAXON = 26 CHARACTER = 535 TEXT = 'The proximal tarsals are expoased in posterior view in the only specimen that preserves the hindlimbs (MDE, April 7 2015)';

TEXT TAXON = 28 CHARACTER = 535 TEXT = 'The better preserved proximal tarsals are preserved in posterior view (MDE, April 8 2015).';

TEXT TAXON = 94 CHARACTER = 536 TEXT = 'The condition of this character is extremely similar to that of Smilosuchus and Parasuchus (MDE, 10 November 2014).';

TEXT TAXON = 98 CHARACTER = 536 TEXT = 'Inferred from the morphology of the distal end of tibia (MDE, 9 October 2014)';

TEXT TAXON = 102 CHARACTER = 536 TEXT = 'The supposed second basin on the astragalus, if present, is placed posterolateral rather than posteromedial (PVL 3871), contrasting with Riojasuchus, suchians and Lagerpetidae (MDE, 20 October 2014).';

TEXT TAXON = 108 CHARACTER = 537 TEXT = 'Inferred from tibial morphology (MDE, April 14 2015).';

TEXT TAXON = 10 CHARACTER = 539 TEXT = 'Based on Gottman-Quesada and Sander (2009: fig. 25c) (MDE, 13 February 2014).';

TEXT TAXON = 65 CHARACTER = 539 TEXT = 'The identification of the posterior groove by Gower (1996) was tentative (MDE, 27 March 2014).';

TEXT TAXON = 25 CHARACTER = 541 TEXT = 'Based on SAM-PK-7416 (MDE, 23 September 2014).';

TEXT TAXON = 74 CHARACTER = 541 TEXT = 'Nesbitt (2011) scored Euparkeria as (1), but the posterior portion of the dorsolateral margin of the astragalus overlaps the calcaneum to the same extent as the anterior portion (UMCZ T692) (MDE, 25 September 2014).';

TEXT TAXON = 6 CHARACTER = 542 TEXT = 'The astragalus possesses a deep, ventrally opened notch in anterior or posterior view to receive the distal tarsal 4 (see Evans, 1981: fig. 27a, b) (MDE, 17 February 2014).';

TEXT TAXON = 13 CHARACTER = 542 TEXT = 'Based on Nosotti (2007: 72) (MDE, 13 August 2013).';

TEXT TAXON = 19 CHARACTER = 545 TEXT = 'Gregory (1945: 316) described the absence of a strong "tuber calcis" in Trilophosaurus (MDE, 24 February 2014).';

TEXT TAXON = 24 CHARACTER = 545 TEXT = 'Based on Carroll (1976: 48) (MDE, 18 September 2012).';

TEXT TAXON = 25 CHARACTER = 545 TEXT = 'Although a calcaneal tuber was scored as absent in Mesosuchus by Dilkes (1998: character 119) it can be observed in the right hindlimb of SAM-PK-7416 (MDE, 27 August 2012).';

TEXT TAXON = 26 CHARACTER = 545 TEXT = 'Based on SAM-PK-5886 (MDE, 3 September 2012).';

TEXT TAXON = 105 CHARACTER = 545 TEXT = 'The calcaneal tuber is incipient in Silesaurus (ZPAL Ab III 361) (MDE, April 23 2015)';

TEXT TAXON = 24 CHARACTER = 556 TEXT = 'The reconstruction of the ankle of Noteosuchus by Carroll (1976) looks rather weird (MDE, 17 September 2014).';

TEXT TAXON = 10 CHARACTER = 557 TEXT = 'Based on SMNS 55387, cast of the Simon Bartholomaeus specimen, and Gottman-Quesada and Sanger (2009: figs. 25) (MDE, 10 September 2012).';

TEXT TAXON = 12 CHARACTER = 557 TEXT = 'Based on Rieppel (1989: 380) (MDE, 18 February 2014).';

TEXT TAXON = 24 CHARACTER = 557 TEXT = 'I consider that cannot be confidently assessed that the central did not contact the tibia (MDE, 18 September 2012).';

TEXT TAXON = 86 CHARACTER = 560 TEXT = 'The bones interpreted by Schoch and Sues (2013) as astragalus and calcaneus are reinterpreted as distal tarsals 3 and 4 (MDE, 28 April 2014).';

TEXT TAXON = 3 CHARACTER = 563 TEXT = 'Based on Broom (1921) (Smith and Evans: 1996: fig. 8d) (MDE, 3 September 2013).';

TEXT TAXON = 10 CHARACTER = 563 TEXT = 'It would be possible that the distal tarsal 5 is fused to the distal tarsal 4 (Gottman-Quesada and Sander (2009: 172) (MDE, 3 September 2012).';

TEXT TAXON = 24 CHARACTER = 563 TEXT = 'Based on Carroll (1976: 48) (MDE, 18 September 2012).';

TEXT TAXON = 1 CHARACTER = 564 TEXT = 'Based on Peabody (1952: fig. 6a) (MDE, 12 February 2014).';

TEXT TAXON = 3 CHARACTER = 564 TEXT = 'The metatarsal IV + digit 4 are considerably longer than the tibia (Smith and Evans, 1996: table 1) (MDE, 4 September 2013).';

TEXT TAXON = 12 CHARACTER = 564 TEXT = 'Based on PIMUZ T4822 (MDE, 18 February 2014).';

TEXT TAXON = 13 CHARACTER = 564 TEXT = 'Based on PIMUZ T2817 (MDE, 2 September 2012).';

TEXT TAXON = 25 CHARACTER = 564 TEXT = 'The distal phalanges of the fourth pedal digit are not preserved (MDE, 27 August 2012).';

TEXT TAXON = 28 CHARACTER = 564 TEXT = 'Based on SHYMS 5 (MDE, 12 September 2014).';

TEXT TAXON = 38 CHARACTER = 564 TEXT = 'The complete length of the fourth digit is not known in Proterosuchus specimens (MDE, 20 June 2012).';

TEXT TAXON = 3 CHARACTER = 566 TEXT = 'The metatarsals overlap proximally with each other (BP/1/3859). Accordingly the scoring of this character was changed from (?) to (1) (MDE, 11 August 2013).';

TEXT TAXON = 24 CHARACTER = 566 TEXT = 'Based on Carroll (1976: 48) (MDE, 18 September 2012).';

TEXT TAXON = 10 CHARACTER = 576 TEXT = 'Based on Simon-Bartholomaeus specimen cast SMNS 55387 (MDE, 23 April 2014).';

TEXT TAXON = 25 CHARACTER = 576 TEXT = 'Based on SAM-PK-7416 (MDE, 23 April 2014).';

TEXT TAXON = 26 CHARACTER = 576 TEXT = 'Based on Carroll (1976: fig. 8).';

TEXT TAXON = 41 CHARACTER = 576 TEXT = 'Based on the Mtt V identified as a ?calcaneum by Young (1936: fig. 13d) (MDE, 23 April 2014).';

TEXT TAXON = 107 CHARACTER = 576 TEXT = 'Based on PVSJ 373 (MDE, 13 October 2014).';

TEXT TAXON = 78 CHARACTER = 577 TEXT = 'Based on PVL 4606 (MDE, 26 September 2014).';

TEXT TAXON = 25 CHARACTER = 581 TEXT = 'The pedal digit IV is not completely preserved in the available specimens of Mesosuchus (MDE, 11 September 2012).';

TEXT TAXON = 6 CHARACTER = 582 TEXT = 'Based on Evans (1981: 105) (MDE, 17 February 2014).';

TEXT TAXON = 74 CHARACTER = 588 TEXT = 'Based on SAM-PK-6048 (MDE, 1 April 2014).';

TEXT TAXON = 35 CHARACTER = 32 TEXT = 'I can''t discern the pineal foramen in BSPG 1995 I 5 and Gottman-Quesada and Sander (2009) didn' t describe the position of the foramen in the skull roof. So , I have to score this character as unknown ( MDE , 3 September 2012 ) . ';'

TEXT TAXON = 37 CHARACTER = 32 TEXT = 'The suture between the frontals and parietals is not clear in AM 3585 (MDE, 4 September 2012).';

END;

BEGIN ASSUMPTIONS;

OPTIONS DEFTYPE = unord PolyTcount = MINSTEPS;

TYPESET * UNTITLED = unord: 3 - 6 8 - 9 11 - 16 18 22 - 27 30 - 35 37 - 39 41 43 - 49 51 - 53 55 - 65 67 - 70 72 - 74 77 - 121 123 - 126 128 - 145 147 - 152 154 - 155 158 - 170 172 - 175 178 - 186 188 - 201 203 - 220 222 - 226 228 - 262 264 - 265 267 - 278 280 - 282 284 - 323 325 - 326 328 - 330 332 - 336 338 - 344 346 - 350 353 355 - 360 362 - 364 366 - 369 371 - 376 378 380 - 397 399 - 409 411 - 423 425 - 429 431 - 434 436 - 445 447 449 - 453 455 - 457 459 461 - 462 464 - 471 473 - 477 479 - 481 484 - 488 491 - 503 505 - 509 511 - 515 517 - 528 530 - 536 538 - 545 547 - 551 553 - 555 558 - 566 568 570 572 - 573 575 - 580 583 - 587 589 - 620, ord: 1 - 2 7 - 10\3 17 19 - 21 28 - 29 36 40 42 50 54 66 71 75 - 76 122 127 146 153 - 156\3 157 171 176 - 177 187 202 221 227 263 - 266\3 279 283 324 - 327\3 331 337 345 351 - 352 354 361 365 370 377 379 398 410 424 430 435 446 448 454 458 460 - 463\3 472 478 482 - 483 489 - 490 504 510 516 529 537 546 552 556 - 557 567 569 571 - 574\3 581 - 582 588;

END;

BEGIN MESQUITECHARMODELS;

ProbModelSet * UNTITLED = 'Mk1 (est.)': 1 - 620;

END;

Begin MESQUITE;

MESQUITESCRIPTVERSION 2;

TITLE AUTO;

tell ProjectCoordinator;

timeSaved 1479417449881;

getEmployee #mesquite.minimal.ManageTaxa.ManageTaxa;

tell It;

setID 0 2249447511013316972;

tell It;

setDefaultOrder 0 1 2 3 4 5 6 7 8 9 10 11 12 13 14 15 108 112 16 109 110 111 107 17 18 19 20 21 22 23 24 25 26 27 28 29 30 31 32 33 34 35 36 37 38 39 40 41 42 43 44 45 46 47 48 49 50 51 52 53 54 55 56 57 58 59 60 61 62 63 64 65 66 67 68 69 70 71 72 73 74 75 76 77 78 79 80 81 82 83 84 85 86 87 88 89 90 91 92 93 94 95 96 97 98 99 100 101 102 105 106;

attachments ;

endTell;

endTell;

getEmployee #mesquite.charMatrices.ManageCharacters.ManageCharacters;

tell It;

setID 0 1048097893960857826;

tell It;

setDefaultOrder 0 1 2 3 4 5 6 7 8 9 10 11 12 13 14 15 16 17 18 19 20 21 22 23 24 25 26 27 28 29 30 31 32 33 34 35 36 37 38 39 40 41 42 43 44 45 46 47 48 49 50 51 52 53 54 55 56 57 58 59 60 61 62 63 64 65 66 67 68 69 70 71 72 73 74 75 76 77 78 79 80 81 82 83 84 85 86 87 88 89 90 91 92 93 94 95 96 97 98 99 100 101 102 103 104 105 106 107 108 109 110 111 112 113 114 115 116 117 118 119 120 121 122 123 124 125 126 127 128 129 130 131 132 133 134 135 136 137 138 139 140 141 142 143 144 145 146 147 148 149 150 151 152 153 154 155 156 157 158 159 160 161 162 163 164 165 166 167 168 169 170 171 172 173 174 175 176 177 178 179 180 181 182 183 184 185 186 187 188 189 190 191 192 193 194 195 196 197 198 199 200 201 202 203 204 205 206 207 208 209 210 211 212 213 214 215 216 217 218 219 220 221 222 223 224 225 226 227 228 229 230 231 232 233 234 235 236 237 238 239 240 241 242 243 244 245 246 247 248 249 250 251 252 253 254 255 256 257 258 259 260 261 262 263 264 265 266 267 268 269 270 271 272 273 274 275 276 277 278 279 280 281 282 283 284 285 286 287 288 289 290 291 292 293 294 295 296 297 298 299 300 301 302 303 304 305 306 307 308 309 310 311 312 313 314 315 316 317 318 319 320 321 322 323 324 325 326 327 328 329 330 331 332 333 334 335 336 337 338 339 340 341 342 343 344 345 346 347 348 349 350 351 352 353 354 355 356 357 358 359 360 361 362 363 364 365 366 367 368 369 370 371 372 373 374 375 376 377 378 379 380 381 382 383 384 385 386 387 388 389 390 391 392 393 394 395 396 397 398 399 400 401 402 403 404 405 406 407 408 409 410 411 412 413 414 415 416 417 418 419 420 421 422 423 424 425 426 427 428 429 430 431 432 433 434 435 436 437 438 439 440 441 442 443 444 445 446 447 448 449 450 451 452 453 454 455 456 457 458 459 460 461 462 463 464 465 466 467 468 469 470 471 472 473 474 475 476 477 478 479 480 481 482 483 484 485 486 487 488 489 490 491 492 493 494 495 496 497 498 499 500 501 502 503 504 505 506 507 508 509 510 511 512 513 514 515 516 517 518 519 520 521 522 523 524 525 526 527 528 529 530 531 532 533 534 535 536 537 538 539 540 541 542 543 544 545 546 547 548 549 550 551 552 553 554 555 556 557 558 559 560 561 562 563 564 565 566 567 568 569 570 571 572 573 574 575 576 577 578 579 580 581 582 583 584 585 586 587 588 589 590 591 592 593 594 595 596 597 598 599 605 606 608 609 610 611 612 613 614 615 616 617 623 619 600 601 602 603 604 620;

attachments ;

endTell;

mqVersion 275;

checksumv 0 3 2068313017 null getNumChars 620 numChars 620 getNumTaxa 111 numTaxa 111 short true bits 2305843009213694015 states 63 sumSquaresStatesOnly 128920.0 sumSquares -1.4296226657124903E21 longCompressibleToShort false usingShortMatrix true NumFiles 1 NumMatrices 1;

mqVersion;

endTell;

getWindow;

tell It;

suppress;

setResourcesState false false 100;

setPopoutState 400;

setExplanationSize 0;

setAnnotationSize 0;

setFontIncAnnot 0;

setFontIncExp 0;

setSize 1366 649;

setLocation -8 -8;

setFont SanSerif;

setFontSize 10;

getToolPalette;

tell It;

endTell;

desuppress;

endTell;

getEmployee #mesquite.charMatrices.BasicDataWindowCoord.BasicDataWindowCoord;

tell It;

showDataWindow #1048097893960857826 #mesquite.charMatrices.BasicDataWindowMaker.BasicDataWindowMaker;

tell It;

getWindow;

tell It;

setExplanationSize 30;

setAnnotationSize 20;

setFontIncAnnot 0;

setFontIncExp 0;

setSize 1266 582;

setLocation -8 -8;

setFont SanSerif;

setFontSize 10;

getToolPalette;

tell It;

setTool mesquite.charMatrices.BasicDataWindowMaker.BasicDataWindow.ibeam;

endTell;

setActive;

setTool mesquite.charMatrices.BasicDataWindowMaker.BasicDataWindow.ibeam;

colorCells #mesquite.charMatrices.ColorByState.ColorByState;

tell It;

setStateLimit 9;

toggleUniformMaximum on;

endTell;

colorRowNames #mesquite.charMatrices.TaxonGroupColor.TaxonGroupColor;

colorColumnNames #mesquite.charMatrices.CharGroupColor.CharGroupColor;

colorText #mesquite.charMatrices.NoColor.NoColor;

setBackground White;

toggleShowNames off;

toggleShowTaxonNames on;

toggleTight off;

toggleThinRows off;

toggleShowChanges on;

toggleSeparateLines off;

toggleShowStates on;

toggleAutoWCharNames on;

toggleAutoTaxonNames off;

toggleShowDefaultCharNames off;

toggleConstrainCW on;

toggleBirdsEye off;

toggleShowPaleGrid off;

toggleShowPaleCellColors off;

togglePaleInapplicable on;

toggleShowBoldCellText off;

toggleAllowAutosize on;

toggleColorsPanel off;

toggleDiagonal on;

setDiagonalHeight 80;

toggleLinkedScrolling on;

toggleScrollLinkedTables off;

endTell;

showWindow;

getWindow;

tell It;

forceAutosize;

endTell;

getEmployee #mesquite.charMatrices.ColorCells.ColorCells;

tell It;

setColor Red;

removeColor off;

endTell;

getEmployee #mesquite.categ.StateNamesEditor.StateNamesEditor;

tell It;

makeWindow;

tell It;

setExplanationSize 30;

setAnnotationSize 20;

setFontIncAnnot 0;

setFontIncExp 0;

setSize 1266 582;

setLocation -8 -8;

setFont SanSerif;

setFontSize 10;

getToolPalette;

tell It;

setTool mesquite.categ.StateNamesEditor.StateNamesWindow.ibeam;

endTell;

rowsAreCharacters on;

toggleConstrainChar on;

toggleConstrainCharNum 3;

togglePanel off;

toggleSummaryPanel off;

endTell;

showWindow;

endTell;

getEmployee #mesquite.categ.StateNamesStrip.StateNamesStrip;

tell It;

showStrip off;

endTell;

getEmployee #mesquite.charMatrices.AnnotPanel.AnnotPanel;

tell It;

togglePanel off;

endTell;

getEmployee #mesquite.charMatrices.CharReferenceStrip.CharReferenceStrip;

tell It;

showStrip off;

endTell;

getEmployee #mesquite.charMatrices.QuickKeySelector.QuickKeySelector;

tell It;

autotabOff;

endTell;

getEmployee #mesquite.charMatrices.SelSummaryStrip.SelSummaryStrip;

tell It;

showStrip off;

endTell;

getEmployee #mesquite.categ.SmallStateNamesEditor.SmallStateNamesEditor;

tell It;

panelOpen true;

endTell;

endTell;

endTell;

endTell;

end;

BEGIN MacCladeStart;

Extended;

END;
