# Supplementary material for: A new horned and long-necked herbivorous stem-archosaur from the Middle Triassic of India
Source: Sci Rep. 2017 Aug 21;7:8366. doi: 10.1038/s41598-017-08658-8 (PMC5567049; doi:10.1038/s41598-017-08658-8)
Supplement: Supplementary file 3 — data matrix TNT [file 41598_2017_8658_MOESM3_ESM.doc]

xread

620 111

Petrolacosaurus_kansensis 0000110000010-0000101000000200000100---00[1 2]10000-0--10---0---0--000-0001000300000-0-000--0000000-00000-?03?0100001101-0-000000000-000000-0000--000?0001??3???1101-0-0100000000000-0-0?-10001000000000000110?0000010001000?00000??00-00-10?00?2?0????0?100?--??????0-000-0000101010-0-1000010-00100000?00000000000010000000?0000020--00010110100?010100100100-0010200000??2[0 1]0110010000??0030000?00?0?010010100?20000-0001000000000?1000000001012?00011000000010?02000000000?0000100111000000020-10-20?00001?0000?????00001000??2001??200?00000??000?000000000??0000-------00020000100001203?0002?101??00100000----------0000001000000000??0000

Acerosodontosaurus_piveteaui 0?0?????????0-?0?0???????????????????????????????-???--??????--0???0??10004???????-010--000?000-??000000?0?????0?1?1???000??00????00?0????????0002?????????????1???????????????0-??0?-????????????????????0???0?????????????????????????????????????????????????????00-???2???????????00??10??0?????000??100?00000?000000?0000000--??0?????????00?1???0?-?0?0011?01000000001-001??????1???????0????????????????????0????????????????????101000-?00?110000001??0100????00?100-010000010?001020-00-?????0??1?000??-??00000000?0??1????0?????????0????????????????????????????????????????????????????????????0----------00?00010000??00???0???

Youngina_capensis ?0000-0000010-000011?00000010100?11110-01300000-1--00---0-0-0--00??0001000310000-0-010--??10100-10010000000000001101-0-000100100-1000010101000000200010020001101-0-01000000000002000?-00000000100000100101000?00100011001000011001000-1000002-0000010100?--0000000-000-00?010?[0 1]10-0-?0000110?00000010?00010010[0 1]0[0 1]000?0000?0000000--0??0????0000000100000-00-0?11000000000001-001?00?11[0 1]0100001201000100?0?00020000-?000010001000000000?1101000-00?11100?00110201000000000100-0000011100001020-00-001000011000000-0100??0000?00111?0200?00??????0??1000000????0000-------0002000010000120[2 3]01002?00100001000010-001-200-000?0010000?10000?0?00

Paliguana_whitei ??000-00????0-?000???0?????????????????0?????????-???--??????--???????1?????0?????-0??--???0100-?00??????????00??10?00-??10000?010000010????????10?00???????2-??-????000002-1??22?0???0????????0?????????2?????????????????????????0?????????????????????????????0-??0-?0??00?????????00???0?0????????????????200000????????????????????????????????????????????????????????????????????????????????????????????????????????????????????????????????????????????????????????????????????????????????????????????????????????????????????????????????????????????????????????????????????????????????????????????????????0??0????????????????

Planocephalosaurus_robinsonae 0?000-0000000-0000?01000000201000100---00210?00-?--10---10011--000-0001000200000-0-010--??00001-00-10010?0000001010100-0011000200000001000010001010000------2-10-0-0100?002-10022000?00?000001101012-00002000?0???0??????????0??01010-10??0?0?000????110???????????020-?02200?00110-0000--2010000-011????1301020[0 1]000?0000?0000000--??1?001?000?00?1???0??00?00?2?00000010101?001?00?1111???00??0000001101001020000-101?01??0??0???0?????100???????????????????????????000?00-0100101101000000-0--10???01????0???-???0000000?0?1????????0???????????-1000001??1?00-------0?0????????????????????01111????????????????????000010000?100?0?00?1

Gephyrosaurus_bridensis 0?00110000010-0000?00000000101000100---00110?00-1--10---10001--000-0001000400000-0-010--0000001-00-10000-0000001110000-001100020000000100010100000000[0 1]000--02-10-0-0100?0?2-10021000?000000001101011-0010200010???0???????0000??01010-10000?0?000??10110?01????????020-00020010011[1 2]11000012010010101100??12010200010?0000?00000??????1000?00000000000?0??00?00?3001000010101?001?0011011?0??01[0 1]0?0?01?0110???200?0-100001??0??0???0?????100???????????????????????????0?0?00-0?00000101???000-1--???0000??00?0?????00001000?0?1????????0?????0?000?-100000?0?1?00-------0?0?????00?????????????11111?[0 1]??1?1?????????????00001000??10??0?0?01

Cteniogenys_sp. 0?10100010??0-??00???00?[0 1]??[3 4]?000?000---00[1 2]00??0-0--10--?1[0 1]101--000-00010003?0?????-010--0100000-000100?00?1-0000-102-0-0?10010?0-000001???????0???0??10?3??1??00-0-2-?0?0?2-???130?0000101?000?0??01000???0??10???0??2????0000??010?1000000?0?000?0?0120?01?0??????000-?0?01000?11???00001????0???????????0010000100?101??00000?????002????000?????0??????????110010??????????????????0????00???????????????????????????????????0000000?1111-1?00?????????????????????0?0?00-0?000101?????????????????????00?000-0100000000?????????????????????????????????????????????????????????????????????????????????????????????000010?????0????[0 1]???

Simoedosaurus_lemoinei 0?100-0010200-000030?000011?0?000--0---00301?00-0--?0---10001--000-00000004101?0-0-010--??00000-00-11000201-0000-102-0-002--1000-0000011000010000000110020022-00-0-2-101102-1001?0100?01010000?0103--0011000010110001200101000101101101000000-0000010120?01?000-0??000-00010000110[1 2]01000011000000101010???0010100000?101000000010[0 1]-100000001000000100000-???0010001000000001-?0111010-0?1000?1?1100011101000020000-?01101010??001000100111[0 1]00[1 2]1000?110????????????????000?0000110000100?01000-0--?000000??00000000100001000?0111001?00100000000?0?0?????????????????????????????????????????????????????????????????????00?011000?100?00000?

Aenigmastropheus_parringtoni ?????????????????????????????????????????????????????????????????????????????????????????????????????????????????????????????????????????????????????????????????????????????????????????????????????????????????????????????????????????????????????????????????????????????????????????????????????????????????????00010111000[0 2]?????????????000?1???????????1???100?000?0?????????????????????????????????????????????????????????????111002000???????????????????????????????????????????????????????????????????????????????????????????????????????????????????????????????????????????????????????????????????????????1100???0????????

Protorosaurus_speneri ?0000-0200010-000011??0000020100?10110-0?300?00-1--10---10???--00??0001000310000-0-01?--0000000-0000??00-00000000100-0-??0000000-0000010001?100000000???????2-[0 1]0-0-[0 1 2]?10000??10012?00?0?0??200?????[0 1]????????????010?0110010?????????1???????????????????????????????000-?0?2000010-[1 2]010000020000?00010?????0010[0 1]00000?100101111020--0?0[0 1]0011?1000[0 1]?100110310?2110?100010010010101?0000-000000?100000011011?00011010-?0000000?0010?000000111101201?011000000101202?00010????0[0 1]?0?000???00??1?20-?0-????0011?00?0??-???0??0000?1101???20???000???010??00000001??0000-------00020000001001[0 2]030100[0 2 3]?11100[0 1]0100000----------00000010?000?0?00??0?0

Amotosaurus_rotfeldensis 0-?0100?????????10????[0 1]0?0??????????????0110?00-????0???1110???00??000?0003????????0???????0000?00?0?000-00?0?001?0???????????????????????????????????????????????????????????????????????2000??003--00102??????????????????????0?????????0?0?0????1?000???????????000-00?200?010-0-?0000??0000???0??????100101001?011001?1100?00--1???1112210??0?10?111-1??211[1 2]00?001??0011-[0 1]01000111??20????01?00110000?00010000-?????????1??0?0000?1?0??1-1?[0 1]?0?100???????000??0???0???0100111000101001100-0--10??001???0???????00000000?1??1???20?????????01????0???0????0??0-------00022101??1111203?1002?011??1?110[0 1]00----------0000001???0????00?0?0?

Macrocnemus_bassanii 00000-0200010-0000?10?0000120000?0021000010??00-?--1?---11101--00??00010003?0?0?-?-0?0--???0000-000???????000000110100-01000100000100010?00???????0?0?????????01-0-2-10?0????0?1???0?0??0?0010???001200???????????????0????????????????????????????????????????????000-?0?0?000??????0000????00?00????????001000000011001?1?10000--1???1111210?10?111110-[0 1]??21130?0001000011-1010101111020?00101?0011?000?0??10000-10110000000?0??000???0111-0-000[1 2]100??????110?0000000?01011011101[0 1]101001020-00?10??0011[1 2]?0?0?????0000----?1??1???2???00??0??010??00000001??0000-------00021100001111203?1002?12011?0100000----------00000010?00010100?0??0

Tanystropheus_longobardicus 0-000-0200010-0[0 1]1001?00000120000?0021000010??00-0--00---11101--000-3001000110000-0-010--0000100-0001?000-0001000111000-01110[0 1]01000[0 1]0001000-1001-----00------??11-0-0[0 2]1000?2-10011000?0?0002[0 1]1010005-2-010200??0010101?00??0?00?00??01?10?????????0?1?000?11??10-?0-000-?002000010-0-10000120000000010?01110[0 1]1[0 1][0 1 2]0010[0 1]1100101111000--2?0111132100101110111-11?212?200001000011-10100010-10[1 2 3]100010110011[0 1]000?00010000-10???0?0?0010??00001?0111-0-0?0[0 1]10000111010010100010?01011021120[0 1]101001100-0--10??00111100000-0100000000?110110120???000???000?100000001??1010-------00022110001111203?2000?0111020110000----------00000[0 2]11000010100??0?0

Jesairosaurus_lehmani 0?000-00????0-?000[1 2 3]1?000?01?0????????????100?00-?-?10--?1000?--00??00010002???????-010--??00001-00-10000-00????01101-0-??0100020-1100?100010000000000?????????01-0-000000?2-10?12?00?0?????00??0?03--00102?????0?0??11??????????0??1??1???0?0?0????????????????????000-00?210?010-0-???00020000??001??????[0 4]01010???01?00??10?0?00--[0 1]??[1 2]0000????00?1???00-?????200[0 1]0000??[0 1]001-10?00????1?100???00?010?11???0002??00-10110001?1?10??0?0???0101-??????????????????????????0??0[1 2][0 1]0?10000100?01100-0--?0?0?00???0???????????----?0??????[0 1 2]????????1?110??????????????????????????????????????????????????????????0----------000?00?0000?110???????

Pamelaria_dolichotrachela 0?000-0110??0-0[0 1]10[0 1]???[0 1]??01??0???--20??0?20??00-?-???---111??--00??0??1000[1 2]??????0-010--??00000-001?0?00-0000010?00?0?-0000000001?100010????00??????11??????2-0?-????101002-10?120011110000011?0??1?-?0??????0?0100011?0?0100?10011110000?11?10000010000???0?1?????020-0012110010-0-01000?110???0?01?00???10?0211000?1001011110100-100100?12100000100100-?1??1100[0 1]0111101001-00100011010??0002?1100011001?00000100-?0????????01100010001111010001011000?00??12000011?0000101001100001000000102000100010010100000-0100000000?020010120010000010011102001000000001100000100001100000??01203?10020120??????1010----------0?00?0100000?010000000

Azendohsaurus_madagaskarensis 0?000-0110010-0110112000001100001--200000200?00-0--10---11[0 1]00--100-00010001?000?-0-01?----00000-00110000-00??0101100011??00000001110001000?0?000000011000-01??0000-02111002-10?12001011000201111?02-1001010000001010011020100010111010000?11211????10?0??1?0?11??10000-0022001010-0-10000?1??01?10010?01111010221001?100101111000--1010001[0 1]21001?0100110-11?[1 2]110000101101001-10000000-1010000111100001101000010100-?00001010?01100000001111011001[0 1]??00000010??02011110000?020011000[0 1]100?000111000?0?0100??00000000100001000?0200101?00100000100?0?020010000000011000000000021000001?01?030100101201020101010----------0001101010001010000000

Azendohsaurus_laaroussi 0???????10??0-????????0?????0???1??2????0????0???-?10--?1110?--??0-000?000[1 2]????????????????????????????????????????????????????????????????????????????????????????????????????????????????????????????????????????????????????????????????????????????????????????00???0220010??????000??????????????????1010221001????????????????????????????????????????????????????????????????????????????????????????????????????????????????????????????????????????????????????????????????????????????????????????????????????????????????????????????????????????????????????????????????????????????????????????????????????0110????????????????

Shringasaurus_indicus 0?0?0-0110010-?????1?0???011[0 1]1001--2000?0200?0???-??0--?1????--????[0 2]???000[1 2]?00?0-0-010--???????-??????????0??000100000-00000??????????????????0??????1????????0010-??11?0?????0120?1011?002?1???????????????????????????????[0 1]?1?111?10001?11011???0?0000????????????20-??????1????0-??00??????????????????1??0221000?100101111020--???10??011001001000001?1??1100001111010014?0100010-1??00002?1?00?011010??0101010?00001?10?0110001000211101100?0???0????????????????000?010011010[0 1]10??000[1 2]1?????000?0???10000000100001000?0200????0010000010010?02?010000000??????????????????????01??????????????????1??0----------00001010111?001?000000

Trilophosaurus_buettneri ??000-0200010-0[0 1]010?21001-1200010?02000?150-000-0-010---10101--10??01010001?1000-0-010--???0001-00-?0?-??-001010110000-??01000201?00001010-1001-----11?-?-??2-00-0-??101002-10012001?100???1-1???1----0??????1?0101001?030100010111110000?1101000??1?000?11????????220-102200?0?????110001111000100?0?01111010202000?100100011000--0?11000021001?1100010-01??11[0 1]000-11000001-00100000-101000012000[0 1]011101000020100-100000011?010000110011110110010110000?0001202001110000[2 3]00-0110[0 1]00100001000-1--10000001100000100100001000?010000120010000010010?02000000??00011000000000021000001001203?1002?120??00101010----------0010012100111111000000

Trilophosaurus_jacobsi 0?000-01????0-?001???10???????????????????????0-?-??0--??0???--????0??[0 1]000[1 2]??????0-0?0--???0001-00-???-??-0?00?0?00??0-???100020??000010?0-?00???-??11?-??????0?00-2-10??????0????0????????????????????????????????????????????????????????????????????????????????220-?022???0??????1000?????0??0????????10?0202000?1?01????????????11????21001?1100?10???????????????????????????????0???00????0???????????20?00-???????????10000010011110110010????00???????2????1?0?0?0100100?0010??01000-1--??????0??00000000100000000?0?00001?00100000???????2000000000001100000000002?????0???1????????0?????????1?10----------??10012??1?1?1?1??00?0

Spinosuchus_caseanus ?????????????????????????????????????????????????????????????????????????????????????????????????????????????????????????????????????????????????????????????????????????????????????????????????????????????????????????????????????????????????????????????????????????????????????????????????????????????????????100101111000--0??1???0210?1?1?0???0??1???1[0 1]000-11?0?001-?010?0?0-????3????????????????????????????????????????????????????????????????????????????????????????????????????????????????????????????????????????????????????????????????????????????????????????????????????????????????0----------??????21011?????0?????

Spinosuchus_combined 0?000-01????0-?001???10???????????????????????0-?-??0--??0???--????0??[0 1]000[1 2]??????0-0?0--???0001-00-???-??-0?00?0?00??0-???100000??000010?0-?00???-??11?-??????0?00-2-10??????0????0????????????????????????????????????????????????????????????????????????????????220-?022???0??????1000?????0??0????????10?0202000?100101111000--0?11???021001?1100?10??1???1[0 1]000-11?0?001-?010?0?0-?0??300????0???????????20?00-???????????10000010011110110010????00???????2????1?0?0?0100100?0010??01000-1--??????0??00000000100000000?0?00001?00100000???????2000000000001100000000002?????0???1????????0?????????1?10----------??1001210111?1?10?00?0

Teraterpeton_hrynewichorum ??000-0001010-00010[1 2]20000-141??000010000?5--??0-?--10---1010?--00??0101000210000-0-0?1--??10000-00-000-02-010010110100-??00000201000?01010-?00001-0011-----12-01-????101??2-10011001?0???0?01???????????????0??0101011?0?????0?????010????0??????001?????????1???0?00?????2?0??1????1100??10??0?10?10????01010200001?1?00??????00--???0???0[1 2]00?1??10?010-11??1???????1?00???-10???????????????[0 1]??0??011?1????2???0-?0?????????????????????????????????????????????10???????????????????????????????????????????????????????????????????????????????????????????????????????????????????????????????????????0----------??0?0110????1?????????

Noteosuchus_colletti ?????????????????????????????????????????????????????????????????????????????????????????????????????????????????????????????????????????????????????????????????????????????????????????????????????????????????????????????????????????????????????????????????????????????????????????????????????????????????????1?0???????0[0 1]??????????????????????????????0000??1???001?0?1000111?0200?0?0??????????????????????????????????????????????????0????00001??1?2?0100?0??100-0110[0 1]0010000??2????01??0?00??100000-0100000000?0201101200100000??01???200?000?0000110000000000?[0 1]000001?01202?1002?12010?0?000?0----------00?????0??0????00?00?0

Mesosuchus_browni 0000100110210-0110101000201111001--200010400?00-0-010---10001--00100101010-00000-0-010--0100000-01010000-00000001000010??10101001000101000000000010001000-02001010-00101002-100120000000011000000000000112010110100011112010?01?110110100?0101000??10100?1???110?0-000-00?000?01????002100210110010100???01010-00010?1001?0000000--1?010000110010?100000-1???110000?01??1001-0010001101020[2 3]??00000[0 1]011100?00010000-10100001000??????????1111-0-00??10000???0??0200??0?00020100100001100001010-0000000?00??100000-0100000000?020110120010000010?10?1200?0000?00011000000000021000001?0120201002?120?0?0?00000----------00000010000000000?0000

Howesia_browni 0?00100210?????[0 1]10[0 1]??00????????????????1????????????????1???1??00??1??1111-?0100?0?010????00000?010?0000-00000001000-10001010?00-000?01000?00000000?0???????0?10-0-1??0?0?2-1?0120?0?0?0???000?0[0 1]001000102010010100?1?11??1??0???101???00?0?0?000??1?000?????????0-?[0 1]0-???????????????21??21??1?01??0?????10?0-00010?1?0?????????????0[1 2]000?????????????????????0?00??1??1????0??000?101??0[1 2]0?0011??00?000??0010?????01001?????1?????????01????????????????????????????0?0?0100100000100001020-00010???00???0?000-0??0000000?0?011?1?00100000?0010?1200?00?0?000110?0000000021000001?01?0???00??120???0?0???0----------??0?00?00?????0?0?0000

Eohyosaurus_wolvaardti ??00??02???????110[0 1]???????????????????????????11???????????????0???1??1111-??????????????100000?01110000-0??????????-??00[0 1]??0100-100101000000000011001001002001?-????1?1?02-1??12000000?0?201??0??[0 2 5]???0????????0?0??11??????????????????????????????????????????????00-???1????10-[1 2]0??210021011?1101??????1???-000101?????????????????????????????????????????????????????????????????????????????????????????????????????????????????????????????????????????????????????????????????????????????????????????????????????????????????????????????????????????????????????????????????????????????????????????????????????00????????????????

Rhynchosaurus_articeps 0000100210210-00200010-12-1-2-000--20002?5--?0111-010---10001--00??1101111-10100-0-010--0100100-00111000-00000000000-11??1000100-1001010000100000000010010020110-0-2-100002-1001?00000000?31-0?000----0102??1111??00111????????0?1?1??0???01??000???0000???0?1?????110-?0?10000?????0122002?0?100101000??01010-000101100??0000000--1??????0?10?00?10000?-11???1?000?0100000?-1??00011110?0[0 1]0?0010000011?0?0001000111010000100010?00000011111-0?000110000????1?01?00?010?0?0100?001[0 1]0100?010?0-0??10???00?2?0????????000000000??[0 1]???20???0000???1???2?0?00?0??0?110?0??00000?100?0??001202?1002?110?01??000?0----------000000?00000?0?0000?00

Bentonyx_sidensis 0?000-0210210-00200010-12-1-2-000--20002?5--?0101-010---1000?--00??1?01111-10100-0-010--01?1000-001?1?????0000000000-11??1010100-100101000?10?00????0???????1110-0-2-100002-1001?000???00131-0?000----01?2?????1???0111?[0 2]0????1?110110000?010?1??????100???????????110-?0?100?01?????122????0?1???????????1?1?-00010????????????????????????????????????????????????????????????????????????????????????????????????????????????????????????????????????????????????????????????????????????????????????????????????????????????????????????????????????????????????????????????????????????????????????????????????????00?0????????????????

Eorasaurus_olsoni ?????????????????????????????????????????????????????????????????????????????????????????????????????????????????????????????????????????????????????????????????????????????????????????????????????????????????????????????????????????????????????????????????????????????????????????????????????????????????????1001?11100?[0 2]??????????1?000[0 1]?11???0?011??1?????1????????????????????????????????????????????????????????????????????????????????????????????????????????????????????????????????????????????????????????????????????????????????????????????????????????????????????????????????????????????????????????0??????????????

Prolacertoides_jimusarensis 0??0??0?00?10-???????00????????????????0?????00-?-???--?10000--0???2?010002?00?0?0-010--???0000-??????????00100?????????????????????????????????????????????????????????????????????????0?31-0?0?0????01??000?0???????????????????????????????????????????????????????????????????????????????????????????[0 4]???2000??????????????????????????????????????????????????????????????????????????????????????????????????????????????????????????????????????????????????????????????????????????????????????????????????????????????????????????????????????????????????????????????????????????????????????????????????????0??0????????????????

Prolacerta_broomi 00000-0[1 2]00010-0[0 1]10111001201210?00?0210001100000-?-010---100?0--000-000100031000?-0-010--0000100-010100[0 1]0-0000000110100-0010000000100001010000000000001000-011101[0 1]0-[0 1 2]0101002-1001[1 2]00001101000001100000011010110?01000111000110000010110[0 1]000002100010101000110011000-000-0000100010-0-000000210001110100011110100010001100111010020--1001101121000[0 1][0 1]1011103011[1 2]111000001001001200100011010100?0??0000010000000011000-101100010?000000100010111-0-0??11000??0??1?010000?000010100[1 2 3]01001100001020-00-1000001??100000-0100000000001001012001000001??10?120010000?0001100000?00002100000110120301002?120??20100000----------00000010000010?0000000

K_australiensis_holotype ???00??1?????????01????????????????????????????????????????????????????????????????????????????????????????????0???100-??[0 1]00?00?0?????101000?0000[0 1]00?1000-01[0 1]?0111000101002-1?????0???????????????????????????????0???10????????0?????????0?2?000????100????????????????????????????????0?2??0??1??????????????????????????????????????????????????????????????????????????????????????????????????????????????????????????????????????????????????????????????????????????????????????????????????????????????????????????????????????????????????????????????????????????????????????????????????????????????????????????0????????????????

K_australiensis_combined ???00-?1????0-???01???????????????????????????0-?-???--?1000?--0???[0 3]??[0 1]00????????0-???--???????-??????????00???0???100-??[0 1]00?00?0?????101000?0000[0 1]00?1000-01[0 1]?0111000101002-1?????0???????????????????????????????0???10????????0?????????0?2?000????100????????????????????????????????0?2??0??1??????????????????????????????????????????????????????????????????????????????????????????????????????????????????????????????????????????????????????????????????????????????????????????????????????????????????????????????????????????????????????????????????????????????????????????????????????????????????????????0????????????????

Boreopricea_funerea ??000-0?????0-?0?0[0 1]??00????????????2???011???00-?-??0---1000?--00??0??100?3?0????0-010--??-??00-0?-0------0000000100?0-0?000?000??000010????00???????1000-?1???????1010?0?2-10?12000?10?1?[0 1 2]0???1??????1????????????????????????????????????????00????1?????????????000-00?200??????????00????0????01??????[0 2 4]01000?0?0?1001?1000020--???0???0210010010??00[1 2]1???11?00000100100?[2 3 4]??1????????????0??100001?0000000???????0??00???100?0000000?0111-0-0??1100???????20?0?00?0??????????????????????????????????12?0????????????????010????2?0??0???1?01??02???000???00110000000000?1??000??01?0?0?001012010?0?0000?????????????0??010000??0?0?0??00

Archosaurus_rossicus_holotype ????????00?1???????????????220010?1201??1101??????????????????????????????????????????????????????????????????????????????????????????????????????????????????????????????????????????????????????????????????????????????????????????????????????????????????????????????????????????????????????????????1?????????????????????????????????????????????????????????????????????????????????????????????????????????????????????????????????????????????????????????????????????????????????????????????????????????????????????????????????????????????????????????????????????????????????????????????????????????????????????????????????

'Proterosuchus_ferugsi' ??0?????????100???????[0 1]?????????????????????0??????????-1100?020???0?0?0??[2 3]?0????????????????0?????[1 2]0??0????????????????????????????????????????????????????????????????????????????????1?[1 2]0001100000?100101101????????????????????????????????????????????????????????????????????????0??????????????????10100????01???????????????????????????????????????????????????????????????????????????????????????????????????????????????????????????????????????????????????????????????????????????????????????????????????????????????????????????????????????????????????????????????????????????????????????????????????????????????????????

Proterosuchus_fergusi [0 1]?000-01000110021021110121122001011201001101000-010[0 1]00--110000200100000000320000-0-0101100001000000201100[0 1]000000000000-??100010001000010101000000100010[0 1][0 1]-02110[0 1][0 1]11[1 2]010100011001200001001??0001100000010010110100001111000101?00010110101?01210000010101?111?11?01?0010001110?01110-00000111010110010101111010011000110011100[0 1]020--100[0 1]0010110[0 1]0[1 2]0100000201?210?01001110[1 2]001[0 2]001??????1?????0?0?????????????????????01101100?????????????????01000?1000100???20200?0??????????????????????????????????????100010-010000000000201101?001????????????2001000000001100?????000?1000001?01?03010020?2??0?0?00000??--------00000010000????0???0?0

Proterosuchus_goweri [0 1]?000-010001100210[2 3]1[0 1]00121122?010?1??1?01101000-010?00--11?0002001021000003?00???0-01011000??0??00120?10??0000000?0000-00100?1000??00010?01???000?0001????????000111010100011??130000100112000?1000[0 1]?01???01?0101001111030101?0?010110101?01210000010101???1?110?10????????????????????????????????????11?1?????1??0????????????????????????????????????????????????????????????????????????????????????????????????????????????????????????????????????????????????????????????????????????????????????????????????????????????????0010????100?0?0?????????????????????????????????????????????????????????????????????00?0??????????????0?

Proterosuchus_alexanderi ?0001001?0??1002102??[0 1]01????2??????????0??????0-?1?????-11?00?20???0?0?0003[1 2]0????0?0?0?????01000???20110-0000000000000-??10001000100?01010100000010001000-0211000112-101000110?1300001001??00????000001001?11?101001111000101?0?010110101?0?2?0??????101???1?11??10001000011?001110-00000111000?10??010??1[0 4]010011000110011?000020--1?000010110?0101000001011210??1?011?010014?0?000111101001?10?000010000?00010000-1011011000???0?0?000??????010?0????????????????????000?0100?1??00100?01020-00-?000?00??10?010-01000000000????????????0000???1??120010000000011000001000011000001?01?0?0?00??12000?0?0???0----------?00?0010????0???0????0

'Chasmatosaurus'_yuani 1?000-010001100210?11001?11[2 3]2001011201001101?00-011000--110000200100100000320000-0-01011??00100000120110-1000000000000-??1000100010000101010??00020001000-02???000-2-1010?[0 1]110?130?001??1?2000?1?0[0 2]?0??001?11?1?????111?30??1?0??11110000?0?210??????001???????????001?001110001110-?000011102?110010????1[1 4]010011000?100??1000120--???[1 2]?????????0?1???0?[0 1]???[1 2]?100[0 1]0??1?0[1 2]001310100010-1030010?[0 1]00000100???00010000-1010011000001000001011011-[0 1]10001100????????0?0?10??000?0100110000100?01020-00-?0?0?0011100010-0100000000?0201101[1 2]0010000010010?120???0?0??00110011000000?[0 1]000001?01?????????120??????00000---------000?0010000001?00??0?0

'Chasmatosaurus_ultimus' 2??0????????1??????????????????????????????????????003???????010?210?1?0001?????????????????????????????????????????????????????????????????????????????????????????????????????????????11???1110?????0??????11????????????????????????????????????????????????????00???001010111?0-?000?????????????????140100[1 2]1000????????????????????????????????????????????????????????????????????????????????????????????????????????????????????????????????????????????????????????????????????????????????????????????????????????????????????????????????????????????????????????????????????????????????????????????????????0?00????????????????

Ankistrodon_indicus [0 1]?????????????????????????????????????????????????????????????????????????????????????????????????????????????????????????????????????????????????????????????????????????????????????????????????????????????????????????????????????????????????????????????????????????????????????????????????????????1???0110?0???????????????????????????????????????????????????????????????????????????????????????????????????????????????????????????????????????????????????????????????????????????????????????????????????????????????????????????????????????????????????????????????????????????????????????????????????????0????????????????

Tasmaniosaurus_triassicus 0?0?????????100???????????1?10?????20????10??????[1 2]?????-110??0100??0???000[2 3]???????????1????????????????????????0?1?0???00?????????????????????????????????????0????2-?0???01??????????????????????????1????????????????????????????????????????????????????????????0????0?0??001100-?000?????????????????110?00[1 2]10?0?100??1010????????????????????????????????1??1??????10???????????????????1[0 1]?????????????????????01100010???????????????????????????????????????????????????????????????????????????????????????????????????????[0 1 2]??????????????????????????????????????????????????[2 3]0???????020????????0??????????????00010?1????????????

Exilisuchus_tubercularis ????????????????????????????????????????????????????????????????????????????????????????????????????????????????????????????????????????????????????????????????????????????????????????????????????????????????????????????????????????????????????????????????????????????????????????????????????????????????????????????????????????????????????????????????????????????????????????????????????????????????????????????????????????????????????????????????????????0?0110??1?001???????????????????????????????????????????????????????????????????????????????????????????????????????????????????????????????????????????????????????

Blomosuchus_georgii ????????????????????????????????????????????????????????????????????????????????????????????????????????????????????????????????????????????????????????????????????????????????????????????????????????????????????????????????0??????????121000?0?0101?11?????????????????????????????????????????????????????????????????????????????????????????????????????????????????????????????????????????????????????????????????????????????????????????????????????????????????????????????????????????????????????????????????????????????????????????????????????????????????????????????????????????????????????????????????????????????????

Vonhuenia_fredericki ?????????????????????????????????????????????????????????????????????????????????????????????????????????????????????????????????????????????????????????????????????????????????????????????????????????????????????????????????????????????????????????????????????????????????????????????????????????????????????1001110110101-???????????0?1?1?????-?????????????????0?????????????????????????????????????????????????????????????????????????????????????????????????????????????????????????????????????????????????????????????????????????????????????????????????????????????????????????????????????????????????1???????????????

C_rossicus_combined ?????????????????????????????????????????????????????????????????????????????????????????????????????????????????????????????????????????????????????????????????????????????????????????????????????????????????????????????????????????????????????????????????????????????????????????????????????????????????????100111111020--???1???0111[0 1]02010?000?0?????001???1001?0?????????????????????????????????????????????????????????????????????????????????????????????????????????????????????????????????????????????????????????????????????????????????????????????????????????????????????????????????????????????????1011????????????

Chasmatosuchus_magnus ?????????????????????????????????????????????????????????????????????????????????????????????????????????????????????????????????????????????????????????????????????????????????????????????????????????????????????????????????????????????????????????????????????????????????????????????????????????????????????1?01???1?????????1????111?02010?000-???????????????????????????????????????????????????????????????????????????????????????????????????????????????????????????????????????????????????????????????????????????????????????????????????????????????????????????????????????????????????????????????????1???????????????

Gamosaurus_lozovskii ?????????????????????????????????????????????????????????????????????????????????????????????????????????????????????????????????????????????????????????????????????????????????????????????????????????????????????????????????????????????????????????????????????????????????????????????????????????????????????1?01?11111???????1???01110020?0????????????????????????????????????????????????????????????????????????????????????????????????????????????????????????????????????????????????????????????????????????????????????????????????????????????????????????????????????????????????????????????????????????1???????????????

C_magnus_combined ?????????????????????????????????????????????????????????????????????????????????????????????????????????????????????????????????????????????????????????????????????????????????????????????????????????????????????????????????????????????????????????????????????????????????????????????????????????????????????1?01?11111???????1???0111002010?000-???????????????????????????????????????????????????????????????????????????????????????????????????????????????????????????????????????????????????????????????????????????????????????????????????????????????????????????????????????????????????????????????????1???????????????

Chasmatosuchus_vjushkovi [0 1]????????00????????????????220010?0201??110???????????????????????????????????????????????????????????????????????????????????????????????????????????????????????????????????????????????????????????????????????????????????????????????????????????????????????????????????????????????????????????????1????[1 2]1??????????????????????????????????????????????????????????????????????????????????????????????????????????????????????????????????????????????????????????????????????????????????????????????????????????????????????????????????????????????????????????????????????????????????????????????????????????0????????????????

SAM_P41754_Long_Reef ?????????????????????????????????????????????????????????????????????????????????????????????????????????????????????????????????????????????????????????????????????????????????????????????????????????????????????????????????????????????????????????????????????????????????????????????????????????????????????1?0???????1[2 3]10?????????????????????????????210??1002001-???????????????????????????????????????????????????????????????????????????????????????????????????????????????????????????????????????????????????????????????????????????????????????????????????????????????????????????????????????????????11000???????????

Koilamasuchus_gonzalezdiazi ????????????????????????????????????????????????????????????????????????????????????????????????????????????????????????????????????????????????????????????????????????????????????????????????????????????????????????????????????????????????????????????????????????????????????????????????????????????????????????????????[0 1]?????????????????????????1??????20???????0???????????????????????????????????????????????????1???00????0??1-?????????????????????????0???021?[0 1]1??00???????????????????????????????????????????????????????????????????????????????????????????????????????????????????????[2 3]0-10?11010??????????????????0???

Kalisuchus_rewanensis_holotype 2?00????????10??????????????2??????[1 2]?????????0???[0 1]?000-?1[1 2]???0???10000?000[2 3 4]???????-???????????????????????????????????????????????????????????????????????????????????????????????????????????????????????????????????????????????????????????????????????????????????????????????????????????????????????1???0210?0?????????????????????????????????????????????????????????????????????????????????????????????????????????????????????????????????????????????????????????????????????????????????????????????????????????????????????????????????????????????????????????????????????????????????????????????????????0?0????????????????

Fugusuchus_hejiapanensis ??000-00?0??100210[2 3]1?10????????????????0?????00-?[1 2]??00--110000200??0?010002?0?0?-000?011??00000010010011??00000?200?-0-??100010001000010?01??00001000???????????-0-2-1010?011?????0??????????01???????????????100011111100-01100011110001?01111001010101??1??110??????????????????????????????????????????10100?10?0?????????????????0???????????????????????????????????????????????????????????????????????????????????????????????????????0?00??100????????????00????????????????????????????????????????????????????????????????????????????????????????????????????????????????????????????????????????????????????0??0????????????????

Sarmatosuchus_otschevi 2?000-0?0001?????0[2 3]???????1220010?0201??110???????????????????????????0???[1 2]?????????????????00??00?[1 2]0??1???????0??0????00?????0???????10?01?00000[1 2]0001???????????????1?1???????12000110????0001??00?000002?????0?0111?1000-01?00111110000?1111000?0?0000??1??110???0[0 1]???0211001??????000??????????????????10???21000?1001110111101-[1 2]??1001011000[0 1]0100000-01????0001??110?00?-??????????????0????100010000000010000-??????????????????????????01000?????????????????????????????????????????????????????????????????????????????????????????????????????????????????????????????????????????????????????????????????????????010000??????0????

Guchengosuchus_shiguaiensis ??001000?00?101???[2 3]??1[0 1]????????????2??20?????0??010010-011-??020?10001[0 1]0001?0000000?1??????????????????????????0??00-0-00[0 1]?0????-?????????????????????????????00-0-2-10110[0 1]?1??????????????1-0???0----00?2?????0?1?11?10??-?????????????????????????????0??1?1100???????????????????????0?1??20?????0?1???1010021000?10011?111?100-???1????[1 2]10001010?010-?11[1 2]1????????10200?-?00???????????????1?00?01?00?????????????????????1?????????011??0?0???101??????????????????????????????????????????????????????????????????????????????????????????????????????????????????????????????????????????????????????????????????00?01010???0????????

Cuyosuchus_huenei ???0?????????????0??????????????????????????????????????????????????????????????????????????????10?20??0??????????????????????????????????????????????????????????????????????????????????????????????????????1??????????????????????????????????????????????????????????????????????????????????????????????????????1001111110101-????????1?0001?10??00-?1???10010011?01001-??00?01111?10[0 1]0??[0 1]1?0?00?0????0010000-???????????10000000?10111-0100???0?????????????????0?0[0 1]0110110100100000021?0001?00?0?[1 2 3]110?000001?0???????0[0 1 2]11100[0 1 2]001000001001111?????????????????????????????????????????????????????????10?????????[1 2]????10?????00?00000?

GHG_7433MI 2?000-01????1????????????????????????????????????????????????130?100?1?0001[1 2]?????0?????????????????????????????0[0 1]?0000-??100?10101?0??????????????????????????00111??10?1?????????????????????????????000??????????????????????????????????????????????????????????????0??[0 1]00?1??????000??????????????????4???02100011?01???????????????????????1?1?????????????010??1??1?0??10???????????????0??????????????????????????????01?00000????121-010????01?1?????00??????????????????????00?0?021?000?000?00????????????????????????????001????????????01???????????????????????????????01?03?1010?????????????0?---------0??0?0?0?????0???????0

Garjainia_prima 2?000001000110021021210120111010000200201100110-011011--1200?1300100111000120000100010011-020101100100100000010?001000-1-100010101000110001000000100010020022-?0111??10110[1 2]?100120000010???[0 1]00???0022000020111100011111130-010001101100001111100000?0101021111100110010002100011101?00000111030000010?1??1[1 4]0100210?0?1001111111101-1??200101101010110000-?1??100010111001001-?0000010-0??00???[0 1]1100001100100010000-?0???0110??11000001011111-01001??01????????????????000?0100110101100000021200010?0000??100010-0100000000?0202100?0010000010010?101?????????0110000010000?????????01?????????????????????0?---------??000010000?000???0000

Garjainia_madiba_holotype ??00????????????10?????????????????????????????????????????????????????????????????0????????????100100?00????????????0-??10001?10?0001???????????????1??[2 3]??????????????????????????????????????????????????????0?0??1?11??-?????????????????????????????????????????????????????????????????????????????????????????????????????????????????????????????????????????????????????????????????????????????????????????????????????????????????????????????????????????????????????????????????????????????????????????????????????????????????????????????????????????????????????????????????????????????????????????????????????????????????

Garjainia_madiba_combined 2?00??[0 1]100011???10???????01210100?020[0 1]??1100????????12??1[1 2]???????100?1?000???0?????0??????0?????1001001000???10??????????10001?10?0001???????????[0 1]???100[2 3]002????1????1?111??1????0?0?11????????????????????????0?0??1?1130-?1100010?1000011111000?0?0101????111?0??0????021000[0 1]??????0000?11?[2 3]??1?01?11???[1 4]0?0021000?1001110100101-???[1 2]???0110????11????-?1???00?10??1??1001-?0???010-?????1???1?00001100110?00000-???????????11000001011111-01001????????????????????0?0?0000110?01100?001[1 2]1????1000000??100010-010000000000201100?00100000?????????????????????????????????????????????????????????????????????????????0?01000???00?0?00??

Erythrosuchus_africanus 2?000-00000111021021110010111000100200201100?10-0100120-12000020011311200011000000101001??0201011001001[0 1]010001000000-0-1-1000101-1000010000000000210010020122-00-112-1011111100120001000???1-0?000----00020?10100011111041-0211011111000011?1100001?000002011111011001100?00001110100000011103001001011??14010021000?1101111110101-10?2???01101010100000-0111100[0 2]10011102001-00000010-00?0?00[0 1]01100001100110010000-??????????011000001021111-010111101????????040?????000?0110110[0 1]0110000102110001000000??1000100010000100000302100?0010000010011?0011-0000000011000100000022110001?01?0?03000002110???000?00---------??0000100000?01?000000

Shansisuchus_shansisuchus 2?000-00000110121031210??01110001002[0 1]0201100?10-0110120112-00110011011000012000010101000??0[1 2]000?10010010010000001000-0-1-1000101-?000110?00?000002100100[2 3]012??00-102-101001110012?00?10????1-0???0----01?2?????001111?1041-?2110?11110?00?11?1?0??1?0?00?2011111?11001?0020000111010?0000?110300?0010?????4010[0 1]21000?1101?11?1?101-????0010?10?00?10??0?-?1?2?0001011100?001-000?00?0-0??????0?1100001100?10010000-???????????1100000?0101[1 2]1-0?011??01????????030??00?0?0?0110210010100?0102120?0?0?0000??000??000?00002000??2111?0?00100000101???10101000?0000110011000000?????????01?????????021??????0000?---------??00?01000???010??0??0

Shansisuchus_kuyeheensis ???????????????????????120?110001?0??????10???????10???????????????[0 1 3]?1?00??????????????????????????????????????????????????????????????????????????????????????????????????????????????????????????????????????????????????????????????????????????????????????????????????00?1??????000??????????????????[0 2 4]????????0?1?0??11??????????[1 2]???0110?????0??????????0???00?1?0???1???0???????????????1?0000110???0010000-???????????1100000?0101?1-??????????????????????????????????????????????????????????????????????????????????????????????????????????????????????????????????????????????????????????????????????????????010??????????????

Chalishevia_cothurnata 2?000-???00?101???????[0 1]1[1 2]??????????2??20?????1??0?1012?112-??12??10211?0001??00010??1????????????????????????????????????????????????????????????????1?????2??????????????????01?0?0010??????????????????????????????????????????????????????????????????????????????????????????????????????00?0?????????4???021000?110??110?0????????????1?0?00??0??????????0?010????0????????????????????????????????????????????????????????????????????????????????????????????????????????????????????????????????????????????????????????????????????????????????????????????????????????????????????????????????????????????????00?01???????????????

Youngosuchus_sinensis ??000-010001100210[1 2 3]111000001100000011000?200?00-01-012--111000101??001100?120011-0001002??0101011001001000000000101000-??100010110001010000010000100010020022-?010-2-101102-10012000001???????????????????0???10?1??111????????????1???????????????????????????????0010?0200??11????000001110200000??????1[0 2 4]010021000?10011??11?0[0 2]??1???00?0110?0101??000-1112?????????????0????????????????????110[0 1]001101??00100110???????????100000001101[1 2]1-0?0?12100??????????????????????????????????????????????????????????????????????????????????????????????????????????????????????????????????????????????????????????????????0?00???????0???0????

'Dongusia_colorata' ?????????????????????????????????????????????????????????????????????????????????????????????????????????????????????????????????????????????????????????????????????????????????????????????????????????????????????????????????????????????????????????????????????????????????????????????????????????????????????1?0??11110???????????????????????????????0??2000?1010??????????????????????????????????????????????????????????????????????????????????????????????????????????????????????????????????????????????????????????????????????????????????????????????????????????????????????????????????????????????????11??????????????

Uralosaurus_holotype ???????????????????????????????????????????????????????????????????????????????????????????????????????????????????????????????????????????????????????????????????????????????????????????1-0???0----0002??????????????????????????????????????????????????????????????????????????????????????????????????????????????????????????????????????????????????????????????????????????????????????????????????????????????????????????????????????????????????????????????????????????????????????????????????????????????????????????????????????????????????????????????????????????????????????????????????????????????????????????????????

Uralosaurus_combined 2?????????????????????????????????????????????????????????????????????????1????????????????????????????????????????????????????????????????????????????????????????????????????????????????1-0???0----0002?????????????????????????????????????????????????????????0????0210001??????000??????????????????4????21??????????????????????????????????????????????????????????????????????????????????????????????????????????????????????????????????????????????????????????????????????????????????????????????????????????????????????????????????????????????????????????????????????????????????????????????????????????0????????????????

Vancleavea_campi 2000100-00110-0100101?110011000001020000?1???00-0--00---11001--0-1?010000?100000-0------??00000-01010000?0000000-?02-0-??2--0100--000010-01110000000-10020022-00-0-??00?002-1011001000?????????????????????????0?1??111???100??0010110000??????????????????1??1??1?00???03200?010-[1 2]10000001100???001?????14001-21000?1?0????00?00--???1????1???10?10???0-1????1?3100?100[0 1]00?-??0?00????????00?21100001100010?????????????????000?00000110121-1?0000100???????2?101????000?00-001010010??0????????10?0?00210000000010000----00111100010100000100???101010001000011000000000012110??1?0?30[3 4]0????0001?????????30-101?001?120?00100????00??00000

Asperoris_mnyama 2?001000?0011?0?1????1[0 1]0001100000?0200201200?0????-01[0 1]?-110??0??011[0 3]00?000??00?000?01?????????????0???????00?010[0 1]002-0-00??0????-?????????????????????????????00-0-[1 2]?10?01[0 1]-??????????????????????????????????????????????????????????????????????????????????????????????????????????????????????????????4?????1??0?????????????????????????????????????????????????????????????????????????????????????????????????????????????????????????????????????????????????????????????????????????????????????????????????????????????????????????????????????????????????????????????????????????????????????????????????????0?0????????????????

Euparkeria_capensis 2000[0 1]0010000100110112100[0 2]0110100010200001200100-000[0 1]010-110000200100001000110000000010111-0000000001001000000000110100-??100010000000010000010010000010020022-0100-2-10100011001200000001020011110020001020100101010011040-00000110110000?110100010101000101?11001?0010001100001102?0000002102011101010??1401002100011001011110101-10?10000100?010100000-010211[0 1]010001??1001-00000010-10??[0 1]00?01100001100?00010000-10????000001[0 1]00000?110121-000001100????????0[2 3]01?0??00??0110110100100001011?00020000001[1 2]0010000010000200000[1 2]11000200100000001?0?0211-0000000011111001000012110001?012030[1 2]00[0 1]?010?0?0000[0 1]020-101010110000001000001000000000

Dorosuchus_neoetus ???????????????????????????????????????????????????????????????????????????????????????????????????????????????????????????????????????????????????????????????????????????????????????????????????????????????0111?1?1040-01?0?111?10000?110?000???00000?1?11100??????????????????????????????????????1?????????????1???????????????????????????????????????????????????????????00???1???????????????????????????????????????????????????????????????????????????????0?0101101101001?????????????????????00100000100002000?0211101200100000????????????????????????????????????????????????????????????????????????????????????????1???00??

Proterochampsa_barrionuevoi ?21[0 1]202000111001?0311010011210010?021000?100100-?2-0?12-1100100001?0?020001[1 2]000000001000?-0000000001000000000000[0 1]100-0-??2--0100-0000010001000000100010020022-00-0-2-101002-10?1?0?00000102000?01042-10102?101101011111040-0?01?110111001?0?20001??100000????????1?001?00?0001?10-110?000?0-031000000?0???40100[1 2]10?0?1000?0000?00--[0 1]?021000110??0?10?000-11?[1 2]??1000?1100000?-10000??0-1????0??00?0?001100??00100?0-??????????01000000?1101[1 2]1-0-???11??????????????????0??????????????00??????????????????1001?????1?0002?00?01121?02100000011??10??2?1-00?0??0???????????????????????1[2 3]?????????????????0000----------000??01???0????0???000

Proterochampsa_nodosa 2?1120200011100110312010?11?10?1??021000?100?00-?????0--110010000??0?0200012000100-01000??000000000?0?00?000000??100-0-?????0100-?000010001?00?00?0?0100[2 3]0022-?0-????10100??1001?00001001?2??0???0????0????????0???1111????????????????????????????????????????????001000?000?????1??0?00?0-031??0000?0???40100?00?0????????????????????????????????????????????????????????????????????????????????????????????????????????????????????????????????????????????????????????????????????????????????????????????????????????????????????????????????????????????????????????????????????????????????????????????????????0??0????????????????

Tropidosuchus_romeri ?000202100111[0 1]0010[1 2]1?0100???00010102?000?100?00-11-0000-110010100??0?0100?1?0001?0001010??0000011012000000000000010000-??2--01000010101010[0 1]000000100110121022-0?00-2-10100??1001[2 3]0000000???00011[0 1]002210102?????0?0??111040?0??1?110111000?0?2?000??1?000???????????0011?0?201?0??????0?01?2??3????110?0???[0 2 4]0100[1 2]1000?1001?0000000--100200?0110?00?100010-11???110100?1??0011-10?00010-10[1 2]1000??11000011??0?00000?0-?0?????????10??000?1?0111-0?0??11??????????????????000?0110????00100001011300010?0?001?0010000??00002000?01111?02100000011011101211-0001000011101000000012110001?01201011100000???2--00010-101-0??-0?0?00?0??00?0?000?000

Cerritosaurus_binsfeldi ??0020200011110110[1 2 3]11010??1110?1?10210?0?1???00-12?0011-110?10100??000100?1?0001?0001002??000000100100000000000001??-0-??2--0100-010101010??110001001100310?2-0?-0-2-1010???10013000?00????????????????????????0???1111??????????????????????????????????????????????11???2????????????00?11?3???001??????[0 2 4]010[0 1]01000???????????????1???????????000?????0?????????????1??0?0???0????????????????????????????????????????????????????????????????????????????????????????????????????????????????????????????????????????????????????????????????????????????????????????????????????????????????????????????10-???-1??-??0??0?0??????????????

Gualosuchus_reigi 2?0021210011110210[2 3]1002000121001010210001100?00-11-000--110010300100101000110001-0-01000??01000010[0 1]1000000000000010000-002--01000000111010[0 1]?11000100110021022-0010-2-101002-10012000000010200011?002210102011110?01?1110?0-?0???110111000?0?2?0????1?000???????????0012000201?010-1100001?1003000011000??14010001000?1001?0000000--10020000110?000100000-11?2?0[0 1]0100?1??0001-100??????????????0100[0 1]00110001[0 1]010010-???????????10?0100?1?0121-????????????????????????????????????????00?????????????0?001200100000100002001?0[1 2]1110121000000110110?1?????????????????????0????????????1???????????????????0?10-011-100-0?0000?0??0??-???0?00?

Chanaresuchus_bonapartei 21[0 1]0212[0 1]0011100210310020001[2 3]1001010210001100100-1[1 2]-000--110010100100101000210001-0-01002?-0100001001000000000000010200-??2--010000001[0 1]10100011000100110121022-0010-2-101002-10012000000010200001[0 1]002210002?101101011111020-00?1011011100000121000??1?000???1?????1?00110002010010-1100001?110300[0 1]011000??14010011000?100001100000--100200[0 1]0110?00?100000-11?2?[0 1]0010011000001-10000110-102100?0?1101001100010010010-??????????010001000110121-?????1???????????????????0001011010010010000101130001000000[1 2]300100000100002001001111?021000000110110?1211-00000000111010000000?2111?0110120201110?000??22--00010-011-000-00000010000??010000000

Pseudochampsa_ischigualastensis ?11021210011110?10[1 2 3]1?020001210010?02100??10??00-?2-000--110??0100??0101000[2 3]20001?0-0???0??0100001001000000000000010000-??2--010000?01110101000000100110121022-?010-2-101?0??1001?00000001??000???002210102????1?????1110???0??1?110111000?0?1?000????000?????1?????00???0??0????????0000??0-???????10?0??14010001000??00??0000??[0 1]??1??[1 2]?????10?0?0?0?????11?2?11010??10000?1-100?0????1011?0000000?00?100??00000?0-1?????????????000001????????????????????????????????????1???0??????????011?000??????????0????????0002000????????21??00?011?110?1????0000??0?11[0 1]????0????????????101201?2110?000??????00010-01?-100-000??0????0?1??0?0????

Rhadinosuchus_gracilis ??0021??00111?0???????2???1?1??1?10????0?[1 2]0??0??11??02?-110??0100??0?0?00???0001?00?1??2???[0 1]?????????????????????????????????????????????????????????10?[2 3]1?[1 2]????????????????????????????????????????????????????????1?1?40???0???????????????????????????????1?????0????00211?0??????000?????????????????14??0[0 1]21000?1??????????????????????????????????????[1 2]1????????????????????????????????0?????????????????????????????????????????????????????????????????????????????????????????????????????????????????????????????????????????????????????????????????????????????????????01?????????????????????10-01??000-??0??01???????????????

Archeopelta_arborensis ???????????????????????????????????????????????????????????????????????????????????????????????????????????????????????????????????????????????????????????????????????????????????????????????????????????????0111?0??041-00?1?110?10100?110?000???0000?????110?????????????????????????????????????????????????????1?0???????00--???????????????????????????00000?1????001-1??1?????0?????????????????????????????????????????1010101???????????????????????????????0??111???0?00??00?????????????0?00??00100001100002000?02011?0?101????????????????????????????????????????????????????????????????????[2 3]110110210???????????0????????0??

Tarjadia_ruthae ????20?1???????????????????????????????????????????????????????????????????????????????????????????????????????0??0000-0?100??????????????????????????????????001????101????1??????????????????????????????????010????????????????????????????????????????????????????????????????????????10?3????????0??????????????1?0??10000?????????????????????????????????010??20000???????????????????????????????????????????????????????????????????????????????????????????????????????????????????????????????????????????0020000???????????????????????????????????????????????????????????????????????????????[2 3]1101??2101??????1100????????????

Jaxtasuchus_salomoni [0 1]??020??????1????0????[0 1]??????????????????????0???100-1??1100?000010000?0002????????????????????????????????????????????????????????0?????????????????????????????????????????????????????????0???0????0????????0?11???????-?01??????110??????????????????????????????1???????????????????1???????0?1??????40?0001100?1?01??????0[0 2]??1??[0 1]???1[1 2]10000010?110-???[1 2]?1?[2 3]10?????????-11?????????????0?????????????????????????????????10001010110111-0-??0?000???????2?300??????????????????????????????????????2300100?00??0??2000?0?101002?0?00?011?00001????????????????????????????0????0120???00??00100?1?0?0?311201?01011000?0?0??0??0?????0??

Doswellia_kaltenbachi 2?1020[0 1]0????????-13???????????????????????????0-???????????????0??????0000[3 4]????????0??????11?0??00--0--0--?????0???0-0-??2---100-0-0-0100-1110000-0001----022-00-0-??001002-1??120000000???000???0002101120101101111111020-0011?110011000?010?001??1?000???????????000-?00200001?????0000120001000010001114???0?0????1001?00000101-1???10?01100000110110-10?[1 2]11[1 2]010012000000-11011110-??3000?1[0 1]?????????????????????00000012??????????????????????????????????????????000?111010000010000101130001000000???01???????0???????011?1??????????????????????????????????????????????????????????????????????????31120?1[0 2]101?0???010000?1?0?0?00??

Parasuchus_angustifrons ??10201001111000001??000001?0??-00--1-001[0 1 2]0?100-1?-0?20-1101002001020020002?0000-0001002??10000110020000110000101???-0-????00100-0101010100011000100110020022-0?-0-2-101002-1011210001001131-12011----01020111101010111040-???1?110110000?0?0?1[1 2]-??1?000?????????1??????????????????????????????????????????????????????????????????????????????????????????????????????????????????????????????????????????????????????????????????????????????????????????????????????????????????????????????????????????????????????????????????????????????????????????????????????????????????????????????????????????????????????0???????????????????

Parasuchus_hislopi 2110201001111?00001210000014000-00--1-001000100-12-0020-110100200102002000210000-0001002??10?00?10020000110000100100-0-001010100-0101010100?11000100110020022-00-????101002-1011210000001131-12??????????????11010101110??-1111?1111100100010001-???0001???1011?01?0010?10001?110-2?10000?11010?01010?1??140?1-[0 2]1000?1001011110031[0 1]10010000110000?10000?-11?2?10010111000001-10000010-1???????00000001100?00100000?1000101120010101010111111-[1 2]?000[0 1]100????????0?01100100010110110200100001021?00?10???001100100100100002000?01101001001000101001200111-000000001111110011012211000100120311001011???201002021020011010000??01000001000000000

Nicrosaurus_kapffi 2?10101001111000001230000012000-00--0-001000?00-11-000--110100200102002000220?10-0-[0 1]1000??100-0100020000110000100100-0-??1000100-1100010000011001000011020022-00-0-[1 2]1101002-1011210000001131-12011----00020111101010111040-01??0111110000?000?0[1 2]-??1?000?????11????01111100010110-2010000010120110010?1??04011-[1 2][0 1]000?1?01?111100310[1 2]??1???0110000?1??000-1????0??1011?10000?-1????????1??02?????????????????????????00?1011??010001001111111-[1 2]?000????????????????????????????????????????????????????????00100001100002000?0?101?0?00100000101?210?????????????????????????????????????????????????????????????????????0?0010001?100?00000?

Smilosuchus_spp. ??1010100111100000122000001[2 3]000-00--0-001000?00-11-0-0--110000100102002000[2 3]10000-0-11000??100-01000[1 2]0010110000000100-0-001000100-1100010000011011100011020022-00-0-2-101002-10112100?0001131-12011----00020111101010111040-0101?112010000?010001-0110000?100?11??1?01111100010110-20100000110200010101?1?140?1-20100?1101011110031[0 1]1?01001011000001??000-11?2100010111100001-10010010-1???110?01000001100100111000-?00010?1??010001010111111-1?000?100????????????????000?011011010010000102120001000000??0010000010000200000110100?00100000001?210111-000000001111110011012211??01?0??0?1????00?????????2?210000?0000?00?0010000?100??00000

Ornithosuchus_longidens 2?00100100011002?011110001121100000210101300?00-01-0031-1101001002?000100001000000001101???20000000[1 2]0010000-0000010000-??100010111000010001011000100110030022-0100-2-101002-10?1[2 3]00000??1131-110?1----0002011110?0?0111??????????????????????????????????????????1?001?01?201?0??????0000?1?030?1???0?????4010021000?100??11110031?????????1?0?00?10??0?-11????10101?1?0?00?-1?0?10????0??010?01001001100?10011011110?????1?001??010101?0121-000??1100????????00??0???10010110110100100211011000022?0?00??00101100100102000?0110?0???11000????1?2?02????0??????112?110??00???????????1?0???00???????????02020-1001100[0 1]000?001000001?10000000

Riojasuchus_tenuisceps 2?00?-000001110210112100011221?0000210101300?00-01-0?31-1101000002?000100001001000001101??02000000010010000000002100-0-??100010000000010000011000100010030022-01-0-2-001002-1001200000001131-110[0 1]1----00020?1110?010111040-???1?111110000?01001?????0000???1?1???1?001201321??010-2100000010030?10010?11114010021000?1000000000031010020010110000?100000-1??[1 2]?00000111000001-100011???1??0?0???100?00?100?11?110111??????????011001010110121-100111??101??????010?????1001011[0 2][2 3]102001001110110000?20?????201100100100102000?0[1 2]100002111000100011210211-1000010011211101100122110011?0120302000001000?[0 1]?002020-000120000?0?0010000??010?00000

Nundasuchus_songeaensis 2??????????????????????????????????????????????????????????????????????????????????????????????????????????????????????????????????????????????????????????????????????????????????????????00?????3--001?2?????????????????????????????????????????????????????????0????0[0 1]11100??????000??21?20?0?????????4????21000?1?01?110100311?00????01?0?0??10??0?-11????0[0 3]10??110?001-1?00?010-1?????0101?00011100?100100110?01010??1??11001010110111-??????????????????????????1??????????????0??10112000???????1100101?0010000200000210100201100010001?200111-0000000011111100110122???01??012031?00?0110???0?0?2?20-10100011?0???01000???0??00?0?0

Turfanosuchus_dabanensis ?0001001?001100010?1[1 2]?0000110100000210201100?00-00?0020-110000001??0011000[1 2][1 2]001001101111??010001100100100?000000111100-??100010110011010010?11001000010?20??2-0010-??10?00??100120000000???001???13--?01?2????????1?1??040-2?01?110?10000?1100100???0000?10??110???0010000101?0?10??00000111020?0001??????40??021000?10010?11100310???[1 2]000??10000?10??10-?1??????20??1?01011-???00010-1???0000??????0???1?????????????????????10000000110111-100????0?????????????????00?1021020010?1?01010110000???????12001?0100?00002000?0110100?01100010101????111-10010?0011221110[1 2]101?????????01?0???????010?????????20-101?0011??0??0100000?01?0000?0

Gracilisuchus_stipanicicorum 2?001[0 1]11000110001011[1 2]0000011010000021020?200?00-00-0020-11[0 1]00010120001100021000001101111??01000110-10-1031000000110000-??10001111100101100-011001-00110020022-0110-2-10100[1 2]?100130100000???1-1?0?1----01020110101010111040-?[0 1]???010???????1?001[1 2]-????000?????1???1?001000?10100?10??0000011102000001010??14010[0 1]21000?1?01?11110031010?10?10110102?100010-11?11100200?1001011-100000?0-1?100000[0 1]???[0 1]??1????????????????????????10000001110121-??????????????????????????001021011000010000102100001?00000?000100100?00002000000111002[1 2]?100010???1100111-1000000011221110210122???01??0120302000001000?0?002020-10100011??0000100?????100??000

Aetosauroides_scagliai 2?0011000001100??????100001[2 3 4]0?0?00?21000?100?0??0[0 1]-0030-1110001?1??000?000?[1 2]0001-10?110[1 2]???????1??????????000000[0 1]100-0-??[0 1]???0??-?????????????????????????????00-????11?0????????????????????1???1??-?01????????????????????0????10?1000????????????????????????????21???01?10?10-[1 2]??100?????????????????14???0210???100?01111002101?0100?0100?00?101000-11?[1 2]?[0 1][0 1]020011000001-10000010-10[1 2 3]????1?1?0000?100?10??00????01010000?01[0 1]000010111111-100111100????????0???????01010310000100100001011100010000002200100100100002000002101002011000101011210111-1000000011221110111122110011?0120302000001000???????21020012000120??01000??1000000000

Batrachotomus_kupferzellensis 2?001001?0011002102?2100101101010?0110001200?10-0[0 1]00130-1110001?02100110001?0011-11010011-01000?10010?00?00000?0101000-001000101100110100?0011000?01010020?22-0010-2-101012-1?012000000??1?1-?1???----000201101011101?1040-11101110?100100110012-???0000121?11111??0010001001011102100000111020010??011??140??021000?1001111110031[0 1]???200101100000100000-11?[1 2]1[0 1]0020001101001-?00010?0-1???31?001?00001101010?100110??????????010000010110111-11011??00????????????????010?0211110101110?010210010?001000??0010110010000200000110100?01100010001?21011??1??????111221100?111?????????01?0???????12000?????2?20-10011011??000010000?100?000000

Prestosuchus_chiniquensis 200010010001110210212100001[0 1]0100000210001200?00-01-0020-11010010121001100012001100001001??0100010001001001000000000000-??100010110000110110011000001010020022-0010-2-101012-100110000000???1-110?1----01020?1?101010111040-11[0 1]1?110110000?110012-0010000??01?111?1?0010002000011102[0 1]00000111020010010111114010021000?1001?111100310100?00001100000100000-11?[1 2]100020001100001-100000?0-1??001?0011000011010100100110?0101011???10000010110111-1101???00????????????????0101021?110201110001021001110010101200101100100002000?01101002011000100011210111-10000000112211002111221100?1?0120?0?00?01[1 2]????0?0?2?20-1011001100000010000??00?000000

Dimorphodon_macronyx ?[1 2]00??0?0001100210?22?0000-3000001111000?200?00-11-0?20-111000100??000200?0??00?0??01002???20000000-------???????1??????????0000??00?010?01?1-00000??10?2-?1?????????????????001???0?0?????????????????????????????????????????????????????????????????????????????00??0001?1001????1000??????0??????????0[0 2 4]110[0 1]01000?1?0???????00--???1?????????????????-11????[0 1]010??1?0????-10????????????01??0001000100?10?21010-??????????010?110101101201???????????????0?0-0101110?10031?10021010000112??00?10???000?01100100?0000----10111?00300?0-00-??110?0-11-?0111???00-------??1?2111101?1?10403000111100?0?00010----------000?[0 1]01???????01???1?1

Lagerpeton_chanarensis ?????????????????????????????????????????????????????????????????????????????????????????????????????????????????????????????????????????????????????????????????????????????????????????????????????????????????????????????????????????????????????????????????????????????????????????????????????????????????????1?0????????0--????????????????????????????[1 3]?????????????1??00010-1???0???????????????????????????????????????????????????????????????????????????0011021?10020010000001130001000?00??01110101100002100?01111102101000001011101-11-1011100-00-------2-1-21111110112000000200000022--000?????????????????????????00?????1

Marasuchus_lilloensis [0 1]???????????10???????????????????????????????????[0 1]??????11?0??0????0?0?0001????????????????????????????????????????????????????????????0????10?????????????????????????????????12000?0?????????????????????????0?01?????41-1101?110???000?010002-?0?00[0 1]0?????1110?????????????????????????????????????????4010021??0?100101000000--10020010110?000100100-1????[1 2]10100????0011-1?000010-1011?000?001?001100?100010110??????????01?1?1010110121-1001?[1 2 3]10??????????????????011021210020011011001100001100?001200110100000112000?011100021000100110110?0211-011010001120000010011211111111120302000000000?2--00?0----------0??0?0?0??01?010??0??0

Lewisuchus_admixtus [0 1]?00????????11??00[1 2]???0?0????????????????????0???[0 1]-002??110??0000[1 2]0000?0002????????0????????00??10010-0031??????????????????0??01?001110????10000000?1002002???????????????????120000000???00????05-2-0102101?10111?111040-100111101100000010002-00110000??1?11101?????????????????????????????????????1??40?0[0 1]21000?1001?1111000--11010000110?00?100??0-110??12010001??0011-10???????????0????00110011000100110111???????????10?010?01[1 2]0?21-???????????????????????????????????????????????????????????????????????????????????????10?01001???????????????????????????????????????????????????????????????10-000-10?-0?0000?0??0??????1111?

Asilisaurus_kongwe 0?0?????????1?????????0?????????????????????????????????????????????????????0????0?01????????????????????????00????????????????????????????????????????????????????????????????1?0?????????????????????????????????????????????????????????????????????????????????0????0????0???????100??21????1????00???1????20001?1001?1111000--?1?2????1100000?0?1?0-1??2?1?0100?11000?1-1???01????????00???01?001100?10?110110?????????????0010101?0121-?????????????????????????000?0[1 2 3]?2100?00110?11011??0??1?1?0???0010110001111200010110??0?10001001111????111-0110100011210000100112???????1???????0?1??????????100----------?????01000?0?01?111110

Silesaurus_opolensis 00000-11?00?10??002????0001100000??2100?1200?0???1-103??1????00002100010001???00-0?011?2??1[1 2]000?10010??0??0?0000110100-00???0?0???011010?00??0010100?1002102??0010-2-111002-1??12000000????1-???[0 1]0----0102?01??0?11?111040-1?011110110000?01000000011[0 1]001201011101?00100010001111?1101000121010?1001000???10?0220111?1001?1111000--110[1 2][0 1]0101100000100100-11?2110010001100001-10001110-10100111[0 1]0?110011000100110111??????????010001000110121-000003100????????????????000[1 2]02121021001102100[1 2]1000021110001?001011[0 1]0011112000101110002101110011111101211-0211100000-------20112???1???1021?020001000???2--0100----------000000110000?000111110

Heterodontosaurus_tucki 00000-02?00110000020210101110001000200011300100-01-00[2 3]3-110000000200101000110000-1001012??0100011000001000000000110010-002--110010001010000011001000011020022-00-0-2-111002-11?1200001001?31-???1?----010210???0111?111041-1001?110210000?010002-??11000???1?111?1?02100021001010-101001012012111001000??0411012[0 1]110?1001?0000000--11?00010100?10?100000-11??0?1?10111?00001-100?20???10?0000121?11001100?10?0??10-11-------0110?110?0110121-1001011001-100110101101121011031011001011020112--00-32?1?0011011?0?0??-?102110?0?1?0?022001110?1101101-11-?2111?0-00-------201?2111101010213020001000??22--0000----------00000011??00?000??-101

Herrerasaurus_ischigualastensis 2?000-0100011011002121001011000100020000?200?00-01001200110000000[1 2]130010002?0000-0001102??01000010010-0030000000?10010-002--0[0 1]00110010100000[0 1]0000000010020022-0010-2-101002-10?130000000???1-1???0----01?210?0101110111040-?0???111110000?010?12-????000???1?????1?001100[1 2]0011011010000001[1 2]1020010010?[0 1]1114010021000?1?01?1111002001102001011001[0 1]0100100-11?2?[0 1]00100?1100001-10000010-101??01000?11000100011?110111??????????110011011120121-1001011001-1111001[1 2]11111210110212111200110[1 2]10121001112110103?01110100012112010?01110[0 1]0220[0 1]011011101101211-0211100000-------2010211110101121302000110000?1000000----------000?001100?1?00010-100

Yarasuchus_deccanensis ?????????????????????????????????????????????????????????????????????????????????????????????????????????????????????????????????????????????????????????????????????????????????????????????????????????????????????????????????????????????????????????????????????????????????????????????????????????????????????100111111000--[1 2]??1???[0 1]2100110100010-?1???10010111001001-1???0010-1???30???1?0?00??00????00000-???????????10000000110111-00010????????????????????00000??21100001?010001100002011000210010100010010200000112100200100000???????11???0?????0011010001001??????????????????????????????????????????-??????11001??00?1?1??0

Dongusuchus_efremovi ??????????????????????????????????????????????????????????????????????????????????????????????????????????????????????????????????????????????????????????????????????????????????????????????????????????????????????????????????????????????????????????????????????????????????????????????????????????????????????????????????????????????????????????????????????????????????????????????????????????????????????????????????????????????????????????????????????????????????????????????????????????0010100010010200000112100?????????????????????0????????????????????????????????????????????????????????????????????????????????1??

Teleocrater_combined 2?000-0?????100?0???????0????????????????????????[0 1]?0020-1?0??010021000?0001????????????????????????????????????0?10110-00??????????????????????????????????????????????????????130?0100????????????????????????0?0??1?10???110???10?10000????????????????????1????????????????????????????????????????????4????21????100111111000--??01001?210010010?010-1???11001?101101001-1???00?0-1010?00????01?01100010??????????????????10000000110111-00010??00????????????????000?0212????0010??0?????????011000??0010100010010200000112100200100000101110011???0?????0011010001001?????????????????????????????????????????????00?01100?0?0??111110

Spondylosoma_absconditum ?????????????????????????????????????????????????????????????????????????????????????????????????????????????????????????????????????????????????????????????????????????????????????????????????????????????????????????????????????????????????????????????????????????????????????????????????????????????????????1?01?1111000--??????????0??1?10??10-1????1??100?110???1?1??0101??1????????1????011000??????????????????????00101?????????????????????????????????????????????????0?01?[1 2]1???????????????????????????????0?11100?????????????????????????????????????????????????????????????????????????????????????????1100??????11????

;

cnames

{0 Skull_and_lower_jaws,_interdental_plates absent present,_small_and_well-spaced_from_each_other present,_large_and_close_to_or_contacting_with_each_other;

{1 Skull,_total_length_versus_length_of_the_presacral_vertebral_column 0.22-0.38 0.44-0.72 0.94-0.98;

{2 Skull,_strongly_dorsoventrally_compressed_skull_with_mainly_dorsally_facing_antorbital_fenestrae_and_orbits absent present;

{3 Skull,_well-developed_nodular_prominences_on_the_lateral_surface_of_maxilla,_jugal,_quadratojugal,_squamosal_and_angular absent present;

{4 Skull,_dermal_sculpturing_on_the_dorsal_surface_of_the_skull_roof absent shallow_or_deep_pits_scattered_across_surface_and_low_ridges prominent_ridges_or_tubercles_on_frontals,_parietals,_and_nasals;

{5 Skull,_dorsal_surface_of_nasals_and/or_frontals_ornamented_by_ridges_radiating_from_centres_of_growth absent present;

{6 Skull,_dorsal_orbital_margin orbital_margin_of_the_frontal_level_with_skull_table_or_raised_slightly orbital_margin_of_the_frontal_elevated_above_skull_table shelf/ridge_elevated_above_skull_table_and_extends_along_the_lateral_surface_of_the_lacrimal,_prefrontal,_frontal_portion_of_orbital_rim,_and_postorbital;

{7 Skull,_dorsal_surface_of_the_temporal_region flat supratemporal_fossa_immediately_medial_or_anterior_to_the_supratemporal_fenestra thin,_blade-like_median_sagittal_crest;

{8 External_nares,_confluent absent present;

{9 External_naris,_anteroposterior_position_in_the_snout terminal,_on_the_anterior_end_of_the_snout nonterminal,_considerably_posteriorly_displaced,_but_posterior_rim_of_the_naris_well_anterior_to_the_anterior_border_of_the_orbit nonterminal,_considerably_posteriorly_displaced_and_posterior_rim_of_the_naris_approximately_at_level_with_the_anterior_border_of_the_orbit;

{10 External_naris,_directed laterally dorsally anteriorly;

{11 External_naris,_shape sub-circular oval;

{12 Antorbital_fenestra absent present;

{13 Antorbital_fenestra,_anterior_margin gently_rounded nearly_pointed;

{14 Secondary_antorbital_fenestra,_immediately_anterior_to_the_antorbital_fenestra absent present;

{15 Orbit,_shape anteroposteriorly_longer_than_tall subcircular dorsoventrally_taller_than_long;

{16 Orbit,_elevated_rim_along_the_jugal,_postorbital,_frontal,_prefrontal_and_lacrimal absent_or_incipient present,_restricted_to_the_ascending_process_of_the_jugal_and_sometimes_also_onto_the_ventral_process_of_the_postorbital present,_well-developed_along_the_jugal,_postorbital,_frontal,_prefrontal_and_lacrimal;

{17 Infratemporal_fenestra present absent;

{18 Posttemporal_fenestra,_size larger_than_or_subequal_to_the_supraoccipital smaller_than_the_supraoccipital developed_as_a_small_foramen_ absent;

{19 Snout,_antorbital_length_(anterior_tip_of_the_skull_to_anterior_margin_of_the_orbit)_versus_total_length_of_the_skull 0.29-0.40 0.43-0.62 0.70-0.76;

{20 Snout,_dorsoventral_height_at_the_level_of_the_anterior_tip_of_the_maxilla_versus_dorsoventral_height_at_the_level_of_the_anterior_border_of_the_orbit <=0.33 0.42-0.52 0.59-0.80;

{21 Snout,_proportions_at_the_level_of_the_anterior_border_of_the_orbit transversely_broader_than_dorsoventrally_tall_or_subequal dorsoventrally_taller_than_transversely_broad;

{22 Snout,_lateral_margin_of_the_snout_anterior_to_the_prefrontal formed_by_the_nasal formed_by_the_nasal_and_maxilla_with_gently_rounded_transition_along_the_maxilla_from_the_lateral_to_dorsal_side_of_rostrum formed_by_the_nasal_and_maxilla_with_sharp_edge_along_the_maxilla_between_the_lateral_and_dorsal_sides_of_this_bone_(=_box-like_snout_of_Kischlat,_2000);

{23 Premaxilla-maxilla,_suture simple_continuous_contact notched_along_the_ventral_margin;

{24 Premaxilla-maxilla,_subnarial_foramen_between_the_elements absent present_and_the_border_of_the_foramen_is_present_on_both_the_maxilla_and_the_premaxilla present_and_the_border_of_the_foramen_is_present_on_the_maxilla_but_not_on_the_premaxilla;

{25 Premaxilla,_alveolar_margin_does_not_reach_the_contact_with_the_maxilla_and_forms_a_diastema_(=_subnarial_gap) absent present;

{26 Premaxilla,_main_body_size small,_the_premaxillary_body_forms_less_than_half_of_snout_in_front_of_the_posterior_border_of_the_external_nares large,_the_premaxillary_body_forms_half_or_more_than_half_of_snout_in_front_of_the_posterior_border_of_the_external_nares;

{27 Premaxilla,_anteroposterior_length_of_the_main_body_versus_its_maximum_dorsoventral_height 0.70-0.73 1.07-2.00 2.22-3.80 4.15-4.68 >5.00;

{28 Premaxilla,_downturned_main_body absent,_alveolar_margin_sub-parallel_to_the_main_axis_of_the_maxilla slightly,_alveolar_margin_kinked_approximately_20º_from_the_alveolar_margin_of_the_maxilla strongly,_prenarial_process_obscured_by_the_postnarial_process_in_lateral_view_(if_the_postnarial_process_is_long_enough)_and_postnarial_process_parallel_or_posteroventrally_oriented_with_respect_to_the_main_axis_of_the_premaxillary_body;

{29 Premaxilla,_angle_formed_between_the_alveolar_margin_and_the_anterior_margin_of_the_premaxillary_body_in_lateral_view acute_or_right-angled obtuse;

{30 Premaxilla,_longitudinal_groove_placed_approximately_at_mid-height_and_extending_along_most_of_the_length_of_the_lateral_surface_of_the_main_body_of_the_bone absent present one_longitudinal_groove_slightly_displaced_ventrally_or_at_the_point_of_mid-height_of_the_main_body;

{31 Premaxilla,_narial_fossa absent_or_shallow expanded_in_the_anteroventral_corner_of_the_naris_;

{32 Premaxilla,_peg_on_the_posterior_edge_of_the_premaxillary_body absent present;

{33 Premaxilla,_prenarial_process_length less_than_the_anteroposterior_length_of_the_main_body_of_the_premaxilla greater_than_the_anteroposterior_length_of_the_main_body_of_the_premaxilla;

{34 Premaxilla,_base_of_the_prenarial_process anteroposteriorly_shallow anteroposteriorly_deep;

{35 Premaxilla,_postnarial_process absent short,_ends_well_anterior_to_the_posterior_margin_of_the_external_naris well-developed,_forms_most_of_the_border_of_the_external_naris_or_excludes_the_maxilla_from_participation_in_the_external_naris;

{36 Premaxilla,_postnarial_process wide,_platelike thin;

{37 Premaxilla,_sharp_dorsal_flange_at_the_base_of_the_postnarial_process_delimiting_the_posteroventral_border_of_the_external_naris absent present;

{38 Premaxilla,_postnarial_process fits_between_the_nasal_and_the_maxilla_or_lies_on_the_anterodorsal_surface_of_the_maxilla overlaps_the_anterodorsal_surface_of_the_nasal fits_into_slot_of_the_nasal_;

{39 Premaxilla,_contact_with_prefrontal absent present,_marginal present,_extensive;

{40 Premaxilla,_palatal_process_on_the_medial_surface absent present;

{41 Premaxilla,_number_of_tooth_positions 10_or_more 5_or_more 4 three 2 1_or_edentulous;

{42 Premaxilla,_orientation_of_the_tooth_series_or_the_occlusal_surface_of_premaxilla_in_ventral_view approximately_parasagittal strongly_transverse_and_anterior_teeth_covering_each_other_in_lateral_view;

{43 Premaxilla,_lateroventrally_opened_anterior_alveoli_in_mature_individuals absent present;

{44 Septomaxilla present absent;

{45 Maxilla-nasal,_maxillo-nasal_tuberosity,_delimiting_anteriorly_the_antorbital_fossa_if_present absent present;

{46 Maxilla-jugal,_anguli_oris_crest absent present;

{47 Maxilla-jugal,_anterior_extension_of_the_anguli_oris_crest restricted_to_the_main_body_of_the_jugal extending_onto_the_maxilla,_but_not_the_anterior_process_of_the_jugal;

{48 Maxilla,_anterior_extent posterior_to_the_anterior_extent_of_the_nasals anterior_to_the_nasals;

{49 Maxilla,_length_of_the_portion_of_the_bone_anterior_to_the_antorbital_fenestra_versus_the_total_length_of_the_bone 0.12-0.22 0.29-0.60 0.64-0.76;

{50 Maxilla,_posterior_border_of_the_subnarial_foramen_extending_posteriorly_as_a_groove_on_the_lateral_surface_of_the_anterior_process absent present;

{51 Maxilla,_anterior_maxillary_foramen absent present;

{52 Maxilla,_neurovascular_foramina_on_the_lateral_surface_of_the_anterior_and_horizontal_processes laterally_or_lateroventrally_facing lateroventrally_facing_and_extending_ventrally_as_deep,_well-defined_grooves;

{53 Maxilla,_antorbital_fossa_on_the_lateral_surface_of_the_bone absent_or_not_exposed_in_lateral_view present_on_the_ascending_process_of_the_maxilla,_but_not_along_the_horizontal_process_of_the_maxilla present_on_the_horizontal_process_of_the_maxilla,_but_not_reaching_the_posteroventral_corner_of_the_fenestra_ present_on_the_horizontal_process_of_the_maxilla,_reaching_the_posteroventral_corner_of_the_opening;

{54 Maxilla,_anteroposterior_length_of_the_antorbital_fossa_anterior_to_the_antorbital_fenestra_versus_length_of_the_antorbital_fenestra 0.09-0.23 0.28-0.43 0.90-0.94 >2.00;

{55 Maxilla,_secondary_antorbital_fossa_anteriorly_to_the_antorbital_fossa_and_adjacent_to_the_dorsal_margin_of_the_anterior_process absent present;

{56 Maxilla,_ascending_process absent present;

{57 Maxilla,_ascending_process_shape simply_tapers_to_a_point_dorsally the_dorsal_apex_of_the_maxilla_is_a_separate,_distinct_process_that_has_a_posteriorly_concave_margin sub-vertical_anterior_margin_of_the_base_of_the_process;

{58 Maxilla,_anterodorsal_margin_at_the_base_of_the_ascending_process convex_or_straight concave;

{59 Maxilla,_ascending_process_remains_the_same_width_for_its_length absent present;

{60 Maxilla,_contact_with_prefrontal absent present;

{61 Maxilla,_ventral_margin_of_the_antorbital_fossa_or_fenestra_(if_the_antorbital_fossa_is_absent_from_the_horizontal_process_of_the_maxilla)_in_the_horizontal_process mainly_sub-parallel_to_the_alveolar_margin_of_the_bone diagonal,_anteroventrally-to-posterodorsally_oriented_in_an_angle_close_to_45º;

{62 Maxilla,_shape_of_the_posterior_portion_of_the_bone_(ventral_to_the_antorbital_fenestra_if_it_is_present) tapers_posteriorly has_a_similar_dorsoventral_depth_as_the_anterior_portion_ventral_to_the_antorbital_fenestra expands_dorsoventrally_towards_the_distal_end_of_the_horizontal_process_with_a_concave_ventral_margin_of_the_antorbital_fenestra expands_dorsoventrally_towards_the_distal_end_of_the_horizontal_process_with_a_straight_ventral_margin_of_the_antorbital_fenestra;

{63 Maxilla,_posterior_end_of_the_horizontal_process_distinctly_ventrally_deflected_from_the_maxin_axis_of_the_alveolar_margin absent present;

{64 Maxilla,_triangular_dorsal_process_with_clear_dorsal_apex_formed_by_discrete_expansion_of_the_posterior_end_of_the_horizontal_process_in_lateral_view absent present;

{65 Maxilla,_palatal_process_on_the_anteromedial_surface_of_the_bone absent present_and_both_counterparts_do_not_meet_at_the_midline_ present_and_both_counterparts_meet_at_the_midline;

{66 Maxilla,_position_of_the_palatal_process adjacent_to_the_base_of_the_interdental_plates distinctly_dorsally_to_the_base_of_the_interdental_plates;

{67 Maxilla,_alveolar_margin_in_lateral_view concave,_straight_or_gently_convex distinctly_convex sigmoid,_anteriorly_concave_and_posteriorly_convex_ sigmoid,_anteriorly_convex,_starting_close_to_mid-length,_and_posteriorly_concave;

{68 Maxilla,_edentulous_anterior_portion_of_the_ventral_margin_of_the_bone absent present;

{69 Maxilla,_alveolar_margin_on_the_anterior_third_of_the_bone_(anterior_to_the_level_of_the_anterior_border_of_the_antorbital_fenestra_if_present) approximately_aligned_to_the_posterior_half_of_the_alveolar_margin abruptly_upturned;

{70 Maxilla,_posterior_extension_in_mature_individuals at_level_or_posterior_to_posterior_orbital_border anterior_to_posterior_orbital_border_but_posterior_to_anterior_orbital_border at_level_or_anterior_to_anterior_orbital_border;

{71 Maxilla,_tooth_plate absent present;

{72 Maxilla,_number_of_tooth_rows single_row multiple_rows;

{73 Maxilla,_location_of_teeth only_on_occlusal_surface on_occlusal_and_lingual_surfaces;

{74 Maxilla,_number_of_tooth_positions 8-9_ 10-14_ 15-22 23-35 36-40;

{75 Nasal,_total_length_versus_total_length_of_the_frontal 0.68-0.79 0.92-2.07 2.26-3.09;

{76 Nasal,_exposure_(excluding_descending_process_if_present) largely_dorsal_element nearly_vertical_contribution_to_the_snout;

{77 Nasal,_shape_of_anterior_margin_at_midline strongly_convex_with_anterior_process transverse_with_little_convexity;

{78 Nasal,_anterior_portion_in_lateral_view below_or_at_the_same_level_as_skull_roof elevated_above_skull_roof,_giving_the_skull_a_?Roman_nose?_appearance;

{79 Nasal,_dorsal_surface_around_posterior_margin_of_external_naris smooth_or_sculpturing_of_ridges_and_grooves_present depression_around_entire_posterior_margin_that_lacks_sculpturing;

{80 Nasal,_descending_process,_which_results_from_the_articulation_of_the_postnasal_process_of_the_premaxilla_on_the_anterodorsal_surface_of_the_nasal_and_has_an_extensive_contact_with_the_ascending_process_of_the_maxilla anteroposteriorly_narrow anteroposteriorly_very_broad,_being_considerably_broader_than_the_ascending_process_of_the_maxilla;

{81 Nasal,_dorsolateral_margin_of_the_anterior_portion smoothly_rounded distinct_longitudinal_ridge_on_the_lateral_edge;

{82 Nasal,_participation_in_the_dorsal_border_of_the_antorbital_fossa absent present;

{83 Lacrimal-postorbital,_contact_between_bones absent present;

{84 Lacrimal,_participation_in_the_posterior_border_of_the_external_naris present absent;

{85 Lacrimal,_exposure_on_the_skull_roof_in_dorsal_view absent_or_marginal present;

{86 Lacrimal,_anterior_process_forming_the_entire_or_almost_the_entire_dorsal_border_of_the_antorbital_fenestra absent present;

{87 Lacrimal,_antorbital_fossa_forming_a_distinct_inset_margin_to_the_antorbital_fenestra_on_the_lateral_surface_of_the_bone absent present,_but_strongly_restricted_anteirorly present_and_occupies_almost_half_or_more_of_the_anteroposterior_length_of_the_ventral_process_;

{88 Lacrimal,_naso-lacrimal_duct completely_enclosed_by_the_lacrimal enclosed_by_the_lacrimal_and_prefrontal;

{89 Lacrimal,_naso-lacrimal_duct_position opens_on_the_posterolateral_edge_of_the_lacrimal opens_on_the_posterior_surface_of_the_lacrimal;

{90 Jugal-quadratojugal,_ventral_margin_in_lateral_view straight_or_convex concave,_though_nowhere_dorsal_to_tooth_row;

{91 Jugal,_anterior_process_shape_in_lateral_view continuously_tapering_or_subrectangular,_being_lower_than_the_portion_of_the_maxilla_underneath_it subrectangular_or_slightly_dorsoventrally_expanded,_being_higher_than_the_portion_of_the_maxilla_underneath_it with_an_ascending_subprocess_excluding_the_lacrimal_from_the_anteroventral_border_of_the_orbit;

{92 Jugal,_anterior_process_continuously_dorsally_curved absent,_straight_or_curved_only_at_its_proximal_half present;

{93 Jugal,_ventral_border_of_the_orbit:_ gently_concave V-shaped;

{94 Jugal,_anterior_extension_of_the_anterior_process anterior_to_the_level_of_mid-length_of_the_orbit up_to_or_posterior_to_the_level_of_mid-length_of_the_orbit;

{95 Jugal,_participation_of_the_anterior_process_in_the_border_of_the_antorbital_fenestra present absent,_excluded_by_contact_between_the_maxilla_and_lacrimal;

{96 Jugal,_longitudinal_ridge_or_bump(s)_on_the_lateral_surface_of_the_main_body absent present;

{97 Jugal,_multiple_pits_on_the_lateral_surface_of_the_main_body absent present;

{98 Jugal,_ascending_process_forming_the_entire_anterior_border_of_the_infratemporal_fenestra absent present,_postorbital_excluded_from_the_anterior_border_of_the_infratemporal_fenestra;

{99 Jugal,_length_of_the_posterior_process_versus_the_height_of_its_base 0.49-1.27 1.59-3.77 4.07-5.37;

{100 Jugal_posterior_process_with_a_distinct_lateroventral_orientation_with_respect_to_the_sagittal_axis_of_the_snout absent present;

{101 Jugal,_distal_half_of_the_posterior_process tapering subrectangular;

{102 Jugal,_posterior_process_forms_entirely_or_almost_entirely_the_ventral_border_of_the_infratemporal_fenestra_(it_also_applies_in_the_lower_temporal_bar_is_incomplete) absent present;

{103 Jugal,_base_of_the_posterior_process_with_a_semi-elliptical,_ventral_expansion_in_lateral_view absent present;

{104 Jugal,_posterior_process lies_dorsal_to_the_anterior_process_of_the_quadratojugal lies_ventral_to_the_anterior_process_of_the_quadratojugal splits_the_anterior_process_of_the_quadratojugal is_splited_by_the_anterior_process_of_the_quadratojugal_;

{105 Jugal,_posterior_termination_of_the_posterior_process anterior_to_or_at_level_with_the_posterior_border_of_the_infratemporal_fenestra posterior_to_the_infratemporal_fenestra;

{106 Prefrontal,_contact_its_counterpart_in_the_median_line_of_the_skull_roof absent present;

{107 Prefrontal,_suture_with_the_nasal parasagittal,_at_least_in_its_posterior_third,_or_anterolateral anteromedial;

{108 Prefrontal,_subtriangular_medial_process absent,_nasal-frontal_suture_transversely_broad present,_nasal-frontal_suture_strongly_transversely_reduced;

{109 Prefrontal,_groove_on_the_lateral_surface_of_the_main_body_opening_into_the_orbital_border absent present;

{110 Prefrontal,_lateral_surface_of_the_orbital_margin smooth_or_slight_grooves_present rugose_sculpturing_present;

{111 Frontal,_frontals_fused_to_one_another absent present;

{112 Frontal,_suture_with_the_nasal transverse oblique,_forming_an_angle_of_at_least_60°_with_long_axis_of_the_skull_and_frontals_entering_between_both_nasals oblique_and_nasals_entering_considerably_between_frontals_in_a_non-interdigitate_suture;

{113 Frontal,_orbital_border absent_or_anteroposteriorly_short anteroposteriorly_long_and_forms_most_of_the_dorsal_edge_of_the_orbit;

{114 Frontal,_dorsal_surface flat_or_slightly_depressed with_longitudinal_ridge_along_midline;

{115 Frontal,_suture_with_parietal mostly_transverse_or_parietals_slightly_entering_between_frontals_on_the_median_line,_forming_an_obtuse-angled_suture parietals_strongly_entering_between_both_frontals,_forming_an_acute-angled_suture W-shaped_suture;

{116 Frontal,_participates_on_the_anteromedial_corner_of_the_supratemporal_fossa absent present;

{117 Frontal,_dorsal_surface_adjacent_to_sutures_with_the_postfrontal_(if_present)_and_parietal flat_to_slightly_concave possesses_a_longitudinal_and_deep_depression;

{118 Frontal,_longitudinal_groove longitudinally_extended_along_most_of_the_surface_of_the_frontal anterolaterally-to-posteromedially_extended_along_the_posterior_half_of_the_frontal;

{119 Frontal,_ventral_surface hourglass-shaped_median_longitudinal_canal_for_the_passage_of_the_olfactory_duct_and_olfactory_bulb_moulds_on_the_anterior_end_of_the_bone median_longitudinal_canal_for_the_passage_of_the_olfactory_duct_only_slightly_constricted,_no_olfactory_bulb_moulds_and_distinct_semilunate_posteromedially-to-anterolaterally_oriented_ridge_on_the_orbital_roof,_extending_onto_the_prefrontal;

{120 Frontal,_olfactory_tract_on_the_ventral_surface_of_the_frontal maximum_transverse_constriction_point_well_posterior_to_the_moulds_of_the_olfactory_bulbs_and_posterolateral_margin_of_the_bulbs_delimited_by_a_low_ridge maximum_transverse_constriction_of_the_olfactory_bulbs_immediately_posterior_to_the_moulds_of_the_olfactory_bulbs_and_posterolateral_margin_of_the_bulbs_well_delimited_by_a_thick,_tall_ridge;

{121 Postfrontal equivalent_in_size_to_postorbital reduced_to_approximately_less_than_half_the_size_of_the_postorbital absent;

{122 Postfrontal,_participation_in_the_border_of_the_supratemporal_fenestra absent present;

{123 Postfrontal,_shape_of_dorsal_surface flat_or_slightly_concave_towards_raised_orbital_rim depression_with_deep_pits;

{124 Postorbital-jugal,_postorbital_bar composed_by_both_jugal_and_postorbital_in_nearly_equal_proportion composed_mostly_by_the_postorbital;

{125 Postorbital-squamosal,_upper_temporal_bar located_approximately_at_level_of_mid-height_of_the_orbit located_approximately_aligned_to_the_dorsal_border_of_the_orbit;

{126 Postorbital-squamosal,_contact restricted_to_the_dorsal_margin_of_the_elements continues_ventrally_for_much_or_most_of_the_ventral_length_of_the_squamosal,_but_squamosal_does_not_contact_jugal continues_ventrally_for_much_or_most_of_the_ventral_length_of_the_squamosal_and_squamosal_contacts_jugal;

{127 Postorbital,_lateral_boss_adjacent_to_orbital_margin absent present;

{128 Postorbital,_supratemporal_fossa_extending_onto_the_ascending_process absent present;

{129 Postorbital,_posterior_process_extends_close_to_or_beyond_the_level_of_the_posterior_margin_of_the_supratemporal_fenestrae absent present;

{130 Postorbital,_extension_of_the_ventral_process ends_much_higher_than_the_ventral_border_of_the_orbit ends_close_to_or_at_the_ventral_border_of_the_orbit;

{131 Postorbital,_ventral_process_in_lateral_view continuously_anteriorly_curved_or_straight distinctly_anteriorly_flexed;

{132 Postorbital,_depression_on_the_lateral_surface_of_the_ventral_process absent present;

{133 Postorbital,_anteriorly_projecting,_rounded_spur_on_the_anterior_edge_of_the_ventral_process_indicating_the_lower_delimitation_of_the_eyeball absent present;

{134 Squamosal,_completely_covering_the_quadrate_in_lateral_view present absent;

{135 Squamosal,_overhanging_quadrate_laterally absent present;

{136 Squamosal,_anterior_process_forms_more_than_half_of_the_lateral_border_of_the_supratemporal_fenestra absent present;

{137 Squamosal,_anteroventral_process absent present;

{138 Squamosal,_transition_between_the_anterior_and_ventral_processes sharp,_squared_posterodorsal_border_of_the_infratemporal_fenestra gentle,_widely_rounded_posterodorsal_border_of_the_infratemporal_fenestra;

{139 Squamosal_medial_process short,_forming_approximately_half_or_less_of_the_posterior_border_of_the_supratemporal_fenestra long,_forming_entirely_or_almost_entirely_the_posterior_border_of_the_supratemporal_fenestra;

{140 Squamosal,_posterior_process does_not_extend_posterior_to_the_head_of_the_quadrate extends_posterior_to_the_head_of_the_quadrate;

{141 Squamosal,_posterior_process_shape straight_ ventrally_curved;

{142 Squamosal,_ventral_process present absent;

{143 Squamosal,_ventral_process_shape anteroposteriorly_broad_and_plate-like anteroposteriorly_narrow_and_strap-like;

{144 Squamosal,_ventral_process_orientation posteroventrally_directed,_vertical,_or_more_than_45º_from_the_vertical anteroventrally_directed_at_45º_or_less;

{145 Squamosal,_contribution_of_the_ventral_process_to_the_posterior_border_of_the_infratemporal_fenestra forms_less_than_half_of_the_border_of_the_fenestra forms_more_than_half_of_the_border,_but_quadratojugal_or_quadrate_broadly_participates_in_the_border_of_the_fenestra forms_almost_completely_the_border_of_the_fenestra;

{146 Squamosal,_posterodorsally-to-anteroventrally_oriented_tuck_on_the_lateral_surface_of_the_ventral_process absent present;

{147 Squamosal,_longitudinal_ridge_on_the_lateral_surface_of_the_ventral_process absent present;

{148 Squamosal,_posterodorsal_portion_with_a_supratemporal_fossa absent present;

{149 Quadratojugal absent_or_fused_to_the_quadrate present;

{150 Quadratojugal,_shape L-shaped_or_strip-like_bone subtriangular;

{151 Quadratojugal,_infratemporal_fossa_marked_by_a_sharp_edge absent present;

{152 Quadratojugal,_anterior_process absent,_anteroventral_margin_of_the_bone_rounded incipient,_short_anterior_prong_on_the_anteroventral_margin_of_the_bone distinctly_present,_in_which_the_lower_temporal_bar_is_complete,_but_process_terminatesnwell_posterior_to_the_base_of_the_posterior_process_of_the_jugal distinctly_present,_in_which_the_lower_temporal_bar_is_complete_and_participates_in_the_posteroventral_border_of_the_infratemporal_fenestra,_and_process_terminates_close_to_the_base_of_the_posterior_process_of_the_jugal;

{153 Quadratojugal,_widely_concave_notch_on_the_anterior_margin_of_the_ascending_process absent present;

{154 Quadratojugal,_depression_along_the_posterior_half_of_the_ascending_process_up_to_the_exposed_lateral_surface_of_its_distal_tip absent present;

{155 Quadratojugal,_posterior_extension_of_the_ventral_end absent,_without_a_posteriorly_arched_quadratojugal limited,_ventral_condyles_of_the_quadrate_broadly_visible_in_lateral_view_ strongly_developed,_overlapping_completely_or_almost_completely_the_ventral_condyles_of_the_quadrate_in_lateral_view_;

{156 Supratemporal broad_element slender,_in_parietal_and_squamosal_trough absent;

{157 Supratemporal,_bifurcated_medial_border,_in_which_a_ventromedial_process_extends_underneath_the_posterolateral_process_of_the_parietal present absent;

{158 Parietal,_median_contact_between_both_parietals suture_present fused_with_loss_of_suture;

{159 Parietal,_extension_over_interorbital_region absent_or_slight present;

{160 Parietal,_supratemporal_fossa_medial_to_the_supratemporal_fenestra well_exposed_in_dorsal_view_and_mainly_dorsally_or_dorsolaterally_facing poorly_exposed_in_dorsal_view_and_mainly_laterally_facing;

{161 Parietal,_pineal_fossa_on_the_median_line_of_the_dorsal_surface absent present;

{162 Parietal,_position_of_the_pineal_fossa restricted_to_the_parietal extended_along_frontal_and_parietal;

{163 Parietal,_pineal_foramen large reduced_to_a_small,_circular_pit absent;

{164 Parietal,_position_of_the_pineal_foramen_in_dorsal_view completely_enclosed_by_parietals_in_the_anterior_half_of_the_bone_(excluding_posterolateral_processes_of_the_parietals) completely_enclosed_by_parietals_close_to_mid-length_or_in_the_posterior_half_of_the_bone_(excluding_posterolateral_processes_of_the_parietals) enclosed_by_both_frontals_and_parietals;

{165 Parietal,_distinct_transverse_emargination_adjacent_to_the_posterior_margin_of_the_bone_in_late_ontogeny absent present;

{166 Parietal,_posterolateral_process nearly_vertical ventrally_inclined_greater_than_45º;

{167 Parietal,_posterolateral_process_height dorsoventrally_low,_usually_considerably_lower_than_the_supraoccipital dorsoventrally_very_deep,_being_plate-like_in_occipital_view_and_subequal_to_the_height_of_the_supraoccipital;

{168 Parietal,_posterolateral_process_with_a_strongly_transversely_convex_dorsal_margin_elevated_from_the_median_line_of_the_posterior_margin_of_the_skull_roof absent present;

{169 Parietal,_tuberosity_on_the_posterior_surface_of_the_base_of_the_posterolateral_process absent present;

{170 Postparietal,_size_(pair_of_postparietals_if_they_are_not_fused_to_each_other) sheet-like,_not_much_narrower_than_the_suproccipital small,_splint-like absent_as_a_separate_ossification;

{171 Postparietal,_fusion_between_counterparts absent present,_forming_an_interparietal;

{172 Tabular present absent;

{173 Palpebral/s absent present;

{174 Neomorphic_bone_(=_septomaxilla_of_phytosaurs),_separate_ossification_anterior_to_nasals_and_surrounded_by_the_premaxilla_on_the_dorsal_surface_of_the_snout absent present;

{175 Quadrate,_shape straight_posteriorly shallowly_emarginated with_conch;

{176 Quadrate,_angle_between_the_posterior_margins_of_the_dorsal_and_ventral_ends 41-47º 91-96º 106-137º 143-158º;

{177 Quadrate,_dorsal_head does_not_have_a_sutural_contact_with_the_paroccipital_process_of_the_opisthotic has_a_sutural_contact_with_the_paroccipital_process_of_the_opisthotic;

{178 Quadrate,_dorsal_head partially_exposed_laterally completely_covered_by_the_squamosal;

{179 Quadrate,_dorsal_end_hooked_posteriorly_in_lateral_view absent present;

{180 Quadrate,_foramen_on_the_medial_wall_of_the_quadrate_foramen absent present;

{181 Quadrate,_posterior_margin_of_the_ventral_half_in_lateral_view concave convex;

{182 Quadrate,_ventral_condyles subequally_distally_extended medial_condyle_distinctly_more_distally_projected_than_the_lateral_one;

{183 Neomorph_ossification,_present_between_the_pterygoid,_quadrate_and_skull_roof absent,_the_quadrate_flange_of_the_pterygoid_meets_the_quadrate_but_remains_free_of_the_skull_roof present;

{184 Vomer,_shape broad,_plate-like_bone,_at_least_as_transversely_broad_as_the_internal_naris stick-like_bone,_transversely_narrower_than_the_internal_naris;

{185 Vomer,_contact_with_maxilla absent present;

{186 Vomer,_teeth present,_more_than_one_row_or_no_rows_are_distinguishable_ present,_mainly_in_a_single_row,_but_multiple_teeth_present_immediately_anterior_to_the_contact_with_the_pterygoid present,_single_row_along_entire_extension_ absent;

{187 Palatine-pterygoid,_teeth_on_the_palatine_and_ventral_surface_of_the_anterior_ramus_of_the_pterygoid present absent;

{188 Palatine-pterygoid,_height_and_dimatre_of_teeth_on_the_palatine,_ventral_surface_of_the_anterior_ramus_of_the_pterygoid_and_vomer considerably_smaller_than_those_of_the_marginal_dentition_ similar_to_those_of_the_marginal_dentition;

{189 Palatine,_transverse_extension narrow,_subequal_contribution_of_the_palatine_and_pterygoid_to_or_pterygoid_main_component_of_the_palate_posteriorly_to_the_choanas broad,_the_palatine_is_the_main_component_of_the_palate_posteriorly_to_the_choanas;

{190 Palatine,_anterior_processes_forming_the_posterior_border_of_the_choana subequal_in_anterior_extension_or_anterolateral_process_longer anteromedial_process_longer single_process;

{191 Pterygoids,_contact_with_each_other present,_anteriorly_ absent,_remain_separate_along_their_entire_length;

{192 Pterygoid,_anterior_ramus_(=_palatal_process) extends_anterior_to_the_anterior_limit_of_the_palatine forms_oblique_suture_with_palatine_but_process_ends_before_reaching_anterior_limit_of_palatine forms_transverse_suture_with_palatine;

{193 Pterygoid,_anterior_ramus_(=_palatal_process)_shape transversely_broad_at_its_base,_converging_gradually_with_the_transverse_ramus transversely_narrow_along_its_entire_extension,_converging_in_a_right_or_acute_angle_with_the_transverse_ramus_and_the_bone_acquires_an_overall_L-shape_contour_in_ventral_or_dorsal_view;

{194 Pterygoid,_teeth_on_the_ventral_surface_of_the_anterior_ramus_(=_palatal_process),_excluding_tiny_palatal_teeth_if_present present_in_two_distinct_fields_(=_T2_and_T3_of_Welman,_1998) present_in_three_distinct_fields_(=_T2,_T3a_and_T3b) present_in_three_distinct_fields_(=_T2a,_T2b_and_T3) present_in_one_field_that_occupies_most_of_the_transverse_width_of_the_ramus_(=_T2_+_T3) present_in_only_one_posteromedially-to-anterolaterally_oriented_field_(=_T2) present_in_only_one_field_adjacent_to_the_medial_margin_of_the_ramus_(=_T3) absent;

{195 Pterygoid,_number_of_rows_on_palatal_tooth_field_T2 more_than_two_or_do_not_dispose_on_distinct_rows two_rows_parallel_to_each_other single_row;

{196 Pterygoid,_number_of_rows_on_palatal_tooth_field_T3 more_than_two_or_not_disposed_in_distinct_rows two_parallel_rows single_row;

{197 Pterygoid,_most_lateral_row_of_teeth_on_the_ventral_surface_of_the_anterior_ramus_(tooth_field_T2)_raised_on_a_thick,_posteromedially-to-anterolaterally_oriented_ridge absent present;

{198 Pterygoid,_a_row_of_fang-like_teeth_on_the_medial_edge_of_the_anterior_ramus_(=_palatal_process)_(=_T4_of_Welman,_1998) absent present;

{199 Pterygoid,_orientation_of_the_lateral_ramus posterolaterally,_forming_an_obstuse_angle_with_the_anterior_ramus laterally_or_anterolaterally,_forming_a_right_or_acute_angle_with_the_anterior_ramus;

{200 Pterygoid,_lateral_margin_of_the_lateral_ramus_in_dorsal_or_ventral_view posterolateral_margin_with_an_acute_corner posterolateral_margin_merges_smoothly_into_anterolateral_margin_forming_a_smoothly_convex_lateral_outline;

{201 Pterygoid,_teeth_on_the_lateral_ramus present,_more_than_a_single_row_or_no_rows_recognizable present,_single_row_on_the_posterior_edge_(=_T1_of_Welman,_1998) absent;

{202 Ectopterygoid,_body arcs_anteriorly arcs_anterodorsally;

{203 Ectopterygoid,_articulation_with_pterygoid simple_overlap_of_ectopterygoid_and_pterygoid complex_overlap_between_ectopterygoid_and_pterygoid;

{204 Ectopterygoid,_shape_along_suture_with_pterygoid does_not_reach_the_posterolateral_corner_of_the_transverse_flange reaches_the_posterolateral_corner_of_the_transverse_flange;

{205 Ectopterygoid,_contact_with_maxilla absent present;

{206 Ectopterigoid,_posterior_expansion_in_contact_with_jugal absent present;

{207 Supraoccipital,_shape_in_occipital_view plate-like inverted_V-shape;

{208 Supraoccipital,_participation_in_the_dorsal_border_of_the_foramen_magnum absent present;

{209 Supraoccipital,_posterior_surface smooth_or_with_a_low_median_ridge with_a_prominent_median,_vertical_ridge;

{210 Otoccipital,_fusion_between_opisthotic_and_exoccipital absent_or_partial present;

{211 Opisthotic,_contact_between_paroccipital_process_and_parietal_immediately_lateral_to_supraoccipital absent present;

{212 Opisthotic,_paroccipital_processes_orientation extend_laterally_forming_aproximately_a_90°_angle_with_the_parasagittal_plane deflected_posterolaterally_at_an_angle_of_more_than_20°_from_the_transverse_plane_of_the_skull;

{213 Opisthotic,_paroccipital_process_attachment ends_freely contacts_supratemporal_or_proximal_end_of_quadrate_and/or_squamosal sutured_to_the_pterygoid_and_the_pterygoid_wing_of_the_quadrate;

{214 Opisthotic,_paroccipital_process_morphology unflattened_and_tapered_ anteroposteriorly-flattened_distally_;

{215 Opisthotic,_fossa_immediately_lateral_to_the_foramen_magnum absent present;

{216 Opisthotic,_ventral_ramus_shape club-shaped_ pyramidal,_with_a_tapering_distal_end rod-like,_with_a_cylindrical_distal_end_and_relatively_thin rod-like_and_very_robust plate-like;

{217 Opisthotic,_ventral_ramus extends_further_laterally_than_the_lateralmost_edge_of_the_exoccipital_in_posterior_view covered_by_the_lateralmost_edge_of_the_exoccipital_in_posterior_view;

{218 Exoccipital,_morphology_of_the_dorsal_end exoccipital_columnar_throught_dorsoventral_height,_forming_transversely_narrow_dorsal_contact_with_more_dorsal_occipital_elements dorsal_portion_of_exoccipital_exhibits_dorsomedially_inclined_process_that_forms_transversely_broad_contact_with_more_dorsal_occipital_elements;

{219 Exoccipital,_lateral_surface without_subvertical_crest_(=_metotic_strut) with_clear_crest_(=_metotic_strut)_lying_anterior_to_both_external_foramina_for_hypoglossal_nerve_(CN_XII)_ with_clear_crest_(=_metotic_strut)_present_anterior_to_the_more_posterior_external_foramina_for_hypoglossal_nerve_(CN_XII);

{220 Exoccipital,_medial_margin_of_their_distal_ends no_contact_with_its_counterpart contact_with_its_counterpart_to_exclude_basioccipital_from_the_floor_of_the_endocranial_cavity_and_diverge_from_each_other_on_the_occipital_condyle,_exposing_the_basioccipital_dorsally contact_with_its_counterpart_along_the_entire_dorsal_surface_of_the_basioccipital,_excluding_the_basioccipital_from_the_floor_of_the_endocranial_cavity_and_the_dorsal_surface_of_the_occipital_condyle;

{221 Exoccipital,_number_of_foramina_for_the_passage_of_the_hypoglossal_nerve_(CN_XII) two one;

{222 Pseudolagenar_recess,_between_the_ventral_surface_of_the_ventral_ramus_of_the_opisthotic_and_the_basal_tubera present absent;

{223 Lagenar/cochlear_recess absent_or_short_and_strongly_tapered present_and_elongated_and_tubular;

{224 Basioccipital-parasphenoid/parabasisphenoid,_contact_with_each_other_in_mature_individuals loose,_overlapping_suture tightly_sutured,_sometimes_by_an_interdigitated_suture,_or_both_bones_fused_to_each_other;

{225 Basioccipital-parasphenoid/parabasisphenoid,_basal_tubera absent present;

{226 Basioccipital-parasphenoid/parabasisphenoid,_basal_tubera_shape clearly_separated partially_connected medially_expanded_and_nearly_or_completely_connected;

{227 Basioccipital,_position_of_the_posterior_margin_of_the_occipital_condyle even_with_craniomandibular_joint_ anterior_to_craniomandibular_joint posterior_to_craniomandibular_joint;

{228 Basioccipital,_articular_surface_of_the_occipital_condyle concave hemispherical;

{229 Basioccipital,_notochordal_scar_on_the_occipital_surface_of_the_occipital_condyle absent_or_developed_as_a_small_subcircular_pit developed_as_a_vertical_furrow_or_a_large_sub-circular_fossa_that_occupies_approximately_half_of_the_height_of_the_occipital_surface_of_the_condyle;

{230 Basioccipital,_occipital_neck present,_distinctly_separating_the_occipital_condyle_from_the_basioccipital_body absent_or_extremely_short;

{231 Basioccipital,_shape_of_the_basal_tubera rounded_and_anteroposteriorly_elongated bladelike_and_anteroposteriorly_shortened;

{232 Basioccipital,_orientation_of_the_basal_tubera lateroventral,_basal_tubera_divergent_from_each_other ventral,_basal_tubera_parallel_with_each_other;

{233 Parasphenoid-basisphenoid/parabasisphenoid,_exposure_on_the_median_line_of_the_endocranial_cavity_floor present absent;

{234 Parasphenoid/parabasisphenoid,_orientation horizontal_ oblique,_main_axis_posterodorsal-to-anteroventrally_oriented;

{235 Parasphenoid/parabasisphenoid,_posterodorsal_portion incompletely_ossified completely_ossified;

{236 Parasphenoid/parabasisphenoid,_intertuberal_plate absent present_and_straight present_and_arched_anteriorly;

{237 Parasphenoid/parabasisphenoid,_semilunar_depression_on_the_posterolateral_surface_of_the_bone absent present;

{238 Parasphenoid/parabasisphenoid,_recess_(=_median_pharyngeal_recess,_=_hemispherical_sulcus,_=_hemispherical_fontanelle) absent present;

{239 Parasphenoid/parabasisphenoid,_position_of_the_foramina_for_entrance_of_the_cerebral_branches_of_the_internal_carotid_artery_leading_to_the_pituitary_fossa ventral posterolateral anterolateral;

{240 Parasphenoid/parabasisphenoid,_position_of_the_foramina_for_the_entrance_of_the_cerebral_branches_of_the_internal_carotids_on_the_ventral_surface_of_the_bone immediately_medial_or_posteromedial_to_the_base_of_the_basipterygoid_process close_to_the_suture_between_basioccipital_and_parabasisphenoid;

{241 Parasphenoid/parabasisphenoid,_shape_of_the_cultriform_process_in_lateral_view continuously_tapering_anteriorly,_without_dorsoventral_constriction_at_its_base_ dorsoventrally_compressed_at_its_base_;

{242 Parasphenoid/parabasisphenoid,_base_of_the_cultriform_process relatively_dorsoventrally_short tall,_with_the_dorsal_edge_extending_up_between_clinoid_processes_and_ventral_parts_of_the_crista_prootica_;

{243 Parasphenoid/parabasisphenoid,_dentition_on_cultriform_process present absent;

{244 Basisphenoid/parabasisphenoid,_anterior_tympanic_recess_on_the_lateral_side_of_the_braincase absent present;

{245 Basisphenoid/parabasisphenoid,_parasphenoid_crests absent_so_that_there_is_no_ventral_floor_for_the_vidian_canal present_as_a_pair_of_thick_crests_running_along_the_ventrolateral_border_of_the_basisphenoid_body_and_framing_the_ventromedial_floor_of_the_vidian_canal;

{246 Basisphenoid/parabasisphenoid,_basipterygoid_processes moderately_short,_finger-like_and_with_short_articulating_facets long,_with_hemispherical_articulating_facets very_short_and_subcylindrical;

{247 Basisphenoid/parabasisphenoid,_orientation_of_basipterygoid_processes anterolateral_or__lateral posterolateral;

{248 Prootic-supraoccipital,_floccular_(=_auricular)_recess largely_restricted_to_the_prootic extends_onto_internal_surface_of_the_supraoccipital;

{249 Prootic-basisphenoid/parabasisphenoid,_position_of_the_external_foramina_for_passage_of_the_abducens_nerves_(CN_VI) within_the_dorsum_sellae track_between_the_dorsum_sellae_and_prootic,_grooving_the_articular_facets within_the_prootic;

{250 Prootic-basisphenoid/parabasisphenoid,_orientation_of_the_external_foramina_for_passage_of_the_abducens_nerves_(CN_VI) open_anteriorly open_dorsally;

{251 Prootic,_extensive_contact_with_parietal absent present;

{252 Prootic,_contact_with_its_counterpart_on_the_median_line_of_the_floor_of_the_endocranial_cavity absent present;

{253 Prootic,_lateral_surface continuous_and_slightly_convex crista_prootica_present;

{254 Prootic,_anterior_inferior_process absent_or_developed_as_a_small,_peg-like_projection well_developed;

{255 Prootic,_ridge_on_the_lateral_surface_of_the_inferior_anterior_process_ventral_to_the_trigeminal_foramen present absent;

{256 Prootic,_vestibule_on_the_medial_surface incompletely_ossified almost_completely_ossified;

{257 Laterosphenoid,_ossification absent present;

{258 Laterosphenoid,_anterodorsal_channel absent present;

{259 Lower_jaw,_symphysis formed_largely_by_dentary formed_only_by_splenial;

{260 Lower_jaw,_distinct_dorsal_process_behind_the_alveolar_margin absent,_with_a_slightly_convex_dorsal_margin_behind_the_alveolar_portion present,_formed_by_a_dorsally_well-developed_surangular present,_formed_by_a_dorsally_well-developed_posterodorsal_ramus_of_the_dentary_and_sometimes_a_dorsally_well-developed_coronoid_bone;

{261 Lower_jaw,_external_mandibular_fenestra absent present;

{262 Lower_jaw,_anteroposterior_length_of_the_external_mandibular_fenestra_versus_anteroposterior_length_of_the_dentary_anterior_to_the_fenestra 0.07-0.36 0.44-0.53 0.71-0.88;

{263 Lower_jaw,_Meckelian_fossa_orientation dorsomedially mostly_dorsally_due_to_greatly_expanded_prearticular_resulting_in_a_ventral_border_of_the_fossa_situated_dorsal_to_the_half-height_of_the_lower_jaw_at_that_level;

{264 Dentary-splenial,_mandibular_symphysis_length positioned_distally present_along_one-third_of_the_lower_jaw;

{265 Dentary,_minimum_height_of_the_bone_versus_length_of_the_alveolar_margin_(including_edentulous_anterior_end_if_present) 0.05-0.14 0.16-0.19 0.22-0.29 0.34-0.36;

{266 Dentary,_shape_of_the_tooth_bearing_portion mostly_straight distinctly_dorsally_curved_during_all_or_most_of_its_extension ventrally_curved_or_deflected;

{267 Dentary,_large_foramina_aligned_in_two_distinct_rows_starting_on_the_anteroventral_corner_of_the_bone absent present;

{268 Dentary,_longitudinal_groove_approximately_centred_dorsoventrally_on_the_lateral_surface absent present;

{269 Dentary,_position_of_the_Meckelian_groove_on_the_anterior_half_of_the_bone dorsoventral_centre_of_the_dentary restricted_to_the_ventral_border;

{270 Dentary,_anterior_portion unexpanded,_dorsal_margins_of_the_anterior_and_posterior_portions_of_the_bone_in_the_same_plane dorsally_expanded,_whole_dorsoventral_height_of_the_anterior_portion_is_greater_than_that_of_the_posterior_portion;

{271 Dentary,_posterodorsal_process,_in_which_its_dorsal_margin_is_confluent_with_the_dorsal_margin_of_the_lower_jaw absent present;

{272 Dentary,_posterocentral_process,_in_which_its_margins_are_not_confluent_with_the_dorsal_or_ventral_margin_of_the_lower_jaw absent present;

{273 Dentary,_distal_end_of_the_posterocentral_process_(process_that_contributes_to_the_anterodorsal_border_of_the_external_mandibular_fenestra) tapering rounded;

{274 Dentary,_posteroventral_process,_in_which_its_ventral_margin_is_confluent_with_the_ventral_margin_of_the_lower_jaw absent present_and_excluded_from_the_anteroventral_border_of_the_external_mandibular_fenestra present_and_contributing_to_the_anteroventral_border_of_the_external_mandibular_fenestra_;

{275 Dentary,_posteroventral_process_length extended_posteriorly_to_the_level_of_the_posterodorsal_and/or_posterocentral_processes_ extended_posteriorly_beyond_the_level_of_the_posterodorsal_and/or_posterocentral_processes;

{276 Posteriormost_dentary_teeth on_the_anterior_half_of_lower_jaw on_the_posterior_half_of_lower_jaw;

{277 Dentary,_alveolar_margin present_along_entire_length_of_the_dentary absent_in_the_anterior_portion;

{278 Dentary,_number_of_tooth_rows one_ two more_than_two_;

{279 Dentary,_occlusion_with_upper_teeth single-sided_overlap flat_occlusion blade_and_groove;

{280 Surangular-angular,_suture even_with_lateral_surface_of_hemimandible elevated_and_separates_dorsal_concave_area_on_surangular_from_concave_area_on_angular;

{281 Surangular-angular,_suture_along_the_anterior_half_of_the_bones_in_lateral_view anteroposteriorly_convex_ventrally anteroposteriorly_concave_ventrally;

{282 Surangular/articular,_retroarticular_process absent anteroposteriorly_short,_being_poorly_developed_posteriorly_to_the_glenoid_fossa anteroposteriorly_long,_extending_considerably_posteriorly_to_the_glenoid_fossa;

{283 Surangular-articular,_retroarticular_process not_upturned upturned;

{284 Surangular,_anterior_extension beyond_coronoid_eminence posterior_to_reaching_the_anterior_border_of_the_coronoid_eminence;

{285 Surangular,_lateral_shelf absent present,_low_ridge_near_dorsal_margin present,_presence_of_laterally_or_ventrolaterally_projecting_shelf_with_straight_or_gently_convex_lateral_edge present,_presence_of_laterally_projecting_shelf_with_strongly_convex_lateral_edge;

{286 Surangular,_dorsal_margin_in_lateral_view straight_or_gently_convex strongly_convex;

{287 Surangular,_anterior_surangular_foramen_on_the_lateral_surface_of_the_bone,_near_surangular-dentary_contact absent present;

{288 Surangular,_posterior_surangular_foramen_on_the_lateral_surface_of_the_bone,_positioned_directly_anterolateral_to_the_glenoid_fossa absent present;

{289 Angular,_dorsoventral_exposure_on_the_lateral_surface_of_the_lower_jaw wide narrow;

{290 Angular,_ventrolateral_surface continuous_with_lateral_surface_of_angular laterally_projecting_ridge_present_that_separates_lateral_and_ventral_sides_of_the_angular;

{291 Angular,_posteroventral_surface ridged_or_keeled transversely_convex;

{292 Articular,_fused_to_the_prearticular absent present;

{293 Articular,_foramen_on_the_medial_side absent present;

{294 Articular,_ventromedially_directed_process absent present;

{295 Stapes,_shape robust,_with_thick_shaft slender,_rod-like_shaft;

{296 Stapes,_stapedial_foramen_piercing_the_columellar_process present absent;

{297 Teeth,_posterior_extent_of_mandibular_and_maxillary_tooth_rows subequal maxillary_teeth_extending_further_posteriorly;

{298 Teeth,_tooth_attachment subthecodont_(=_protothecodont) ankylothecodont pleurodont acrodont thecodont;

{299 Teeth,_maxillary_and/or_dentary_tooth_crowns generally_homodont markedly_heterodont_(gross_change_in_morphology)_;

{300 Teeth,_enlarged_caniniform_region_in_maxilla present absent;

{301 Teeth,_maxillary_tooth_crowns_in_labial_view all_the_tooth_crowns_possess_a_rather_similar_distal_edge_morphology_along_the_entire_alveolar_margin the_distal_edge_of_the_posterior_tooth_crowns_possess_a_distinct_different_morphology_from_those_of_the_anterior_tooth_crowns,_usually_the_posterior_edge_becomes_convex;

{302 Teeth,_distal_edge_of_the_maxillary_tooth_crowns_in_labial_view concave_in_all_tooth_crowns straight_or_gently_sigmoid convex_in_at_least_some_anterior_tooth_crowns;

{303 Teeth,_serrations_on_the_maxillary/dentary_crowns absent distinctly_present_on_the_distal_margin_and_usually_apically_restricted,_low_or_absent_on_the_mesial_margin present_and_distinct_on_both_margins_;

{304 Teeth,_labiolingual_compression_of_the_marginal_dentition only_distally_or_nowhere_ present;

{305 Teeth,_multiple_maxillary_or_dentary_tooth_crowns_with_longitudinal_labial_or_lingual_striations_or_grooves absent present;

{306 Teeth,_multiple_maxillary_and_dentary_tooth_crowns_with_extensive_wear_facets absent present;

{307 Teeth,_multiple_maxillary_and_dentary_tooth_crowns_distinctly_mesiodistally_expanded_above_the_root absent present;

{308 Hyoid_apparatus,_length_and_orientation_of_the_ceratobranchial short,_directed_to_quadrate_region long,_directed_posteriorly_and_extending_posteriorly_beyond_the_quadrate_condyles;

{309 Cervical,_dorsal,_sacral_and_caudal_vertebrae,_notochordal_canal_piercing_completely_the_centrum present_throughout_ontogeny absent_in_adults;

{310 Cervical_and_dorsal_vertebrae,_anteroposterior_compression_of_centra_in_the_cervico-dorsal_transition_(=_pectoral_centra) moderate very_strong,_being_considerably_anteroposteriorly_shorter_than_tall;

{311 Cervical_and_dorsal_vertebrae,_neurocentral_sutures close_in_adults remain_open_in_sub-adults_and_adults;

{312 Cervical_and_dorsal_vertebrae,_at_least_one_or_more_cervical_or_anterior_dorsal_with_parallelogram-shaped_centra_in_lateral_view,_in_which_the_anterior_articular_surface_is_situated_higher_than_the_posterior_one absent present;

{313 Cervical_and_dorsal_vertebrae,_one_or_more_vertebrae_with_an_accessory_rib_articular_facet_between_the_diapophysis_and_parapophysis_in_the_cervico-dorsal_transition absent present;

{314 Cervical_and_dorsal_vertebrae,_anterior_centrodiapophyseal_lamina_or_paradiapophyseal_lamina_in_posterior_cervicals_and_anterior_dorsals absent present;

{315 Cervical_and_dorsal_vertebrae,_posterior_centrodiapophyseal_lamina_in_cervicals_and_anterior_dorsals absent present;

{316 Cervical_and_dorsal_vertebrae,_prezygodiapophyseal_lamina_in_posterior_cervicals_and_anterior_dorsals absent present;

{317 Cervical_and_dorsal_vertebrae,_postzygodiapophyseal_lamina_in_posterior_cervicals_and_anterior_dorsals absent present;

{318 Cervical_and_dorsal_vertebrae,_thick,_mainly_vertical_tuberosity_immediately_below_the_transverse_process,_but_both_structures_are_not_connected_with_each_other,_in_posterior_cervicals_and_anterior_dorsals absent present;

{319 Cervical_and_dorsal_vertebrae,_gradual_transverse_expansion_of_the_distal_half_of_the_neural_spine absent present,_but_lacking_distinct_mammillary_processes_on_the_lateral_surface_of_the_neural_spine present,_with_distinct_mammillary_processes_on_the_lateral_surface_of_the_neural_spine;

{320 Cervical_and_dorsal_vertebrae,_spine_table_in_the_distal_end_of_the_postaxial_neural_spines_(not_mammillary_process) absent_ present_in_cervicals,_but_not_in_dorsals present_in_dorsal,_but_not_in_cervicals present_in_both_cervicals_and_dorsals;

{321 Cervical_and_dorsal_vertebrae,_distal_surface_of_transverse_expansion_of_the_neural_spine convex_ flat_;

{322 Cervical_and_dorsal_vertebrae,_outline_of_the_spine_tables_in_dorsal_view suboval_or_subrectangular subtriangular_or_heart-shaped;

{323 Cervical_vertebrae,_number_of_vertebrae_in_the_neck fewer_than_eight eight_or_nine more_than_ten;

{324 Cervical_vertebrae,_atlantal_articulation_facet_on_the_axial_intercentrum saddle-shaped concave_with_upturned_lateral_borders;

{325 Cervical_vertebrae,_centrum_of_atlas_in_mature_individuals separate_from_axial_intercentrum fused_to_axial_intercentrum;

{326 Cervical_vertebrae,_ventral_surface_of_the_centrum_on_anterior_cervicals transversely_convex with_a_low_median_longitudinal_keel with_a_median_longitudinal_keel_that_extends_ventral_to_the_centrum_rims_in_at_least_one_anterior_cervical_;

{327 Cervical_vertebrae,_height_of_the_neural_spine_of_the_axis dorsoventrally_tall strongly_dorsoventrally_low;

{328 Cervical_vertebrae,_shape_of_the_neural_spine_of_the_axis expanded_posterodorsally_or_the_height_of_the_anterior_portion_is_equivalent_to_the_posterior_height expanded_anterodorsally;

{329 Cervical_vertebrae,_dorsal_margin_of_the_neural_spine_of_the_axis dorsally_convex mostly_straight_or_dorsally_concave;

{330 Cervical_vertebrae,_lengths_of_the_fourth_and_fifth_cervical_centra_versus_the_height_of_their_anterior_articular_surface 0.63-2.67 2.92-4.12 6.09-6.80 14.16-14.33;

{331 Cervical_vertebrae,_diapophysis_and_parapophysis_of_anterior_to_middle_cervical_postaxial_vertebrae single_facet_or_both_situated_on_the_same_process situated_on_different_processes_and_well_separated situated_on_different_processes_and_nearly_touching;

{332 Cervical_vertebrae,_position_of_diapophysis_or_dorsal_margin_of_synapophysis_in_anterior_postaxial_cervicals at_or_near_dorsoventral_level_of_pedicles near_the_dorsoventral_mid-point_of_the_centrum;

{333 Cervical_vertebrae,_longitudinal_lamina_or_tuberosity_extended_posteriorly_from_the_base_of_the_transverse_process_in_postaxial_anterior_and_middle_cervicals absent_or_poorly_developed,_not_well_laterally_developed strongly_developed,_flaring_laterally_as_a_prominent_and_thick,_wing-like_shelf;

{334 Cervical_vertebrae,_posterior_portion_of_the_neural_arch_ventral_to_the_postzygapophysis_in_postaxial_cervicals smooth with_a_shallow,_posterolaterally_facing_fossa;

{335 Cervical_vertebrae,_epipophysis_in_postaxial_cervicals absent present_in_at_least_the_third_to_fifth_cervical_vertebrae;

{336 Cervical_vertebrae,_excavation_immediately_lateral_to_the_base_of_postaxial_cervical_neural_spines absent_ shallow_ represented_by_a_deep_pocket_or_pit;

{337 Cervical_vertebrae,_anterior_cervical_vertebrae_(presacral_vertebrae_3?5)_postzygapophyses separated_posteriorly connected_through_a_horizontal_lamina_(=_transpostzygapophyseal_lamina)_with_a_notch_at_the_midline;

{338 Cervical_vertebrae,_shape_of_the_postaxial_neural_spines_in_lateral_view sub-triangular rectangular;

{339 Cervical_vertebrae,_distinct_longitudinal_lamina_extending_along_the_lateral_surface_of_the_centrum_at_mid-height_in_postaxial_anterior_and_middle_cervical_vertebrae absent present;

{340 Cervical_vertebrae,_longitudinal_lamina_connecting_the_prezygapophysis_and_postzygapophysis_in_the_third_cervical_neural_arch absent present;

{341 Cervical_vertebrae,_shape_of_postaxial_anterior_cervical_neural_spines tall,_with_height_and_length_approximately_equal_or_height_larger long_and_low,_with_height_lower_than_length;

{342 Cervical_vertebrae,_anterior_and_middle_postaxial_cervical_neural_spines_with_an_anterior_overhang absent present;

{343 Cervical_vertebrae,_relative_location_of_dorsal_margin_of_mid-cervical_neural_spines spines_are_equivalent_in_height_and_length_to_other_cervical_neural_spines_ spines_are_dorsoventrally_depressed_at_their_anteroposterior_midpoints,_leaving_them_little_more_than_midline_dorsal_ridges;

{344 Cervical_vertebrae,_position_of_the_mammillary_processes_of_the_neural_spines_along_the_neck present_from_the_fourth_presacral present_from_the_fifth_presacral present_from_the_sixth_or_seventh_presacral present_from_the_eighth_or_ninth_presacral;

{345 Cervical_vertebrae,_postaxial_cervical_intercentra present absent;

{346 Cervical_and_dorsal_ribs,_tuberculum_in_posterior_cervical_or_anterior_dorsal_ribs short long_and_distinct;

{347 Cervical_and_dorsal_ribs,_at_least_one_rib_of_the_cervico-dorsal_transition_with_a_thin_lamina_webbing_tuberculum_and_capitulum absent present;

{348 Cervical_ribs,_shape short,_being_less_than_two_times_the_length_of_its_respective_vertebra,_and_tapering_at_a_high_angle_to_the_neck_ short,_being_less_than_two_times_the_length_of_its_respective_vertebra,_and_shaft_parallel_to_the_neck_ very_long,_being_two_times_the_length_of_its_respective_vertebra,_and_parallel_to_the_neck;

{349 Cervical_ribs,_accessory_process_on_anterolateral_surface_of_anterior_cervical_ribs absent present;

{350 Dorsal_vertebrae,_length_versus_height_of_the_centrum_in_anterior_dorsals 0.45-1.10 1.18-2.00 2.19-2.74;

{351 Dorsal_vertebrae,_length_versus_height_of_the_centrum_in_posterior_dorsals 0.66-1.39 1.48-1.86 1.95-2.04 2.39-2.46;

{352 Dorsal_vertebrae,_ventral_surface_of_middle_and_posterior_centra transversely_convex ridged,_with_slightly_swollen_sides single_keel double_keel;

{353 Dorsal_vertebrae,_lateral_fossa_on_the_centrum_below_the_neurocentral_suture absent_ present,_but_not_well-rimed present_and_well-rimed;

{354 Dorsal_vertebrae,_subcentral_foramen_in_the_lateral_surface_of_the_centra absent present;

{355 Dorsal_vertebrae,_diapophysis_and_parapophysis_in_anterior_dorsals close_to_the_body_of_the_midline expanded_on_stalks;

{356 Dorsal_vertebrae,_ratio_between_transverse_width_of_diapophysis_and_length_of_the_centrum_in_anterior_dorsals <0.70 >0.75;

{357 Dorsal_vertebrae,_development_of_the_transverse_processes_in_middle_and_posterior_dorsals short moderately_long extremely_long,_being_considerably_broader_than_its_respective_centrum;

{358 Dorsal_vertebrae,_hyposphene-hypantrum_accessory_intervertebral_articulation_in_middle-posterior_dorsals absent present;

{359 Dorsal_vertebrae,_zygosphene-zygantrum_articulation absent present;

{360 Dorsal_vertebrae,_dorsally_opened_pit_lateral_to_the_base_of_the_neural_spine absent shallow_ developed_as_a_deep_pit_;

{361 Dorsal_vertebrae,_anterior_and_middle_dorsal_neural_spines subrectangular,_with_the_anterior_margin_vertical,_anterodorsally_or_slightly_posterodorsally_inclined subtriangular,_with_the_anterior_margin_strongly_posterodorsally_oriented;

{362 Dorsal_vertebrae,_fan-shaped_neural_spine_in_lateral_view absent present;

{363 Dorsal_vertebrae,_position_of_middle_dorsal_neural_spines situated_at_mid-length_between_the_zygapophyses displaced_posteriorly_from_mid-length_between_the_zygapophyses;

{364 Dorsal_vertebrae,_position_of_the_mammillary_processes_of_the_neural_spines_in_the_trunk extend_up_to_the_tenth_presacral extend_up_to_the_eleventh_presacral extend_up_to_the_twelfth_presacral extend_up_to_the_thirteenth_presacral extend_up_to_the_sixteenth_presacral_or_beyond;

{365 Dorsal_vertebrae,_intercentra present absent;

{366 Dorsal_ribs,_angle_between_heads_and_shaft_in_anterior_dorsal_ribs close_to_90º low,_gentle_posteroventral_bowing_of_the_base_of_the_shaft;

{367 Dorsal_ribs,_proximal_end_of_middle_dorsal_ribs dichocephalous holocephalous;

{368 Sacral_vertebrae-sacral_ribs,_ratio_between_the_width_of_the_neural_arch_+_ribs_of_the_first_primordial_sacral_and_the_length_of_the_neural_arch_across_the_zygapophyses less_than_three_times three_times_or_more;

{369 Sacral_vertebrae,_number two three four_or_more;

{370 Sacral_ribs almost_entirely_restricted_to_a_single_sacral_vertebra shared_between_two_sacral_vertebrae;

{371 Sacral_ribs,_anteroposterior_length_of_the_first_primordial_sacral_rib_versus_the_second_primordial_sacral_rib_in_dorsal_view primordial_sacral_rib_one_is_longer_anteroposteriorly_than_primordial_sacral_rib_two primordial_sacral_rib_two_is_about_the_same_length_or_longer_anteroposteriorly_than_primordial_sacral_rib_one;

{372 Sacral_ribs,_second_rib_shape single_unit bifurcates_distally_into_anterior_and_posterior_processes;

{373 Sacral_ribs,_morphology_of_posterior_process pointed_bluntly truncated_sharply;

{374 Sacral_and_caudal_vertebrae,_transverse_processes_and_ribs_of_sacral_and/or_anterior_caudal_vertebrae_in_mature_individuals sutured_to_the_vertebra fused_to_the_vertebra;

{375 Caudal_vertebrae,_autotomic_septa_within_the_centrum absent present;

{376 Caudal_vertebrae,_length_of_the_transverse_process_+_rib_versus_length_across_zygapophyses_in_anterior_caudal_vertebrae 0.29-0.41 0.62-1.20 1.51-1.68 2.20-2.72;

{377 Caudal_vertebrae,_distal_end_of_the_transverse_processes_+_ribs_of_anterior_caudals_in_dorsal_or_ventral_view tapering_or_squared anteroposteriorly_expanded;

{378 Caudal_vertebrae,_neural_spine_height_versus_anteroposterior_length_at_its_base_in_anterior_caudals 0.66-2.21 2.36-2.65 2.92-3.05 3.42-3.54;

{379 Caudal_vertebrae,_accessory_laminar_process_on_the_anterior_face_of_the_neural_spine_on_middle_caudals absent present;

{380 Caudal_vertebrae,_prezygapophysis_of_posterior_caudals not_elongated_ elongated_more_than_a_quarter_of_the_adjacent_centrum;

{381 Chevrons,_distal_anteroposterior_width_of_anterior_and_middle_haemal_spines_in_lateral_view equivalent_to_proximal_length tapering_distally_ longer_than_proximal_width_(=_paddle-like_haemal_spine);

{382 Gastralia present,_forming_an_extensive_ventral_basket_with_closely_packed_elements present,_well_separated absent;

{383 Scapulacoracoid,_both_bones_fused_with_each_other_in_mature_individuals present absent;

{384 Scapulacoracoid,_notch_on_the_anterior_margin_at_level_of_the_suture_between_both_bones absent present;

{385 Scapulacoracoid,_glenoid_fossa_orientation posterolateral_ posteroventral_;

{386 Scapula,_total_length_of_the_scapula_versus_minimum_anteroposterior_width_of_the_scapular_blade 1.23-6.73 7.92-11.31;

{387 Scapula,_large_fenestra_between_scapula_and_coracoid_immediately_anterior_to_the_glenoid_region absent present;

{388 Scapula,_strong_curvature_or_inflexion_of_the_posterior_margin_of_the_scapular_blade absent present,_the_angle_formed_is_close_to_90º;

{389 Scapula,_anterior_margin_of_the_scapular_blade_in_lateral_view straight_or_convex_along_entire_length distinctly_concave;

{390 Scapula,_constriction_distal_to_the_glenoid anteroposterior_length_greater_than_half_the_proximodistal_length_of_the_scapula anteroposterior_length_less_than_half_the_proximodistal_length_of_the_scapula;

{391 Scapula,_supraglenoid_foramen absent present;

{392 Scapula,_lateral_tuber_on_the_posterior_edge,_just_dorsal_of_the_glenoid_fossa absent present;

{393 Scapula,_diagonal_ridge_adjacent_to_the_anterior_margin_on_the_medial_surface_of_the_scapular_blade abent present;

{394 Scapula,_acromion_process in_about_the_same_plane_as_the_ventral_edge_of_the_scapula distinctly_raised_above_the_ventral_edge_of_the_scapula;

{395 Scapula,_acromion_process gently_raised_from_the_anterior_margin_of_the_scapular_blade sharply_raised_in_an_angle_close_to_90º_from_the_anterior_margin_of_the_scapular_blade;

{396 Coracoid,_anterior_border_in_lateral_view rounded distinctly_hooked;

{397 Coracoid,_posterior_border_in_lateral_view unexpanded_posteriorly moderately_expanded_posteriorly strongly_expanded_posteriorly_-_the_entire_border,_not_only_the_posteroventral_region_as_is_the_case_in_the_postglenoid_process_-_and,_as_a_result,_the_scapular_girdle_acquires_an_L-shape_in_lateral_view;

{398 Coracoid,_subglenoid_lip_posteror_extension as_developed_as_or_less_developed_than_the_supraglenoid_lip_on_the_scapula more_extended_than_the_supraglenoid_lip_on_the_scapula;

{399 Coracoid,_subglenoid_lip_lateral_extension poorly_developed,_resembling_the_development_of_the_supraglenoid_lip_on_the_scapula strongly_developed_as_a_shelf-like_structure,_more_developed_than_the_supraglenoid_lip_on_the_scapula;

{400 Coracoid,_biceps_process absent_or_small large;

{401 Coracoid,_postglenoid_process_separated_from_the_glenoid_fossa_by_a_notch absent present;

{402 Coracoid,_postglenoid_process_shape_in_lateral_view rounded_posterior_margin tapering_posterior_margin;

{403 Cleithrum present absent;

{404 Interclavicle present absent;

{405 Interclavicle,_anterior_process present absent;

{406 Interclavicle,_anterior_margin_with_a_median_notch absent present;

{407 Interclavicle,_lateral_processes well_developed reduced_or_absent;

{408 Interclavicle,_webbed_between_lateral_and_posterior_processes present,_proximal_half_of_the_bone_subtriangular_or_diamond-shaped absent,_rather_sharp_angles_between_processes;

{409 Interclavicle,_transverse_width_at_mid-length_of_the_posterior_process_versus_the_length_of_the_posterior_process 0.07-0.14 0.20-0.27;

{410 Interclavicle,_posterior_ramus little_change_in_width_along_entire_length gradual_transverse_expansion_present;

{411 Clavicle,_articulation_with_interclavicle on_the_anteroventral_surface_of_the_interclavicle on_the_anterodorsal_surface_of_the_interclavicle_ into_a_deep,_anteriorly_facing_socket;

{412 Sternum not_mineralized mineralized_(bone_or_calcified_cartilage);

{413 Forelimb-hindlimb,_length_ratio >0.55 <0.55;

{414 Humerus,_torsion_between_proximal_and_distal_ends approximately_45°_or_more_ 35°_or_less_;

{415 Humerus,_transverse_width_of_the_proximal_end_versus_total_length_of_the_bone_in_mature_individuals 0.20-0.41 0.44-0.70;

{416 Humerus,_proximal_articular_surface_in_proximal_view subrectangular_to_crescent-shape sub-oval;

{417 Humerus,_proximal_articular_surface continuous_with_the_deltopectoral_crest separated_by_a_gap_from_the_deltopectoral_crest;

{418 Humerus,_proximal_end_in_anterior_view approximately_symmetric medially_expanded,_being_asymmetric;

{419 Humerus,_conical_process_on_the_proximal_surface,_placed_immediately_adjacent_to_the_base_of_the_deltopectoral_crest absent present;

{420 Humerus,_internal_tuberosity_distinctly_separated_proximally_from_the_articular_surface absent present;

{421 Humerus,_shape_of_the_deltopectoral_crest_in_lateral_view rounded_or_subtriangular_ subrectangular_or_trapezoidal_;

{422 Humerus,_ventral_margin_of_the_deltopectoral_crest_developed_as_a_thick_subcilindrical_tuberosity_that_is_well_differentiated_from_the_thinner_dorsal_margin present absent;

{423 Humerus,_length_of_the_deltopectoral_crest_relative_to_total_length_of_the_bone_in_mature_individuals 0.16-0.18 0.24-0.49 0.52-0.55;

{424 Humerus,_entepicondyle_size_in_mature_individuals moderately_large strongly_developed;

{425 Humerus,_entepicondylar_foramen present absent;

{426 Humerus,_ectepicondylar_region foramen_present foramen_absent,_supinator_process_and_groove_present supinator_process,_groove_or_foramen_absent;

{427 Humerus,_capitellum_(radial_condyle)_and_trochlea_(ulnar_condyle) ball-shaped_structures_distinct_from_the_ectepicondyle_and_entepicondyle absent_or_incipient;

{428 Humerus,_trochlea_(ulnar_condyle)_situated_approximately_at_mid-width_on_the_distal_end_of_the_bone present absent,_being_considerably_laterally_displaced_;

{429 Ulna,_olecranon_process absent_or_low prominent_but_lower_than_its_anteroposterior_depth_at_base strongly_developed,_being_higher_than_its_anteroposterior_depth_at_base;

{430 Ulna,_olecranon_process_in_lateral_view tapering_towards_the_proximal_tip_of_the_bone subrectangular_or_slightly_expanded_towards_the_proximal_tip_of_the_bone;

{431 Ulna,_olecranon_process_as_a_separate_ossification absent present;

{432 Ulna,_lateral_tuber_(=_radius_tuber)_on_the_proximal_portion absent present;

{433 Ulna,_distal_end_in_posterolateral_view rounded_and_convex_ squared_off_where_the_distal_surface_is_nearly_flat;

{434 Radius,_total_length_versus_total_length_of_the_humerus 0.62-0.66 0.69-0.92 0.95-0.97 1.12-1.17;

{435 Radius,_length_in_comparisons_with_that_of_the_ulna shorter_ longer_or_subequal;

{436 Radius,_shape straight twisted_in_lateral_view;

{437 Radius,_distal_end unexpanded_or_poorly_anteroposteriorly_expanded strongly_anteroposteriorly_expanded;

{438 Carpals,_intermedium present absent;

{439 Carpals,_perforating_foramen_between_intermedium_and_ulnare present absent;

{440 Carpals,_medial_centrale present absent;

{441 Carpals,_lateral_centrale large small_or_absent;

{442 Carpals,_pisiform present absent;

{443 Carpals,_distal_carpal_five absent present;

{444 Manus,_longest_metacarpal_+_digit longer_than_humeral_length_ subequal_to_shorter_than_humeral_length_;

{445 Metacarpus,_length_of_the_longest_metacarpal_versus_length_of_the_longest_metatarsal 0.34-0.39 0.43-0.45 0.54-0.98;

{446 Metacarpus,_proximal_ends overlap_ abut_one_another_without_overlapping;

{447 Metacarpus,_width_of_the_distal_end_of_the_metacarpal_I_versus_its_total_length 0.26-0.33 0.36-0.45 0.48-0.53 0.58-0.64 0.73-0.75;

{448 Metacarpus,_extensor_pits_on_the_dorsodistal_portion_of_the_metacarpals_I-III absent_or_shallow_and_symmetrical deep_and_asymmetrical;

{449 Metacarpus,_metacarpal_IV longer_than_metacarpal_III equal_or_shorter_than_metacarpal_III;

{450 Manual_digits,_unguals_length about_the_same_length_or_shorter_than_the_last_phalanx_of_the_same_digit distinctly_longer_than_the_last_phalanx_of_the_same_digit;

{451 Manual_digits,_unguals_of_manual_digits_I-III blunt_on_at_least_digits_II_and_III trenchant_on_digits_I-III;

{452 Manual_digits,_second_phalanx_of_manual_digit_II shorter_than_the_first_phalanx_of_manual_digit_II_ longer_than_the_first_phalanx_of_manual_digit_II;

{453 Manual_digits,_number_of_phalanges_in_digit_IV five four three_or_less;

{454 Pelvic_girdle,_acetabulum completely_closed perforated;

{455 Pelvic_girdle,_acetabulum_orientation mainly_laterally_facing lateroventrally_or_mainly_ventrally_facing;

{456 Pelvic_girdle,_acetabular_antitrochanter absent present;

{457 Ilium,_maximum_height_of_the_acetabulum_versus_length_of_the_femur 0.12-0.17 0.21-0.47 0.54-0.57;

{458 Ilium,_laterally_deflected_dorsal_blade absent present;

{459 Ilium,_preacetabular_process absent_or_incipient present,_being_considerably_anteroposteriorly_shorter_than_its_dorsoventral_height present,_being_longer_than_two_thirds_of_its_height_and_not_extending_beyond_the_level_of_the_anterior_margin_of_the_pubic_peduncle present_and_extending_beyond_the_level_of_the_anterior_margin_of_the_pubic_peduncle;

{460 Ilium,_preacetabular_process semicircular_ subtriangular_or_finger-like;

{461 Ilium,_lateral_crest_dorsal_to_the_supraacetabular_crest/rim absent present_and_divides_the_preacetabular_process_from_the_postacetabular_process confluent_with_the_anterior_extent_of_the_preacetabular_process;

{462 Ilium,_length_of_the_postacetabular_process_versus_anteroposterior_length_of_the_acetabulum 0.31-0.63 0.79-1.24 1.31-1.37 1.49-1.55;

{463 Ilium,_main_axis_of_the_postacetabular_process_in_lateral_or_medial_view posterodorsally_oriented mainly_posteriorly_oriented;

{464 Ilium,_caudifemoralis_brevis_muscle_origin_on_the_lateroventral_surface_of_the_postacetabular_process not_dorsally_or_laterally_rimed_by_a_brevis_shelf dorsally_rimed_by_a_brevis_shelf,_but_lacking_a_brevis_fossa dorsolaterally_rimed_by_a_brevis_shelf_and_with_a_lateroventrally_facing_brevis_fossa laterally_rimed_by_a_brevis_shelf_and_with_a_ventrally_facing_brevis_fossa;

{465 Ilium,_dorsal_margin_of_the_iliac_blade convex mostly_straight concave;

{466 Ilium,_angle_between_anterior_margin_of_the_pubic_peduncle_and_longitudinal_axis_across_pubic_and_ischiadic_peduncles lower_than_45º equal_or_higher_than_45º_;

{467 Ilium,_posteriorly_projected_heel_on_the_posterior_margin_of_the_ischiadic_peduncle_in_lateral_view absent present,_with_its_dorsal_margin_settled_at_45º_or_lower_to_the_longitudinal_axis_of_the_bone;

{468 Ilium,_acetabulum_shape irregular,_marked_by_posterodorsal_invasion_by_finished_bone_ roughly_circular,_no_posterodorsal_invasion_by_finished_bone;

{469 Pubis-ischium,_contact present_and_extended_ventrally present_and_reduced_to_a_thin_proximal_contact;

{470 Pubis-ischium,_thyroid_fenestra: absent present;

{471 Pubis,_total_length_versus_anteroposterior_length_of_the_acetabulum 1.15-2.58 2.84-3.43 3.94-4.87;

{472 Pubis,_anterior_and_posterior_portions_of_the_acetabular_margin continuous_ recessed_;

{473 Pubis,_tuberosity_for_the_attachment_of_the_ambiens_muscle_in_mature_individuals prominent incipient_or_absent;

{474 Pubis,_shaft_orientation anteroventral vertical_or_posteroventral_;

{475 Pubis,_form_of_the_shaft_(=_pubic_tubercle,_=_pectineal_tuberosity) plate-like rod-like_and_curved_posteriorly rod-like_and_straight;

{476 Pubis,_anterior_apron absent,_symphysis_extended_along_the_ventral_margin_of_the_pelvic_girdle_and_visible_in_lateral_view present,_symphysis_restricted_anteriorly_and_obscured_by_the_pubic_shaft_in_lateral_view;

{477 Pubis,_transverse_width_of_conjoined_aprons_versus_total_length_of_the_bone 0.27-0.59 0.77-0.97 1.12-1.28 1.48-1.94;

{478 Pubis,_pectineal_process absent present;

{479 Pubis,_distal_end_in_lateral_or_medial_view unexpanded_or_gently_expanded_anteroposteriorly sharply_expanded_anteroposteriorly,_forming_a_distinct_pubic_boot;

{480 Pubis,_transverse_width_of_the_distal_portion nearly_as_broad_as_the_proximal_width significantly_narrower_than_the_proximal_width;

{481 Ischium,_total_length_versus_anteroposterior_length_of_the_acetabulum 1.04-1.24 1.55-2.50 2.72-3.53 4.31-4.48;

{482 Ischium,_proximal_articular_surface articular_surface_with_the_ilium_and_pubis_continuous articular_surfaces_with_the_ilium_and_pubis_continuous_but_separated_by_a_fossa articular_surfaces_with_the_ilium_and_pubis_separated_by_a_nonarticulating_concave_surface;

{483 Ischium,_longitudinal_groove_on_the_dorsal_surface_of_shaft absent present;

{484 Ischium,_medial_contact_with_antimere restricted_to_the_medial_edge extensive_contact_but_the_dorsal_margins_are_separated;

{485 Ischium,_symphysis_raised_on_a_distinct_low_peduncle absent present;

{486 Ischium,_cross-section_of_the_distal_portion platelike semicircular_or_subtriangular;

{487 Ischium,_shape_of_posterior_margin linear_posterior_margin posterior_process_extends_from_posterodorsal_ischiadic_margin;

{488 Femur,_total_length_versus_total_length_of_the_humerus 0.92-0.97 1.09-1.56 1.62-1.74 1.86-1.96;

{489 Femur,_minimum_transverse_width_versus_minimum_transverse_width_of_the_humerus 0.95-1.01 1.08-1.32 1.46-1.80 1.93-2.00;

{490 Femur,_proximal_articular_surface well_ossified,_being_flat_or_convex partially_ossified,_being_concave_and_sometimes_with_a_circular_pit;

{491 Femur,_femoral_head not_distinctly_offset_from_the_shaft distinctly_offset_from_the_shaft;

{492 Femur,_femoral_head_orientation_(long_axis_of_the_femoral_head_angle_with_respect_to_the_transverse_axis_through_the_femoral_condyles_Parrish,_1986) anterior_(60º-90º) anteromedial_(20º-60º);

{493 Femur,_proximal_articular_surface_(=_posterolateral_portion_of_the_head_sensu_Nesbitt_2011) limited_to_the_proximal_surface_of_the_bone extends_under_the_proximal_surface_of_the_bone;

{494 Femur,_proximal_surface rounded_and_smooth_ transverse_groove_present;

{495 Femur,_posteromedial_tuber_(=_anteromedial_tuber_of_Nesbitt,_2011)_on_the_femoral_head absent present;

{496 Femur,_posterior_tuber_on_the_femoral_head present absent;

{497 Femur,_anterior_tuber_(=_anterolateral_tuber_of_Nesbitt,_2011)_on_the_femoral_head present_as_an_expansion absent_;

{498 Femur,_fossa_trochanterica_(sensu_Novas,_1996)_on_the_ventral/posterior_surface_of_the_proximal_end present absent;

{499 Femur,_dorsolateral_trochanter_on_the_anterolateral_surface_of_the_proximal_end absent present;

{500 Femur,_transition_between_femoral_head_and_shaft smooth_ notch_ concave_emargination;

{501 Femur,_anterior_trochanter_(=_lesser_or_minor_trochanter)_(=_iliofemoralis_cranialis_muscle_insertion) absent present;

{502 Femur,_trochanteric_shelf absent present_in_mature_individuals;

{503 Femur,_attachment_of_muscle_caudifemoralis_on_the_posterior_surface_of_the_bone crest-like_and_with_intertrochanteric_fossa_(=_internal_trochanter),_and_convergent_with_proximal_end crest-like_and_with_intertrochanteric_fossa_(=_internal_trochanter),_and_not_convergent_with_proximal_end crest-like_and_without_intertrochanteric_fossa_(=_fourth_trochanter),_and_not_convergent_with_proximal_end;

{504 Femur,_shape_of_the_process_for_the_attachment_of_the_caudifemoralis_musculature mound-like_and_rounded sharp_flange;

{505 Femur,_process_for_the_attachment_of_the_caudifemoralis_musculature_in_medial_or_lateral_view symmetrical,_with_the_proximal_and_distal_margins_forming_similar_low-angle_slopes_to_the_shaft asymmetrical,_with_the_distal_margin_forming_a_steeper_angle_to_the_shaft;

{506 Femur,_proximodistal_extension_of_the_process_for_the_attachment_of_the_caudifemoralis_musculature restricted_to_the_proximal_half_of_the_shaft_and_low distally_extended_beyond_mid-shaft_and_well_posteriorly_developed;

{507 Femur,_bone_wall_thickness_at_or_near_midshaft thickness/diameter_>0.3 thin,_thickness/diameter_<0.3_(1);

{508 Femur,_shaft diameter_constant_or_widening_distally diameter_distally_narrowed;

{509 Femur,_distal_transverse_width_versus_total_length 0.08-0.11 0.13-0.24 0.26-0.36 0.39-0.41;

{510 Femur,_distal_condyles prominent,_strong_dorsoventral_expansion_(in_sprawling_orientation)_restricted_to_the_distal_end not_projecting_markedly_beyond_shaft_and_expand_gradually_if_there_is_any_expansion;

{511 Femur,_distal_articular_surface uneven,_fibular_condyle_projecting_distally_distinctly_beyond_tibial_condyle both_condyles_prominent_distally_and_approximately_at_same_level both_condyles_do_not_project_distally_(distal_articular_surface_concave_or_almost_flat);

{512 Femur,_anterior_extensor_groove absent,_anterior_margin_of_the_bone_straight_or_convex_in_distal_view present,_anterior_margin_of_the_bone_concave_in_distal_view;

{513 Femur,_surface_between_the_lateral_condyle_and_crista_tibiofibularis_on_the_distal_surface smooth_ deep_groove;

{514 Femur,_shape_of_lateral_condyle_in_distal_view lateral_surface_is_rounded_and_mound-like lateral_surface_is_triangular_and_sharply_pointed_;

{515 Tibia,_total_length_versus_total_length_of_the_femur 0.46-0.51 0.60-0.65 0.70-1.27 1.41-1.46;

{516 Tibia,_distinctly_anteriorly_projected_process_beyond_the_articular_portion_for_the_femur_on_the_proximal_end_(=_cnemial_crest) absent present_and_anteriorly_straight_ present_and_curved_anterolaterally_;

{517 Tibia,_proximal_surface_of_the_lateral_condyle convex_or_flat depressed_;

{518 Tibia,_lateral_posterior_condyle_of_the_proximal_end offset_anteriorly_from_the_medial_posterior_condyle level_with_the_medial_posterior_condyle_at_its_posterior_border;

{519 Tibia,_lateral_surface_of_the_proximal_half smooth with_a_longitudinal_crest_(=_fibular_crest);

{520 Tibia,_posterolateral_process_(=_lateral_malleolus)_on_the_distal_end absent present;

{521 Tibia,_posterior_surface_of_the_distal_end rounded distinct_proximodistally_oriented_ridge_present;

{522 Tibia,_posterior_side_of_the_distal_portion smooth_and_featureless dorsoventrally_oriented_groove_or_gap;

{523 Tibia,_lateral_side_of_the_distal_portion smooth/rounded proximodistally_oriented_groove;

{524 Fibula,_proximal_end_in_proximal_view round_or_slightly_elliptical transversely_compressed;

{525 Fibula,_anterior_edge_of_the_proximal_portion rounded_ tapers_to_a_point_and_arched_anteromedially;

{526 Fibula,_proximal_portion_in_lateral_view symmetrical_or_nearly_symmetrical posterior_part_expanded_posteriorly;

{527 Fibula,_transverse_width_at_mid-length subequal_to_transverse_width_of_the_tibia distinctly_narrower_than_transverse_width_of_the_tibia;

{528 Fibula,_area_of_attachment_of_the_iliofibularis_muscle not_on_a_prominent_process_ on_a_low,_distinct_tubercle on_a_hypertrophied_tubercle;

{529 Fibula,_location_of_the_attachment_site_of_the_iliofibularis_muscle near_the_proximal_portion near_the_midpoint_between_the_proximal_and_distal_ends;

{530 Fibula,_distal_end_in_lateral_view angled_anterodorsally_(asymmetrical) rounded_or_flat_(symmetrical);

{531 Proximal_tarsals,_articulation_between_astragalus_and_calcaneum roughly_flat concavoconvex_with_concavity_on_the_calcaneum concavoconvex_with_concavity_on_the_astragalus;

{532 Proximal_tarsals,_foramen_for_the_passage_of_the_perforating_artery_between_the_astragalus_and_calcaneum_(=_perforating_foramen) present absent;

{533 Astragalus,_crural_facets separated_by_a_non-articular_surface continuous;

{534 Astragalus,_margin_between_tibial_and_fibular_facets grades_smoothly_into_anterior_hollow separated_by_a_prominent_ridge_from_anterior_hollow_;

{535 Astragalus,_tibial_facet concave,_flat_or_flexed divided_into_distinct_posteromedial_and_anterolateral_basins;

{536 Astragalus,_ascending_process_(=_anterior_ascending_process) absent present,_occupying_most_of_the_anteroposterior_depth_of_the_astragalus present,_restricted_to_the_anterior_half_of_the_astragalar_depth_;

{537 Astragalus,_anterior_hollow shallow_depression reduced_to_a_foramen_(=_extensor_canal)_or_absent;

{538 Astragalus,_posterior_groove present absent;

{539 Astragalus,_anteromedial_corner_in_proximal_view obtuse acute;

{540 Astragalus,_dorsolateral_margin overlaps_the_anterior_and_posterior_portions_of_the_calcaneum_equally posterior_corner_dorsally_overlaps_the_calcaneum_much_more_than_the_anterior_portion_;

{541 Astragalus,_articulation_with_distal_tarsal_4 poorly_defined well_defined;

{542 Calcaneum,_articular_facet_for_the_astragalus lies_completely_medial_to_the_fibular_facet lies_partially_ventral_to_the_fibular_facet;

{543 Calcaneum,_development_of_lateral_margin calcaneum_terminating_in_unthickened_margin roughened_tuberosity_present_laterally;

{544 Calcaneum,_calcaneal_tuber_(=_expansion_of_the_lateral_margin_of_the_bone) absent_or_incipient_ prominent;

{545 Calcaneum,_orientation_of_calcaneal_tuber lateral,_between_0º?35º_ posterolateral,_deflected_between_36º?70º posterior,_between_71º?90º;

{546 Calcaneum,_proportions_of_calcaneal_tuber_at_the_midshaft taller_than_broad about_the_same_or_broader_than_tall just_short_twice_the_transverse_width_of_the_fibular_facet;

{547 Calcaneum,_calcaneal_tuber_distal_end rounded_and_unexpanded flared,_dorsally_and/or_ventrally;

{548 Calcaneum,_calcaneal_tuber_distal_end_in_proximal_or_distal_view tapering_or_squared expanded;

{549 Calcaneum,_distal_surface_of_calcaneal_tuber_with_a_vertical_median_depression absent present;

{550 Calcaneum,_ventral_notch_between_the_main_body_and_the_calcaneal_tuber absent present;

{551 Calcaneum,_ventral_articular_surface_for_distal_tarsal_4_and_the_distal_end_of_the_calcaneal_tuber continuous_ separated_by_a_clear_gap separated_by_a_gap_with_a_laterally_and_medially_delimited_ventral_fossa;

{552 Calcaneum,_fibular_facet slightly_convex_or_flat_ hemicylindrical_ concave_;

{553 Calcaneum,_articular_facets_for_the_fibula_and_astragalus connected_by_a_continuous_surface separated;

{554 Calcaneum,_articular_surfaces_for_fibula_and_distal_tarsal_4 separated_by_a_non-articular_surface continuous_;

{555 Calcaneum,_transverse_width_of_the_distal_articular_surface_versus_transverse_width_of_the_astragalus 0.28-0.33 0.42-0.48 0.54-1.22;

{556 Distal_tarsals,_medial_pedal_centrale present_and_does_not_contact_tibia present_and_contacts_tibia absent_as_a_separate_ossification;

{557 Distal_tarsals,_distal_tarsal_1 present absent;

{558 Distal_tarsals,_distal_tarsal_2 present absent;

{559 Distal_tarsals,_distal_tarsal_4_transverse_width broader_than_distal_tarsal_3 subequal_to_distal_tarsal_3_;

{560 Distal_tarsals,_articular_facet_for_metatarsal_V_on_distal_tarsal_4 more_than_half_of_the_lateral_surface_of_the_bone less_than_half_of_the_lateral_surface_of_the_bone;

{561 Distal_tarsals,_proximal_surface_of_distal_tarsal_4 flat distinct,_proximally_raised_region_on_the_posterior_portion_(=_heel_of_Sereno_and_Arcucci,_1994);

{562 Distal_tarsals,_distal_tarsal_5 present absent;

{563 Pes,_foot_length_(articulated_fourth_metatarsal_and_digit)_versus_tibia-fibula_length >1 <1;

{564 Metatarsus,_configuration metatarsals_diverging_from_ankle_ compact,_metatarsals_I?IV_tightly_bunched;

{565 Metatarsus,_metatarsals_overlapping_proximally absent present;

{566 Metatarsus,_length_of_the_longest_metatarsal_versus_length_of_the_tibia 0.20-0.23 0.29-0.32 0.37-0.59 0.62-0.65;

{567 Metatarsus,_metatarsals_I_and_V_mid-shaft_diameters subequal_or_greater_than_those_of_metatarsals_II_to_IV lower_than_those_of_metatarsals_II_to_IV;

{568 Metatarsus,_length_of_metatarsal_I_versus_metatarsal_III 0.17-0.21 0.27-0.33 0.38-0.42 0.46-0.79 0-93-0.97;

{569 Metatarsus,_anteromedial_portion_of_the_shaft_of_metatarsal_I smooth_or_slight_ridge distinct,_rugose_ridge_present;

{570 Metatarsus,_length_of_the_metatarsal_II_versus_length_of_the_metatarsal_IV 0.52-0.56 0.60-0.85 0.90-1.02 1.06-1.15;

{571 Metatarsus,_metatarsal_II_midshaft_diameter less_than_or_equal_to_the_midshaft_diameter_of_the_metatarsals_I-IV more_than_the_midshaft_diameter_of_metatarsal_I;

{572 Metatarsus,_metatarsal_IV_mid-shaft_diameter subequal_to_that_of_metatarsal_III lower_than_that_of_metatarsal_III;

{573 Metatarsus,_length_of_metatarsal_IV_versus_length_of_metatarsal_III 0.85-1.00 1.04-1.08 1.11-1.28 1.31-1.34;

{574 Metatarsus,_distal_articulation_surface_of_the_metatarsal_IV broader_than_deep_(nearly_symmetrical) broad_as_deep_or_deeper_than_broad_(asymmetrical);

{575 Metatarsus,_dorsal_prominence_separated_from_the_proximal_surface_by_a_concave_gap_in_metatarsal_V absent present;

{576 Metatarsus,_metatarsal_V_with_a_hook-shaped_proximal_end absent,_articular_face_for_distal_tarsal_4_aligned_to_the_medial_margin_of_the_shaft present,_with_a_gradually_medially_curved_proximal_process present,_with_an_abruptly_medially_flexed_proximal_process_and,_as_a_result,_the_metatarsal_acquires_a_L-shape_in_dorsal_or_ventral_view;

{577 Metatarsus,_metatarsal_V_outer_process_on_the_proximal_lateral_margin absent,_smooth_curved_margin present,_prominent_pointed_process;

{578 Metatarsus,_metatarsal_V_lateral_plantar_tubercle_in_mature_individuals absent present;

{579 Metatarsus,_metatarsal_V_medial_plantar_tubercle_in_mature_individuals absent present;

{580 Pedal_digits,_length_of_digit_III_versus_length_of_digit_IV 0.64-0.77 0.82-0.83 0.87-1.44;

{581 Pedal_digits,_phalanges_on_pedal_digit_V present_and_'fully'_developed_first_phalanx present_and_'poorly'_developed_first_phalanx absent;

{582 Pedal_digits,_ratio_of_lengths_of_pedal_digits_V_and_I 0.30-0.85 1.37-3.07;

{583 Pedal_digits,_phalanx_V-1 subequal_to_or_shorter_than_other_non-ungual_phalanges metatarsal-like,_considerably_longer_than_other_non-ungueal_phalanges;

{584 Pedal_digits,_distal_articular_portion_of_distal_pedal_phalanges lateral_and_medial_sides_parallel_or_near_parallel_ lateral_and_medial_sides_converging_anteriorly;

{585 Pedal_digits,_pedal_unguals weakly_transversely_compressed,_rounded_and_triangular_in_cross-section dorsolaterally_compressed strongly_transversely_compressed,_with_a_sharp_dorsal_keel;

{586 Pedal_digits,_ventral_tubercle_in_unguals absent_or_small well_developed_and_extended_ventral_to_the_articular_portion_of_the_ungual;

{587 Osteoderms,_dorsal_osteoderms absent present,_one_row_ present,_two_rows present,_more_than_two_rows;

{588 Osteoderms,_sculpture_on_their_external_surface absent present;

{589 Osteoderms,_coarse_and_incised_ornamentation_composed_of_central_regular_pits_of_subequal_size_and_contour_on_the_external_surface_of_the_dorsal_osteoderms absent present;

{590 Osteoderms,_dorsal_prominence_on_the_external_surface_of_paramedian_osteoderms absent longitudinal_keel,_extending_along_all_or_most_of_the_anteroposterior_length_of_the_osteoderm_as_a_transversely_compressed_flange blunted,_anteroposteriorly_restricted_eminence;

{591 Osteodemrs,_paramedian_osteoderms thin very_thick;

{592 Osteoderms,_relation_between_paramedian_dorsal_osteoderms_and_presacral_vertebrae one_to_one_(includes_pairs) more_than_one_osteoderm;

{593 Osteoderms,_dorsal_osteoderm_alignment_dorsal_to_the_dorsal_vertebrae staggered_ one_to_one;

{594 Osteoderms,_dimensions_of_presacral_dorsal_osteoderms square-shaped,_about_equal_dimensions longer_than_wide wider_than_long_;

{595 Osteoderms,_unornamented_anterior_articular_lamina_on_paramedian_osteoderms absent present;

{596 Osteoderms,_anterior_edge_of_paramedian_presacral_osteoderms straight_or_rounded with_a_distinct_anterior_process;

{597 Osteoderms,_presacral_paramedian_osteoderms_with_a_distinct_longitudinal_bend_near_the_lateral_edge absent present;

{598 Osteoderms,_appendicular_osteoderms absent present;

{599 Osteoderms,_ventral_osteoderms absent present,_scattered,_not_forming_a_carapace_ present,_forming_a_carapace_;

{600 Maxilla,_posterolateral_surface:_directly_adjacent_to_alveolar_margin_(0),_lateral_process_of_maxilla_present,_creating_distinct_space_between_maxillary_alveoli_and_posterolateral_surface_of_the_maxilla_(1);

{601 Maxilla,_medial_surface_dorsal_to_tooth_row:_smooth_(0),_prominent_anteroposteriorly_oriented_ridge_present_(1);

{602 Teeth,_crown_height_of_the_upper_dentition_compared_with_lower_dentition:_similar_tooth_crown_height_(0),_the_upper_dentition_is_shorter_relative_to_the_taller_lower_dentition_(1);

{603 Teeth,_morphology_of_crown_base:_single,_pointed_crown_(0),_flattened_platform_with_pointed_cusps_(1),_mesiodistally_arranged_cusps_(2);

{604 Cervical_and_dorsal_vertebrae,_shape_of_posterior_articular_surface:_planar_(0),_concave_(1),_convex_(2);

{605 Dorsal_vertebrae,_diapophysis,_position:_anterior_portion_of_the_neural_arch/centrum_(0),_anteroposterior_middle_of_the_neural_arch/centrum_(1);

{606 Dorsal_vertebrae,_spinoprezygapophyseal_lamina:_absent_(0),_present_(1);

{607 Dorsal_vertebrae,_spinopostzygapophyseal_lamina:_absent_(0),_present_(1);

{608 Dorsal_vertebrae,_height_of_neural_spine_in_anterior_dorsals:_lower_than_two_times_the_height_of_its_respective_centrum_(0),_equal_or_higher_than_two_times_the_height_of_its_respective_centrum_(1);

{609 Caudal_vertebrae,_length_of_the_anterior_caudal_vertebrae_(caudal_vertebrae_1?10)_relative_to_posterior_caudal_vertebrae_(25+):_nearly_the_same_length_(0),_posterior_caudal_vertebrae_much_longer_(1);

{610 Clavicle,_portion_articulated_with_the_interclavicle,_shape:_broader_than_distal_portion_of_clavicle_(0),_similar_in_narrowness_to_the_distal_portion_of_the_clavicle_(1);

{611 Humerus,_entepicondylar_crest:_exhibits_a_curved_proximal_margin_(0),_exhibits_a_prominently_angled_proximal_margin_(1);

{612 Ilium,_postacetabular_process_in_lateral_view:_squared_or_rounded_(0),_subtriangular,_tapering_posteriorly_(1);

{613 Pedal_digits,_penultimate_phalanges_(last_phalanx_before_ungual):_shorter_or_sub-equal_than_the_more_proximal_phalanges_(0),_distinctly_longer_than_the_more_proximal_phalanges_(1);

{614 Primordial_sacral_vertebra_two,_sacral_rib consist_of_a_single_body_in_one_plane_(could_contain_a_lateral_notch) has_a_separate_posterolateral_process_positioned_dorsal_and_posterior_to_the_main_body_of_the_sacral_rib;

{615 Scapula,_posterior_edge_of_the_blade_just_dorsal_to_the_glenoid_region smoothly_transversely_convex with_a_distinct,_longitudinal_sharp_ridge;

{616 Ilium,_ventral_portion,_ischial_peduncle,_lateral_view nearly_straight_or_slightly_concave distinct_notch_(=dorsal_expansion)_between_the_posterior_and_anterior_ends;

{617 Femur,_distal_end,_medial_condyle_in_posterior_view smooth_surface_or_a_small_depression well_defined_proximodistally_oriented_scar_extending_from_the_posterior_portion_of_the_condyle_well_proximally;

{618 Fibula,_anterior_edge gently_rounded distinct_ridge_paralleling_the_shaft;

{619 Proximal_tarslas,_fusion_between_astragalus_and_calcaneum absent present;

;

ccode + 0 1 6 9 16 18.20 27 28 35 39 41 49 53 65 70 74 75 121 126 145 152 155 156 170 175 176 186 201 220 226 262 265 278 282 323 326 330 336 344 350 351 353 360 364 369 376 378 397 409 423 429 434 445 447 453 457 459 462 471 477 481 482 488 489 503 509 515 528 536 545 551 555 556 566 568 570 573 580 581 587 *;

proc /;

comments 647

{0 51 Based on Reisz (1977: fig. 2) (MDE, 18 September 2012).;

{0 164 Borderline_--_depends_on_left_vs_right_parietal..._(DM);

{0 170 Borderline._(DM);

{0 239 Based on Reisz (1981: 24) (MDE ,10 February 2014).;

{0 267 Based on Reisz (1981: fig. 11) (MDE, 10 February 2014).;

{0 291 Based on Reisz (1981: 26) (MDE, 10 February 2014).;

{0 302 Based on Reisz (1981: 27) (MDE, 10 February 2014).;

{0 324 Based on Reisz (1981: 30) (MDE, 10 February 2014).;

{0 330 Ratio=2.02 in the fourth cervical vertebra based on Peabody (1952: fig. 3) (MDE, 18 September 2012).;

{0 331 Based on Reisz (1981: 37) (MDE, 28 March 2015).;

{0 352 Based on Reisz (1981: 33, 34) (MDE, 6 March 2014).;

{0 356 The ratio is lower than 0.5 based on Reisz (1981: 34) (MDE, 2 April 2013).;

{0 364 Based on Reisz (1981: 34) (MDE, 12 February 2014).;

{0 380 Based on Reisz (1981: 36) (MDE, 12 February 2014).;

{0 382 Based on Peabody (1952: 22) and Reisz (1981: 4) (MDE, 12 February 2014).;

{0 473 Based on Reisz (1981: 42): a second, smaller process extends ventrolaterally from the ridge connecting the lateral pubic tubercle to the acetabulum. This process ... probably served as the origin of the ambiens and pubotibialis muscles (MDE, 12 February 2014).;

{0 563 Based on Peabody (1952: fig. 6a) (MDE, 12 February 2014).;

{1 89 The lacrial duct opens laterally on the lacrimal, close to the posterior margin of the bone (MNHN 1908-32-57) (MDE, 10 August 2013).;

{1 107 The morphology of the suture between the prefrontal and nasal cannot be confidently determined and, particularly, because the skull is exposed in medial view (MNHN 1908-32-57) (MDE, 11 August 2013).;

{1 122 The anterior end of the anterior process of the preserved postorbital and the parietals are missing. As a result, it is not possible to determine if the postfrontal participated in the supratermporal fenestra or not (i.e. if it was a contact between the postorbital and parietal) (MNHN 1908-32-57) (MDE, 10 August 2013).;

{1 127 Only the mould of the medial surface of the postorbital is preserved and it cannot be determined the morphology of the lateral surface of the bone (MNHN 1908-32-57). Accordingly, the state of this character was changed from (0) to (?) (MDE, 10 August 2013).;

{1 149 It is not clear if the quadratojugal was absent or not (Bicklemann et al. 2009) (MDE, 10 August 2013).;

{1 336 The cervicals lack a depression or pit lateral to the base of the neural arch (MNHN 1908-32-57). Accordingly, the state of this character was changed from (1) to (0) (MDE, 11 August 2013).;

{1 349 Based on Currie (1980) (MDE, 6 February 2014).;

{1 352 The condition of this character cannot be confidently determined in the holotype of Acerosodontosaurus (MNHN 1908-32-57). As a result, it is preferred here to score the character as (?) rather than (1) (MDE, 11 August 2013).;

{1 356 This ratio should be similar to Youngina because of the very short transverse processes (MDE, 6 February 2014).;

{1 367 Currie (1980) described that the dorsal ribs are holochepahlous and agrees with personal observation of MNHN 1908-32-57 (MDE, 6 February 2014).;

{1 427 Based on MNHN 1908-32-57 (MDE, 2 June 2013).;

{2 20 The skulls that preserve the anterir tip of the maxilla are strongly dorsoventrally compressed and, as a result, the scoring is codified as missing data (MDE, 7 February 2014).;

{2 26 Based on Gow (1975: fig. 1) (MDE, 15 October 2012).;

{2 33 Based on GHG K 106 (MDE, 7 February 2014).;

{2 35 Based on BP/1/2871 (MDE, 27 March 2015).;

{2 41 Based on Broom (1922:273) and Gow (1975) (MDE,12 February 2014).;

{2 44 Based on SAM-PK-K7578 (MDE, 6 September 2014).;

{2 48 Based on GHG K 106 (MDE, 7 February 2014).;

{2 75 Based on GHG K106 (MDE, 20 November 2012).;

{2 88 The foramen for the opening of the nasolacrimal duct could not be located in any of the studies specimens (e.g. BP/1/2459, 3859, GHG K 106, GHG RS 160, TM 1490, 4095) (MDE, 11 August 2013).;

{2 89 The foramen for the opening of the nasolacrimal duct could not be located in any of the studies specimens (e.g. BP/1/2459, 3859, GHG K 106, GHG RS 160, TM 1490, 4095) (MDE, 11 August 2013).;

{2 90 The ventral margin of the ventral temporal bar (i.e. formed by the posterior process of the jugal and the anterior process of the quadratojugal) is concave in BP/1/3859 (MDE, 11 August 2013).;

{2 94 Based on BP/1/3859 (MDE, 7 February 2014).;

{2 107 The suture between the prefrontal and nasal is anterolaterally oriented along its entire extension (BP/1/3859, GHG K 106) (MDE, 11 August 2013).;

{2 164 Ratio_suture_anterior/posterior_to_foramen_in_Carroll_1981:_fig._9:_7.15/5.58_(ML);

{2 191 Based on UC 1528 (MDE, 6 September 2014).;

{2 192 Based on UC 1528 (MDE, 6 September 2014).;

{2 196 Based on Gow (1975: 94) (MDE, 10 February 2014).;

{2 221 Based on Evans (1987: 195) (MDE, 7 September 2014).;

{2 224 Based on Gardener et al. (2010: fig. 3) (MDE, 15 October 2012).;

{2 233 Based on Evans (1987: fig. 2a) (MDE, 7 September 2014).;

{2 239 Based on Evans (1987: 196) (MDE, 7 September 2014).;

{2 249 Based on Evans (1987: 201) (MDE, 31 March 2015).;

{2 256 Based on Gardner et al. (2010: 8) (MDE, 9 February 2014).;

{2 270 Based on GHG K 106 (dorsally expanded) and SAM-PK-K8565 (mostly horizontal) (MDE, 9 February 2014).;

{2 298 Based on Gow (1975) and pers. obs. of BP/1/3859 and GHG K 106 (MDE, 11 August 2013).;

{2 304 Crowns are labiolingually compressed in GHG RS 160 and GHG K106, but not compressed in TM 1490 (MDE, 9 February 2014).;

{2 308 The hyoids were not recognized in the studied specimens and neither mentioned by Gow (1975) (MDE, 12 August 2013).;

{2 323 Based on Smith and Evans (1996) (MDE, 11 February 2014).;

{2 356 Ratio=0.46 based on BP/1/3859 vertebrae 9?12 (MDE, 2 April 2013).;

{2 365 Based on Gow (1975: 95) (MDE, 15 October 2012).;

{2 382 Based on Smith and Evans (1996: fig. 4c, d) (MDE, 3 September 2013).;

{2 403 The presence of cleithrum is uncertain in Youngina capensis (Smith and Evans, 1996: 296) (MDE, 3 September 2013).;

{2 408 The anterior half of the interclavicle is T-shaped, with sharp angles between lateral and posterior processes (Gow, 1975: 9c; Smith and Evans, 1996: 6c) (MDE, 3 September 2013).;

{2 411 Based on Gow (1975: 95) (MDE, 15 October 2012).;

{2 412 The presence of mineralized sternal plates in Youngina was confirmed by Smith and Evans (1996) (MDE, 4 September 2013).;

{2 413 50.9 mm estimated forelimb in SAM-PK-K7710a based on BP/1/3859. 67 hindlimb of SAM-PK-K710a. Ratio= 0.76 (MDE, 22 September 2014).;

{2 414 Based on BPI/1/3859 (MDE, 15 October 2012).;

{2 426 A supinator process and an ectopicondylar groove are present is Youngina (BP/1/3859). Accordingly, the scoring of this character was changed from (?) to (1) (MDE, 11 August 2013).;

{2 429 Youngina lacks an olecranon process in the ulna (Gow 1975: fig. 9a) (MDE, 11 August 2013).;

{2 433 The distal end is concave in BP/1/3859, but possibly because of the lack of complete ossification of the distal end (MDE, 11 February 2014).;

{2 435 Based on Gow (1975: 97) (MDE, 15 October 2012).;

{2 440 Based on Gow (1975: 95) (MDE, 29 March 2015).;

{2 454 The pelvic girdle is solid in Youngina (BP/1/3859). Accordingly the scoring of this character was changed from (1) to (0) (MDE, 11 August 2013).;

{2 470 Based on Smith and Evans (1996: 297) (MDE, 3 September 2013).;

{2 478 Based on Smith and Evans (1996: 297) (MDE, 3 September 2013).;

{2 507 BP/1/3859 was prepared with acid and, as a result, the thickness of the bone wall may look artificially thinner (MDE, 11 February 2014).;

{2 532 It seems that it was present in adult specimens (Broom, 1921; Smith and Evans, 1996: fig. 8d) (MDE, 3 September 2013).;

{2 562 Based on Broom (1921) (Smith and Evans: 1996: fig. 8d) (MDE, 3 September 2013).;

{2 563 The metatarsal IV + digit 4 are considerably longer than the tibia (Smith and Evans, 1996: table 1) (MDE, 4 September 2013).;

{2 565 The metatarsals overlap proximally with each other (BP/1/3859). Accordingly the scoring of this character was changed from (?) to (1) (MDE, 11 August 2013).;

{3 76 The snout of AM 3585 is broad and the natural moulds of the nasals indicate that these bones were dorsal elements (MDE, 3 September 2012).;

{3 163 The dorsal surface of the parietals are too damaged to determine confidently the size and shape of the pineal foramen (MDE, 14 February 2014).;

{3 191 Based on AM 3585 (MDE, 16 September 2012).;

{3 216 Based on AM 3585 (MDE, 16 September 2012).;

{3 298 In AM 3585 it couldn't be discern the presence of alveoli in the tooth bearing bones and the tooth seem to be ankylosed to the bone, but the poor state of preservation of the specimen prevents assessing the condition confidently (MDE, 3 September 2012).;

{4 115 The posterolateral process of the frontal is long and the suture with the parietal forms an acute angle with the parasagittal plane (Fraser, 1982: fig. 1c). Accordingly, the scoring of this character was changed from (1) to (2) (MDE, 11 August 2013).;

{4 163 25.5% based on Fraser (1982: fig. 1c). Accordingly, the scoring of this character was changed from (1) to (0) (MDE, 11 August 2013).;

{4 191 Based on Fraser (1982: 718) (MDE, 16 September 2012).;

{4 289 The postdentary bones of Planocephalosaurus are fused with each other (Fraser, 1982) and, as a result, it is not possible to determine the degree of exposition of the angular in lateral view (MDE, 11 August 2013).;

{4 304 Based on Fraser (1982: 714, 719) (MDE, 3 March 2014).;

{4 330 The exact position of the cervical vertebra illustrated by Fraser and Walkden (1984: fig. 5) (MDE, 12 September 2012).;

{4 331 Based on Fraser and Walkden (1984: 576) (MDE, 3 March 2014).;

{4 332 Based on Fraser and Walkden (1984: 576) (MDE, 31 March 2015).;

{4 345 Based on Fraser and Walkden (1984: 580) (MDE, 12 September 2012).;

{4 356 Ratio=0.18?0.25 based on Fraser and Walkden (1984: figs. 5, 6) (MDE, 2 April 2013).;

{4 369 Second sacral bifurcated, contrasting with the first one (Fraser Walkden, 1984) (MDE, 12 August 2013).;

{4 409 The posterior ramus of the interclavicle is not known (Fraser and Walkden 1984) (MDE, 12 August 2013).;

{4 424 In Planocephalosaurus the entepicondyle is strongly developed in comparison with the shaft width at mid-length (Fraser and Walkden 1984: fig. 14). Accordingly, the scoring of this character was changed from (0) to (1) (MDE, 14 September 2012).;

{4 454 The acetabulum is completely closed (Fraser and Walkden 1984: fig. 16) (MDE, 12 August 2013).;

{5 21 Based on Evans (1980: fig. 1) (MDE, 20 September 2012).;

{5 41 Based on Evans (1980: 223) (MDE, 20 September 2012).;

{5 44 The presence of a septomaxilla is equivocal (Evans 1980: 220) (MDE, 20 September 2012).;

{5 74 Based on Evans (1980: 225) (MDE, 20 September 2012).;

{5 75 Based on Evans (1980: fig. 1c) (MDE, 20 September 2012).;

{5 89 Based on Evans (1980: 212) (MDE, 20 September 2012).;

{5 137 The condition is almost identical to that of Planocephalosaurus (compare Evans 1980: fig. 1 and Fraser 1982: fig. 1) (MDE, 20 September 2012).;

{5 149 Based on Evans (1980: 216) (MDE, 20 September 2012).;

{5 156 Based on Evans (1980: 212) (MDE, 20 September 2012).;

{5 163 Ratio=21.67% based on Evans (1980: fig. 9a) (MDE, 20 September 2012).;

{5 224 Based on Evans (1980: 227) (MDE, 14 February 2014).;

{5 227 Based on Evans (1980: fig. 1) (MDE, 21 September 2012).;

{5 246 Based on Evans (1980: 227) (MDE, 20 September 2012).;

{5 298 Based on Evans (1980: 236) (MDE, 20 September 2012).;

{5 306 Based on Evans (1980: 237) (MDE, 15 February 2014).;

{5 330 Ratio=ca. 1.22 based on Evans (1981: fig. 4c) (MDE, 21 September 2012).;

{5 331 Based on Evans (1981: 84) (MDE, 15 February 2014).;

{5 356 Ratio=0.15?0.30 based on Evans (1981: figs. 5, 6) (MDE, 2 April 2013).;

{5 381 Based on Evans (1981: fig. 11b) (MDE, 21 September 2012).;

{5 382 Based on Evans (1981: 91) (MDE, 10 October 2012).;

{5 391 Based on Evans (1981: 95) (MDE, 21 September 2012).;

{5 411 Evans (1981: fig. 15) clearly shows that the clavicle articulates with the ventral surface of the interclavicle (MDE, 10 October 2012).;

{5 429 Based on Evans (1981: 97) and the presence of a distinct and large posterior depression in the distal end of the humerus (MDE, 21 September 2012).;

{5 541 The astragalus possesses a deep, ventrally opened notch in anterior or posterior view to receive the distal tarsal 4 (see Evans, 1981: fig. 27a, b) (MDE, 17 February 2014).;

{5 581 Based on Evans (1981: 105) (MDE, 17 February 2014).;

{6 48 Based on Evans (1990: 208) (MDE, 4 April 2015).;

{6 331 Based on Evans (1991: 190) (MDE, 5 April 2015).;

{7 368 Based on MNHN.F.BR12154 (MDE, April 10 2015).;

{8 353 The condition cannot be determiend in most of the dorsal vertebrae (MDE, 10 March 2014).;

{8 424 The condition seems to be more similar to that of Youngina capensis (BP/1/3859) than to that of Prolacerta broomi (BP/1/2675) (MDE, 20 June 2012).;

{9 7 The parietals have a median, longitudinal sagital crest, and the surface immediately lateral to it are not depressed as it should be the case in a supratemporal fossa (NMK S 180) (MDE, 13 February 2014).;

{9 18 Based on Gottman-Quesada and Sander (2009: fig. 9) (MDE, 12 February 2014).;

{9 21 The condition of this character cannot be confidently assessed because the skulls of Protorosaurus are hevely crushed and compressed (MDE, 3 September 2012).;

{9 41 Based on Gottman-Quesada and Sander (2009: 140) (MDE, 3 September 2012).;

{9 51 Based on Modesto and Sues (2004) and Gottman-Quesada and Sander (2009) (MDE, 16 September 2012).;

{9 74 Gottman-Quesada and Sander (2009: 141) described 28 (+-1) tooth positions in the maxilla of NMK S 180 (MDE, 3 September 2012).;

{9 149 The presence of a quadratojugal is doubtful in Protorosaurus (MDE, 3 September 2012).;

{9 152 The presence of a quadratojugal is doubtful in Protorosaurus (MDE, 3 September 2012).;

{9 158 Gottman-Quesada and Sander (2009: 142) (MDE, 10 September 2012).;

{9 163 Gottman-Quesada and Sander (2009: 142) described that the presence of a pineal foramen seems to be a variable condition in Protorosaurus (MDE, 3 September 2012). ;

{9 186 Based on Gottman-Quesada and Sander (2009: 146) (MDE, 13 February 2014).;

{9 194 Gottman-Quesada and Sander (2009:146) (MDE, 10 September 2012).;

{9 213 Based on Gottman-Quesada and Sander (2009:146) (MDE, 3 September 2012).;

{9 216 Based on BSPG 1995 I 5 (MDE, 16 September 2012).;

{9 271 In at least two specimens (NMK S 180 and the Simon and Bartholomaeus specimen) seem to be two posterior processes in the dentary (MDE; 31 March 2015).;

{9 298 The height of the maxillary tooth crowns in labial and linguals views is subequal in the Simon Bartholomaeus specimen (SMNS 55387). This suggests that the tooth implantation is subthecodont (MDE, 15 February 2015).;

{9 302 Gottman-Quesada and Sander (2009: 148) described the crowns of Protorosaurus as straight. However, in BSPG 1995 I 5 (cast of WMsN P47361) a few maxillary teeth are slightly distally curved. As a result, I scored this character as polymorphic in Protorosaurus (MDE, 3 September 2012).;

{9 309 Gottman-Quesada and Sander (2009: 153) said that the presence of a notochordal canal in the vertebrae of the Protorosaurus specimens cannot be assessed. However, in the cast MB R2173 (original probably destroyed during WWII) the surface of the posterior facet of the centrum is concave and clearly not notochordal (MDE, 24 September 2012).;

{9 331 The specimens are too transversely compressed to determine the condition of this character (MDE, 13 February 2014).;

{9 336 The seventh cervical of WMsN P47361 has a shallow excaviation next to the base of the neural spine, but the more anterior cervical vertebrae lack such depression (MDE, 3 September 2012).;

{9 356 Ratio=0.38?0.45 based on postaxial vertebrae 8, 9 and 11 of BSPG 1995-I-5 cast of WMSN P47361 (MDE, 2 April 2013).;

{9 367 Based on Gottman-Quesada and Sander (2009: 158) (MDE, 3 September 2012).;

{9 372 Based on SMNS 55387, cast of the Simon Bartholomeus specimen (MDE, 13 February 2014).;

{9 374 Based on Gottman-Quesada and Sander (2004: 159) (MDE, 10 October 2012).;

{9 403 The presence of a cleithrum is ambiguous in Protorosaurus Gottman-Quesada and Sander (2009) (MDE, 3 September 2012).;

{9 413 Length of the forelimb of BSPG 1995 I 5, cast of WMsN P 47361: 203.8 mm. Lenth of the hindlimb of BSPG 1995 I 5, cast of WMsN P 47361, based on extrapolations with NHMW 1974 1635: 318.3 mm. Ratio: 0.64 (MDE, 22 September 2014).;

{9 424 The degree of development of the entepicondyle in Protorosaurus is more similar to Youngina than to Prolacerta (WMsN P47361) (MDE, 3 September 2012).;

{9 427 IN BSPG 1995 I 5 there are a well-developed condyle next to the ectepicondyle and it is very likely that its degree of development is understimated due to the strong dorsoventral degree of compression that suffered the bone. The same condition or even more developed distal condyles are observed in other individuals and in BSPG AS VII 1207 there is evidence of at least three distinct distal condyles (MDE, 13 September 2012).;

{9 444 Based on BSPG 1995 I 5 (MDE, 10 September 2012).;

{9 462 Based on Gottman-Quesada and Sander (2009:165) (MDE, 10 September 2012).;

{9 463 Based on Gottman-Quesada and Sander (2009: 165) (MDE, 26 February 2014).;

{9 470 In the Simon/Bartholomaeus specimen the thyroid fenestra is absent (SMNS cast, MDE pers. obs.) (MDE, 9 August 2013);

{9 487 Based on Simon and Bartholomaeus specimen (MDE, 31 March 2015).;

{9 489 The humeri and femora are strongly compressed and the diameter of the bones cannot be measured (MDE, 3 September 2012).;

{9 490 Based on BSPG AS VII 1207 (MDE, 13 February 2014).;

{9 508 Based on SMNS 55387 cast of Simon and Bartholomeus specimen (MDE, 13 February 2014).;

{9 510 Based on SMNS 55387 cast of Simon Bartholomaeus specimen (MDE, 12 March 2014).;

{9 538 Based on Gottman-Quesada and Sander (2009: fig. 25c) (MDE, 13 February 2014).;

{9 556 Based on SMNS 55387, cast of the Simon Bartholomaeus specimen, and Gottman-Quesada and Sanger (2009: figs. 25) (MDE, 10 September 2012).;

{9 562 It would be possible that the distal tarsal 5 is fused to the distal tarsal 4 (Gottman-Quesada and Sander (2009: 172) (MDE, 3 September 2012).;

{9 575 Based on Simon-Bartholomaeus specimen cast SMNS 55387 (MDE, 23 April 2014).;

{10 362 Based on SMNS 54783 (MDE, 22 September 2014).;

{10 365 There is an intercentrum at least between the last two dorsals in SMNS 90600 (MDE, 29 August 2014).;

{11 21 All the skulls of Macrocnemus bessanii are strongly compressed to assess the state of this character (MDE, 30 August 2012).;

{11 33 Based on Peyer (1937: fig. 32) (MDE, 17 February 2014).;

{11 34 Based on Peyer (1937: fig. 32) (MDE, 17 February 2014).;

{11 51 Based on PIMUZ T4822 (MDE, 16 September 2012).;

{11 58 Based on PIMUZ T4355 (MDE, 29 August 2014).;

{11 94 Based on PIMUZ T4822 (MDE, 10 October 2012).;

{11 112 Based on PIMUZ T4822 (MDE, 10 October 2012).;

{11 120 Based on PIMUZ T2472 (MDE, 17 February 2014).;

{11 129 The posterior process of the quadratojugal does not reach the posterior border of the supratemporal fenesrtra defined by the squamosal (PIMUZ T4822) (MDE, 30 August 2012).;

{11 149 The quadratojugal is probably absent in Macrocnemus (PIMUZ T4822), but I cannot assess the condition confidently (MDE, 30 August 2012).;

{11 152 I couldn't recognize a quadratojugal in Macrocnemus specimens (MDE, 30 August 2012).;

{11 184 Based on Peyer (1937: 55) (MDE, 17 February 2014).;

{11 188 Based on Besano II (BSPG 1973 I 86);

{11 194 Based on Peyer (1937: 60) and Besano II (MDE, 6 September 2012).;

{11 260 Based on PIMUZ T2472 (MDE, 17 February 2014).;

{11 298 The teeth are implanted into deep alveoli and not ankylosed to the tooth-bearing bone. The lingual margen of the alveoli seems to be lower than the labial margen, thus, the character is scores as subthecodont based on PIMUZ T2472, T4355 and T4822 (MDE, 30 August 2012).;

{11 312 Based on PIMUZ T4355 (MDE, 2 June 2013).;

{11 314 Based on PIMUZ T2472 (picture 386) (MDE, 10 September 2012).;

{11 315 Although a centrodiapophyseal lamina seems to be present in PIMUZ T4822, the strongly compressed nature of the Macrocnemus specimens turns difficult to assess the condition of this character (MDE, 10 September 2012).;

{11 316 Based on PIMUZ T4822 (10 September 2012).;

{11 330 Ratio: 3.76-3.83 in PIMUZ T4822 and this ratio is higher in larger specimens such as PIMUZ T4355 (MDE, 6 September 2012).;

{11 339 Based on PIMUZ T4822 (MDE, 16 September 2012).;

{11 345 Intercentra are present in the cervical series of PIMUZ T4822, but absent in PIMUZ T2472 and T4355 (MDE, 11 September 2012).;

{11 354 Although in at least one dorsal vertebra of PIMUZ T4822 there is a circular foramen in the lateral surface of the centrum, I couldn't recognize this feature in any other vertebra of other specimens (MDE, 10 October 2012).;

{11 356 Ratio=0.56 based on PIMUZ T2472 (MDE, 2 April 2013).;

{11 369 I have observed the presence of a dorsosacral vertebra in which a rib contacts the medial surface of the anterior end of the iliac blade in PIMUZ T2472 and T4822 (MDE, 30 August 2012).;

{11 372 Based on PIMUZ T2472 (MDE, 10 September 2012).;

{11 381 Based on PIMUZ T2472 (MDE, 22 September 2014).;

{11 411 Based on PIMUZ T4355 (MDE, 10 October 2012).;

{11 414 The condition of the character is not clear in the available specimens due to the strong degree of compression of the materials (MDE, 10 September 2012).;

{11 435 Based on PIMUZ T4355 (MDE, 10 October 2012).;

{11 454 The acetabular wall appears to be partially open in PIMUZ T4822 probably because of lack of preservation (MDE, 18 February 2014).;

{11 556 Based on Rieppel (1989: 380) (MDE, 18 February 2014).;

{11 563 Based on PIMUZ T4822 (MDE, 18 February 2014).;

{12 44 Following Dilkes (1998) and Nosotti (2007) (MDE, 2 September 2012).;

{12 60 Based on WIld (1973: fig. 5, 6) (MDE, 20 June 2012).;

{12 65 Based on Wild (1973: fig. 26) (MDE, 7 March 2014).;

{12 75 Based on Wild (1973) (MDE, 2 September 2012).;

{12 77 Based on PIMUZ T2484 (MDE, 11 September 2012).;

{12 84 Based on Wild (1973: fig. 1: f.n.) (MDE, 20 June 2012). ;

{12 89 Based on Wild (1973: fig. 1: f.n.) (MDE, 20 June 2012). ;

{12 107 Based on Nosotti (2007: fig. 44) (MDE, 2 Septermber 2012).;

{12 114 Based on PIMUZ T2189 (MDE, 7 March 2014).;

{12 115 Based on PIMUZ T2189 (MDE, 7 March 2014).;

{12 122 Based on Nosotti (2007: 81) (MDE, 11 August 2013).;

{12 149 The quadratojugal is absent (Wild, 1973, Nosotti, 2007) (MDE, 22 June 2012).;

{12 152 Tanystropheus lacks of a quadratojugal (Wild, 1973: figs. 8?10) and Nosotti (2007) (MDE, 20 June 2012).;

{12 156 This character is scored as unkown based on Nosotti (2007) (MDE, 2 September 2012).;

{12 158 In small individuals the parietals are not fused to each other, whereas the opposite occurs in large individuals (Nosotti 2007: 51). We have considered here only the adult condition (MDE, 11 September 2012).;

{12 163 The size of the pineal foramen seems to range between 26.90% to 18.09% (Wild, 1973: fig. 1) (MDE, 20 June 2012).;

{12 164 In different specimens the pineal foramen is positioned at the frontal-parietal suture or it is completely enclosed by the parietals (Nosotti 2007: 51). As a result, this character was scored as polymorphic for Tanystropheus longobardicus (MDE, 2 September 2012).;

{12 185 Based on Wild (1973).;

{12 187 The condition of this character seems to be variable within Tanytropheus longobardicus (Wild 1973, Nosotti 2007: 80) (MDE, 11 September 2012). Based on Wild (1973) (MDE, 10 March 2014).;

{12 210 Based on Nosotti (2007: 12) (MDE, 10 March 2014).;

{12 221 Based on PIMUZ T2189 (MDE, 10 March 2014).;

{12 297 Based on comparisons between the maxillae and dentaries several specimens (MDE, 10 March 2014).;

{12 298 Wild (1973) says that the kind of tooth implantation of Tanystrophaeus is subthecodont (page 48) (MDE, 20 June 2012).;

{12 302 Tanystropheus is strongly heterodont and the crowns possess a strong to slight distal curvature or completely lack it (MDE, 20 June 2012).;

{12 308 Based on Nisotti (2007, fig. 12) (MDE, 2 September 2012).;

{12 330 Ratio=14.25 in the fourth cervical of PIMUZ T2818 (MDE, 11 September 2012).;

{12 331 Based on Pritchard et al. (2015) (MDE, 28 March 2015).;

{12 335 Based on PIMUZ T2189 (MDE, 10 March 2014).;

{12 339 Based on PIMUZ T2818 (MDE, 11 September 2012).;

{12 340 Based on PIMUZ T2818 (MDE, 11 September 2012).;

{12 352 Based on SMNS 55341 (MDE, 09 August 2013).;

{12 356 Ratio=0.46 based on Wild (1973: fig. 52) (MDE, 3 April 2013).;

{12 357 Based on PIMUZ T2818 (MDE, 2 September 2012).;

{12 367 Based on Wild (1973: fig. 35) (MDE, 20 June 2012).;

{12 381 Based on PIMUZ T2817 (MDE, 11 September 2012).;

{12 389 The anterior margin of the scapular blade is not distintly concave, but neither continuously convex (MDE, 12 March 2014).;

{12 435 Based on Nosotti (2007: table 4, 6) (MDE, 11 October 2012).;

{12 439 Based on Nosotti (2007: 30, fig. 23) (MDE, 12 March 2014).;

{12 440 Based on Nosoti (2007: fig. 23) (MDE, 20 June 2012).;

{12 441 Based on Nosoti (2007: fig. 23) (MDE, 20 June 2012).;

{12 510 Based on SMNS 54626 (MDE, 12 March 2014).;

{12 532 Based on Wild (1974: 116) and Nosotti (2007: 35) (MDE, 11 October 2012).;

{12 541 Based on Nosotti (2007: 72) (MDE, 13 August 2013).;

{12 563 Based on PIMUZ T2817 (MDE, 2 September 2012).;

{13 91 Based on ZAR 08 (MDE, 1 May 2014).;

{13 94 Based on ZAR 08 (MDE, 1 May 2014).;

{13 263 Based on the right side of ZAR 08 (MDE, 2 May 2014).;

{13 326 A keel is present on the axis of ZAR 07 (MDE, 2 May 2014).;

{13 356 Based on ZAR 08 (MDE, 2 May 2014).;

{13 406 Based on ZAR 06 (2 May, 2014);

{15 88 No foramen for the lacrimal duct is present (Flynn et al., 2010: 677) (MDE, 18 February 2014).;

{15 89 No foramen for the lacrimal duct is present (Flynn et al., 2010: 677) (MDE, 18 February 2014).;

{15 99 The height of the base of the posterior process is measured orthogonally to the main axis of the process (MDE, 18 February 2014).;

{15 122 Based on Flynn et al. (2010: 679) (MDE, 14 February 2014).;

{15 136 Based on Flynn et al. (2010: fig. 1A) (MDE, 18 February 2014).;

{15 282 Flynn et al. (2010: 683) (MDE, 18 February 2014).;

{15 356 Ratio=0.49 in UA 8-26-98-265 (MDE, 18 February 2014).;

{18 35 The dorsolateral process of the premaxilla is well extended beyond the posteiror margin of the external naris (Spielmann et al. 2008: fig. 20). Accordingly, the scoring of this character was changed from (?) to (1) (MDE, 4 September 2012).;

{18 44 Based on Gregory (1945: 277) (MDE, 25 February 2014).;

{18 75 No skull of Trilophosaurus buettneri preserves both antorbital and postorbital regions (MDE, 4 September 2012).;

{18 76 The snout of Trilophosaurus buettneri is narrow and tall, and the nasal has a strong vertical contribution to the snout (Spielmann et al. 2008: figs. 20-22) (MDE, 4 September 2012).;

{18 88 The new interpretations of the skull of Trilophosaurus buettneri strongly differs from those of Gregory (1945) regarding the shape and position of the lacrimal and the foramen showed by this author should not be positioned within the lacrimal based on Parks (1969) and Spielmann et al. (2008). However, the latter author did not give information about the passage of the lacrimal duct in Trilophosaurus buettneri (MDE, 14 September 2012).;

{18 89 The new interpretations of the skull of Trilophosaurus buettneri strongly differs from those of Gregory (1945) regarding the shape and position of the lacrimal and the foramen showed by this author should not be positioned within the lacrimal based on Parks (1969) and Spielmann et al. (2008). However, the latter author did not give information about the passage of the lacrimal duct in Trilophosaurus buettneri (MDE, 14 September 2012).;

{18 90 The ventral margin of the postorbital region of the skull is severely damaged in TMM 31025-140 and the condition of the character cannot be confidently assessed (Spielmann et al. 2008: fig. 19) (MDE, 4 September 2012).;

{18 113 The orbital border of the frontal is broad in TMM 31025-207 (Spielmann et al. 2008: fig. 20e, f). Accordingly, the scoring of this character was changed from (0) to (1) (MDE, 4 September 2012).;

{18 149 The quadratojugal of TMM 31025-140 is broken and the anterior extent of this bone cannot be assessed with the currently available specimnes (Spielman et al. 2008: fig. 19). As a result, the scoring of this character has been changed from (2) to (?) (MDE, 4 September 2012). ;

{18 159 The parietals do not extend over the interorbital region in TMM 31025-140 (Spielmann et al. 2008: fig. 19) (MDE, 4 September 2012).;

{18 170 The postparietal cannot be discerned in Trilophosaurus buettneri (Gregory 1945: 278) (MDE, 4 September 2012).;

{18 205 Based on Spielmann et al. (2008: 27) (MDE, 13 November 2012).;

{18 213 The paroccipital processes of Trilophosaurus buettneri have a weak contact with the squamosal (Spiealmann et al. 2008: fig. 18) (MDE, 14 September 2012).;

{18 216 Based on Spielmann et al. (2008: figs. 25, 26) (MDE, 16 September 2012).;

{18 221 Based on Spielmann et al. (2008: 32) (MDE, 25 February 2014).;

{18 283 Scoring changed from that of PhD from (0) to (1) (MDE, 30 January 2015).;

{18 287 Based on Spielmann et al. (2008: fig. 27) (MDE, 16 September 2012).;

{18 288 Based on Pritchard et al. 2015 (MDE, 28 March 2015).;

{18 289 The exposition of the angular in lateral view is wide in TMM 31025-5 (Spielmann et al. 2008: fig. 28). Accordingly, the scoring was changed from (?) to (0) (MDE, 4 September 2012).;

{18 302 This scoring was changed from (1) to (2) because Trilophosaurus buettneri lacks of a distal curvature in the margin teeth (Spielmann et al. 2008: figs. 18, 20, 21) (MDE, 4 September 2012).;

{18 323 In the eighth postaxial vertebra the capitular rib faet is lost (Spielmann et al., 2008: 41) (MDE, 25 February 2014).;

{18 330 Some cervical vertebrae are longer then the posterior dorsals and others shorter (Spielman et al. 2008: appendix 10). Accordingly, the scoring of this character was changed from (0) to (0&1) (MDE, September 2012). Ratio=1.84-2.5 in the fourth and fifth cervical vertebrae based on Spielmann et al. (2008: appendix 10) (MDE, 13 September 2012).;

{18 345 Based on Spielmann et al. 2008 (fig. 30) (MDE, 13 September 2012).;

{18 356 Ratio=0.84 based on Spielmann et al. (2009: fig. 37) (MDE, 3 April 2013).;

{18 369 Trilophosaurus buettneri has two unequal sacral vertebrae (Spielmann et al. 2008) and, as a result, the scoring of this character was changed from (?) to (0) (MDE, 4 September 2012).;

{18 382 Based on the scoring of Pritchard et al. (2015: character 137) (MDE, 29 March 2015).;

{18 408 Gregory_1945:_pl._26._(DM);

{18 411 Based on Gregory (1945: 301) (MDE, 13 November 2012).;

{18 429 In Trilophosaurus buettneri the olecranon process is prominent but it is not ossified separately (Spielmann et al. 2008: figs. 69, 70). As a result, the scoring of this character was changed from (0) to (1) (MDE, 5 September 2012).;

{18 444 In TMM 31025-140 the lengh of manus is 16.8 cm based on Spielmann et al. (2008: fig. 74) and length of humerus is 17.0-17.6 based on Spielmann et al. (2008: appendix 10) (MDE, 13 September 2012).;

{18 470 Based on Gregory (1945: fig. 9) (MDE, 25 February 2014).;

{18 544 Gregory (1945: 316) described the absence of a strong "tuber calcis" in Trilophosaurus (MDE, 24 February 2014).;

{20 372 Based on a second primordial sacral previously referred to Trilophosaurus jacobsi (Spielmann et al. 2009: 285) (Spielmann et al. 2008, fig. 96) (MDE, 23 August 2016).;

{23 309 Based on Carroll (1976: 42) (MDE, 18 September 2012).;

{23 352 Based on AM 3591 (MDE, 18 September 2012).;

{23 357 Based on AM 3591 (MDE, 18 September 2012).;

{23 360 Dilkes (1998: 530) reported the absence of pits lateral to the neural spines in Noteosuchus. This condition is right for the preserved posterior dorsal vertebrae of AM 3591, but it could not be assessed in the anterior dorsal vertebrae. Although the pits are prensent in the posterior dorsal vertebrae of Howesia (SAM-PK-5886), in Mesosuchus the posterior dorsal vertebrae also lack of the pit, but it is present in the anterior dorsal vertebrae (SAM-PK-6046). Accordingly, the condition of this character is scored as missing data in Noteosuchus (MDE, 18 September 2012).;

{23 372 Based on AM 3591 (MDE, 18 September 2012).;

{23 470 Based on Carroll (1976: 47) (MDE, 10 October 2012).;

{23 544 Based on Carroll (1976: 48) (MDE, 18 September 2012).;

{23 555 The reconstruction of the ankle of Noteosuchus by Carroll (1976) looks rather weird (MDE, 17 September 2014).;

{23 556 I consider that cannot be confidently assessed that the central did not contact the tibia (MDE, 18 September 2012).;

{23 562 Based on Carroll (1976: 48) (MDE, 18 September 2012).;

{23 565 Based on Carroll (1976: 48) (MDE, 18 September 2012).;

{24 8 Dilkes (1998: character 10) scored Mesosuchus has having confluent external nares and the condition is clearly visible in SAM-PK-6536 (MDE, 27 August 2012).;

{24 18 See discussion of Modesto and Sues (2004: 347) regarding the scoring of this character in Mesosuchus (MDE, 16 September 2012).;

{24 41 Mesosuchus has two premaxillary teeth (Dilkes, 1998: 511) (MDE, 27 August 2012).;

{24 65 Based on Dilkes (1998: 505) and SAM-PK-6536 (MDE, 3 March 2014).;

{24 89 Based on Dilkes (1998: 505) (MDE, 13 September 2012).;

{24 156 The supratemporal of SAM-PK-6536 is considerably broader than the slit-like bone of Proterosuchus fergusi. Accordingly, it is scores as condition (0) (MDE, 27 August 2012).;

{24 163 Ratio=18.89% based on SAM-PK-6536 (Dilkes, 1998: fig. 5) (MDE, 27 August 2012).;

{24 170 Dilkes (1998: character 29) scores Mesosuchus as lacking of a postparietal (MDE, 27 August 2012).;

{24 185 Based on Dilkes (1998: character 38) (MDE, 11 September 2012).;

{24 203 Based on Dilkes (1998: 508) (MDE, 3 March 2014).;

{24 220 Cannot be determined bcause of the fusion between exoccipitals and basioccipital (MDE, 3 March 2014).;

{24 253 Based on Dilkes (1998: 509) (MDE, 3 March 2014).;

{24 254 Based on Dilkes (1998: character 48) (MDE, 3 March 2014).;

{24 255 Following Nesbitt (2011: character 94) (MDE, 5 March 2014).;

{24 312 Based on SAM-PK-6536 (MDE, 3 March 2014).;

{24 323 Based on SAM-PK-5882 and SAM-PK-6536 (MDE, 3 March 2014).;

{24 326 Based on SAM-PK-5882 (MDE, 3 March 2014).;

{24 349 Based on SAM-PK-5882 (MDE, 27 August 2012).;

{24 356 Ratio=ca. 0.5 based on Dilkes (1998: 513) (MDE, 2 April 2013).;

{24 372 Based on Dilkes (1998: character 87) (MDE, 11 September 2012).;

{24 408 Although DIlkes (1998) described the interclavicle of Mesosuchus as T-shaped its anterior end is diamond-shaped (MDE, 27 August 2012).;

{24 427 Based on SAM-PK-6536 (MDE, 11 September 2012).;

{24 432 Based on SAM-PK-6046 (MDE, 4 March 2014).;

{24 444 No specimen preserves both complete humerus and manus (MDE, 11 September 2012).;

{24 508 Based on the femur of SAM-PK-7416 (MDE, 11 September 2012).;

{24 540 Based on SAM-PK-7416 (MDE, 23 September 2014).;

{24 544 Although a calcaneal tuber was scored as absent in Mesosuchus by Dilkes (1998: character 119) it can be observed in the right hindlimb of SAM-PK-7416 (MDE, 27 August 2012).;

{24 563 The distal phalanges of the fourth pedal digit are not preserved (MDE, 27 August 2012).;

{24 575 Based on SAM-PK-7416 (MDE, 23 April 2014).;

{24 580 The pedal digit IV is not completely preserved in the available specimens of Mesosuchus (MDE, 11 September 2012).;

{25 18 See discussion of Modesto and Sues (2004: 347) regarding the scoring of this character in Howesia (MDE, 16 September 2012).;

{25 39 Based on Dilkes (1995: 669) (MDE, 10 September 2012).;

{25 67 Based on Dilkes (1998: character 16) (MDE, 10 September 2012).;

{25 74 I think that this character is not applicable for Howesia and more derived rhynchosaurs (MDE, 3 September 2012).;

{25 120 The maximum constriction of the olfactory tract mould is situated well posteriorly to the broken anterior margin of the frontals (SAM-PK-5885) (MDE, 7 March 2014).;

{25 163 Based on Dilkes (1995: 670) (MDE, 7 March 2014).;

{25 175 Based on SAM-PK-5885 (MDE, 3 September 2012).;

{25 191 Based on Dilkes (1995: 670) (MDE, 7 March 2014).;

{25 203 Based on Dilkes (1995: 670) (MDE, 10 September 2012).;

{25 205 Based on Dilkes (1995: 671) (MDE, 7 March 2014).;

{25 206 Based on DIlkes (1998: character 39) (MDE, 10 September 2012).;

{25 208 Based on Dilkes (1995: 671) (MDE, 7 March 2014).;

{25 224 SAM-PK-5885 seems to not be a fully mature individual (MDE, 7 March 2014).;

{25 228 The posterior surface of the occipital condyle of SAM-PK-5885 is strongly damaged (MDE, 7 March 2014).;

{25 239 Although Dilkes (1998: character 45) scored this character as (?) in Howesia the foramina for the exit of the carotids are clearly ventrally situated in the parashpenoid in SAM-PK-5885 (MDE, 10 September 2012).;

{25 289 Dilkes (1995) said that the mould of the angular indicates that it had a limited lateral exposure (MDE, 3 September 2012).;

{25 326 At least in the axis of SAM-PK-5885 (MDE, 3 September 2012).;

{25 336 At least in the axis of SAM-PK-5885 (MDE, 3 September 2012).;

{25 340 Based on the axis of SAM-PK-5885 (MDE, 10 September 2012).;

{25 372 Based on Dilkes (1998: character 87) and SAM-PK-5886 (MDE, 10 September 2012, 7 March 2014).;

{25 382 Based on Dilkes (1995: 676) (MDE, 7 March 2014).;

{25 386 The scapular blade of SAM-PK-5885 was apparently damaged (MDE, 7 March 2014).;

{25 389 The scapular blade of SAM-PK-5885 was apparently damaged (MDE, 7 March 2014).;

{25 391 Based on Broom (1906: plate 15) (MDE, 3 September 2012). ;

{25 405 Based on Broom (1906: plate 15) (MDE, 3 September 2012). ;

{25 408 Based on Broom (1906: plate 15) (MDE, 3 September 2012). ;

{25 427 The condition of this character cannot be confidently assessed based on Broom (1906) drawing and the humerus is currently lost (DIlkes 1998) (MDE, 11 September 2012).;

{25 534 The proximal tarsals are expoased in posterior view in the only specimen that preserves the hindlimbs (MDE, April 7 2015);

{25 544 Based on SAM-PK-5886 (MDE, 3 September 2012).;

{25 575 Based on Carroll (1976: fig. 8).;

{26 89 Based on the natural mould of the passage of the nasolacrimal duct (MDE, 8 September 2014).;

{27 191 Based on Benton (1990: 231) (MDE, 3 April 2014).;

{27 205 Based on NHMUK R1236 (MDE, 11 September 2014).;

{27 352 Based on NHMUK R1238 (MDE, 11 September 2014).;

{27 356 Based on SHYMS 2 (MDE, 11 September 2014).;

{27 358 Based on BATGM M20a (MDE, 30 October 2014).;

{27 359 Based on BATGM M20a (MDE, 30 October 2014).;

{27 372 Based on SHYMS 5 (MDE, 11 September 2014).;

{27 384 Based on NHMUK R1239 (MDE, 12 September 2014).;

{27 453 Based on SHYMS 6 (MDE, 12 Septemebr 2014).;

{27 476 Based on BATGM M20a, b (MDE, 30 October 2014).;

{27 534 The better preserved proximal tarsals are preserved in posterior view (MDE, April 8 2015).;

{27 563 Based on SHYMS 5 (MDE, 12 September 2014).;

{28 208 The occipital surface of hte braincase is very damaged (MDE, 9 September 2014).;

{29 356 Ratio=0.70 based on PIN 156/110 (MDE, 2 April 2013).;

{30 302 Young (1973: fig. 1) illustrates a tooth crown with a convex mesial margin that it is not currently preserved in the specimen (MDE, April 7 2015).;

{31 44 The septomaxilla of Prolacerta broomi possesses a curved lateral lip (Modesto and Sues, 2004) and, as a result, is not a completely flat sheet of bone. Accordingly, the scording of this character was changed from (2) to (1) (MDE, 11 August 2013).;

{31 102 State 0 in SAM-PK-K10797 and state 1 in BP/1/3575 and BP/1/5375 (MDE, 5 March 2014).;

{31 136 Based on UMZC 2003.41R (MDE, 5 April 2014).;

{31 156 However, the specimen that I identified as Prolacerta broomi in the GHG collection (431) seems to lack supratemporals and a supratemporal facet in both squamosals (MDE, 19 June 2012). Is this specimen actually Prolacerta broomi?;

{31 170 The postparietals are absent in Prolacerta broomi (Modesto and Sues, 2004). Accordingly, the scording of this character was changed from (1/2) to (2) (MDE, 11 August 2013).;

{31 185 Based on the desarticulated vomer of BPI/1/2675 (MDE, 13 September 2012).;

{31 195 Based on BP/1/5066 (MDE, 5 March 2014).;

{31 196 Based on BP/1/5066 (MDE, 5 March 2014).;

{31 204 Based on BP/1/5066 (MDE, 5 March 2014).;

{31 208 Based on SAM-PK-K10018 and Gow (1975: 105) (MDE, 5 September 2014).;

{31 213 In Prolacerta broomi the paroccipital processes possess a weak contact with the posterior temporal region of the skull (BPI/1/471) (MDE, 14 September 2012). ;

{31 216 Based on BP/1/2675 (MDE, 5 March 2014).;

{31 219 Based on BP/1/5066 (MDE, 5 March 2014).;

{31 220 Based on BP/1/5066 (MDE, 5 March 2014).;

{31 221 Based on BP/1/5066 (MDE, 5 March 2014).;

{31 230 The occipital neck is extremely short in BP/1/2675 and anteroposteriorly long in BP/1/5066 (MDE, 5 March 2014).;

{31 246 Not_absolutely_sure.__Some_specimens_might_be_closer_to_1,_and_apparently_variable_(Evans,_1986:_186-187_and_figures_4,_5).;

{31 251 Based on BPI/1/2675 and GHG 431 (MDE, 12 October 2012).;

{31 267 Based on BP/1/2675 (MDE, 5 March 2014).;

{31 294 Based on BP/1/2675 (MDE, 23 September 2014).;

{31 298 Modesto_&_Sues_2004._(DM);

{31 308 Based on SAM-PK-K10797 (MDE, 5 March 2014).;

{31 314 Based on BPI/1/2675 (MDE, 13 September 2012).;

{31 315 Based on BPI/1/2675 (MDE, 13 September 2012).;

{31 316 Based on BP/1/2675 (MDE, 13 September 2012).;

{31 317 Based on BPI/1/2675 (MDE, 13 September 2012).;

{31 319 The posterior cervical and anterior dorsal vertebrae of BPI/1/2675 have mammillary processes on the lateral surface of the neural spines. Accordingly, the scoring of this character was changed from (0) to (1) (MDE, 14 September 2012).;

{31 330 Ratio=3.26 in the fourth cervical 3.68 in the fifth cervical vertebrae of BPI/1/2675 (MDE, 13 September 2012).;

{31 337 A transpostzygapophyseal lamina is present in the third cervical, but not in the fourth and fifth cervicals (BP/1/2675) (MDE, 2 April 2015).;

{31 345 Based on Gow (1975: 107) (MDE, 5 March 2014).;

{31 356 Ratio=0.55 based on BP/1/2675 vertebra 10 (MDE, 2 April 2013, 6 March 2014).;

{31 391 The supraglenoid foramen is absent in BP/1/2675. Accordingly, the scoring of this character was changed from (?) to (0) (MDE, 11 August 2013).;

{31 405 The interclavicle of Prolacerta lacks an anterior process (BP/1/2675). Accordingly, the scoring of this character was changed from (0) to (1) (MDE, 11 August 2013).;

{34 18 Based on Tatarinov (1978: 508) (MDE, 25 March 2014).;

{34 35 Based on Tatarinov (1978: 508) (MDE, 2 April 2015).;

{34 41 Based on Tatarinov (1978: 510) (MDE, 2 April 2015).;

{34 115 Based on Tatarinov (1978: 508) (MDE, 2 April 2015).;

{34 119 Based on Tatarinov (1978: 508) (MDE, 25 March 2014).;

{34 124 The condition is not consistent between Tatarinov (1978) and Benton & Allen (1997) (MDE, 25 March 2014).;

{34 136 The condition is not consistent between Tatarinov (1978) and Benton & Allen (1997) (MDE, 25 March 2014).;

{34 138 The condition is not consistent between Tatarinov (1978) and Benton & Allen (1997) (MDE, 25 March 2014).;

{34 144 The condition is not consistent between Tatarinov (1978) and Benton & Allen (1997) (MDE, 25 March 2014).;

{34 145 The condition is not consistent between Tatarinov (1978) and Benton & Allen (1997) (MDE, 25 March 2014).;

{34 147 The condition is not consistent between Tatarinov (1978) and Benton & Allen (1997) (MDE, 25 March 2014).;

{34 163 Based on Tatarinov (1978: 508) (MDE, 2 April 2015).;

{34 182 Based on Tatarinov (1978: 510) (MDE, 2 April 2014).;

{34 184 Based on Tatarinov (1978: 509) (MDE, 2 April 2015).;

{34 191 Based on Benton and Allen (1997: 936) (MDE, 2 April 2014).;

{34 198 Based on Tatarinov (1978: 510) (MDE, 2 April 2014).;

{34 257 The sphenethmoid described by Tatarinov (1978: 510) seems to have been too anterior to be a laterophenoid (MDE, 2 May 2014).;

{34 260 Based on Tatarinov (1978: 510) (MDE, 2 April 2014).;

{34 263 Based on Tatarinov (1978: 510) (MDE, 2 April 2014).;

{34 266 Based on Benton and Allen (1997) (MDE, 19 September 2014).;

{34 304 Based on personal observation of the cast of the skull (MDE, April 7 2015).;

{34 326 Based on Benton & Allen (1997: 936) (MDE, 25 March 2014).;

{34 349 Based on Tatarinov (1978: 511) (MDE, 2 April 2015).;

{34 365 Intercentra were probably absent in the dorsal series, but the condition cannot be determined confidently (MDE, April 12 2015).;

{34 396 Based on Tatarinov (1978: 511) (MDE, 25 March 2014).;

{34 404 Based on Tatarinov (1978: 511) (MDE, 25 March 2014).;

{34 408 Based on Tatarinov (1978: 511) (MDE, 25 March 2014).;

{34 412 Based on Tatarinov (1978: 511) (MDE, 25 March 2014).;

{34 453 Based on Tatarinov (1978: 511) (MDE, 25 March 2014).;

{37 18 In SAM-PK-K10603 the posttemporal fenestra cannot be observed, but it may be a result of the strong dorsoventral artificial compression suffered by the skull. In RC 846 the posttemporal fenestra is present as a foramen (MDE, 5 September 2014).;

{37 65 Based on CT data of BSPG 1934 VIII 514 (MDE, 5 September 2014).;

{37 88 Based on RC 846 (MDE, 5 September 2014).;

{37 89 Based on RC 846 (MDE, 5 September 2014).;

{37 115 In large specimens of Proterosuchus fergusi (e.g. SAM-PK-K10603) the fr-p suture forms a right to obtuse angle to parasagittal plane, but in smaller specimens (e.g. RC 59) the fr-p suture forms an acute angle, similar to Prolacerta (Modesto & Sues, 2004: fig. 4). Thus, this feature seems to be related to intraspecific variation (probably ontogenetic) in Proterosuchus fergusi (MDE, 19 June 2012).;

{37 129 The posterior process of the postorbital extends beyond the posterior margin of the supratemporal fenestra in Proterosuchus fergusi (e.g. BP/1/4016 and SAM-PK-K10603) (MDE, 19 June 2012).;

{37 156 Both supratemporals are clearly present in SAM-PK-K10603 and SAM-PK-K140 (MDE, 19 June 2012).;

{37 159 Proterosuchus fergusi possesses intraspecific variation for this character, in large specimens (e.g. SAM-PK-K10603) the parietal does not extend over the interorbital region, but in smaller specimens (e.g. RC 59) the parietal considerably extends over the interorbital region. Probably it is related to an ontogenetic variation (MDE, 19 June 2012).;

{37 163 In only one specimen of Proterosuchus fergusi I have seen a very small pineal foramen (BP/1/3993). In other well-preserved specimens the pineal foramen is definitely absent (e.g. RC 59, SAM-PK-K10603). So, this character seems to be polymorphic, as in Prolacerta broomi (MDE, 19 June 2012).;

{37 164 Scoring based on BP/1/3993 (MDE, 19 June 2012).;

{37 208 Based on BSPG 1934 VIII 514 and SAM-PK-K1603 (MDE, 5 September 2014).;

{37 245 Based on BP/1/3993 (MDE, 23 September 2014).;

{37 293 Based on RC 846 (MDE, 5 September 2014).;

{37 294 e.g. BSPG 1934 VIII 514 (MDE, 23 September 2014).;

{37 313 Based on SAM-PK-11208 (MDE, 5 September 2014).;

{37 314 Based on SAM-PK-K140 and GHG 363 (MDE, 12 September 2012).;

{37 317 SAM-PK-11208 has a thick postzygodiapophyseal lamina but other specimens (e.g. SAM-PK-K140 lack this lamina) (MDE, 12 September 2012).;

{37 336 The fifth to eight cervical vertebrae of Proterosuchus fergusi possess an excavation lateral to the neural spine, which is shallow in the middle cervicals and very deep in posterior cervicals, particularly in the eight cervical (BP/1/3993). These excavations are also present in Prolacerta broomi (MDE, 20 June 2012).;

{37 349 The presence of this accesory process is clear in BSPG 1934 VIII 514 (MDE, 20 June 2012).;

{37 352 Scoring based on SAM-PK-K140 (MDE, 20 June 2012).;

{37 358 Based on SAM-PK-K140 y GHG 363 (MDE, 5 September 2014).;

{37 406 Based on GHG 363 (MDE, 12 September 2012).;

{37 439 Based on SAM-PK-K140 (MDE, 6 September 2014).;

{37 563 The complete length of the fourth digit is not known in Proterosuchus specimens (MDE, 20 June 2012).;

{38 62 Most of the suture between maxilla and jugal cannot be diserned possibly because of the partial fusion between both bones (NMQR 880) (MDE, 24 March 2014).;

{38 149 Based on the facet on the quadrate (NMQR 1484) (MDE, 24 March 2014).;

{39 329 Based on a photograph in Cruickshank (1972), the neural spine of the axis is currently broken in NMQR 1484 (MDE, 11 May 2015).;

{40 575 Based on the Mtt V identified as a ?calcaneum by Young (1936: fig. 13d) (MDE, 23 April 2014).;

{43 0 The absence of interdental plates is particularly evident in the anterior end of the right dentary (UTGD 54655) (MDE, 12 March 2014).;

{53 319 Proterosuchids lack a transverse expansion of the neural spine posteriorly to the vertebrae with mammillary processes and, as a result, Koilamasuchus is scored with a question mark (MDE, 5 April 2014).;

{54 28 The palatal process of the maxilla is distinctly anteroventrally oriented and in other basal archosauriforms the palatal process of the premaxilla forms a ventrally facing obtuse angle with the alveolar margin of the bone. As a result, the premaxilla of Kalisuchus should have been downturned, resembling the condition in proterosuchids and Sarmatosuchus (MDE, 18 September 2014).;

{55 325 The atlantal intercentrum is preserved, based on unpublished photographs (MDE, 3 April 2015).;

{56 43 The holotype of Sarmatosuchus seems to be non-fully grown specimen because of the presence of an open neurocentral suture in the dorsal centrum (MDE, 9 March 2014).;

{56 221 Although scored in the data matrix of Gower and Sennikov (1997: character 34), they say in the text that no trace of the hypoglosal foramina can be detected (2 April 2014).;

{56 249 Based on scoring of Gower and Sennikov (1997: character 21) (MDE, 31 March 2014).;

{56 358 Hyposphene is present in the ninth presacral and an isolated dorsal neural arch (MDE, 13 March 2014).;

{56 383 The type specimen of Sarmatosuchus is not a mature individual (MDE, 13 March 2014).;

{57 293 There is an area filled with matrix that may represent a medial foramen on the articular, but it cannot be determined confidently (MDE, 12 April 2014).;

{62 226 Based on BP/1/6232aa (MDE, 29 April 2014).;

{62 293 Based on NMQR 3051 (MDE, 26 August 2014).;

{63 21 Based on BP/1/5207 and NHMUK R5392 (MDE, 21 June 2012).;

{63 24 Based on the right side of BP/1/5207 (MDE, 21 March 2014).;

{63 28 Based on the articulated skull BP/1/5207 (MDE, 21 June 2012).;

{63 82 Based on the left side of BP/1/5207 (MDE, 21 March 2014).;

{63 103 In BP/1/5207 the base of the posterior process of the jugal seems to be semi-elliptical, but it is not the case in BP/1/3893 (MDE, 10 April 2014).;

{63 113 The orbital margin of the frontal is narrow (MDE, 22 June 2012).;

{63 163 In well-preserved specimens of Erythrosuchus africanus (e.g. NM QR 1473) the pineal foramen is absent (MDE, 22 June 2012).;

{63 191 Based on Gower (2003: 31) (MDE, 16 September 2012).;

{63 204 Based on Gower (2003) (MDE, 21 March 2014).;

{63 208 Based on Gower (1997: 560) (MDE, 21 March 2014).;

{63 221 Based on Gower (1996: 560) (MDE, 21 March 2014).;

{63 251 Based on Gower (1997: fig. 4a) (MDE, 23 March 2014).;

{63 293 Based on Gower (2003: 35) (MDE, 26 August 2014).;

{63 298 It was added the fifth state. ' thecodont' to that of the data matrix of Reisz et al. (2010) for Erythrosuchus africanus (MDE, 20 June 2012).;

{63 326 Based on SAM-PK-3028 (MDE, 24 March 2014).;

{63 342 Based on NHMUK R3592 (MDE, 12 September 2012).;

{63 352 Based on NHMUK 3592 (MDE, 24 August 2012).;

{63 356 Ratio=0.85 based on NHMUK R3592 (MDE, 2 April 2013).;

{63 358 Based on NHMUK R3592 (MDE, 24 March 2014).;

{63 403 Since none of the preserved specimens of Erythrosuchus possess an articulated pectoral girdle I consider that the presence or absence of a cleithrum cannot be determined (MDE, 24 August 2012).;

{63 412 Since none of the preserved specimens of Erythrosuchus possess an articulated pectoral girdle I consider that the presence or absence of a mineralized sternum cannot be determined (MDE, 24 August 2012).;

{64 50 Based on Wang et al. (2013: fig 2a) (MDE, 27 March 2014).;

{64 217 Based on Gower and Sennikov (1996) (MDE, 26 March 2015).;

{64 249 Based on Gower and Sennikov (1996: 896) (MDE, 31 March 2014).;

{64 345 I am not sure if the elements described by Wang et al. (2013) in the neck of S. shansisuchus are actually intercentra. They are too large and overlap considerably the lateral surface of the centra (MDE, 27 March 2014).;

{64 355 Based on Young (1964: fig. 21f) (MDE, 27 March 2014).;

{64 356 ratio=1.13 based on Young (1964: fig. 21f) (MDE, 27 March 2014).;

{64 538 The identification of the posterior groove by Gower (1996) was tentative (MDE, 27 March 2014).;

{65 32 Based on Gower (2003: 15) (MDE, 25 September 2014).;

{71 208 Based on Nesbitt et al. (2009: 826) (MDE, 25 April 2014).;

{71 210 Based on Nesbitt et al. (2009: 826) (MDE, 25 April 2014).;

{71 221 Based on Nesbitt et al. (2009: 826) (MDE, 25 April 2014).;

{71 446 Based on Nesbitt et al. (2009: 831) (MDE, 27 April 2014).;

{72 36 The posterior margin of the base of the postnarial process is broken off, but the facet for its reception on the maxilla shows that it was plate-like (MDE, 1 October 2014).;

{72 169 Nesbitt et al. (2013: 11) (MDE, 25 September 2014).;

{73 0 Based on UMZC T6921 (MDE, 28 March 2014).;

{73 24 SAM-PK-6048 lacks a subnarial foramen, but SAM-PK-6047 possesses a subnarial foramen formed by a notch on the maxilla (MDE, 31 March 2014).;

{73 51 The anterior maxillary foramen is absent in SAM-PK-13665 but it is present in other specimens (Gow 1970) (MDE, 16 September 2012).;

{73 62 Based on SAM-PK-6047A and the left side of SAM-PK-5867 (MDE, 18 September 2014).;

{73 65 Based on UMZC T692 and Gow (1970) (MDE, 28 March 2014).;

{73 66 Based on SAM-PK-6050 (MDE, 21 October 2014).;

{73 67 The ventral margin of the maxilla is slightly convex in SAM-PK-5867 (MDE, 11 September 2012).;

{73 88 Based on Senter (2003) (MDE, 28 March 2014).;

{73 140 Based on the left squamosal of SAM-PK-5867 (MDE, 28 March 2014).;

{73 186 Based on Gow (1970) (MDE, 14 February 2014).;

{73 201 Based on SAM-PK-13664 (MDE, 27 August 2012).;

{73 210 Based on SAM-PK-7696 (MDE, 28 March 2014).;

{73 212 Based on SAM-PK-7696 (MDE, 28 March 2014).;

{73 213 Based on SAM-PK-5867 (MDE, 14 September 2012).;

{73 220 Based on Gower and Weber (1998: 374) (MDE, 18 August 2014).;

{73 236 Based on SAM-PK-5867 and SAM-PK-7696 (cf. Gower and Weber, 1998, contra Nesbitt, 2011) (MDE, 19 April 2014).;

{73 237 Based on Gower and Weber (1998) (MDE, 22 August 2014).;

{73 239 The foramina for the entrance of the internal carotid are positioned ventrally in the basisphenoid in UMZC T692 (MDE, 11 September 2012).;

{73 248 Based on Gower and Weber (1998: 379) (MDE, 31 March 2014).;

{73 249 Based on Gower and Weber (1998: 377) (MDE, 31 March 2014).;

{73 255 Based on SAM-PK-7696 (MDE, 16 March 2015).;

{73 256 Based on Gower and Weber (1998: 379) (MDE, 31 March 2014).;

{73 271 The base of a posterodorsal process seems to be present in SAM-PK-5867 (MDE, 31 March 2014).;

{73 293 Based on UMZC T6921 (MDE, 31 March 2014).;

{73 317 Based on SAM-PK-6047B (MDE, 2 April 2014).;

{73 345 Based on Ewer (1965: 406) (MDE, 31 March 2014).;

{73 348 Based on SAM-PK-13665 (MDE, 31 March 2014).;

{73 356 Ratio=0.48 based on Ewer (1965: fig. 7g, h) (MDE, 2 April 2013).;

{73 365 Based on Ewer (1965: 406) (MDE, 31 March 2014).;

{73 372 The sacral rib of the second sacral of Euparkeria is not bifurcated (SAM-PK-7696) (MDE, 11 September 2012).;

{73 405 I consider that the anterior portion of the interclavicle of the holotype specimen is not enough preserved to assessed the condition of the character (MDE, 24 August 2012).;

{73 408 I consider that the anterior portion of the interclavicle of the holotype specimen is not enough preserved to assessed the condition of the character (MDE, 24 August 2012).;

{73 426 In the holotype of Euparkeria there is no supinator ridge neither a deep groove in the distal end of the right humerus (MDE, 24 August 2012).;

{73 449 Based on SAM-PK-13666 (MDE, 11 October 2012).;

{73 451 Based on SAM-PK-13666 (MDE, 31 March 2014).;

{73 540 Nesbitt (2011) scored Euparkeria as (1), but the posterior portion of the dorsolateral margin of the astragalus overlaps the calcaneum to the same extent as the anterior portion (UMCZ T692) (MDE, 25 September 2014).;

{73 587 Based on SAM-PK-6048 (MDE, 1 April 2014).;

{75 18 Based on Dilkes and Arcucci (2012: 17) (MDE, 13 April 2014).;

{75 191 Based on Dilkes and Arcucci (2012: 16) (MDE, 14 April 2014).;

{75 220 The contact between exoccipitals on the floor of the endocranial cavity is uncertain (Trotteyn and Haro 2010, Dilkes and Arcucci 2012) (MDE, 14 April 2014).;

{75 224 Based on PVL 2063 (MDE, 14 April 2014).;

{75 237 Based on Dilkes and Arcucci (2012: 18) (MDE, 19 April 2014).;

{75 239 Based on Dilkes and Arcucci (2012: 18) (MDE, 19 April 2014).;

{75 247 Based on Dilkes and Arcucci (2012: 18) (MDE, 14 April 2014).;

{75 372 Based on Trotteyn (2011: 433) (MDE, 14 April 2014).;

{75 421 Trotteyn (2011) described that the shape of the deltopectoral crest cannot be determined because of postmortem deformation (MDE, 14 April 2014).;

{77 13 In the holotype the anterior border of the antorbital fenestra is pointed, whereas in PVL 4606 is gently rounded (MDE, 7 July 2014).;

{77 41 Based on the left premaxilla of PVL 4601 (MDE, 7 July 2014).;

{77 75 The suture between the frontals and parietals cannot be confidently traced in available specimens (MDE, 8 July 2014).;

{77 86 Based on PVL 4606 (MDE, 8 July 2014).;

{77 95 Based on PVL 4606 (MDE, 8 July 2014).;

{77 329 The dorsal margin of the neural spine of the axis is damaged in PVL 4601 and PVL 4602 (MDE, 19 October 2014).;

{77 576 Based on PVL 4606 (MDE, 26 September 2014).;

{78 41 Based on Trotteyn et al. (2013) (MDE, 9 July 2014).;

{79 363 Based on Dilkes and Arcucci (2012) character 55 (MDE, 18 August 2014).;

{80 237 Based on Nesbitt (2011: 87) (MDE, 19 April 2014).;

{80 288 The posterior surangular foramen is present in MCZ 4037 but it seems to be absent in PULR 07 and PVL 4586 (MDE, 22 April 2014).;

{80 329 The dorsal margin of the neural spine of the axis is mostly straight in MCZ 4037 and dorsally convex in PVL 4575 (MDE, 19 October 2014).;

{81 15 The skull is strongly dorsoventrally compressed beause of taphonomic processes and the character cannot be confidently scored (MDE, 18 August 2014). ;

{81 43 The premaxillary teeth are lateroventrally oriented, but it may be a result of the strong dorsoventral compressio suffered by the skull (PVSJ 567) (MDE, 28 April 2014).;

{81 130 The skull is strongly dorsoventrally compressed beause of taphonomic processes and the character cannot be confidently scored (MDE, 18 August 2014). ;

{81 266 The specimen is too artificially compressed to determine the character-state (MDE, 19 September 2014).;

{83 239 The entrance for the internal carotid seems to have been labelled as CN VI by Desojo et al. (2011) (MDE, 19 April 2014).;

{84 501 Based on MCZ 4077 (MDE, 28 April 2014).;

{84 502 Based on MCZ 4077 (MDE, 28 April 2014).;

{84 503 Based on MCZ 4077 (MDE, 28 April 2014).;

{84 504 Based on MCZ 4077 (MDE, 28 April 2014).;

{84 505 Based on MCZ 4077 (MDE, 28 April 2014).;

{84 507 Based on MCZ 4077 (MDE, 28 April 2014).;

{85 331 The degree of separation between parapophyses and diapophyses is not clear because of deformation (SMNS 91083) (MDE, 28 April 2014).;

{85 509 The femur of SMNS 91002 seems to be strongly artificially compressed (MDE, 28 April 2014).;

{85 559 The bones interpreted by Schoch and Sues (2013) as astragalus and calcaneus are reinterpreted as distal tarsals 3 and 4 (MDE, 28 April 2014).;

{88 248 The suture between supraoccipital and prootic cannot be discerned (MDE, 22 January 2015).;

{88 255 Poorly preserved in the inferior anterior process (ISI, MDE 22 September 2015).;

{89 0 Nicrosaurus kapffi: NHMUK R38036, 38037, 42743, 42744, 42745, Huene 1923, Hungerbühler 1998, 2000; Nicrosaurus sp. Hungerbühler, 1998 (MDE;

{89 288 Based on NHMUK R38036, 42744 (MDE, 16 March 2015).;

{90 53 Based on UCMP 27200 and Nesbitt (2011: 96) (MDE, 6 May 2014).;

{90 95 Based on UCMP 27200, MCZ 1029 and Camp (1930: fig. 11) (MDE, 6 May 2014).;

{91 65 Based on Walker (1964: 72) (MDE, 20 August 2014).;

{91 86 Based on Walker (1964: fig. 3a) (MDE, 20 August 2014).;

{91 152 Based on the facet along the entire ventral margin of the posterior process of the jugal present in NHMUK R3142 (MDE, 20 August 2014).;

{91 353 Based on Walker (1964: 82) (MDE, 20 August 2014).;

{91 472 Based on NHMUK R2410 and Walker 1964: fig. 11g (MDE, 20 August 2014).;

{92 4 The dorsal surface of the skull of available specimens is too damaged to determine the state of this character (MDE, 7 May 2014).;

{92 461 In PVL 3828 there is a crest cofluent with the anterior border of the preacetabular process, but it is absent in PVL 3826 (MDE, 20 August 2014).;

{92 464 The depression on the ventral surface of the base of the postacetabular process in PVL 3827 does not reach the distal end of the process, contrasting with the condition in dinosauriforms (MDE, 20 August 2014).;

{92 472 The pubis has a groove in PVL 3827 (MDE, 20 August 2014).;

{93 358 Based on Nesbitt et al. (2014: 1364) (MDE, 11 November 2014).;

{93 384 The condition of this character cannot be confidently determined because the anterior margin of the coracoid is misssing (MDE, 11 November 2014).;

{93 455 Based on the orientation of the sacral rib facets and in agreement with the scoring of Nesbitt et al. (2014: character 270) (MDE, 11 November 2014).;

{93 507 Based on Nesbitt et al. (2014: character 323) (MDE, 11 November 2014).;

{93 522 Probably miscored by Nesbitt et al. (2014: character 337) (MDE, 11 November 2014).;

{93 528 Although scored as a low crest-shaped structure by Nesbitt et al. (2014: character 339), the tubercle is considerably better developed than in non-pseudosuchian archosauromorphs (MDE, 11 November 2014).;

{93 535 The condition of this character is extremely similar to that of Smilosuchus and Parasuchus (MDE, 10 November 2014).;

{94 24 Based on Butler et al. (2014) (MDE, 22 August 2014).;

{94 326 Based on Wu and Russell (2001: 44) (MDE, 22 April 2014).;

{94 369 Based on Wu and Russell (2001: 45) (MDE, 22 April 2014).;

{95 58 Both character-states are present in MCZ 4117 (MDE, 18 September 2014).;

{95 65 Based on Lecuona (2013) and Butler et al. (2014) (MDE, 22 August 2014).;

{95 257 Based on the right side of MCZ 4117 (MDE, 22 August 2014).;

{95 283 Based on MCZ 4116 (MDE 5 October 2014).;

{95 293 Based on MCZ 4116 (MDE 5 October 2014).;

{95 294 Based on MCZ 4116 (MDE 5 October 2014).;

{97 88 Based on Gower (1999: 39) (MDE, 22 April 2014).;

{97 219 Based on Gower (2002) (MDE, 19 September 2014).;

{97 535 Inferred from the morphology of the distal end of tibia (MDE, 9 October 2014);

{98 64 The dorsal process is present in UFRGS-PV-0152-T and the dorsal prong of the anterior tip of the jugal figured by Parrish 1993 for UFRGS-PV-0156-T might be a dorsal proess extending from the maxilla (MDE, 4 September 2014).;

{98 208 Based on Mastrantonio et al. (2013) (MDE, 4 September 2014).;

{98 219 Based on Mastrantonio et al. (2013) (MDE, 19 September 2014).;

{98 249 Based on Mastrantonio et al. (2013) (MDE, April 12 2015).;

{101 370 Based on Bonaparte (1975: fig. 6) (MDE, 19 April 2014).;

{101 535 The supposed second basin on the astragalus, if present, is placed posterolateral rather than posteromedial (PVL 3871), contrasting with Riojasuchus, suchians and Lagerpetidae (MDE, 20 October 2014).;

{102 220 Based on Bittencourt et al. (2014: 8) (MDE, 21 August 2014).;

{104 257 Inferred from the well-preserved and defined facet on the ventral surface of the skull roof of ZPAL Ab IIII 1223 (MDE, April 20 2015).;

{104 356 Based on ZPAL Ab III 1930 (MDE, April 23 2015);

{104 379 Based on ZPAL Ab III 1975 (MDE, 30 April 2015).;

{104 380 Based on ZPAL Ab III 1975 (MDE, 30 April 2015).;

{104 465 Based on ZPAL Ab III 364 (MDE, April 23 2015);

{104 484 Based on Nesbitt (2011: 140) (MDE, 24 April 2014).;

{104 544 The calcaneal tuber is incipient in Silesaurus (ZPAL Ab III 361) (MDE, April 23 2015);

{105 14 The secondary antorbital fenestra occurs is defined by the premaxilla, maxilla and sometimes nasal. In Heterodontosaurus the accesory antorbital fenestra is completely enclosed by the maxilla within the antorbital fossa and, as a result, is not considered homologous to the former (MDE, 27 August, 2014).;

{105 287 Based on Sereno (2012: 107) (MDE, 27 August 2014).;

{105 288 Based on Sereno (2012: 107) (MDE, 27 August 2014).;

{105 412 The bones interpreted by Sereno (2012) as sternal plates are likely uncinate processes (MDE, 27 August 2014).;

{106 269 Based on MACN-Pv 18060 (MDE, 26 August 2014).;

{106 382 Based on PVL 2054 (MDE, 26 August 2014).;

{106 443 This scorings is based on the reinterpretation of the distal carpals preserved in PVSJ 373 as dc2-5 instead of dc1-4 (MDE, April 13 2015).;

{106 575 Based on PVSJ 373 (MDE, 13 October 2014).;

{107 536 Inferred from tibial morphology (MDE, April 14 2015).;

;
